# ﻿A new species of scops-owl (Aves, Strigiformes, Strigidae, *Otus*) from Príncipe Island (Gulf of Guinea, Africa) and novel insights into the systematic affinities within *Otus*

**DOI:** 10.3897/zookeys.1126.87635

**Published:** 2022-10-30

**Authors:** Martim Melo, Bárbara Freitas, Philippe Verbelen, Sátiro R. da Costa, Hugo Pereira, Jérôme Fuchs, George Sangster, Marco N. Correia, Ricardo F. de Lima, Angelica Crottini

**Affiliations:** 1 MHNC-UP, Museu de História Natural e da Ciência da Universidade do Porto, Praça Gomes Teixeira, 4050-368 Porto, Portugal Gulf of Guinea Biodiversity Centre São Tomé São Tomé and Príncipe; 2 CIBIO, Centro de Investigação em Biodiversidade e Recursos Genéticos, InBIO Laboratório Associado, Campus de Vairão, Universidade do Porto, 4485-661 Vairão, Portugal Museu de História Natural e da Ciência da Universidade do Porto Porto Portugal; 3 FitzPatrick Institute of African Ornithology, University of Cape Town, Private Bag X3, Rondebosch 7701, Cape Town, South Africa Universidade do Porto Vairao Portugal; 4 Gulf of Guinea Biodiversity Centre, São Tomé, São Tomé and Príncipe BIOPOLIS Program in Genomics, Biodiversity and Land Planning, CIBIO Vairão Portugal; 5 BIOPOLIS Program in Genomics, Biodiversity and Land Planning, CIBIO, Campus de Vairão, 4485-661 Vairão, Portugal University of Cape Town Cape Town South Africa; 6 Departamento de Biologia, Faculdade de Ciências, Universidade do Porto, 4099-002 Porto, Portugal Universidade do Porto Porto Portugal; 7 MNCN-CSIC, National Museum of Natural Sciences, Spanish National Research Council, Calle José Gutiérrez Abascal 2, Madrid 28006, Spain National Museum of Natural Sciences Madrid Spain; 8 EDB, Laboratory of Evolution and Biologic Diversity, UMR 5174 CNRS-IRD, University of Toulouse III Paul Sabatier, 118 route de Narbonne, 31062 Toulouse, France University of Toulouse III Paul Sabatier Toulouse France; 9 Krijgsgasthuisstraat 89, Ghent, Belgium Unaffiliated Ghent Belgium; 10 Praia Lapa, Príncipe, São Tomé and Príncipe, São Tomé and Príncipe Unaffiliated Príncipe São Tomé and Príncipe; 11 Department of Animal Behaviour, Bielefeld University, 33501 Bielefeld, Germany Universidade do Porto Vairão Portugal; 12 Institut de Systématique, Evolution, Biodiversité (ISYEB), Muséum national d’Histoire naturelle, CNRS, SU, EPHE, UA CP51, 57 rue Cuvier, 75005 Paris, France Bielefeld University Bielefeld Germany; 13 Naturalis Biodiversity Center, Darwinweg 2, PO Box 9517, 2300, RA, Leiden, Netherlands Muséum national d’Histoire naturelle Paris France; 14 Department of Bioinformatics and Genetics, Swedish Museum of Natural History, Box 50007, 10405 Stockholm, Sweden Naturalis Biodiversity Center Leiden Netherlands; 15 LIDA, School of Arts and Design, Polytechnic of Leiria, Rua Isidoro Inácio Alves de Carvalho, 2500-321 Caldas da Rainha, Leiria, Portugal Swedish Museum of Natural History Stockholm Sweden; 16 Centre for Ecology, Evolution and Environmental Changes (cE3c), Faculdade de Ciências, Universidade de Lisboa, 1749-016 Lisboa, Portugal School of Arts and Design Leiria Portugal; 17 Departamento de Biologia Animal, Faculdade de Ciências, Universidade de Lisboa, 1749-016 Lisboa, Portugal Universidade de Lisboa Lisboa Portugal

**Keywords:** Biodiversity, endemism, exploration, Gulf of Guinea, integrative taxonomy, *Otusbikegila* sp. nov., Prin­cipe Scops-Owl, systematics, Biodiversidade, endemismo, exploração, Golfo da Guiné, Kitóli-do-príncipe, Mocho-do-príncipe, *Otusbikegila* sp. nov., sistemática, taxonomia integrada

## Abstract

A new species of scops-owl (Aves, Strigiformes, Strigidae, *Otus*) is described from Príncipe Island, São Tomé and Príncipe (Gulf of Guinea, Africa). This species was discovered for science in 2016, although suspicions of its occurrence gained traction from 1998, and testimonies from local people suggesting its existence could be traced back to 1928. Morphometrics, plumage colour and pattern, vocalisations, and molecular evidence all support the species status of the scops-owl from Príncipe, which is described here as *Otusbikegila***sp. nov.** Phylogenetic analyses suggest that this species descended from the first colonisation of the Gulf of Guinea islands, being sister to the clade including the mainland African Scops-Owl *O.senegalensis*, and the island endemics Sao Tome Scops-Owl *O.hartlaubi* and Pemba Scops-Owl *O.pembaensis*. The most diagnostic trait in the field is its unique call which, curiously, is most similar to a distantly related *Otus* species, the Sokoke Scops-Owl *O.ireneae*. The new species occurs at low elevations of the old-growth native forest of Príncipe, currently restricted to the south of the island but fully included within Príncipe Obô Natural Park. *Otusbikegila***sp. nov.** takes the number of single-island endemic bird species of Príncipe to eight, further highlighting the unusually high level of bird endemism for an island of only 139 km^2^.

## Abstract (pg)

Descrevemos uma nova espécie de mocho-de-orelhas ou kitóli (Strigiformes: Strigidae: *Otus*) da Ilha do Príncipe, São Tomé e Príncipe (Golfo da Guiné, África). Esta espécie foi descoberta para a ciência apenas em 2016, embora suspeitas da sua existência tenham ganho força a partir de 1998, e testemunhos de habitantes locais sobre a sua ocorrência já estarem documentados em 1928. A morfometria, a cor e padrão da plumagem, as vocalizações e dados moleculares demonstram que esta população de mocho no Príncipe é uma espécie nova, que foi batizada de mocho-do-príncipe (lista mundial) ou kitóli-do-príncipe (nome nacional), *Otusbikegila***sp. nov.** As análises filogenéticas indicam que esta espécie descende da primeira colonização das ilhas do Golfo da Guiné, sendo irmã do clado que inclui o mocho-d’ore­lhas-africano *O.senegalensis*, do continente, o mocho-de-são-tomé (ou kitóli-de-são-tomé) *O.hartlaubi* e o mocho-de-pemba *O.pembaensis*, ambos endémicos das ilhas que lhes dão o nome. No campo, a característica mais diagnóstica é o seu canto único que, curiosamente, é mais parecido com o da espécie de *Otus* mais afastada, o mocho-de-sokoke *O.ireneae*. A nova espécie ocorre nas zonas baixas da floresta nativa do Príncipe, atualmente restrita ao sul da ilha, mas totalmente inserida no Parque Natural do Obô do Príncipe. *Otusbikegila***sp. nov.** eleva o número de espécies de aves endémicas restritas ao Príncipe para oito, sublinhando ainda mais o nível extremamente elevado de aves endémicas para uma ilha de apenas 139 km^2^.

“*But I discovered that the very same aggregations or groupings of individuals that the trained zoologist called separate species were called species by the New Guinea natives. I collected 137 species of birds. The natives had 136 names for these birds (…)*”

Ernst Mayr - Interview - Omni Magazine, February 1983

## ﻿Introduction

Species are indeed the face of biodiversity with whom everyone relates to. The disco­very of new species consistently makes headlines expressing wonder and joy. And yet, it has been estimated that only ca. 14% of extant species have been described, with invertebrates making most of the undescribed species (Mora et al. 2011). In this age of human-driven extinction (Ceballos et al. 2020), a major global effort should be undertaken to document what may soon not be anymore (Dijkstra 2016). Such new wave of exploration, carried out by professional and amateurs alike, would have the additional benefit of helping to revive a global interest in the natural world and the mysteries it holds. Only by rekindling this link can the current biodiversity crisis be reverted.

The discovery of new species tends to have a higher impact when it occurs in familiar groups like mammals or birds. Birds in particular are likely the best studied animal group, making the discovery of new species more challenging and often restricted to remote locations and/or difficult-to-study groups (e.g., Rheindt et al. 2020; Lane et al. 2021; Milá et al. 2021). This paper illustrates how exploration led to the discovery of a new owl species on the forests of Príncipe Island, Gulf of Guinea, Africa.

Owls (Aves, Strigiformes) are a charismatic bird group that made their way into most human cultures, where they are generally either symbols of wisdom or, on the contrary, omens of bad luck (Marcot and Johnson 2003). This is certainly linked to their nocturnal habits and associated elusiveness, their inquisitive look enhanced by their large eyes facing forward, and their calls heard through the night, which together help in creating an aura of mystery surrounding these species. In many aspects, this mystery has also permeated for a long time the scientific knowledge we have of the group. This is strikingly illustrated by the results of the extensive efforts carried out in recent decades, which through exploration in the field (e.g., Lambert and Rasmussen 1998; Warakagoda and Rasmussen 2004; Sangster et al. 2013) and taxonomic revisions (e.g., Fuchs et al. 2008; Flint et al. 2015; Salter et al. 2020) resulted in the remarkable increase of the number of recognised species of owls from 146 in 1975 (Morony et al. 1975) to up to 230 species in 2021 (Gill et al. 2021). This dramatic increase was supported by the widespread adoption of an integrative taxonomic framework (Padial et al. 2010; Sangster 2018), which combines the use of multiple lines of evidence, such as genetics, morphology, acoustics, geography and behaviour to reach informed decisions on the species status of a given taxon (see also Cadena and Zapata 2021).

Still, compared to other groups, the current discrepancy in the number of owl species accepted by different authorities highlights the challenges associated with the taxo­nomy and systematics of this group. This stems from their nocturnal habits, making them difficult to study, and from being a group where, at the generic level, morphological variation between species can be similar or lower than within-species (Marks et al. 1999). This is because, in owls, the evolution of plumage pattern and colour is driven by the pressure to remain cryptic during daytime as a defence against predators or to avoid being mobbed by other birds, a common occurrence among members of the family (Marks et al. 1999; König et al. 2008). This led to the convergent evolution of similar camouflaged patterns across species, as happens in other nocturnal bird groups like the nightjars (Caprimulgidae: Holyoak 2001). As such, plumage is generally not diagnostic in owls, with distantly related species often being strikingly similar (Marks et al. 1999). This morphological uniformity is especially evident, and taxonomically challenging, in the most speciose genus of the family: *Otus* Pennant, 1769, which includes over 50 recognised species, occurring across Asia, Europe, and Africa (Marks et al. 1999; König et al. 2008; Winkler et al. 2020). Commonly known as scops-owls, these small to medium-sized predators show two main plumage colour types, rufous or grey (or grey-brown) morphs, which often occur in the same populations (Pons et al. 2013).

In contrast to plumage, vocalisations of members of the Strigidae family are species-specific. As with most non-passerines and suboscine passerines, owl songs are not learned (Gahr 2000) and therefore have a strong genetic basis. Additionally, contrarily to the difficulty in observing owls, their vocalisations are conspicuous and easily detected as they play a major role in territorial defence and mate attraction (Marks et al. 1999; König et al. 2008). Vocalisations thus represent the most important trait to differentiate species of owls (e.g., Marshall 1978; Sangster et al. 2013; Flint et al. 2015), and new species are often first discovered through their calls (Melo and Dallimer 2008, 2009; Sangster et al. 2013).

The Gulf of Guinea, Central Africa, has three oceanic islands, Príncipe, São Tomé, and Annobón, in a northeast to southwest line, with São Tomé touching the equator. The rainforests of the islands constitute an independent ecoregion (Gascoigne 2004) characterised by high endemism levels across groups (Jones 1994). Endemism is particularly striking in birds, with the islands having been classified as the third most important in the world for the conservation of forest birds (Buchanan et al. 2011). Relatively to their area, the two larger islands (which together make the Democratic Republic of São Tomé and Príncipe) have by far the highest number of endemic bird species in the world (at least 28 endemic species in 996 km^2^; Melo et al. 2022).

Although birds are the best-studied group of the Gulf of Guinea islands (Jones and Tye 2006; Melo et al. 2022), the presence of a candidate species of owl on Príncipe Island was only confirmed in 2016 (Ryan 2016; Verbelen et al. 2016), following deca­des of the accumulation of evidence pointing towards it (Suppl. material 1). In this paper we confirm the distinctiveness of the population of scops-owls from Príncipe using morphometrics, plumage colouration and pattern, song, and mitochondrial and nuclear DNA sequence data. We discuss the origin of this species by placing it within a large-scale phylogeny of the genus *Otus*. Together with the new species, this phylogeny included 14 taxa never previously analysed, providing novel insights on the relationships within the most speciose genus of the Strigidae family.

## ﻿Materials and methods

### ﻿Study site

**Figure 1.**
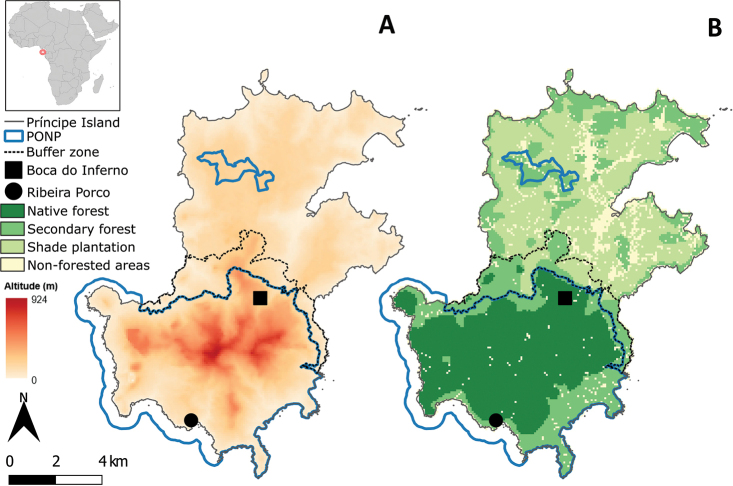
Altitudinal **A** and land use maps **B** of Príncipe Island, with the limits of the Príncipe Obô Natural Park (PONP) and its buffer zone, and the two localities where the four individuals of the candidate species of *Otus* from Príncipe were captured; inset: location of Príncipe in Africa.

Príncipe Island (1°31.80'N–1°43.20'N, 7°19.80'E–7°28.20'E) is located in the Gulf of Guinea, ca. 220 km offshore Gabon (Fig. 1). Part of the Cameroon Line of Volcanoes, its oldest sub-aerial rocks date to the origin of the line at ca. 30 Ma (Burke 2001). With a surface area of 139 km^2^ (ca. 17 km long and 8 km wide), it has a relatively flat low-lying area in the north, contrasting with the rugged central and southern region characterised by high ridges that rise up to 948 m a.s.l. at Pico do Príncipe (Jones and Tye 2006). It has an oceanic equatorial climate, with an annual precipitation that can reach 5,000 mm. Most of the accessible regions of the island were cleared and converted to plantations (Jones and Tye 2006). Some of those areas were abandoned and regenerated into secondary forest (Atkinson et al. 1991; Castanheira-Diniz and Cardoso-de-Matos 2002; Jones and Tye 2006). The remaining area is covered with two types of native forest stratified by altitude: lowland and montane rainforest, the latter being restricted to Pico do Príncipe and the surrounding summits (Exell 1944).

Fieldwork for specimen and tissue sample collection, measurements, and additional bioacoustics recordings took place in May 2017, July 2018, and January 2019. All samples and vocalisation recordings were collected within Príncipe Obô Natural Park, in the south of Príncipe (Fig. 1). Locality information was recorded using a GPS receiver (Garmin GPS Map 62s; Garmin International Inc., Olathe, Kansas, United States).

### ﻿Voucher specimen collection

**Figure 2.**
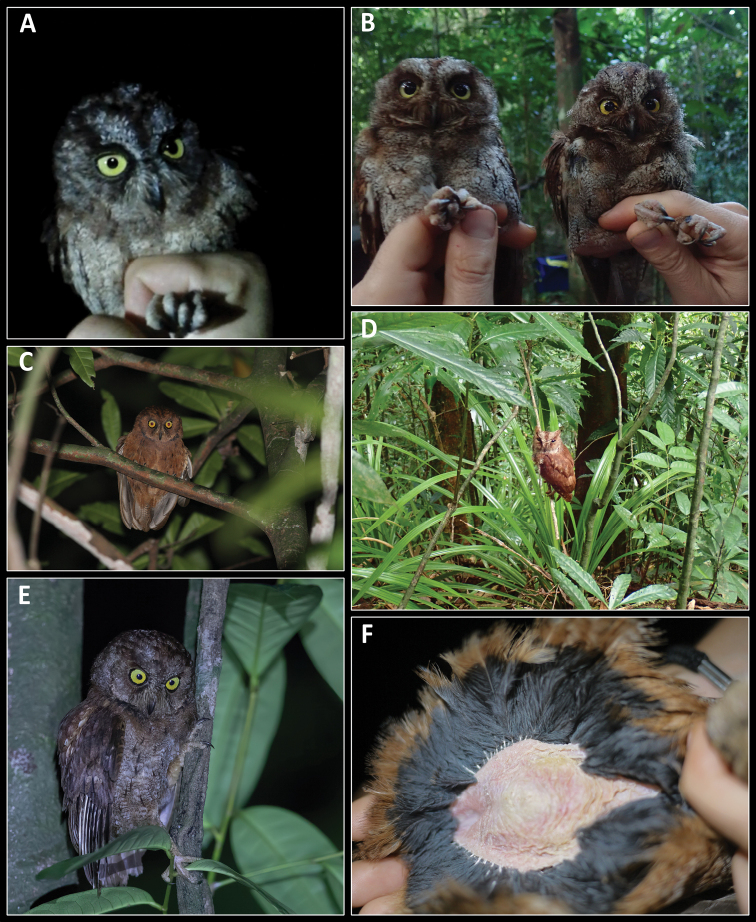
The candidate species of *Otus* from Príncipe **A** female specimen MHNC-UP-AVE7000, showing colouration in life (available also in the Macaulay library (ML): ML470442301; grey-brown morph) **B** female (left, sample P9-037) and male (right, sample P9-038) grey-brown morphs captured at Boca do Inferno on January 28, 2019 (ML470438621) **C** rufous morph individual photographed at Ribeira Porco area on July 04, 2016 **D** daytime sighting of a grey-brown morph individual between Rio São Tomé and Ribeira Porco on January 19, 2019 (ML470443361, only the rufous upperparts are clearly seen) **E** grey-brown morph individual photographed in the Ribeira Porco area on January 21, 2019 **F** fully developed brood patch of a female rufous morph (sample P8-001) captured in the Ribeira Porco area on January 20, 2019 (ML470440211). ML – Macaulay Library. Photographs: **A** – HP **B, D, F** – MM and BF **C** – PV **E** – Paul van Giersbergen.

On May 29, 2017, in the Ribeira Porco area (1°33.03'N, 7°22.29'E, Fig. 1), one individual was captured using mist-nets (Fig. 2A), measured and a blood sample collected. This individual was euthanised by inhaling an Isoflurane 1 mL/5 L solution, dissected, fixed with absolute EtOH, and preserved in 80% EtOH. Afterwards, the specimen was prepared as a study skin and spread wing, and the partial skeleton was prepared following a modified procedure (Cataldo 2017) from that described by Davis and Payne (1992) and Baker et al. (2003). Previously to being captured, recordings of its vocalisations and of those of a second bird were obtained using a recorder (Edirol R-09HR, Roland, Japan) and a microphone (MKE 400, Sennheiser, Germany); these were elicited by playing back previously recorded vocalisations of this taxon to attract it into the nets. The voucher specimen was photographed to document life colouration and appearance (Fig. 2A). The voucher was deposited in the ornithological collection of the Natural History and Science Museum of the University of Porto (Table 1).

**Table 1.** Morphological measurements of the scops-owls specimens included in the present study with their respective institutional catalogue number (superscript letters, when present, indicate: HT, holotype; F, female; M, male) and sampling locality. All measures are in millimetres. NA – not available; STP – São Tomé and Príncipe; DRC – Democratic Republic of the Congo; EG – Equatorial Guinea. Morphological measurements – Bilen: bill length from bill tip to where culmen enters feathers; Biwid: bill width; Bidepth: bill depth; Binares: bill length from the anterior end of the nares to the tip; Hebi: head+bill, from the tip of the bill to the opposite point on the back of the skull; Midt: middle toe length; Tarlen: tarsus length; Wilen: wing length; Tailen: tail length; Bolen: body length; P10-4: length of primary feathers; Wing formula: sequence of primary feathers ordered by size; * specimens not collected, blood samples codes from the collection of MM at CIBIO-InBIO.

Taxon	Catalogue number followed by tissue sample (if available)	Locality	Bilen	Biwid	Bidepth	Binares	Hebi	Midt	Tarlen	Wilen	Tailen	Bolen	P10	P9	P8	P7	P6	P5	P4	Wing formula
*O.bikegila* sp. nov. 1	MHNC-UP-AVE7000 ^HT, F^; P7-04	STP, Príncipe	16.0	9.0	11.3	11.3	38.9	19.9	32.3	147	NA	NA	NA	NA	NA	NA	NA	NA	NA	NA
*O.bikegila* sp. nov. 2	NA; P8-001 * ^F^	STP, Príncipe	19.0	10.9	10.4	11.7	44.0	NA	34.0	148	85	NA	82	104	116	122	120	119	115	7 > 6>5 > 8>4 > 9>10
*O.bikegila* sp. nov. 3	NA; P9-037 * ^F^	STP, Príncipe	19.0	11.8	11.9	12.6	NA	NA	35.1	151	85	205	84	108	118	121	120	119	115	7 > 6>5 > 8>4 > 9>10
*O.bikegila* sp. nov. 4	NA; P9-038 * ^M^	STP, Príncipe	17.4	10.9	12.1	11.6	NA	NA	30.5	145	75	192	80	103	114	118	116	113	108	7 > 6>8 > 5>4 > 9>10
*O.hartlaubi* 1	NA; ST03-294 *	STP, São Tomé	16.1	6.5	9.7	NA	NA	NA	30.8	130	67	NA	NA	NA	NA	NA	NA	NA	NA	NA
*O.hartlaubi* 2	NA; ST-R16-0202 *	STP, São Tomé	NA	NA	NA	NA	NA	NA	NA	139	NA	NA	NA	NA	NA	NA	NA	NA	NA	NA
*O.hartlaubi* 3	NA; ST10-440 *	STP, São Tomé	15.5	9.8	NA	NA	NA	NA	32.9	132	68	NA	NA	NA	NA	NA	NA	NA	NA	NA
*O.hartlaubi* 4	NA; ST-R17-0264 *	STP, São Tomé	NA	NA	NA	NA	NA	NA	27.8	134	67	NA	NA	NA	NA	NA	NA	NA	NA	NA
*O.hartlaubi*	NA; ST15-144 *	STP, São Tomé	16.1	6.3	9.1	10.1	39.0	NA	31.9	135	65	170	74	86	101	107	106	104	100	7 > 6>5 > 8>4 > 9>10
*O.hartlaubi*	SMD C50544	STP, São Tomé	17.1	6.1	NA	10.6	39.5	17.1	30.6	136	69	174	65	91	102	108	107	104	92	7 > 6>5 > 8>4 > 9>10
*O.s.senegalensis*	SMF 25448	Gambia	18.3	6.4	9.8	12.5	42.3	17.3	24.2	141	65	175	66	95	96	96	90	89	81	7 = 8>9 > 6>5 > 5>10
*O.s.senegalensis*	BMNH 1929.2.18.131	Gambia	NA	6.7	NA	NA	41.0	16.5	22.0	140	60	167	81	98	104	106	105	96	87	7 > 6>8 > 9>5 > 4>10
*O.s.senegalensis*	BMNH 1907.12.26.41	Gambia	17.0	8.0	10.2	9.4	42.0	19.0	23.0	140	60	190	73	92	99	103	100	94	87	7 > 6>8 > 5>9 > 4>10
*O.s.senegalensis*	BMNH 1955.6.N-20.3927	Cape Verde	17.0	6.0	11.0	9.0	39.0	18.0	22.0	135	60	170	NA	NA	NA	NA	NA	NA	NA	NA
*O.s.senegalensis*	BMNH 1955.6.N-20.3926	Senegal	15.3	6.5	10.7	8.2	40.0	15.5	23.0	145	65	170	NA	NA	NA	NA	NA	NA	NA	8 > 7>9 > 6>5 > 4>10
*O.s.senegalensis*	BMNH 1955.6.N-20.3930	West Africa	16.5	6.1	11.7	9.9	40.0	15.5	24.0	135	57	170	80	97	100	98	98	93	87	8 > 7=6 > 9>5 > 4>10
*O.s.senegalensis*	BMNH 94.8.15.28	Ghana, Accra	17.1	6.1	11.0	10.5	42.0	15.5	23.0	137	58	175	70	87	94	95	89	84	82	7 > 8>6 > 9>5 > 4>10
*O.s.senegalensis*	BMNH 1930.12.21.13	Ghana, Tamatuku	15.8	6.3	11.0	9.0	40.0	17.5	20.0	125	55	173	NA	NA	NA	NA	NA	NA	NA	NA
*O.s.senegalensis*	BMNH 1911.12.23.506	Nigeria, Bauchi	17.2	5.2	11.0	9.4	44.0	18.5	22.0	135	58	170	89	94	99	98	94	88	85	8 > 7>6 = 9>10 > 5>4
*O.s.senegalensis*	BMNH 1909.12.31.43	DRC, Bunkeya	16.4	6.3	11.1	9.2	40.5	21.0	23.0	140	60	165	76	92	103	105	108	98	93	6 > 7>8 > 5>4 > 9>10
*O.s.senegalensis*	BMNH 1909.12.31.42	DRC, Katanga	15.8	5.0	10.4	8.0	41.5	15.0	23.0	130	57	150	70	90	95	97	93	86	80	7 > 8>6 > 9>5 > 4>10
*O.s.senegalensis*	BMNH 1957.35.44	Angola, Sumbe	15.5	5.0	10.4	8.5	37.0	15.5	22.0	125	60	165	65	83	90	90	85	80	75	7 = 8>6 > 9>5 > 4>10
*O.s.senegalensis*	BMNH 1937.12.27.211	Tanzania	16.7	5.8	9.7	9.5	40.0	16.0	22.0	128	50	160	70	82	90	91	90	86	82	7 > 6=8 > 5>4 = 9>10
*O.s.senegalensis*	BMNH 1936.2.21.479	Djibouti	16.5	6.0	11.0	9.8	42.0	17.0	21.0	137	60	170	72	87	97	99	96	83	83	7 > 8>6 > 9>4 = 5>10
*O.s.senegalensis*	SMF 10119	Ethiopia, Maki	16.8	6.3	9.3	11.0	36.1	17.2	22.1	128	53	151	72	87	93	96	96	91	NA	NA
*O.s.senegalensis*	SMF 10121	Somalia, Bardera	16.5	4.8	8.0	10.1	32.7	13.8	21.5	103	40	126	54	65	72	72	74	72	64	6 > 5=7 = 8>9 > 4>10
*O.senegalensisfeae*	BMNH 1911.12.23.4044	EG, Annobón	16.8	6.4	12.0	10.7	40.0	17.5	20.0	135	60	185	84	95	104	106	102	NA	NA	7 > 8>6 > 9>10 > 5>4
*O.senegalensisfeae*	SMF 25452	EG, Annobón	16.9	6.8	9.2	10.9	36.6	16.9	24.1	130	55	165	74	89	97	96	94	91	85	8 > 7>6 > 5>9 > 4>10
*O.pembaensis*	BMNH 1937.2.14.1 ^HT^	Tanzania, Pemba	19.0	8.9	NA	12.2	49.0	20.0	28.0	152	73	210	75	97	108	111	104	98	90	7 > 8>6 > 5>9 > 4>10
*O.pembaensis*	BMNH 1937.12.14.2	Tanzania, Pemba	20.5	8.5	NA	12.7	47.0	21.0	30.0	155	75	212	76	100	109	111	102	97	90	7 > 8>6 > 9>5 > 4>10
*O.pembaensis*	BMNH 1937.12.14.3	Tanzania, Pemba	19.0	9.0	NA	12.2	48.0	20.0	28.0	155	78	212	75	98	109	110	107	95	91	7 > 8>6 > 5>9 > 4>10
*O.pembaensis*	BMNH 1937.12.14.4	Tanzania, Pemba	20.0	8.5	13.5	12.1	47.0	21.0	28.0	150	80	212	75	99	111	112	108	101	96	7 > 8>6 > 5>9 > 4>10
*O.pembaensis*	BMNH 1937.12.14.5	Tanzania, Pemba	18.2	7.5	NA	11.2	46.5	20.0	29.0	150	76	210	75	96	103	104	103	97	92	7 > 6=8 > 5>9 > 4>10
*O.pembaensis*	BMNH 1937.12.14.6	Tanzania, Pemba	18.9	8.0	11.5	11.9	47.0	20.0	28.0	150	76	210	70	95	107	114	113	109	103	7 > 6>5 > 8>4 > 9>10
*O.pembaensis*	BMNH 1956.29.9	Tanzania, Pemba	NA	NA	NA	NA	NA	NA	NA	152	NA	190	77	98	107	110	107	104	94	7 > 8=6 > 5>9 > 4>10
*O.scops*	BMNH 941.5.30.8805	Spain, Ibiza	16.2	5.7	9.2	9.5	39.0	18.0	22.0	154	65	190	98	113	116	112	106	101	96	8 > 9>7 > 6>5 > 10 > 4
*O.scops*	BMNH 87.11.11.43	Spain, Seville	15.1	5.8	11.0	9.1	37.0	16.5	23.0	152	70	182	94	109	107	105	98	92	90	9 > 8>7 > 6>10 > 5>4
*O.scops*	BMNH 97.11.10.292	Spain, Malaga	16.8	6.2	11.0	9.8	40.5	14.2	28.0	147	68	170	85	102	109	106	98	87	86	8 > 7>9 > 6>5 > 4>10
*O.scops*	BMNH 1947.4.89	France, Var	16.1	6.8	11.0	9.0	43.0	18.0	28.0	160	70	200	96	114	114	114	101	96	85	7 = 8=9 > 6>10 = 5>4
*O.scops*	BMNH 1934.1.1.1510	Italy, Bibbiena	16.5	7.0	10.5	9.5	43.0	17.0	24.0	157	71	186	100	117	118	115	111	98	96	8 > 9>7 > 6>10 > 5>4
*O.scops*	BMNH 1905.6.28.739	Italy, Naples	16.5	6.8	10.5	10.0	43.5	17.0	26.0	155	68	185	95	106	110	105	102	95	93	8 > 9>7 > 6>10 = 5>4
*O.scops*	BMNH 1905.6.28.740	Italy, Naples	16.9	6.6	11.0	9.7	46.0	16.1	27.0	159	70	200	92	110	113	109	100	92	90	8 > 9>7 > 6>10 = 5>4
*O.scops*	BMNH 1955.6.N-20.3874	Morocco, Tangier	16.7	7.8	NA	11.5	43.0	20.0	22.0	155	65	180	92	104	104	102	99	94	94	8 = 9>7 > 6>5 = 4>10
*O.scops*	BMNH 1919.12.11.8	Morocco, Atlas	15.0	6.5	9.8	10.0	39.5	14.0	22.0	150	80	178	94	107	109	107	92	88	88	8 > 9=7 > 10 > 6>5 = 4
*O.scops*	BMNH 73.5.28.10	Algeria	18.1	6.5	NA	11.0	42.0	17.0	26.0	154	70	180	91	105	106	106	98	94	87	8 = 7>9 > 6>5 > 10 > 4
*O.scops*	BMNH 1916.9.20.746	Sudan, Trufikia	15.5	6.7	NA	9.5	45.0	18.5	24.0	163	75	195	100	115	116	111	100	98	98	8 > 9>7 > 10 = 6>5 = 4
*O.scops*	BMNH 1977.20.227	Liberia, Mt. Nimba	15.0	6.5	11.5	10.0	42.0	17.0	23.0	154	70	185	NA	NA	NA	NA	NA	NA	NA	7 > 6>8 > 5>4 > 9>10
*O.scops*	BMNH 1977.20.229	Liberia, Mt. Nimba	14.0	5.5	10.3	10.3	42.0	19.0	26.5	155	70	195	94	112	112	107	98	96	91	8 = 9>7 > 6>5 > 10 > 4
*O.scops*	BMNH 1977.20.232	Liberia, Mt. Nimba	17.0	5.5	10.4	10.0	40.2	17.0	27.0	157	72	175	92	104	NA	NA	NA	NA	NA	NA
*O.scops*	BMNH 1977.20.228	Liberia, Mt. Nimba	17.3	6.0	10.2	9.5	40.0	17.0	23.0	165	73	190	105	115	NA	NA	NA	NA	NA	8 > 9>7 > 6>10 > 5>4
*O.bruceiobsoletus*	SMF 25430	Uzbekistan	18.5	5.8	8.7	11.5	43.1	16.9	29.6	153	72	186	96	112	112	114	109	100	90	7 > 8=9 > 6>5>10>4

Abbreviations of institutional collections: BMNH - The Natural History Museum, Tring, UK; MHNC-UP - Museu de História Natural e da Ciência da Universidade do Porto, Portugal; SMD - Senckenberg Museum Dresden, Germany; SMF - Naturmuseum Senckenberg in Frankfurt am Main, Germany

### ﻿Taxonomy

In this study, species diagnosis was based upon four lines of evidence: morphometrics, plumage colouration and pattern, song, and DNA sequence data.

Species and subspecies limits of scops-owls are challenging to ascertain, leading to numerous taxonomic arrangements. This study follows the taxonomic arrangement and nomenclature of The Clements Checklist of Birds of the World (Clements et al. 2021). Terminology and description scheme follow Sangster et al. (2013), and the features used for the description are depicted in the Suppl. material 3. Description of colour in life is based on the holotype, with some reference to variation as observed in specimens photographed in the field (Fig. 2).

### ﻿Morphology

Four scops-owl individuals from Príncipe (including the vouchered specimen) were captured in the field. These were measured together with representatives of four of the five species of the Afro-Palearctic clade (sensu Pons et al. 2013) and *O.bruceiobsoletus* (Cabanis, 1875). The latter was included due to the potential affinities of *O.brucei* (Hume, 1872) with African species (Pons et al. 2013). The Arabian Scops-Owl *O.pamelae* Bates, 1937 was not included, but we measured individuals of *O.senegalensisfeae* (Salvadori, 1903) from Annobón Island, considered by some authors as a distinct species (Collar and Boesman 2020; Gill et al. 2021). The morphometric dataset includes measurements collected from museum specimens and living individuals measured in the field (Table 1).

Measurements were taken as follows: bill length from the bill tip to where the culmen enter the feathers (**Bilen**); bill length from the anterior end of the nares to the tip (**Binares**); bill width (**Biwid**) and bill depth (**Bidepth**) at the anterior end of nares; head+bill (**Hebi**), from the tip of the bill to the opposite point on the back of the skull; middle toe length (**Midt**); tarsus length (**Tarlen**), from the tibiotarsus joint to the distal end of the tarsometatarsus, when the foot is held to the leg; tail length (**Tailen**), from where the ruler stops at the root of the central pair of rectrices and to the tip of this same pair (by sliding the ruler between the rectrices and the undertail coverts); body length (**Bolen**) from the top of the head to the tip of the central pair of rectrices; wing length (**Wilen**), flattened, from the carpal joint to the tip of the longest primary; wing formula, sequence of primary feathers ordered by size; and length of primary feathers (**P4–P10**, in which **P1** is the closest to the body), which were transformed in shortfall of P4–P10 to tip of longest primary. Body, wing, and tail length were measured with standard wing and tail rulers to the nearest 1.0 mm. The length of the primary feathers was measured to the nearest 1.0 mm with a ruler with a pin at the origin; the pin is inserted between two primary feathers until it touches the skin (Jenni and Winkler 1989). All other measurements were made using a digital calliper (Mitutoyo CD-P15K, Mitutoyo Corporation, Kawasaki, Japan) to the nearest 0.1 mm. All measurements were collected by MM, BF, and RL (Table 1). A constant of one was added to each number to make all shortfalls non-zero. All mea­surements were log-transformed (base-10) to normalise distributions (McDonald 2014).

The four individuals from Príncipe were sexed with a molecular protocol (Griffiths et al. 1998), and comprised three females and one male (Table 1). For the statistical analyses males and females were treated together due to the low sampling size, and the fact that most museum specimens were not sexed.

Morphometric differences were explored using a Principal Component Analysis (PCA), performed using the FactoMineR package (Lê et al. 2008) and carried out using R v. 3.6 (R Core Team 2017) in RStudio v. 1.1.447 (RStudio Team 2015). Measurements were size-standardised to prevent the dominance of variables invol­ving larger measurement units, thus allowing comparisons between variables. The wing formula was not used in the analyses. Since several individuals had missing data, to maxi­mise the number of analysed specimens of each species only the following variables were used in the PCA: Bilen, Binares, Biwid, Tarlen, Wilen, and Tailen. This dataset included 44 specimens from seven taxa, including three individuals from Príncipe. Welch’s ANOVA (recommended for unbalanced designs, different samples sizes, and different standard deviations; McDonald 2014), was used together with Games-Howell post-hoc comparisons to test whether the groups differed from each other.

### ﻿Plumage description

We used colour standards (Köhler 2012) to describe the plumage of the species of the Afro-Palearctic clade, except for *O.pamelae*, but including *O.senegalensisfeae* (see Suppl. material 6). The topographic terms of the scops-owl body are detailed in the Suppl. material 3.

### ﻿Bioacoustic analyses

We compared the calls of the candidate species with the calls of scops-owls from the Afro-Palearctic clade, *O.brucei*, and the Sokoke Scops-Owl *O.ireneae* Ripley, 1966 (Tables 2, 3). *Otusireneae* is not part of the Afro-Palearctic clade but was included because it has the most similar calls to the ones of the candidate species. Recordings were collected from Xeno-canto (XC; www.xeno-canto.org), Avian Vocalizations Center (AVoCet; https://avocet.integrativebiology.natsci.msu.edu), The Internet Bird Collection (www.hbw.com/ibc), the private collection of PV and the collection of vocalisations obtained during fieldwork on Príncipe by co-authors. Newly collected calls were deposited in Xeno-canto (Suppl. material 7). From each independent source, only one recording was used (unless there were only very limited recordings available). In total, 43 recordings from ten taxa were analysed (Suppl. material 7). In most owls, both sexes produce similar calls for territorial defence, mate attraction, and pair-bonding (Marks et al. 1999; König et al. 2008), therefore our analyses included both male and female recordings. Vocalisations of scops-owls are generally made up of a simple primary call composed by the repetition of the same note (Marks et al. 1999). The call of the Cyprus Scops-Owl *O.cyprius* (von Madarász, 1901) is composed by the coupling of one long and one short note (Flint et al. 2015), and both note types were included in the analysis. The candidate species primary call is characterised by a repeated note (Suppl. material 4: Fig. S2A, B), but its repertoire also includes a cat-like “kee-a-u” note (Suppl. material 4: Fig. S2C, D). The latter was not included in the analysis.

**Table 2.** Measurements (in Hz) of bioacoustic variables (frequency parameters) of *Otus* species of the Afro-Palearctic clade, *O.brucei* and of *O.ireneae* (the species whose vocalisations are closest to the ones of the candidate species from Príncipe). *n*: number of individuals. Average ± standard deviation; (minimum-maximum values). F1: frequency at start; F2: frequency at end; F3: frequency at 25% of total duration; F4: frequency at midpoint; F5: frequency at 75% of total duration; F6: frequency at maximum amplitude; F7: maximum frequency; F8: minimum frequency.

Taxon	*n*	F1	F2	F3	F4	F5	F6	F7	F8
*O.bikegila* sp. nov. (main call)	5	891.0 ± 72.9	967.0 ± 32.3	1012.3 ± 44.2	981.3 ± 40.2	967.7 ± 35.8	1005.7 ± 47.6	1054.0 ± 30.1	910.3 ± 43.2
		(781.7–961.7)	(933.3–1020.0)	(980.0–1090.0)	(950.0–1050.0)	(931.7–1020.0)	(976.7–1090.0)	(1035.0–1106.7)	(868.3–973.3)
*O.bikegila* sp. nov. (cat-like call)	2	966.7 ± 80.1	859.2 ± 27.1	1180.8 ± 121.4	1234.2 ± 62.5	1149.2 ± 140.2	1220.0 ± 70.7	1245.0 ± 77.8	853.3 ± 4.7
		(910.0–1023.3)	(840.0–878.3)	(1095.0–1266.7)	(1190.0–1278.3)	(1050.0–1248.3)	(1170.0–1270.0)	(1190.0–1300.0)	(850.0–856.7)
*O.hartlaubi*	5	1236.2 ± 101.5	1220.7 ± 54.9	1409.7 ± 65.9	1396.3 ± 80.0	1340.5 ± 62.2	1383.2 ± 62.5	1461.8 ± 47.2	1178.2 ± 83.0
		(1078.3–1360.0)	(1155.0–1285.0)	(1330.0–1478.3)	(1295.0–1483.3)	(1250.0–1418.3)	(1331.7–1480.0)	(1407.5–1526.7)	(1066.7–1267.5)
*O.senegalensissenegalensis*	4	1235.0 ± 184.2	1133.3 ± 112.7	1092.5 ± 123.3	1156.7 ± 60.5	1074.6 ± 83.9	1134.2 ± 84.3	1422.5 ± 341.5	963.3 ± 70.4
		(1071.7–1433.3)	(1035.0–1260.0)	(1021.7–1276.7)	(1095.0–1236.7)	(1011.7–1198.3)	(1051.7–1251.7)	(1126.7–1908.3)	(885.0–1050.0)
*O.senegalensisfeae*	3	1220.3 ± 146.6	1189.2 ± 50.6	1165.3 ± 76.3	1203.3 ± 92.5	1143.1 ± 46.8	1156.9 ± 75.1	1310.3 ± 54.9	1098.9 ± 68.1
		(1060.0–1347.5)	(1132.5–1230.0)	(1120.0–1253.3)	(1145.0–1310.0)	(1110.0–1196.7)	(1107.5–1243.3)	(1250.0–1357.5)	(1050.0–1176.7)
*O.pembaensis*	5	575.3 ± 47.8	665.7 ± 65.3	693.7 ± 52.8	709.7 ± 52.7	711.0 ± 49.1	702.0 ± 50.0	726.0 ± 48.9	593.0 ± 49.5
		(506.7–636.7)	(613.3–773.3)	(621.7–770.0)	(633.3–780.0)	(651.7–783.3)	(638.3–776.7)	(660.0–790.0)	(530.0–660.0)
*O.pamelae*	5	1116.3 ± 72.7	1190.0 ± 55.4	1219.0 ± 90.5	1241.0 ± 104.8	1179.7 ± 75.6	1250.3 ± 91.5	1349.0 ± 114.4	1054.3 ± 40.4
		(1031.7–1213.3)	(1111.7–1268.3)	(1120.0–1363.3)	(1096.7–1366.7)	(1083.3–1291.7)	(1160.0–1403.3)	(1220.0–1516.7)	(1020.0–1123.3)
*O.scops*	5	1505 ± 137.5	1210.2 ± 44.1	1203.8 ± 39.0	1230.1 ± 34.9	1255.3 ± 44.7	1255.3 ± 46.2	1478.6 ± 121.8	1183.5 ± 43.4
		(1335.0–1695.0)	(1152.5–1275.0)	(1155.0–1260.0)	(1200.0–1285.0)	(1193.3–1318.3)	(1210.0–1326.7)	(1336.3–1628.3)	(1140.0–1253.3)
*O.cyprius* (long note)	5	1312.6 ± 60.0	1079.7 ± 67.1	1084.6 ± 73.1	1091.0 ± 71.7	1101.4 ± 65.1	1139.4 ± 72.7	1391.1 ± 85.4	1059.7 ± 72.2
		(1236.7–1400.0)	(1016.7–1193.3)	(1035.0–1213.3)	(1035.0–1216.7)	(1043.4–1213.3)	(1063.4–1231.7)	(1291.7–1520.0)	(996.7–1183.3)
*O.cyprius* (short note)	5	1128.1 ± 59.7	1038.5 ± 57.1	1094.8 ± 132.1	1046.6 ± 90.6	1045.7 ± 85.5	1067.2 ± 72.9	1212.9 ± 104.9	1007.9 ± 75.4
		(1058.9–1223.3)	(998.3–1135.0)	(1015.0–1330.0)	(976.7–1200.0)	(960.0–1186.7)	(1020.0–1196.7)	(1124.4–1345.0)	(945.0–1138.3)
*O.brucei*	4	355.0 ± 48.8	358.3 ± 49.1	463.8 ± 75.5	447.1 ± 62.0	390.4 ± 50.2	462.1 ± 63.6	469.6 ± 69.5	336.3 ± 48.0
		(285.0–388.3)	(285.0–388.3)	(356.7–530.0)	(356.7–493.3)	(335.0–450.0)	(370.0–510.0)	(370.0–530.0)	(270.0–373.3)
*O.ireneae*	2	936.7 ± 99.0	919.2 ± 38.9	943.3 ± 75.4	945.0 ± 73.1	926.7 ± 51.9	946.7 ± 61.3	958.3 ± 58.9	892.5 ± 43.6
		(866.7–1006.7)	(891.7–946.7)	(890.0–996.7)	(893.3–996.7)	(890.0–963.3)	(903.3–990.0)	(916.7–1000.0)	(861.7–923.3)

**Table 3.** Measurements (in Hz) of bioacoustic variables (temporal parameters) of *Otus* species of the Afro-Palearctic clade, *O.brucei* and of *O.ireneae* (the species whose vocalisations are closest to the ones of the candidate species from Príncipe). *n*: number of individuals. Average ± standard deviation; (minimum-maximum values). DT1: total duration; DT2: time to maximum amplitude; DT3: time to maximum frequency; DT4: internote interval; DF1: frequency drop from start to end; DF2: frequency range; DFT1: slope from 25% to 75% of total duration; DFT2: slope from midpoint to end.

Taxon	*n*	DT1	DT2	DT3	DT4	DF1	DF2	DFT1	DFT2
*O.bikegila* sp. nov.	5	0.238 ± 0.007	0.100 ± 0.014	0.057 ± 0.021	1.046 ± 0.053	76.0 ± 62.8	143.7 ± 25.8	-374.4 ± 252.1	-120.9 ± 110.2
		(0.231–0.248)	(0.078–0.112)	(0.032–0.083)	(0.992–1.121)	(6.7–178.3)	(110–175)	(-581.5–45.2)	(-250.3–21.7)
*O.bikegila* sp. nov. (cat-like call)	2	0.347 ± 0.009	0.161 ± 0.002	0.138 ± 0.025	-	-107.5 ± 53.0	391.7 ± 73.1	-185.7 ± 117.8	-2158.6 ± 146.7
		(0.341–0.354)	(0.160–0.162)	(0.120–0.155)	-	(-145.0 – -70.0)	(340.0–443.3)	(-268.9 – -102.4)	(-2262.4 – -2054.9)
*O.hartlaubi*	5	0.292 ± 0.017	0.161 ± 0.054	0.078 ± 0.038	13.899 ± 2.847	-15.5 ± 126.5	283.7 ± 106.5	-468.4 ± 348.7	-1209.4 ± 672.6
		(0.267–0.315)	(0.113–0.241)	(0.030–0.110)	(9.181–15.998)	(-141.7–186.7)	(140.0–403.3)	(-773.0 – -61.0)	(-2034.1 – -312.7)
*O.senegalensissenegalensis*	4	0.337 ± 0.150	0.201 ± 0.135	0.058 ± 0.047	6.446 ± 2.426	-101.7 ± 72.1	459.2 ± 381.1	-155.8 ± 429.3	-240.3 ± 500.7
		(0.206–0.476)	(0.091–0.370)	(0.010–0.102)	(4.094–9.131)	(-173.3 – -30.0)	(191.7–1023.3)	(-769.7–230.9)	(-570.1–503.1)
*O.senegalensisfeae*	3	0.420 ± 0.019	0.216 ± 0.087	0.119 ± 0.153	7.247 ± 0.201	-31.1 ± 107.7	211.4 ± 71.1	-92.5 ± 143.8	-61.8 ± 301.4
		(0.402–0.441)	(0.121–0.291)	(0.013–0.295)	(7.127–7.479)	(-142.5–72.5)	(146.7–287.5)	(-253.2–23.8)	(-364.3–238.5)
*O.pembaensis*	5	0.225 ± 0.017	0.112 ± 0.045	0.112 ± 0.041	6.121 ± 0.916	90.3 ± 38.8	133.0 ± 7.1	156.4 ± 121.3	-409.1 ± 333.4
		(0.207–0.246)	(0.061–0.171)	(0.043–0.155)	(5.043–7.570)	(48.3–136.7)	(126.7–145.0)	(-37.3–246.9)	(-784.9 – -58.1)
*O.pamelae*	5	0.390 ± 0.078	0.160 ± 0.035	0.103 ± 0.034	5.665 ± 4.550	73.7 ± 59.3	294.7 ± 80.7	-202.1 ± 130.9	-278.6 ± 443.4
		(0.281–0.481)	(0.120–0.206)	(0.068–0.157)	(0.444–11.381)	(21.7–158.3)	(200.0–393.3)	(-407.3 – -65.4)	(-588.9–383.5)
*O.scops*	5	0.248 ± 0.033	0.102 ± 0.050	0.020 ± 0.017	2.608 ± 0.174	-294.8 ± 95.4	295.1 ± 97.6	430.5 ± 193.2	-187.5 ± 314.0
		(0.206–0.291)	(0.047–0.169)	(0.010–0.050)	(2.423–2.789)	(-420.0 – -182.5)	(178.8–401.7)	(181.5–662.3)	(-730.4–66.1)
*O.cyprius* (long note)	5	0.226 ± 0.014	0.088 ± 0.039	0.019 ± 0.006	3.245 ± 0.236	-232.8 ± 49.4	331.4 ± 56.8	141.9 ± 160.6	-101.0 ± 114.6
		(0.213–0.245)	(0.049–0.145)	(0.013–0.028)	(3.035–3.643)	(-283.3 – -165.0)	(241.7–395.0)	(-32.1–363.9)	(-211.4–34.0)
*O.cyprius* (short note)	5	0.124 ± 0.035	0.059 ± 0.024	0.014 ± 0.006	3.344 ± 0.227	-89.6 ± 22.9	204.9 ± 72.7	-938.8 ± 1357.8	-357.5 ± 953.6
		(0.084–0.159)	(0.042–0.100)	(0.008–0.020)	(3.116–3.714)	(-113.3 – -58.1)	(129.7–318.3)	(-3144.9 – -2.4)	(-1438.2–561.9)
*O.brucei*	4	0.115 ± 0.021	0.040 ± 0.010	0.032 ± 0.001	0.756 ± 0.140	3.3 ± 15.5	133.3 ± 44.5	-1166.5 ± 960.3	-1545.8 ± 115.2
		(0.090–0.133)	(0.032–0.053)	(0.032–0.033)	(0.629–0.922)	(-11.7–25.0)	(100.0–198.3)	(-2457.3 – -377.4)	(-1653.0 – -1387.3)
*O.ireneae*	2	0.173 ± 0.026	0.091 ± 0.008	0.063 ± 0.055	0.428 ± 0.027	-17.5 ± 60.1	65.8 ± 15.3	-173.7 ± 245.7	-273.8 ± 353.7
		(0.154–0.192)	(0.085–0.097)	(0.023–0.102)	(0.409–0.447)	(-60.0–25.0)	(55.0–76.7)	(-347.5–0.0)	(-523.9 – -23.7)

Recordings were sampled using a 16-bit accuracy and a sampling rate converted to 12 kHz in Avisoft-SASLab pro v. 4.3 (Avisoft Bioacoustics). The following 16 variables were collected for each note: F1, frequency at start (peak frequency at 0s, Hz); F2, frequency at end (peak frequency at last of four call intervals, Hz); F3, frequency at 25% of total duration (peak frequency at the first interval, Hz); F4, frequency at midpoint (peak frequency at the second interval, Hz); F5, frequency at 75% of total duration (peak frequency at the third interval, Hz); F6, frequency at maximum amplitude (frequency at maximum amplitude of note, Hz); F7, maximum frequency (maximum frequency through the note, Hz); F8, minimum frequency (minimum frequency through the note, Hz); DT1, total duration (duration, s); DT2, time to maximum amplitude (time to maximum amplitude of note, s); DT3, time to maximum frequency (time to maximum frequency of note, s); DT4, internote interval (start time – end time of previous note, s); DF1, frequency drop from start to end (F2-F1, Hz); DF2, frequency range (F7-F8, Hz); DFT1, slope from 25% to 75% of total duration ([F5-F3]/∆t, Hz/s); DFT2, slope from midpoint to end ([F2-F4]/∆t, Hz/s). The 16 variables were extracted from the analysis of the spectrograms. We used a Fast Fourier Transformation size of 512 points, a 100% frame size and a temporal resolution overlap of 87.5% (flat top window type), resulting in a frequency resolution of 86 Hz and a temporal resolution of 4.5 ms. Frequencies were analysed between 0.5 Hz (highpass) and 2.25 Hz (lowpass), except for *O.brucei* with the highpass set at 0.0 Hz; the greyscale was set to 30%. When background noise hampered the measurement of the variables, frequencies were filtered and adjusted by shortening the interval between the highpass and the lowpass.

For each recording, variables were measured on six notes and their means (Suppl. material 8) were used as sample points to calculate the ranges, means and standard deviations for each taxon. A constant of 3000 was added to each computed value to ensure that the dataset only included positive numbers. All measurements were log-transformed (base-10) to normalise distributions (McDonald 2014).

PCA was performed using the FactoMineR package (Lê et al. 2008) using R v. 3.6 (R Core Team 2017) in RStudio v. 1.1.447 (R Studio Team 2015). Measurements were size-standardised to make the variables comparable. Welch’s ANOVA and Games-Howell post-hoc comparisons were used to test whether the groups differed from each other (McDonald 2014).

### ﻿Molecular data

Blood samples were collected non-destructively from the brachial vein of mist-netted individuals and were stored in 96% ethanol for genetic analysis (see molecular dataset: Table 4). Blood samples of the scops-owl from Príncipe were collected at Boca do Inferno (1°36.16'N, 7°24.06'E, ca. 300 m a.s.l.) (*n* = 2) and close to Ribeira Porco (1°33.03'N, 7°22.29'E, ca. 100 m a.s.l.) (*n* = 2), both localities within Príncipe Obô Natural Park (Fig. 1; Suppl. material 9). We obtained four blood samples of *O.hartlaubi* (Giebel, 1872) from São Tomé (Suppl. material 9), and were lent one blood sample of *O.senegalensisfeae* (Annobón), and one blood sample of Moheli Scops-Owl *O.moheliensis* Lafontaine & Moulaert 1998. We were lent additional samples from museum specimens, including toe-pad samples for one of each of the two subspecies of the Sandy Scops-Owl, *O.icterorhynchusicterorhynchus* (Shelley, 1873) and *O.i.holerythrus* (Sharpe, 1901), from the American Museum of Natural History, New York (**AMNH**) (Table 4).

**Table 4.** List of scops-owls (*Otus*) samples and GenBank accession numbers for the gene fragments used in this study. Accession numbers in bold indicate sequences newly produced for this study. STP – São Tomé and Príncipe; EG – Equatorial Guinea; DRC – Democratic Republic of the Congo; CHIMERA – sequences for a given taxon obtained from different individuals.

Taxon	Locality	12S	16S	ATP6	COI	CYTB	ND2	ND3	KIAA	MYO2	RAG1	SACS	TGFB2	TTN
*O.bikegila* sp. nov. 1	STP: Príncipe	**OM978880**	**OM978895**	**OM913485**	**OM937282**	**OM937307**	**OM937351**	**ON016156**	**OM937319**	**OM937336**	**ON016107**	**ON016118**	**ON016136**	**ON016141**
*O.bikegila* sp. nov. 2	STP: Príncipe	**OM978881**	**OM978896**	**OM913486**	**OM937283**	**OM937308**	**OM937352**	**ON016157**	–	–	**ON016108**	**ON016119**	**ON016137**	**ON016142**
*O.bikegila* sp. nov. 3	STP: Príncipe	**OM978882**	**OM978897**	–	**OM937284**	**OM937309**	**OM937353**	**ON016158**	–	**OM937337**	**ON016109**	**ON016120**	**ON016139**	**ON016143**
*O.bikegila* sp. nov. 4	STP: Príncipe	**OM978883**	**OM978898**	**OM913487**	**OM937285**	**OM937310**	**OM937354**	**ON016159**	**OM937320**	**OM937338**	**ON016110**	**ON016121**	**ON016140**	**ON016144**
*O.hartlaubi* 1	STP: São Tomé	**OM978884**	**OM978899**	EU601139	**OM937286**	EU601108	EU601032	EU600995	**OM937321**	EU601072	–	**ON016122**	EU600952	**ON016145**
*O.hartlaubi* 2	STP: São Tomé	**OM978885**	**OM978900**	–	**OM937287**	**OM937303**	**OM937349**	**ON016160**	**OM937322**	**OM937329**	**ON016111**	**ON016123**	**ON016130**	**ON016146**
*O.hartlaubi* 3	STP: São Tomé	**OM978886**	**OM978901**	–	**OM937288**	**OM937304**	**OM937347**	**ON016161**	**OM937323**	**OM937330**	**ON016112**	**ON016124**	**ON016131**	**ON016147**
*O.hartlaubi* 4	STP: São Tomé	**OM978887**	**OM978902**	**OM913483**	**OM937289**	**OM937305**	**OM937348**	**ON016162**	**OM937324**	**OM937331**	**ON016113**	**ON016125**	**ON016132**	**ON016148**
*O.senegalensissenegalensis*	South Africa	–	–	EU601166	–	EU601127	EU601056	EU601019	–	EU601098	–	–	EU600976	–
*O.senegalensisfeae*	EG: Annobón	**OM978891**	**OM978908**	**OM913484**	**OM937293**	**OM937306**	**OM937350**	**ON016155**	**OM937327**	**OM937333**	**ON016116**	**ON016128**	**ON016138**	**ON016151**
*O.pembaensis* 1	Tanzania: Pemba	–	–	EU601157	–	EU601123	EU601048	EU601010	–	EU601090	–	–	EU600967	–
*O.pembaensis* 2	Tanzania: Pemba	–	–	EU601158	–	EU601124	EU601049	EU601011	–	EU601091	–	–	EU600968	–
*O.pamelae*	Saudi Arabia	–	–	–	–	–	KC138819	KC138827	–	KC138812	–	–	–	–
*O.scops* 1	France	–	–	EU601146	–	EU601115	EU601039	EU601001	–	EU601079	–	–	EU600958	–
*O.scops* 2	France	–	**OM978906**	–	–	**OM937314**	–	**ON016164**	**OM937325**	**OM937334**	**ON016114**	**ON016126**	**ON016135**	**ON016149**
*O.scops* 3	France	–	**OM978907**	–	–	**OM937313**	–	**ON016165**	**OM937326**	**OM937339**	**ON016115**	**ON016127**	**ON016133**	**ON016150**
*O.scops* 4	France	**OM978890**	**OM978905**	**OM913488**	**OM937292**	**OM937312**	**OM937355**	**ON016163**	–	–	–	–	–	–
*O.cyprius*	Cyprus	–	–	–	KT803674	**OM937311**	–	–	–	–	–	–	–	–
*O.brucei*	CHIMERA– United Arab Emirates; Oman	–	–	–	–	EU348985	KC138817	KC138825	–	KC138811	EU348920	–	–	–
*O.longicornis*	CHIMERA– Philippines – Isabela, Luzon; Unknown	U83751	**OM978909**	EU601151	**OM937294**	EU601119	**OM937356**	EU601005	–	EU601084	–	–	EU600962	–
*O.mirus*	CHIMERA– Philippines: Mindanao; Unknown	U83752	–	–	–	EU601126	EU601057	EU601020	–	EU601099	–	–	EU600978	–
*O.elegans*	Unknown	–	–	–	AB842985	EU123899	–	–	–	–	–	–	–	–
*O.mayottensis*	Mayotte	–	–	EU601154	–	EU601122	EU601046	EU601008	–	EU601087	–	–	EU600965	–
*O.madagascariensis*	Madagascar	**OM978893**	**OM978911**	**OM913489**	**OM937295**	**OM937315**	**OM937357**	**ON016166**	–	EU601082	–	–	EU600960	–
*O.rutilus*	Madagascar	–	–	EU601135	–	EF198270	EF198304	EU600989	–	EU601066	–	–	EU600946	–
*O.capnodes*	Comoros: Anjouan	–	–	EU601145	–	EU601114	EU601038	EU601000	–	EU601078	–	–	EU600957	–
*O.insularis*	Seychelles: Mahe	–	–	EU601128	–	EU601101	EU601022	EU600983	–	EU601059	–	–	EU600940	–
*O.sunia*	CHIMERA– China x 2; Thailand	**OM978894**	**OM978912**	**OM913491**	**ON016106**	**OM937316**	**OM937358**	**ON016167**	–	EU601081	EU348927	–	EU600959	–
*O.socotranus*	Socotra	–	–	–	–	–	KC138824	KC138832	–	KC138816	–	–	KC138810	–
*O.pauliani*	Comoros: Grande Comore	–	–	–	–	EU601125	EU601058	EU601021	–	EU601100	–	–	EU600979	–
*O.moheliensis*	Comoros: Moheli	**OM978892**	**OM978910**	**OM913490**	–	**OM937317**	**EU601045**	**ON016168**	**OM937328**	**OM937335**	**ON016117**	**ON016129**	**ON016134**	**ON016152**
*O.icterorhynchusholerythrus*	CHIMERA– Cameroon: Efulan; DRC: Kivu	–	–	–	–	**OM937318**	**OM937359**	**ON016169**	–	–	–	–	–	–
*O.icterorhynchusicterorhynchus*	Liberia: Lofa County	–	–	–	–	–	**OM937360**	**ON016170**	–	**OM937332**	–	–	–	–
*O.bakkamoenamarathae*	India: Wadi	–	–	–	–	–	**OM937340**	–	–	–	–	–	–	–
*O.lempiji*	CHIMERA– Singapore: Indonesia (captive); Unknown	**OM978888**	**OM978903**	**OM913481**	**OM937290**	**OM937296**	**OM937341**	**ON016153**	–	EU601076	EU348922	–	EU600981	–
*O.lettia*	CHIMERA– Russia; China; Laos	–	–	EU601140	GQ482285	EU601109	EU601033	EU600996	–	EU601073	EU348923	–	EU600953	–
*O.megalotis*	CHIMERA– Philippines – Isabela: Luzon	–	–	EU601133	JQ175645	EU601105	EU601027	EU600988	–	EU601064	–	–	EU600944	–
*O.nigrorum*	CHIMERA– Philippines: Panay; Unknown	U83755	–	–	–	JN131497	KF792802	–	–	–	EU348924	–	–	–
*O.everetti*	CHIMERA– Philippines: Mindanao; Unknown	U83754	–	–	U83779	JN131492	JN131480	–	–	–	–	–	–	–
*O.semitorques*	CHIMERA– Russia; Unknown	AY513588	–	EU601142	AB843645	EU601111	EU601035	EU600998	–	EU601075	–	–	EU600955	–
*O.angelinae*	Indonesia: Java	–	–	–	–	–	**OM937342**	–	–	–	–	–	–	–
*O.silvicola*	Indonesia: Flores	–	–	–	–	**OM937302**	–	–	–	–	–	–	–	–
*O.spilocephalusvandewateri*	Sumatra	–	–	–	–	**OM937297**	**OM937343**	–	–	–	–	–	–	–
*O.spilocephalusvulpes*	Malaysia: Perak	–	–	–	–	**OM937298**	**OM937344**	–	–	–	–	–	–	–
*O.spilocephalusluciae*	Borneo	–	–	–	–	**OM937299**	–	–	–	–	–	–	–	–
*O.spilocephalusspilocephalus*	CHIMERA– China; Unknown	–	–	EU601147	–	EU601116	EU601040	–	–	EU601080	KJ456094	–	EU600980	–
*O.spilocephalushambroecki*	Taiwan: Horisha	–	–	–	–	**OM937300**	**OM937345**	–	–	–	–	–	–	–
*O.spilocephaluslatouchi*	CHIMERA– Laos; China	**OM978889**	**OM978904**	**OM913482**	**OM937291**	**OM937301**	**OM937346**	**ON016154**	–	–	EU348926	–	–	–
*O.ireneae*	Kenya	–	–	EU601144	–	EU601113	EU601037	EU600999	–	EU601077	–	–	EU600956	–
*Bubobubo*	France	–	–	EU601137	–	AJ003969	EU601029	EU600992	–	EU601069	–	–	EU600949	–
*Strixaluco*	France	–	–	EU601138	–	EU601107	EU601030	EU600993	–	EU601070	–	–	EU600950	–

Total genomic DNA was extracted from blood and tissue samples using an overnight Proteinase K digestion (10 mg/ml concentration) followed by a standard high-salt extraction method (Bruford et al. 1992). Before the extraction, blood was removed from the ethanol and left to dry in the incubator. Genomic extractions of toe-pads samples were performed using a specific protocol for museum samples (Dabney et al. 2013).

Mitochondrial and nuclear markers (mtDNA and nuDNA, respectively) were amplified and sequenced for the samples that were available to us. For mtDNA we amplified a fragment of the 12s and 16s ribosomal RNA genes (12S and 16S), ATPase subunit 6 (ATP6), cytochrome oxidase subunit I (COI), cytochrome *b* (CYTB), nicotinamide adenine dinucleotide dehydrogenase subunits 2 and 3 (ND2 and ND3). The nuDNA markers were: leucine-rich repeat and WD repeat-containing protein (KIAA1239), myoglobin intron-2 (MYO2), Recombination Activating Gene 1 (RAG1), sacsin (SACS), TGFb2 intron-5 (TGFB2), and titin (TTN).

Standard polymerase chain reactions (PCR) were performed in a final volume of 25 µl using 1 µl of each primer (10 pmol), 0.4 µl of total dNTPs (10 mM; Promega), 0.1 µl of 5 U/ml GoTaq Flexi DNA Polymerase (Promega), 5 µl of 5X Green GoTaq Flexi Buffer (Promega), 4 µl of MgCl_2_ (25 mM; Promega). The first PCR of the fragments amplified using a nested PCR approach (KIAA1239, SACS, TTN) were performed in half total reaction volume (12,5 µl). Primers and PCR conditions are provided as Suppl. material 10. For the amplification of the toe pads, we amplified shorter fragments of the MYO2, ND2, and ND3 genes. Successfully amplified products were purified and sequenced using dye-labelled dideoxy terminator cycle sequencing on an ABI 3730XL automated sequencer at Macrogen Inc.

PCR amplification of CYTB and ND2 sequences of *O.cyprius*, *O.i.icterorhynchus*, Wallace’s Scops-Owl *O.silvicola* (Wallace, 1864), and four subspecies of the Mountain Scops-Owl (Blyth, 1846) [*O.spilocephalusvandewateri* (Robinson & Kloss, 1916), *O.s.vulpes* (Ogilvie-Grant, 1906), *O.s.luciae* (Sharpe, 1888), and *O.s.hambroecki* (Swinhoe, 1870)] was performed in seven fragments of ca. 180–200 bp using custom-made primers (see Suppl. material 10). PCR products were cycle-sequenced in both directions using the Big Dye Terminator v. 3.1. Sequences were read on an ABI 3100 capillary sequencer (Applied Biosystems, Foster City, CA, USA). Samples amplified and sequenced at Muséum national d'Histoire naturelle, Paris (**MNHN**) (Table 4) followed Pons et al. (2013).

Chromatograms of newly generated sequences were checked by eye, edited and aligned using BioEdit v. 7.0.5.3 (Hall 1999). All newly determined sequences were submitted to GenBank (accession numbers provided in Table 4).

### ﻿Molecular analyses

Six different datasets were compiled for different purposes, detailed below.

#### Dataset 1

ND2 sequences of the species belonging to the Afro-Palearctic clade and of *O.brucei*, whose taxonomic affinities are not resolved, although it appears to be closer to African and Indian Ocean islands species than to the Asian species (Pons et al. 2013). This alignment contained 16 sequences belonging to the candidate species from Príncipe, the five species of the Afro-Palearctic clade, including the mainland and Annobón subspecies of *O.senegalensis*, and *O.brucei*. This dataset was used to compute the mean genetic distances matrix (uncorrected p-distance in percentage, using the pairwise deletion option) within and between taxa. Distances were computed using MEGA, v. 7.0.21 (Kumar et al. 2016).

#### Dataset 2

Molecular dataset used for the phylogenetic and divergence time analyses. This dataset comprised 51 individuals from 39 taxa and a final concatenated sequence alignment of 12,925 bp. This dataset was built with the sequences produced in this study together with previously available sequences. We used homologous sequences of the Tawny Owl *Strixaluco* Linnaeus, 1758 and of the Eurasian Eagle-Owl *Bubobubo* (Linnaeus, 1758) for outgroup rooting. The input files for phylogenetic inference were prepared in “Pipelogeny” (Muñoz-Pajares et al. 2019). Sequences were automatically aligned using the mafft algorithm (Katoh and Standley 2013). The best model of molecular evolution and the best partition scheme to analyse the molecular dataset was identified using PartitionFinder2 v. 2.1.1 (Lanfear et al. 2012, 2016) using the greedy algorithm and applying the Bayesian Information Criterion (BIC).

Bayesian inference (BI) analyses were computed in MrBayes v. 3.2.6 (Ronquist et al. 2012). Two runs of 100 million generations (starting with random trees) and four incrementally heated Markov chains were performed, using default heating values and sampling the Markov chains at intervals of 1000 generations. The first 40% of trees were discarded as burn-in, and the remaining trees were retained and summed up to gene­rate a 50%-majority rule consensus tree. Chain mixing, stabilisation and convergence of likelihood values was assessed by examining the standard deviation of split frequencies. PartitionFinder and the partitioned BI analyses were run on the CIPRES gateway server (Miller et al. 2010) on XSEDE. The purpose of this phylogenetic analysis was: 1) to confirm that the representatives of the candidate species form a monophyletic group; 2) to infer the phylogenetic relationships of the candidate species within *Otus*.

We estimated divergence times (one partition per locus) using Beast 1.10.4 (Drummond et al. 2012). Substitution models for each marker were selected using MEGA X (Kumar et al. 2018) and used default prior distributions for the substitution models parameters. We specified uncorrelated lognormal clock models (Drummond and Rambaut 2007) for the seven mitochondrial loci and strict clock models for six nuclear loci. As calibrations, we used the substitutions rates and corresponding associated uncertainties from Lerner et al. (2011) and specified the following priors: ND2 – normal distribution with mean 0.029 and standard deviation 0.0029; ND3 – normal distribution with mean 0.024 and standard deviation 0.003; ATP6 – normal distribution with mean 0.026 and standard deviation 0.0026; CYTB – normal distribution with mean 0.014 and standard deviation 0.0012. We used uniform distributions for each of the nuclear loci (lower bound: 0.0, upper bound: 0.5). We specified a Birth-Death prior for the tree prior. MCMC chains were run for 50 million iterations with trees and parameters sampled every 1000 iterations. Analyses were run on the CIPRES 3.1 gateway server (Miller et al. 2010). We used Tracer v. 1.7 (Rambaut et al. 2018) to help ensure that the effective sample size for all Bayesian analyses of the underlying posterior distribution was adequate (> 200) for meaningful estimation of parameters.

#### Datasets 3–6

The alignments of each nuclear gene were analysed separately to obtain evidence for genetic differentiation of lineages from unlinked loci and, hence, to provide further support to their status as distinct species following the criterion of genealogical concordance (Avise and Ball 1990; Avise and Wollenberg 1997). These datasets contained the nuclear markers KIAA1239, MYO2, TGFB2 and TTN. In these four datasets we included all species of the Afro-Palearctic clade (sensu Pons et al. 2013) and, when available, se­quences of *O.brucei*. We trimmed all sequences to equal length (KIAA1239: 653 bp, MYO2: 131 bp, TGFB2: 403 bp, and TTN: 706 bp). Sequences were phased using the PHASE algorithm (v. 2.1.1) with default settings (Stephens et al. 2001) as implemented in the software DnaSP (v. 6.12.01; Rozas et al. 2017). PHASE parameters were 1000 iterations, one thinning interval and 100 burn-in iterations and a posterior probability threshold of 0.95 to determine the most probable inferred haplotypes for each nuclear sequence. Analyses were repeated three times with different seed values. Haplotype network reconstruction of phased sequences was performed using the software TCS, v. 1.21 (Clement et al. 2000). This software applies the method of Templeton et al. (1992) to calculate the minimum number of mutational steps between haplotypes, computing the probability of parsimony for pairwise differences until the probability exceeds 0.95. This analysis was used to detect the occurrence and extent of haplotype sharing in the studied *Otus* species. The resulting networks were plotted using the online tool tcsBU (Múrias dos Santos et al. 2016). We interpreted the lack of haplotype sharing among individuals of different phylogenetic lineages as independent evidence of their evolutionary distinctiveness.

### ﻿Nomenclature review

This published work and the nomenclatural acts it contains have been registered in ZooBank, the online registration system for the ICZN. The LSID (Life Science Identifier) for this publication is: urn:lsid:zoobank.org:pub:0731A37D-B363-43C9-A1AC-69F5E10F6810. The electronic edition of this work was published in a journal with an ISSN, and has been archived and is available from the following digital repositories: https://zookeys.pensoft.net/.

## ﻿Data availability

Molecular data are deposited in GenBank. Photographs and audio recordings are deposited in Macaulay Library and Xeno-canto, respectively. All other datasets underpinning this article are available as supplementary files (Suppl. materials 1–16).

## ﻿Results

### ﻿Justification for species delimitation

**Figure 3.**
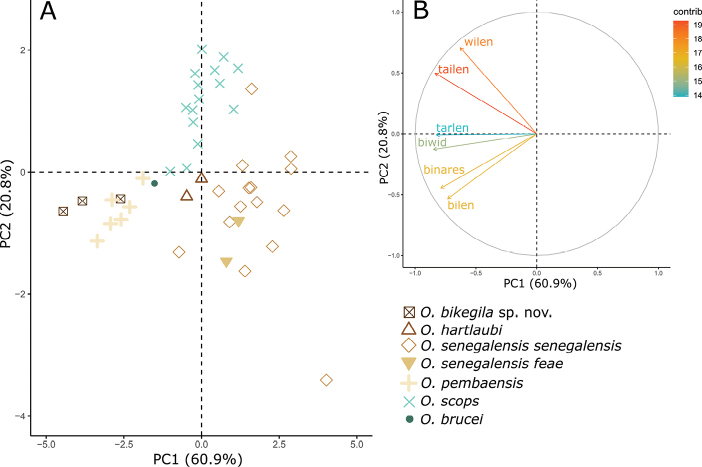
**A** Principal Component Analysis scatterplot of morphological measurements of *Otus* species and **B** the correlation circle in which ‘contrib’ corresponds to the contribution of the variables in accounting for the variability in the Principal components. Morphological measurement abbreviations – Bilen: bill length from bill tip to where culmen enters feathers; Binares: bill length from the anterior end of the nares to the tip; Biwid: bill width; Tarlen: tarsus length; Tailen: tail length; Wilen: wing length.

**Figure 4.**
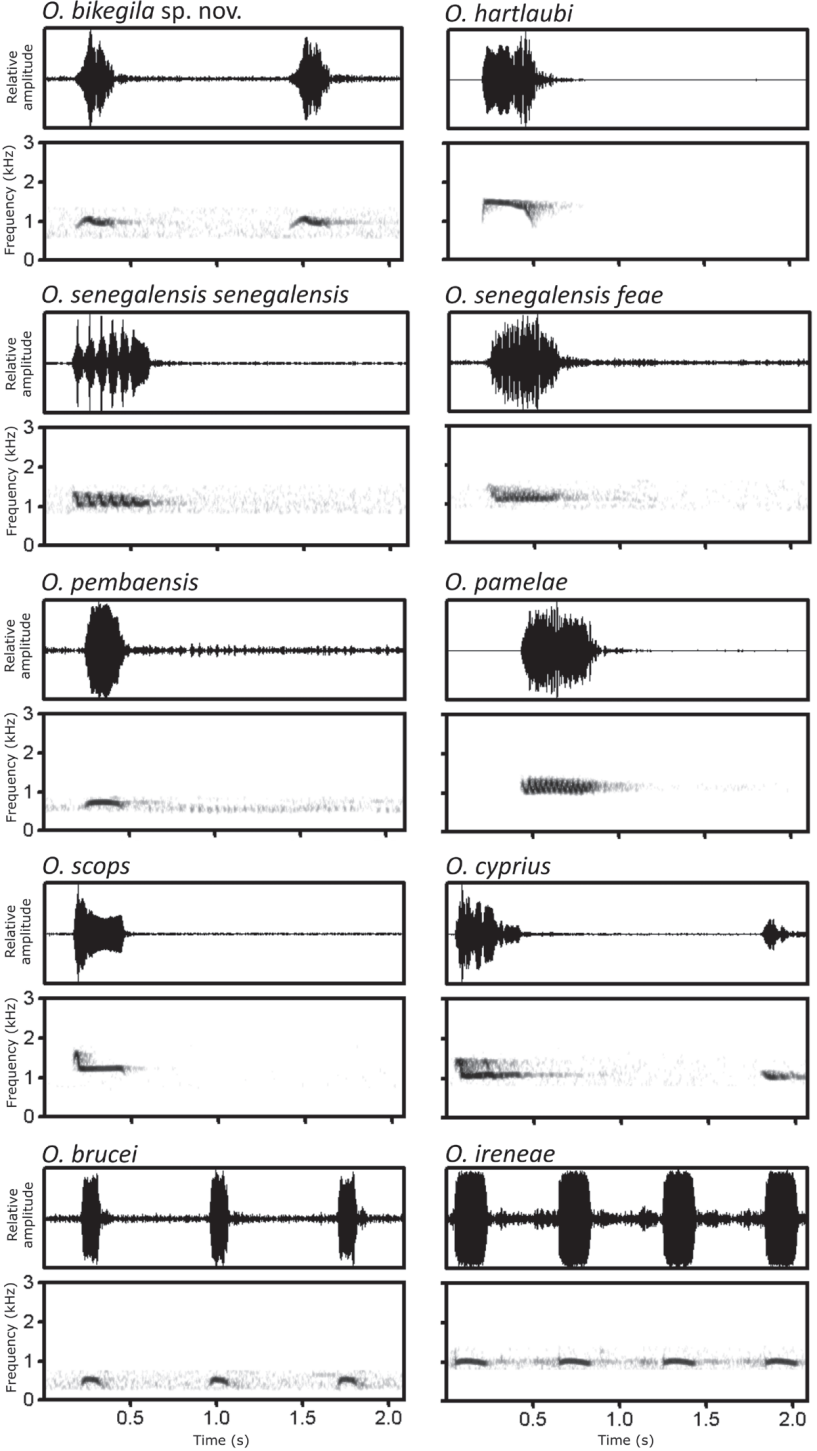
Oscillograms and spectrograms of 2-s sections of the song of *O.bikegila* sp. nov. (XC619448), *O.hartlaubi* (XC673669), *O.senegalensissenegalensis* (XC45502), *O.senegalensisfeae* (XC340505), *O.pembaensis* (XC253581), *O.pamelae* (XC371431), *O.scops* (XC383983), *O.cyprius* (XC256102), *O.brucei* (XC158086), and *O.ireneae* (XC147630). Each section refers to an individual owl. For more information about the recordings used see Suppl. material 7. Codes from Xeno-canto.org database.

**Figure 5.**
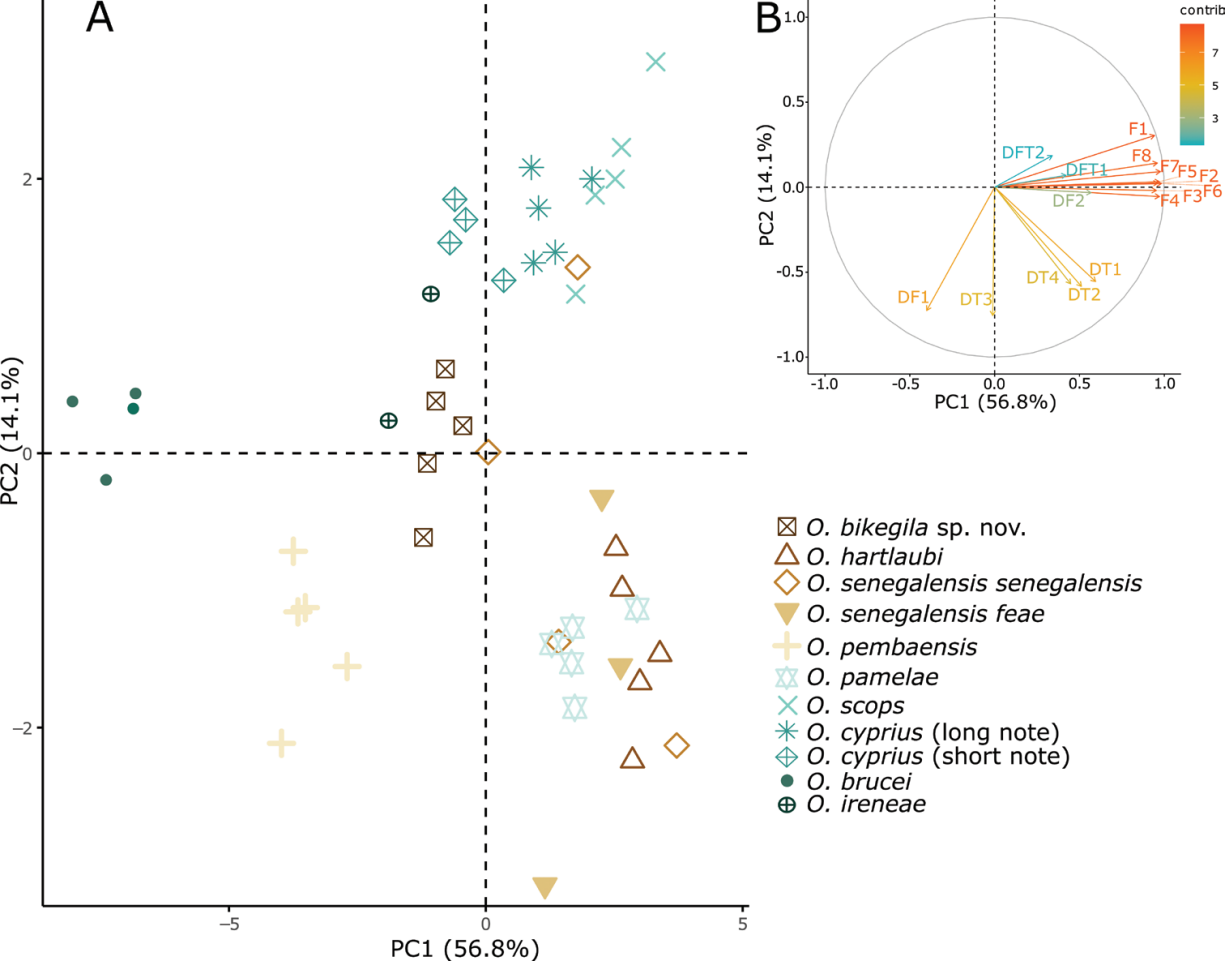
**A** Principal Component Analysis scatterplot of bioacoustics variables of *Otus* species and **B** the correlation circle in which ‘contrib’ corresponds to the contribution of the variables in accounting for the variability in the Principal components. Bioacoustic parameters – F1: frequency at start; F2: frequency at end; F3: frequency at 25% of total duration; F4: frequency at midpoint; F5: frequency at 75% of total duration; F6: frequency at maximum amplitude; F7: maximum frequency; F8: minimum frequency; DT1: total duration; DT2: time to maximum amplitude; DT3: time to maximum frequency; DT4: internote interval; DF1: frequency drop from start to end; DF2: frequency range; DFT1: slope from 25% to 75% of total duration; DFT2: slope from midpoint to end.

**Figure 6.**
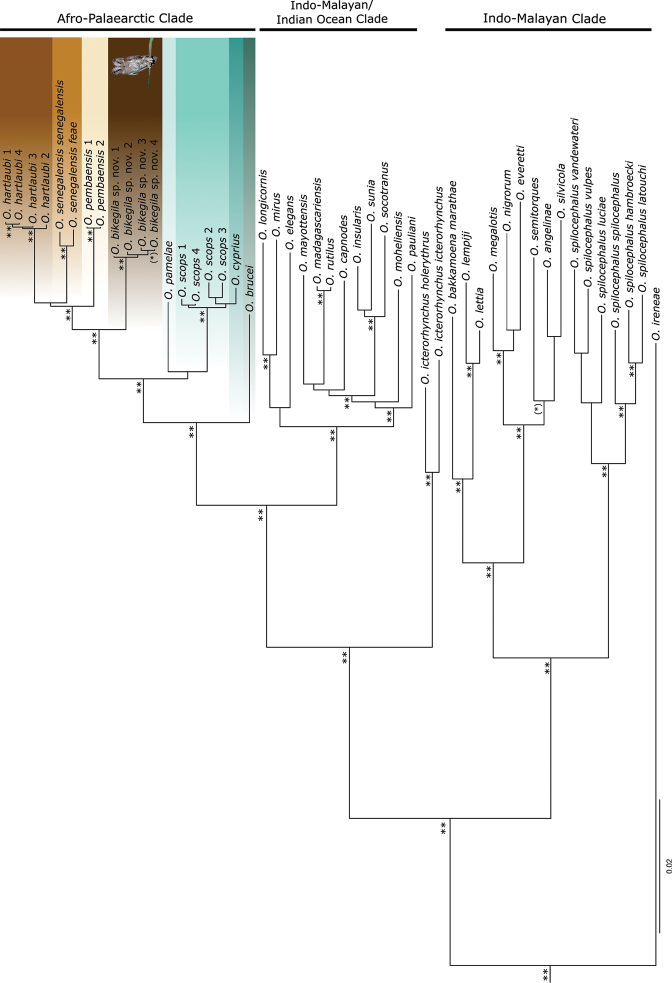
Multi-locus phylogeny of scops-owls (Strigidae: *Otus*). Phylogram (50% majority rule consensus tree) from a Bayesian Inference analysis of the dataset 2, including 12S, 16S, ATP6, COI, CYTB, ND2, ND3, KIAA, MYO2, RAG1, TGFB2, and TTN gene fragments. Asterisks denote posterior probabilities values: (*) 0.85–0.94, * 0.95–0.98, ** 0.99–1. Scale bar corresponds to 0.02 substitutions per site. *Strixaluco* was set as outgroups (not shown on the figure).

**Figure 7.**
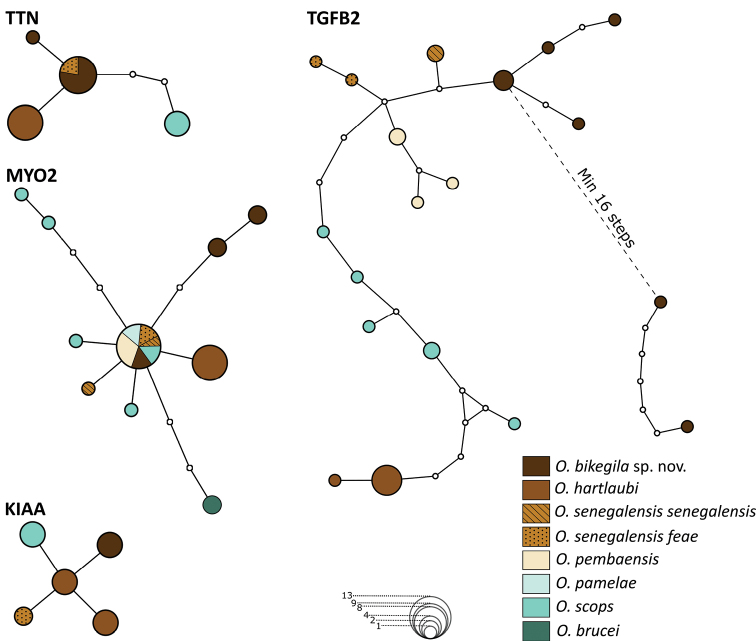
Haplotype network reconstruction for the nuclear KIAA, MYO2, TGFB2, and TTN gene fragments in *Otusbikegila* sp. nov., *O.hartlaubi*, *O.senegalensissenegalensis*, *O.senegalensisfeae*, *O.pembaensis*, *O.pamelae*, *O.scops*, and *O.brucei* (when available). Area of circles is proportional to the number of individuals with that haplotype. The smallest circles (white) represent unsampled or extinct haplotypes.

Following the integration by congruence approach (Padial et al. 2010), we consider independent evolutionary lineages as separate species if two or more independent lines of evidence support their distinctiveness. The candidate species: i) differs in morphology from other *Otus* species (Fig. 3); ii) has a very distinct call (Tables 2, 3; Figs 4, 5); iii) forms a distinct monophyletic group (Fig. 6) that iv) differs from the nearest taxa by a mitochondrial uncorrected pairwise sequence divergence (*p*-distance) similar or larger than the divergence observed between other currently accepted sister species pairs of the genus *Otus* (Table 5); v) has no haplotype sharing at some of the analysed nuclear markers (Fig. 7). We interpret the concordance between these independent lines of evidence as strong support for its distinctiveness and species status (Avise and Ball 1990; Padial et al. 2010; Sangster 2018). Therefore, we conclude that the candidate species from Príncipe is a distinct species that we describe herein as *Otusbikegila* sp. nov., and for convenience we will use this name throughout the manuscript, anticipating its formal description below.

**Table 5.** Genetic divergence, in % base pairs difference, between and within (bold) *Otus* taxa of the Afro-Palearctic clade, estimated from uncorrected pairwise distances of the ND2 fragment (1037 bp). For taxa with a single sample, within-taxon variation could not be calculated (nc).

Taxon		1	2	3	4	5	6	7	8
*O.bikegila* sp. nov. (*n* = 4)	1	**0.0**%							
*O.hartlaubi* (*n* = 4)	2	4.1%	**0.1**%						
*O.senegalensissenegalensis* (*n* = 1)	3	4.4%	3.3%	**nc**					
*O.senegalensisfeae* (*n* = 1)	4	4.7%	3.6%	0.7%	**nc**				
*O.pembaensis* (*n* = 2)	5	4.5%	3.9%	4.2%	4.5%	**0.0**%			
*O.pamelae* (*n* = 1)	6	6.2%	3.6%	3.4%	3.0%	4.5%	**nc**		
*O.scops* (*n* = 2)	7	4.1%	4.7%	4.9%	5.0%	5.3%	3.2%	**0.1**%	
*O.brucei* (*n* = 1)	8	9.1%	7.0%	6.9%	6.5%	7.1%	6.7%	7.7%	**nc**

### ﻿Morphological differentiation

In the PCA, the first two components presented eigenvalues higher than one (Suppl. material 11), representing 81.7% of the variation. PC1 (60.9% of the variance) was negatively correlated with all variables related with bill, tarsus length and tail length, whereas PC2 (20.8% of the variance) was positively correlated with wing length (Suppl. material 11). *Otusbikegila* sp. nov. is within the variation of *O.pembaensis* and separated from the other species (Fig. 3). All other taxa are separated from each other, except *O.senegalensisfeae* that is within the variation of *O.senegalensissenegalensis* (Swainson, 1837).

For the Welch’s ANOVAs, *O.bruceiobsoletus* was not included due to the small sample size. All variables differed significantly between species (*P* < 0.05), except Bidepth (Suppl. material 12). Games-Howell post-hoc test provided significant values for all the species only for Bilen and Biwid because of missing data from some specimens. This test was used to identify the morphological diagnostic characters detailed in the Diagnosis section.

### ﻿Bioacoustic differentiation

In the PCA, four components presented eigenvalues higher than one (Suppl. material 13), representing 87.6% of the variation. PC1 (56.8% of the variance) mostly represented frequency characteristics of the note, being positively correlated with the frequency variables F1-F8, but also with DT1. PC2 (14.1% of the variance) mostly represented temporal characteristics of the note, being negatively correlated with DT2, DT3, DT4, and DF1. PC3 (9.1% of the variance) was negatively correlated with DFT1 and DFT2, and PC4 (7.6% of the variance) was positively correlated with DF2. Plotting individuals on PC1 versus PC2 (Fig. 5) confirmed our hearing-based assessment that the calls of *O.bikegila* sp. nov. are unique but closest to the ones from *O.ireneae*, both characterised by short notes repeated at a fast rate (Fig. 4). One sample of *O.senegalensissenegalensis*, also came close to those of *O.bikegila* sp. nov. but, otherwise, the calls of the new species are clearly separated from the other species of the Afro-Palearctic clade. Samples of *O.brucei*, *O.pembaensis* and the short note of *O.cyprius* are clearly distinct from all other species. Samples of the long note of *O.cyprius* overlap with samples of *O.scops*. Samples of *O.pamelae* and *O.hartlaubi* are contained in the variation of *O.senegalensissenegalensis* and samples of *O.senegalensisfeae* and *O.scops* overlap with it.

Means of all bioacoustic variables differed significantly (*P* < 0.05) between species (Suppl. material 14). Low sample size prevented performing the Games-Howell post-hoc test between all taxa pairs. This test identified the bioacoustic diagnostic characters detailed in the Diagnosis section.

### ﻿Molecular differentiation

The ND2 sequences (Dataset 1; 1037 pb) of the four samples of *Otusbikegila* sp. nov. were identical (Table 5). *Otusbikegila* sp. nov. is a distinct mitochondrial lineage (Dataset 2; Fig. 6), belonging to the Afro-Palearctic clade. The genetic distance between this species and the other taxa included in Dataset 1 ranged between 4.1% (*O.bikegila* sp. nov. vs. *O.hartlaubi* and *O.scops*) and 9.1% (*O.bikegila* sp. nov. vs. *O.bruceiobsoletus*). The smallest genetic distances were observed between *O.senegalensissenegalensis* and *O.senegalensisfeae* (0.7%), followed by *O.senegalensisfeae* and *O.pamelae* (3.0%); the highest value was recorded between *O.bikegila* sp. nov and *O.brucei*/*O.hartlaubi* (9.1%).

The concatenated sequences of the phylogenetic dataset (Dataset 2; 12,925 bp; Suppl. material 2) were optimally partitioned in seven partitions (Suppl. material 15).

The topology of the majority rule consensus tree (Dataset 2; Fig. 6) is largely in agreement with previously published phylogenies of the genus *Otus* (Fuchs et al. 2008; Pons et al. 2013), and increased the resolution of these by resolving some po­lytomies. An important improvement relatively to Fuchs et al. (2008) and Pons et al. (2013) was the inclusion of 13 additional taxa besides *O.bikegila* sp. nov. *Otusicterorhynchus* was found to represent a relatively basal lineage, sister to the clades containing the Afro-Palearctic and the Indo-Malayan/Indian Ocean species (*PP* = 1), rather than being sister to *O.ireneae*, as often hypothesised. The two subspecies of *O.icterorhynchus* were recovered as sister taxa, albeit with a very high genetic divergence (only one sample per taxon). *Otusbrucei* (from the Arabian Peninsula to Asia) was the sister lineage to the Afro-Palearctic clade (*PP* = 1). Other novel insights are detailed in the discussion.

*Otusbikegila* sp. nov. samples were recovered as monophyletic, and formed a clearly distinct lineage belonging to the Afro-Palearctic clade. It was recovered as the sister lineage (*PP* = 1) of the clade formed by *O.senegalensissenegalensis*, *O.senegalensisfeae*, *O.hartlaubi*, and *O.pembaensis* (Fig. 6).

The best model of sequence evolution for each marker used for the divergence times analyses are listed in the Suppl. material 16. The genus *Otus* started to diversify ca. 7.8 mya (95% high posterior density [HPD]: 6.2–9.6). The two primary cla­des diverged at ca. 6.3 mya (95% HPD: 5.1–7.7), and went on to diversify at similar times: i) *O.icterorhynchus*/*O.moheliensis*: 4.3 mya (95% HPD: 3.3–5.3), and ii) *O.spilocephalus*/*O.bakkamoenamarathae* clade 4.6 mya (95% HPD: 3.7–5.6). *Otusbikegila* sp. nov. diverged from the *O.hartlaubi*/*O.pembaensis*/*O.senegalensis* clade ca. 0.9 mya ago (95% HPD: 0.7–1.1), an estimate similar (e.g., *O.mirus*/*O.longicornis*: 1 mya 95% HPD: 0.7–1.3) or greater (*O.socotranus*/*O.insularis*/*O.sunia*: 0.8 mya 95% HDP: 0.6–1.1) than the divergence estimated between closely related and well accepted species.

Nuclear markers independently supported the evolutionary independence of the taxa of the Afro-Palearctic clade. The taxa included in the analysis shared no haplotypes for markers KIAA1239 and TGFB2; the latter was the most variable of the analysed nuclear markers with a total of 19 haplotypes (Fig. 7). For MYO2, *O.hartlaubi* and *O.brucei* had no shared haplotypes, whereas *O.bikegila* sp. nov., *O.scops* and *O.senegalensis* had some unique haplotypes but shared the most common one (Fig. 7). *Otushartlaubi* and *O.scops* did not share any TTN haplotype, with *O.bikegila* sp. nov. having both one unique haplotype and one shared with *O.senegalensisfeae* (Fig. 7).

#### ﻿ Otus bikegila sp. nov.

Taxon classificationAnimaliaStrigiformesStrigidae

BB7A16EB-7B86-5066-A67C-B1617E7D3C25

https://zoobank.org/0731A37D-B363-43C9-A1AC-69F5E10F6810

Figs 2A , 8

##### Material.

***Holotype*.**MHNC-UP-AVE7000: São Tomé and Príncipe • ♀, adult, moulting; Príncipe Island, South Príncipe, ca. 500 m NW of Ribeira Porco river mouth (Fig. 1); 1°33.03'N, 7°22.29'E; ca. 100 m a.s.l.; 29 May 2017; HP and Ceciliano do Bom Jesus leg.; skin prepared by Vanya Rohwer, skeleton prepared by Vanya Rohwer and Daniele Cataldo; left wing removed for wing mounting, all bones with the exception of the left tarso-metatarsus (tarsus) removed for skeleton preparation; Audio-recorded by HP (Xeno-canto audio: XC619445, XC619447; GenBank: 12S OM978880, 16S OM978895, ATP6OM913485, COI, OM937282; CYTB, OM937307; ND2, OM937351; ND3, ON016156; KIAA1239, OM937319; MYO2, OM937336; RAG1, ON016107; SACS, ON016118; TGFB2, ON016136 and TTN, ON016141; Gulf of Guinea database of MM: P7-04.

##### Diagnosis.

The new species (Figs 2, 9) is assigned to the genus *Otus* based on genetic and morphological similarities to other known species of this genus. Phylogenetic analyses place it within the Afro-Palearctic clade, making generic placement unambiguous. Placement of the new species in *Otus* is further supported by its morphological characters: small size, distinctive ear-tufts, facial disc, short rounded wings, and short tail. The new species differs from the other described taxa of the Afro-Palearctic *Otus* clade (*O.hartlaubi*, *O.senegalensis*, including *O.s.feae* sometimes treated as a distinct species, *O.pembaensis*, *O.pamelae*, *O.scops*, *O.brucei*) by high genetic differentiation (pairwise ND2 distance ranging from 4.1% to 9.1%), by the lack of haplotype sha­ring at the KIAA1239 and TGFB2 nuclear markers, as well as from a combination of morphological, genetic and natural history (bioacoustics) traits.

**Figure 8.**
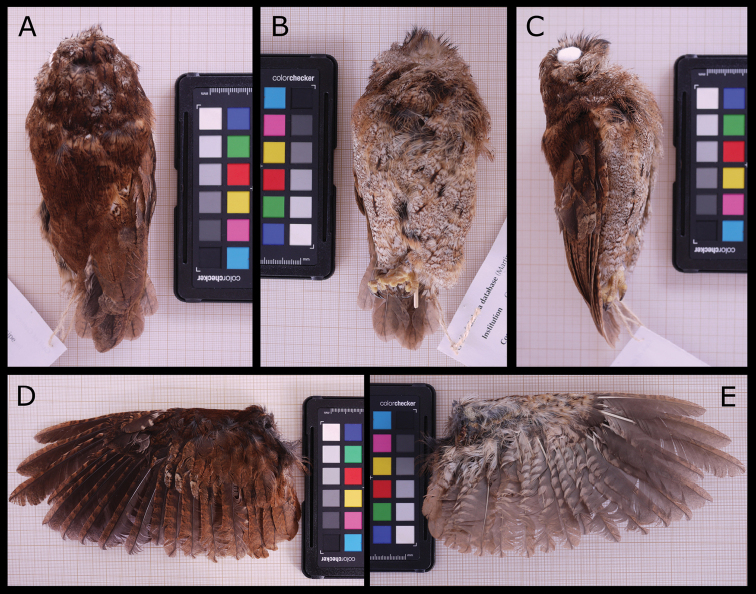
*Otusbikegila* sp. nov., female holotype (MHNC-UP-AVE7000). Views **A** dorsal **B** ventral **C** lateral **D** left wing top and **E** left wing under. Millimetric paper on the background and colour reference plate (Colorchecker, X-Rite Inc.) for size and colour assessment.

**Figure 9.**
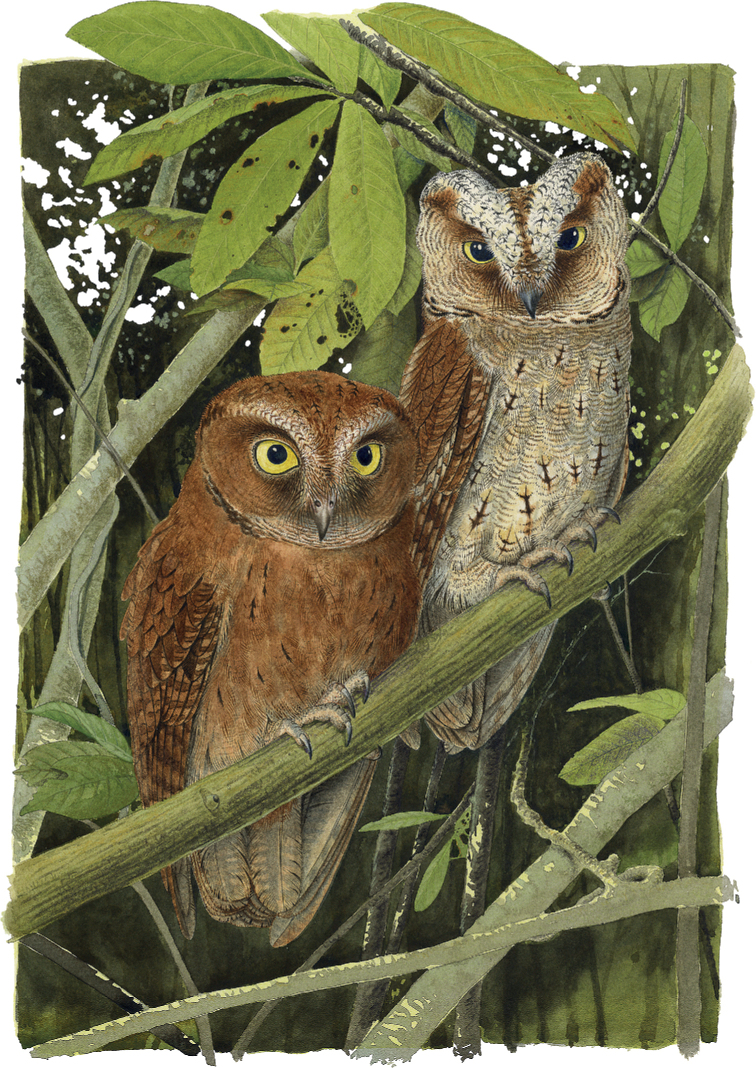
Principe Scops-Owl *Otusbikegila* sp. nov. from Príncipe Island, Africa. Left: Adult rufous morph in the typical posture. Right: Adult grey-brown morph in a stress posture, when it raises the ear tufts to increase the efficiency of camouflage. Original artwork by MNC.

We provide here a diagnosis relatively to the closely related species belonging to the Afro-Palearctic clade and also to *O.ireneae* due to the similarity in their calls. The diagnosis is based on the following analysed morphological characters: 1) Biwid; 2) Binares; 3) Tarlen; 4) Wilen; 5) Tailen); 6) SP10; 7) SP9; 8) SP4; on the following analysed bioacoustics characters: 9) F1; 10) F2; 11) F3; 12) F4; 13) F5; 14) F6; 15) F7; 16) F8; 17) DT1; 18) DT2; 19) DT4; 20) DF1; 21) DF2; 22) DFT1; 23) DFT2; and on the 24) list of diagnostic substitutions identified at the analysed nuclear mar­kers (Tables 1–4; Suppl. materials 12, 14).

In overall appearance, *O.bikegila* sp. nov. is most similar to *O.hartlaubi* from which it differs in one morphological and 10 bioacoustic characters: longer Wilen (145 to 151 mm vs. 130 to 139 mm), lower F1 (781.7 to 961.7 Hz vs. 1078.3 to 1360.0 Hz), lower F2 (933.3 to 1020.0 Hz vs. 1155.0 to 1285.0 Hz), lower F3 (980.0 to 1090.0 Hz vs. 1330.0 to 1478.3 Hz), lower F4 (950.0 to 1050.0 Hz vs. 1295.0 to 1483.3 Hz), lower F5 (931.7 to 1020.0 Hz vs. 1250.0 to 1418.3 Hz), lower F6 (976.7 to 1090.0 Hz vs. 1331.7 to 1480.0 Hz), lower F7 (1035.0 to 1106.7 Hz vs. 1407.5 to 1526.7 Hz), lower F8 (868.3 to 973.3 Hz vs. 1066.7 to 1267.5 Hz), shorter DT1 (0.231 to 0.248 s vs. 0.267 to 0.315 s), shorter DT4 (0.992 to 1.121 s vs. 9.181 to 15.998 s). *Otusbikegila* sp. nov. differs from *O.hartlaubi* also by the following molecular characters: KIAA (T vs. C in site 347); TTN (G vs. C in site 91); MYO2 (G vs. A in site 2); TGFB2 (C vs. A in site 28, G vs. A in site 33, G vs. T in site 47, T vs. C in site 99, A vs. G in site 178, G vs. T in site 305, C vs. G in site 369).

*Otusbikegila* sp. nov. differs from *O.senegalensissenegalensis* in seven morphological and one bioacoustic characters: higher Biwid (9.0 to 11.8 mm vs. 4.8 to 8.0 mm), larger Binares (11.3 to 12.6 mm vs. 8.0 to 12.5 mm), longer Tarlen (30.5 to 35.1 mm vs. 20.0 to 24.2 mm), longer Wilen (145 to 151 mm vs. 103 to 145 mm), longer Tailen (75 to 85 mm vs. 40 to 65 mm), longer SP10 (37 to 40 mm vs. 10 to 32 mm), longer SP9 (13 to 18 mm vs. 1 to 16 mm), lower F4 (950.0 to 1050.0 Hz vs. 1095.0 to 1236.7 Hz); and in the following molecular characters: TGFB2 (A vs. G in site 178, T vs. G in site 344).

*Otusbikegila* sp. nov. differs from *O.senegalensisfeae* in one morphological and four bioacoustic characters: larger Biwid (9.0 to 11.8 mm vs. 6.4 to 6.8 mm), lower F2 (933.3 to 1020.0 Hz vs. 1132.5 to 1230.0 Hz), lower F7 (1035.0 to 1106.7 Hz vs. 1250.0 to 1357.5 Hz), shorter DT1 (0.231 to 0.248 s vs. 0.402 to 0.441 s), shorter DT4 (0.992 to 1.121 s vs. 7.127 to 7.479 s); and in the following molecular characters: KIAA (T vs. C in site 347, T vs. C in site 503); TGFB2 (A vs. G in site 178, T vs. C in site 285, G vs. C in site 392).

*Otusbikegila* sp. nov. differs from *O.pembaensis* in one morphological and nine bioacoustic characters: shorter SP4 (6 to 9.5 mm vs. 11 to 21 mm), higher F1 (781.7 to 961.7 Hz vs. 506.7 to 636.7 Hz), higher F2 (933.3 to 1020.0 Hz vs. 613.3 to 773.3 Hz), higher F3 (980.0 to 1090.0 Hz vs. 621.7 to 770.0 Hz), higher F4 (950.0 to 1050.0 Hz vs. 633.3 to 780.0 Hz), higher F5 (931.7 to 1020.0 Hz vs. 651.7 to 783.3 Hz), higher F6 (976.7 to 1090.0 Hz vs. 638.3 to 776.7 Hz), higher F7 (1035.0 to 1106.7 Hz vs. 660.0 to 790.0 Hz), higher F8 (868.3 to 973.3 Hz vs. 530.0 to 660.0 Hz), shorter DT4 (0.992 to 1.121 s vs. 5.043 to 7.570 s); and in the following molecular characters: TGFB2 (A vs. G in site 178, T vs. C in site 285, C vs. T in site 345).

*Otusbikegila* sp. nov. differs from *O.pamelae* in seven bioacoustic characters (morphology not analysed): lower F1 (781.7 to 961.7 Hz vs. 1031.7 to 1213.3 Hz), lower F2 (933.3 to 1020.0 Hz vs. 1111.7 to 1268.3 Hz), lower F4 (950.0 to 1050.0 Hz vs. 1096.7 to 1366.7 Hz), lower F5 (931.7 to 1020.0 Hz vs. 1083.3 to 1291.7 Hz), lower F6 (976.7 to 1090.0 Hz vs. 1160.0 to 1403.3 Hz), lower F7 (1035.0 to 1106.7 Hz vs. 1220.0 to 1516.7 Hz), lower F8 (868.3 to 973.3 Hz vs. 1020.0 to 1123.3 Hz).

*Otusbikegila* sp. nov. differs from *O.scops* in seven morphological and 11 bioacoustic characters: higher Biwid (9.0 to 11.8 mm vs. 5.5 to 7.8 mm), larger Binares (11.3 to 12.6 mm vs. 9.0 to 11.5 mm), longer Tarlen (30.5 to 35.1 mm vs. 22.0 to 28.0 mm), shorter Wilen (145 to 151 mm vs. 147 to 165 mm), longer SP10 (37 to 40 mm vs. 12 to 24 mm), longer SP9 (13 to 18 mm vs. 0 to 7 mm), shorter SP4 (6 to 9.5 mm vs. 10 to 29 mm), lower F1 (781.7 to 961.7 Hz vs. 1335.0 to 1695.0 Hz), lower F2 (933.3 to 1020.0 Hz vs. 1152.5 to 1275.0 Hz), lower F3 (980.0 to 1090.0 Hz vs. 1155.0 to 1260.0 Hz), lower F4 (950.0 to 1050.0 Hz vs. 1200.0 to 1285.0 Hz), lower F5 (931.7 to 1020.0 Hz vs. 1193.3 to 1318.3 Hz), lower F6 (976.7 to 1090.0 Hz vs. 1210.0 to 1326.7 Hz), lower F7 (1035.0 to 1106.7 Hz vs. 1336.3 to 1628.3 Hz), lower F8 (868.3 to 973.3 Hz vs. 1140.0 to 1253.3 Hz), shorter DT4 (0.992 to 1.121 s vs. 2.423 to 2.789 s), higher DF1 (6.7 to 178.3 Hz vs. -420.0 to -182.5 Hz), lower DFT1 (-581.5 to 45.2 Hz/s vs. 181.5 to 662.3 Hz/s); and in the following molecular characters: KIAA (T vs. C in site 347, A vs. G in site 632); TTN (T vs. C in site 535, G vs. A in site 536, G vs. T in site 634); TGFB2 (G vs. A in site 33, G vs. T in site 47, A vs. G in site 178, T vs. C in site 285).

*Otusbikegila* sp. nov. differs from *O.cyprius* in four bioacoustics characters (morphology and nuclear markers not analysed): presence of a monosyllabic primary song (*O.cyprius* has a distinctive di-syllabic primary song), lower F1 (781.7 to 961.7 Hz vs. 1236.7 to 1400.0 Hz [long note] and 1058.9 to 1223.3 Hz [short note]), shorter DT4 (0.992 to 1.121 s vs. 3.035 to 3.643 s [long note] and 3.116 to 3.714 [short note]), and having a higher DF1 (6.7 to 178.3 Hz vs. -283.3 to -165.0 Hz [long note] and -113.3 to -58.1 [short note]).

*Otusbikegila* sp. nov. differs from *O.brucei* in 11 bioacoustic characters (morphology not analysed): higher F1 (781.7 to 961.7 Hz vs. 285.0 to 388.3 Hz), higher F2 (933.3 to 1020.0 Hz vs. 891.7 to 946.7 Hz), higher F3 (980.0 to 1090.0 Hz vs. 356.7 to 530.0 Hz), higher F4 (950.0 to 1050.0 Hz vs. 356.7 to 493.3 Hz), higher F5 (931.7 to 1020.0 Hz vs. 335.0 to 450.0 Hz), higher F6 (976.7 to 1090.0 Hz vs. 370.0 to 510.0 Hz), higher F7 (1035.0 to 1106.7 Hz vs. 370.0 to 530.0 Hz), higher F8 (868.3 to 973.3 Hz vs. 270.0 to 373.3 Hz), longer DT1 (0.231 to 0.248 s vs. 0.090 to 0.133 s), longer DT2 (0.078 to 0.112 s vs. 0.032 to 0.053 s), higher DFT2 (-250.3 to 21.7 Hz/s vs. -1653.0 to -1387.3 Hz/s); and in the following molecular characters: MYO2 (T vs. C in site 22; C vs. T in site 118; C vs. T in site 129).

*Otusbikegila* sp. nov. differs from *O.ireneae* in one bioacoustic character (morpho­logy and nuclear markers not analysed): longer DT4 (0.992 to 1.121 s vs. 0.409 to 0.447 s).

##### Description of the holotype.

Morphological measurements available in Table 1. The topographic terms of the scops-owl body are detailed in the Suppl. material 3.

***General colouration***: Back, Burnt Amber 48 with Robin Rufous 29 shades; front, Pale Buff 1 feathers with Cinnamon 21 and Dusky Brown 285 markings (forming stripes defined by Dusky Brown 285 lines) with Robin Rufous 29 shades.

***Head***: Chin feathers Pale Buff 1 with Sayal Brown 41 shading along shaft, ending with a bristle-like barb Sepia 286. Throat feathers Pale Buff 1 with Pale Pinkish Buff 3 and Sepia 286 dots and markings sometimes forming bands; Pale Buff 1 shaft proximally becoming Pale Pinkish Buff 3 and Sepia 286 distally. Feathers of forehead Sepia 286 and few Pale Buff 1 shading. Tip of the head triangle Prout’s Brown 47. Triangle outside facial disk with an overall appearance Prout’s Brown 47, triangle with feather with Sepia 286 middle stripe along shaft, Prout’s Brown 47 and Sepia 286 in their internal portion and Sepia 286 and Pale Buff 1 in the outer portion but always ending with Prout’s Brown 47 or Robin Rufous 29 in the distal portion. Triangle delineated by Pale Buff 1/Smoke Grey 266 stripes (the eyebrows). Eyebrows Pale Buff 1/Smoke Grey 266 down to the bill: Pale Buff 1 feathers ending with a thin Cinnamon 21 line followed by a broader Jet Black 300 band; Pale Buff 1 feathers with middle Sepia 286 stripe along shaft and densely vermiculated with Sepia 286 and Cinnamon 21 shades; eyebrows feathers in distal portion are Pale Buff 1 densely vermiculated with Sepia 286 and Cinnamon 21 shades, ending in Prout’s Brown 47. Crown feathers Prout’s Brown 47 with Sepia 286 middle stripe along shaft, Pale Buff 1 in proximal section and Prout’s Brown 47 with Sepia 286 vermiculation along mid and distal portion. Ear tuft not visible in the mounted specimen, but with feathers Pale buff 1 in proximal section becoming Cinnamon 21 with densely Sepia 286 vermiculation and ending with Prout’s Brown 47; ear feathers pull up some of the eyebrow feather with Sepia 286 and Cinnamon 21 dense vermiculation. Nape feathers Pale Pinkish Buff 3 with well-defined Sepia 286 irregular stripes, ending with middle Sepia 286 stripe along shaft and Burnt Amber 48. Neck feathers with longer underfeathers (then in nape) with middle Sepia 286 stripe along shaft with Pale Pinkish Buff 3, Pale Buff 1, Pale Pinkish Buff 3 and becoming Pale Buff 1 and Burnt Amber 48 all with Sepia 286 irregular markings. Overall appearance of rictal bristles: Jet Black 300 patches with Cinnamon 21 and Pale Buff 1 shades next to the bill (following with Pale Buff 1/Smoke Grey 266 eyebrows); bristles with terminal Jet Black 300 colour; bristles closer to the bill Pale Buff 1 in proximal position, changing into Cinnamon 21 and ending in Jet Black 300 or Pale Buff 1 in the proximal section and Jet Black 300 in distal section; bristles closer to the eye Jet Black 300 in proximal position, changing into Cinnamon 21 and ending in Jet Black 300 or only Jet Black 300 (the shortest one). Rim with two narrow Raw Umber 23 bands, one on each side, not extending to the centre; Pale Buff 1 feathers with Sepia 286 irregular markings, becoming Cinnamon 21 (with no Sepia 286 markings), ending with Raw Umber 23. Facial disk feathers Pale Buff 1 with multiple bands of Sepia 286 (generally 3), the terminal Sepia 286 bands is preceded by a thin Robin Rufous 29 band; feather ending with 2 to 5 bristle-like barbs.

***Upperparts***: Overall colour of mantle (i.e., upper back) and rump: Burnt Amber 48 with Robin Rufous 29 shades. Feathers Sayal Brown 41 with middle Sepia 286 line along shaft, and with irregular Sepia 286 markings in the proximal section. Feathers turning Cinnamon-Rufous 31 with Sepia 286 markings in the distal portion of the feather. Mantle is delimited distally (neck) by a Cinnamon-Rufous 31 band and late­rally by a series of 8 feathers that are lighter in colour (Cinnamon-Rufous 31): outer vane Light Buff 2 with Cinnamon-Rufous 31 shades and with a Sepia 286 curve line that defines a Raw Sienna 32 colour close to shaft, one or more Sepia 286 spots on distal outer vane; outer vane ending distally with Raw Sienna 32 with Sepia 286 mar­kings; inner vane is Raw Sienna 32 with Sepia 286 irregular markings. These feathers appear to make a line that delineates the outside of the mantle. Similarly, the feathers of the mantle at the base of the neck form a lighter Cinnamon Rufous 31 line that follows the external side of the folded wings, making a triangle. Scapulars as upperparts. Proximal shaft Pale Buff 1, Vandyke Brown 282 in distal portion; Vandyke Brown 282 middle stripe along shaft. Outer vane is Cinnamon 21 with Sepia 279 markings and inner vane is Sepia 279 with Cinnamon 21 markings.

***Underparts***: Breast overall Pale Buff 1 with Sepia 286 irregular markings and Robin Rufous 29 shading. Breast feathers Pale Pinkish Buff 3 with Sepia 286 dots and markings proximally and Pale Pinkish Buff 3 shaft, distally Sepia 286 with Pale Pinkish Buff 3 dots and markings forming irregular bands, middle Sepia 286 stripes along shaft. Belly overall similar to breast but with colours more defined and with Light Buff 2 shadings. Belly feathers Pale Pinkish Buff 3 in proximal section followed by a Sepia 286 V stripe. This is followed by a Pale Buff 1 broad band delimitated distally with a thin Pale Pinkish Buff 3 line followed by a Sepia 286 line. Distally, these feathers are Pale Buff 1 with irregular spots Sepia 286 and Pale Pinkish Buff 3. Some feathers on the belly and the vent have a marked middle and broad Sepia 286 line along shaft. Vent is similar to breast and belly but with feathers Cinnamon 21 in proximal section followed by a Sepia 286 stripe. This is followed by a Pale Buff 1 broad band delimitated distally with a thin Cinnamon 21 followed by a Sepia 286 line. The feather then becomes Pale Buff 1 with a Cinnamon 21 thin band followed by a Sepia 286 line, ending with a Pale Buff 1 colouration with Sepia 286 irregular dots and markings. Flank feathers Pale Buff 1 with Cinnamon 21 shading followed by a broad Cinnamon 21 V stripe followed by a Sepia 286 line, ending with a broad Pale Buff 1 section with Sepia 286 markings only in the very distal portion. Undertail coverts are similar to flanks but with more defined bands. Feathers are Pale Buff 1 followed by a broad Cinnamon 21 band defined distally by a thinner warm Sepia 40 line. This colouration is repeated twice. Feathers end with a broad Pale Buff 1 band followed by a Cinnamon 21 band with irregular Warn Sepia 40 markings. Tarsus covered with feathers to base of toes. Feathers overall similar to flank but with less Pale Buff 1 and more Cinnamon 21 shading and one or two Sepia 286 dots in distal section. Tarsus fea­thers have a larger proportion of Pale Buff 1 close to toes. Tarsus feathers are Pale Buff 1 proximally, followed by Cinnamon 21 shading and Sepia 286 markings distally (approximately 1/4 of the feather distally), no middle stripe along shaft. Toes feathers are Pale Buff 1 with Cinnamon 21 shadings and Sepia 286 markings only in the distal section.

***Wing***: Overall Prout’s Brown 47 with Dark Greyish Brown 284 leopard blotches. Primaries shaft Vandyke Brown 282. Outer vane of primaries with six or seven ‘leopard’ Dark Greyish Brown 284 spots in Cinnamon 21 background becoming Pale Buff 1 in some instances. Spots interior with a gradient of Cinnamon 21 to Dark Greyish Brown 284 with lighter spots on outer primaries. Spots circumference with Dark Greyish Brown 284 to Jet Black 300. ‘Leopard spots’ start faint (P1-P2-P3) and become stronger moving outwards. Inner vane of primaries with Dark Greyish Brown 284 with Cinnamon 21 shadings towards the distal section of the feather. Exterior edge makes a Pale Buff 1 line. Under-primaries have a Pale Buff 1 shaft proximally becoming Cinnamon 21 towards the distal portion. Outer vane of under-primaries is proximally Hair Brown 277 with Pale Pinkish Buff 3 irregular triangles, becoming Cinnamon 21 in distal section with Sepia 279 lines delimiting the leopard spots that are fading towards the distal portion of the feather. Inner vane of under-primaries is Hair Brown 277 with Light Buff 2 markings in proximal section and Cinnamon 21 markings in distal section. Secondaries shaft Vandyke Brown 282. Outer vane of secondaries is similar but much less marked pattern than primaries: spots on outer vanes less marked, fading into the background towards S10. Inner vane of seconda­ries with Sepia 279 with Cinnamon 21 shadings and markings especially towards the distal section of the feather. Under-secondaries have a Pale Buff 1 shaft proximally becoming Cinnamon 21 and later Sepia 279 towards the distal portion. Outer vane of under-secondaries with Hair Brown 277 with Cinnamon 21 markings. Inner vane of under-secondaries with Hair Brown 277 with six to seven Light Buff 2 triangles only on the outer part of the inner vane, which become irregular markings (Cinnamon 21 in colour) towards the distal portion of the feather. Tertiaries shaft like primaries (Vandyke Brown 282). Outer and inner vane of tertiaries similar in colour and similar to the outer vane of the secondaries. Under-tertiaries have Pale Buff 1 shaft proximally becoming Cinnamon 21 and later Sepia 279 towards the distal portion. Outer and inner vanes of under-tertiaries are similar: Cinnamon 21 with irregular Sepia 279 lines in the proximal portion, becoming irregular dots towards the distal section; terminal 1/5 with a Sepia 279 middle stripe along shaft. Primary coverts with Vandyke Brown 282 shafts. Outer vane of primary coverts with Sepia 279 with Cinnamon 21 markings becoming more packed towards the distal portion of the feather. Inner vane of primary coverts is similar to outer but with less packed Cinnamon 21 markings. Secondary coverts are overall Sepia 279 with Cinnamon 21 markings. Shaft is Vandyke Brown 282. Outer vane of secondary coverts with a Pale Buff 1 blotch delimited proximally by a Sepia 279 thin and sharp line. This blotch can have in its inner parts a Cinnamon 21 blotch delimited by a Sepia 279 thin and sharp line. Additional Sepia 279 lines distributed heterogeneously can be found on the outer vane. Inner vane of secondary coverts Sepia 279 with Light Buff 2 markings in proximal section and Cinnamon 21 markings in the distal portion of the feather. Lesser coverts with shafts Pale Buff 1 proximally and Vandyke Brown 282 in distal portion. Vandyke Brown 282 middle stripe along shaft. Outer vane of lesser co­verts Cinnamon 21 with Sepia 279 markings and Pale Pinkish Buff 3 markings delimited irregularly by Sepia 279 dashed lines. Inner vane of lesser coverts Sepia 279 with Cinnamon 21 markings. Coverts in the under-wings with Pale Buff 1 shaft. Outer vane on the coverts from the under-wings is Pale Buff 1 and Light Buff 2 with one Sepia 279 leopard spot and some additional (but rare) Sepia 279 markings. Inner vane of the coverts from the under-wings Pale Buff 1 and Light Buff 2 with Light Neutral Grey 297 colouration that become Sepia 279 distally. Alula shaft is Vandyke Brown 282. Outer vane of alula with five Verona Brown 37 ‘leopard’ spots delimited by Sepia 279 lines which is sharper in distal portion. Leopard spots separated by Pale Pinkish Buff 3 with Cinnamon 21 shadings. Inner vane of alula Sepia 279 with four Light Buff 2 partial bands.

***Tail***: Verona Brown 37 with Sepia 286 markings that fades towards the distal portion of the feather. Shaft Sepia 286. Outer feathers of the tail have an outer vane Verona Brown 37 with broad Sepia 286 bands, and an inner vane with broad poorly defined Sepia 286 bands intercalated by Light Buff 2, Pale Pinkish Buff 3 and more distally Verona Brown 37 bands.

***Bill***: Dusky Brown 285 and lower bill Light Buff 2.

***Iris***: Yellow.

***Vocalisations***: Call recordings collected at the moment of specimen collection included the call of the holotype and a second individual (XC audios: XC619445, XC619447): one emitted the main call type (the single repetitive note used in the bioacoustic analyses), and the other the cat-like call. We believe that the holotype individual was the one giving the main call, but this was uncertain. Thus, it is not possible to provide bioacoustics parameters specific to the holotype.

***Variation***: Morphometric variation in *O.bikegila* sp. nov. is based on the analysis of three additional individuals, of which one is a male (Table 1; Fig. 2). The male (P9–038) had shorter tarsus and wing length than the female holotype and the other two females. This result is consistent with the reversed sexual dimorphism in size described for all species of scops-owls (Marks et al. 1999; König et al. 2008). Two colour morphs (rufous and grey-brown) have been documented in the field (Figs 2, 9). Molecular sexing of the four captured individuals has shown that colour morph is not associated with sex. Examples of the grey-brown morph include the holotype (Figs 2A, 8), individuals P9-037 and P9-038 (Fig. 2B), and individuals photographed in the field (Fig. 2D, E); examples of the rufous morph include the first photographed individual of this species (Fig. 2C) and individual P8-001 (Fig. 2F). Plumage pattern and colour of the latter is similar to the holotype, although in the rufous morph the eyebrows are less marked, the underparts are more similar in colour to the upperparts and have more prominent sepia marks and stripes along the feather shafts. In the field, we observed no differences in the rate of occurrence of the two morphs.

Vocalisations were recorded at the type locality by MM in 2002, 2007, 2011, 2018 and 2019 and at Boca do Inferno in 2019, and by PV at the type locality in 2016. The call of *O.bikegila* was described in Melo and Dallimer (2009). Among vocalisations of *Otus* species, the primary call of *O.bikegila* sp. nov. is unique in consisting in a short, undulated note emitted at a fast repetition rate, reminiscent of insect calls, of ca. one note per second (Tables 2, 3; Fig. 4; Suppl. material 4: Fig. S2A). Vocalisations were often performed in duet (Suppl. material 4: Fig. S2B), with intercalated or overlapping notes. *Otusbikegila* sp. nov. is able to produce a cat-like “kee-a-u” note, which is emitted both in duets (Suppl. material 4: Fig. S2D) and by single birds (Suppl. material 4: Fig. S2C). We confirmed in the field that the same individual can produce both calls. Bioacoustic parameters (mean ± standard deviation) of the primary and of the cat-like notes are available in Tables 2, 3.

##### Etymology.

The species name is a patronym honouring Ceciliano do Bom Jesus, known as ‘Bikegila’ (Suppl. material 5). The species epithet name is intentionally defined as an invariable noun in apposition (not a noun in the genitive case) for better pronunciation; no confusion with the species authority is possible because the noun is an oral nickname.

Bikegila, a native of Príncipe Island, began the ‘Príncipe Scops-Owl saga’ in 1998, when he shared with MM reports of two sightings of birds that looked like owls in parrot nests. Since then, Bikegila took part in every field effort that led to the bird’s discovery for science; he also led the capture of all sampled individuals, including the holotype, which required ingenious ways to erect canopy nets. For almost 25 years, Bikegila has put all his resources, including bottomless fieldwork skills and a vast knowledge of Príncipe, towards the successful completion of innumerable research projects in a terrain that the collector José Correia considered to be the “*bad among the bad or the worse among the worse*” [sic] (Diary, 2 September 1928, Archives AMNH, New York). Besides his skills, Bikegila’s “*cheerful temperament, possibly the first requirement for an undertaking in inhospitable regions*” (von Humboldt 1841), coupled with an unbeatable gift for story-telling and an underlying quiet wisdom, contributes as much to making the expeditions he leads memorable and successful. A former parrot harvester, Bikegila became a warden of Prín­cipe Obô Natural Park soon after its creation; he is now a much sought-after nature guide.

We believe that most field researchers are grateful to the ‘Bikegilas’ with whom they are/were honoured to work with. As such, the name is also in recognition of all the people, around the world, who through their deep relationship with and knowledge of the regions they inhabit, play key roles in the description of new species and of new sites to science.

##### Common name.

We propose the English common name Principe Scops-Owl, the name for São Tomé and Príncipe as Kitóli-do-príncipe, and the name for the Portuguese list of the birds of the world as Mocho-do-príncipe. All common names refer to Príncipe Island, from where it is endemic.

##### Distribution and natural history.

**Figure 10.**
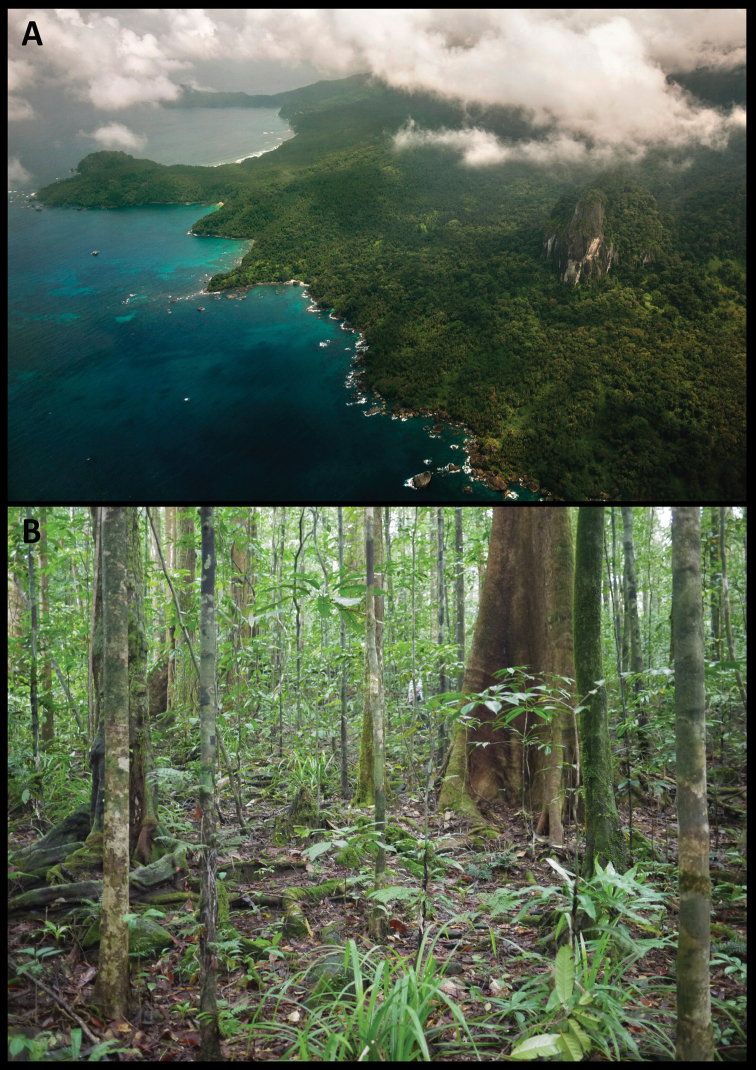
**A** aerial view of the south of Príncipe Island home of *Otusbikegila* sp. nov., and **B** habitat of the type locality at ca. 150 m a.s.l. Photographs: **A** Alexandre Vaz **B** MM.

All records from *O.bikegila* sp. nov. come from old-growth native lowland rainforest with mid-height (14–20 m) trees (Fig. 10), with the species apparently preferring lower elevations (Melo and Dallimer 2009; Freitas et al. 2022). Its area of occurrence is fully within the limits of Príncipe Obô Natural Park. Detailed surveys have been carried out to determine the area of occupancy of this species, to estimate its population size, ecological requirements, and to propose an IUCN Red List category (Freitas et al. 2022).

The holotype (Figs 2A, 8; female MHNC-UP-AVE7000), collected on 29 May 2017, was undergoing a well-advanced moult, a process that takes place after the breeding season. The female captured close to Ribeira Porco, in January 2019 (P8-001) had a fully developed brood patch (Fig. 2F), whereas the female captured at Boca do Inferno on the same month (Fig. 2B, left) was growing back the belly feathers, suggesting that she had a recent brood patch. This indicates that breeding takes place in December-January, as with most bird species of the islands of São Tomé and Príncipe (Jones and Tye 2006; Madeira 2018).

*Otusbikegila* sp. nov. starts calling at dusk and continues throughout the night. Contrarily to the Sao Tome Scops-Owl *O.hartlaubi* that regularly vocalises during the day, *O.bikegila* sp. nov. seems to require darkness to sing, although on a single occasion one individual was heard during the day (Melo and Dallimer 2009). Response to the playback of its call was fast and intense at all times of the year we were able to test it, with birds of either sex approaching the speaker. This indicates that *O.bikegila* sp. nov. is territorial all-year round as it is known from most sedentary cavity-nesting owls (Marks et al. 1999; König et al. 2008). During the day it may roost outside of tree cavities, as suggested when we accidentally flushed one bird when taking habitat measurements. In this situation the bird raised its ear tufts, which are otherwise seldom observed (Fig. 2D).

## ﻿Discussion

### ﻿*Otusbikegila*: a new bird species, endemic to Príncipe Island

Multiple lines of evidence were brought together to demonstrate, unambiguously, that the recently discovered population of scops-owls on Príncipe Island makes a well-differentiated species, *Otusbikegila*. Genetic distances, and associated divergence times, to its closest relatives were in the range of those separating currently accepted species (Table 5). Morphological differences, although present, did not stand out (for the human eye at least), whereas vocalisations were unique and clearly distinctive (even for the human ear) and, in fact, it was bioacoustics that led to the discovery of the population of the Principe Scops-Owl. Its unique vocalisations were closest to those of *O.ireneae*, an *Otus* species from which it is distantly related, underscoring the value of song in scops-owls to assess taxonomic status but not for inferring taxonomic affinities (Fuchs et al. 2007). Phylogenetic data placed *O.bikegila* as the sister lineage of the clade containing all African scops-owl species of the Afro-Palearctic clade (sensu Pons et al. 2013 and Fig. 6), rather than as the sister species of *O.hartlaubi* endemic to the neighbouring island of São Tomé. This leads to the curious conclusion that Príncipe was likely the first island in the Gulf of Guinea to be colonised by a species of scops-owl, albeit the last species to be discovered and described for science. It also begs the question if an undescribed scops-owl waits to be discovered in the extensive rainforests of Bioko Island, the only island of the Gulf of Guinea without records of a scops-owl. This apparent absence is puzzling as Bioko is a land-bridge island, which has been connected to the mainland in multiple instances in the past (Rohling et al. 1998; Lambert and Chappel 2001), and currently lying at ca. 30 km from Cameroon where *O.icterorhynchus*, a rainforest specialist, is present.

Although it may seem odd for a bird species to remain undiscovered for science for so long on such a small island, this is by no means an isolated case when it comes to owls. For example, the recently described Rinjani Scops-Owl *O.jolandae* Sangster, King, Verbelen & Trainor, 2013 was found to be a previously undescribed species from Lombok Island, Indonesia (Sangster et al. 2013). Similarly, the Anjouan Scops-Owl *O.capnodes* (Gurney, JH, 1889) was rediscovered in 1992 (Safford 1993), 106 years after its last observation, in an area of primary forest that is smaller and more regularly visited than that of Príncipe, and the Flores Scops-Owl *O.alfredi* (Hartert, E, 1897), rediscovered in 1994, 98 years after the previous report (Widodo et al. 1999).

### ﻿Novel insights in the phylogenetics of the genus *Otus*

Our phylogenetic analyses confirmed the supported nodes from previous phylogenies (Fuchs et al. 2008; Pons et al. 2013), resolved previously unsupported nodes, and provided novel insights in the affinities of species not previously included.

In relation to African taxa, the most interesting result came from the inclusion of samples from the two subspecies of the only African *Otus* species never sequenced before: the Sandy Scops-Owl *O.icterorhynchus*. Together with *O.ireneae*, this is the only species on the African continent that is a lowland forest specialist (albeit each species occupies very distinct forest types), and the two species were widely hypothesised as being closely related (Marks et al. 1999; König et al. 2008; Holt et al. 2020). Perhaps more surprisingly, *O.icterorhynchus* has been considered to form a superspecies (‘yellow-billed scops-owls’) with two Asian taxa, the Andaman Scops-Owl *O.balli* (Hume, 1873) and the Sumatran *Otusspilocephalusstresemanni* (Robinson, 1927) (Marshall 1978), although ‘*stresemanni*’ could be an anomalous form of another species and/or a hybrid (Pamela Rasmussen in Holt et al. 2020). Our phylogenetic analyses clarify the affinities of *O.icterorhynchus*, which was found to be sister to the clade containing the Afro-Palearctic and the Indo-Malayan/Indian Ocean clades. Each of its subspecies was available to us by a single individual, but their genetic divergence levels overlap with the levels found between many currently accepted scops-owls sister species pairs.

This study better resolved the branching sequence within the Afro-Palearctic clade, except for the position of *O.pamelae* that could not be determined, contra Pons et al. (2013) who recovered it as the sister lineage of this clade. Instead, *O.brucei*, with populations extending from the Arabian Peninsula into Asia, was recovered as the sister lineage of the clade which then branches into African and Eurasian subclades (assu­ming that *O.pamelae* is sister to *O.scops*).

The internodes separating *O.senegalensis* (mainland and Annobón Island), *O.hartlaubi* (São Tomé Island), and *O.pembaensis* (Pemba Island) are very short, indicating that the divergence between these three species (i.e., the colonisation of both islands from their mainland ancestor) occurred almost simultaneously, creating a hard polytomy. Our analyses failed to identify solid lines of evidence for the distinctiveness of *O.senegalensisfeae* from *O.senegalensissenegalensis*, although we did identify a diagnostic morphological character (bill length from tip to nares, Suppl. material 12) and three molecular diagnostic characters at the TGFB2 gene (C vs. T in site 285, T vs. G in site 344, C vs. G in site 392). In a dataset with a wider taxonomic sampling of *O.senegalensis* but fewer sequencing data, the Annobón sample nested within the mainland samples (*unpublished data*). A better sampling of *O.senegalensissenegalensis* from across its range will help to resolve this taxonomic issue.

Our sampling increased considerably the taxon coverage for the centre of the diversity of the *Otus* genus, the Indo-Malayan region (Marks et al. 1999), but has failed to solve most of the many taxonomic pending issues. The Ryukyu Scops-Owl *O.elegans* (Cassin, 1852), a species restricted to small oceanic islands from the northern Philippines to Japan, and included for the first time in a phylogenetic study, was recovered as the fourth Asian representative of the Indo-Malayan/Indian Ocean clade (Fig. 6; sensu Pons et al. 2013), albeit with no statistical support. At this stage, our results mainly highlight the need for using a phylogeographic-level sampling scheme across the region (i.e., multiple samples per site covering all areas of occurrence) to enable a thorough systematic revision of the Indo-Malayan taxa, a crucial step towards reconstructing the diversification history of the genus *Otus*.

Our molecular dataset confirmed the low levels of divergence (well within intraspecific variation) of three taxa pairs that are currently treated either as separate species or subspecies. These pairs are: i) *O.senegalensissenegalensis* (mainland Africa) and *O.s.feae* (Annobón Island), treated as separate species by del Hoyo (2020) and Gill et al. (2021) based on Collar and Boesman (2020); ii) *O.scops* and *O.cyprius*, treated as a distinct species by Gill et al. (2021) and Clements et al. 2021, based on Flint et al. (2015); and iii) the two species from Madagascar, the Malagasy Scops-Owl *O.rutilus* (Pucheran, 1849) and the Torotoroka Scops-Owl *O.madagascariensis* Grandidier, A, 1867, whose specific status was proposed by Rasmussen et al. (2000) and adopted by most authorities (e.g., Clements et al. 2021, del Hoyo 2020, Gill et al. 2021), but contested by Fuchs et al. (2007) using a representative geographic sampling.

## ﻿Conclusions

The discovery of a new bird species inhabiting the forests of Príncipe Island in 2016 (here formally described as *Otusbikegila*) underscores both the actuality of field-based explorations aiming at describing biodiversity (Dijkstra 2016), and how such curio­sity-driven endeavour is more likely to succeed when coupled with local ecological knowledge, the participation of keen amateur naturalists, and persistence.

## Supplementary Material

XML Treatment for Otus bikegila

## Supplementary materials

Supplementary material 1 Timeline Data type Text report. Explanation note Timeline of the discovery for science of the Principe Scops-Owl *Otusbikegila* sp. nov. https://binary.pensoft.net/file/762613This dataset is made available under the Open Database License (http://opendatacommons.org/licenses/odbl/1.0/). The Open Database License (ODbL) is a license agreement intended to allow users to freely share, modify, and use this Dataset while maintaining this same freedom for others, provided that the original source and author(s) are credited.Martim Melo, Bárbara Freitas, Philippe Verbelen, Sátiro R. da Costa, Hugo Pereira, Jérôme Fuchs, George Sangster, Marco N. Correia, Ricardo F. de Lima, Angelica Crottini

Supplementary material 2 Alignment Data type Docx file. Explanation note Concatenated sequences of the phylogenetic dataset. https://binary.pensoft.net/file/762614This dataset is made available under the Open Database License (http://opendatacommons.org/licenses/odbl/1.0/). The Open Database License (ODbL) is a license agreement intended to allow users to freely share, modify, and use this Dataset while maintaining this same freedom for others, provided that the original source and author(s) are credited.Martim Melo, Bárbara Freitas, Philippe Verbelen, Sátiro R. da Costa, Hugo Pereira, Jérôme Fuchs, George Sangster, Marco N. Correia, Ricardo F. de Lima, Angelica Crottini

Supplementary material 3 Figure S1. Topography Owls Data type Image (pdf file). Explanation note **Figure S1.** Topography of owls, with the terms used for the description and diagnosis of the Principe Scops-Owl *Otusbikegila* sp. nov. Illustration by MNC. https://binary.pensoft.net/file/762615This dataset is made available under the Open Database License (http://opendatacommons.org/licenses/odbl/1.0/). The Open Database License (ODbL) is a license agreement intended to allow users to freely share, modify, and use this Dataset while maintaining this same freedom for others, provided that the original source and author(s) are credited.Martim Melo, Bárbara Freitas, Philippe Verbelen, Sátiro R. da Costa, Hugo Pereira, Jérôme Fuchs, George Sangster, Marco N. Correia, Ricardo F. de Lima, Angelica Crottini

Supplementary material 4 Figure S2. Song oscillograms and spectrograms Data type Image (pdf file). Explanation note **Figure S2.** Oscillograms and spectrograms of 10s sections of the call of *O.bikegila* sp. nov.: (**A**) primary call of an individual, recorded on January 20, 2019, XC619448; (**B**) two different individuals duetting, recorded on July 28, 2018, XC619439; (**C**) cat-like “kee-a-u” call of one individual, recorded on January 15, 2019, XC619443; (**D**) two different individuals duetting, in which one emits a cat-like “kee-a-u” call, recorded on January 20, 2019, XC619448. Codes from Xeno-canto.org database. https://binary.pensoft.net/file/762616This dataset is made available under the Open Database License (http://opendatacommons.org/licenses/odbl/1.0/). The Open Database License (ODbL) is a license agreement intended to allow users to freely share, modify, and use this Dataset while maintaining this same freedom for others, provided that the original source and author(s) are credited.Martim Melo, Bárbara Freitas, Philippe Verbelen, Sátiro R. da Costa, Hugo Pereira, Jérôme Fuchs, George Sangster, Marco N. Correia, Ricardo F. de Lima, Angelica Crottini

Supplementary material 5 Figure S3. Bikegila Data type Image (pdf file). Explanation note **Figure S3.** Photograph taken at Boca do Inferno, Príncipe Island, January 27, 2019, showing the Principe Scops-Owl *Otusbikegila* sp. nov., the two first authors of the paper (BF on the left and MM on the right), and Ceciliano do Bom Jesus, known as ‘Bikegila’ (centre), who started the 20-year saga that led to this discovery, and in honour of whom the new species was named (see ‘Etymology’). https://binary.pensoft.net/file/762617This dataset is made available under the Open Database License (http://opendatacommons.org/licenses/odbl/1.0/). The Open Database License (ODbL) is a license agreement intended to allow users to freely share, modify, and use this Dataset while maintaining this same freedom for others, provided that the original source and author(s) are credited.Martim Melo, Bárbara Freitas, Philippe Verbelen, Sátiro R. da Costa, Hugo Pereira, Jérôme Fuchs, George Sangster, Marco N. Correia, Ricardo F. de Lima, Angelica Crottini

Supplementary material 6 Table S1. Description Data type Morphological. Explanation note **Table S1.** Plumage colour and pattern description of *O.bikegila* sp. nov., *O.hartlaubi*, *O.scopsscops*, *O.senegalensissenegalensis*, *O.senegalensisfeae* and *O.pembaensis*. Colour definition follow the colour standards of Köhler (2012). The analysed characters are depicted in the Suppl. material 3: Fig. S1. Abbreviations of institutional collections: BMNH - The Natural History Museum, Tring, UK; MHNC-UP - Museu de História Natural e da Ciência da Universidade do Porto, Portugal; SMD - Senckenberg Museum Dresden, Germany; SMF - Naturmuseum Senckenberg in Frankfurt am Main, Germany. https://binary.pensoft.net/file/762618This dataset is made available under the Open Database License (http://opendatacommons.org/licenses/odbl/1.0/). The Open Database License (ODbL) is a license agreement intended to allow users to freely share, modify, and use this Dataset while maintaining this same freedom for others, provided that the original source and author(s) are credited.Martim Melo, Bárbara Freitas, Philippe Verbelen, Sátiro R. da Costa, Hugo Pereira, Jérôme Fuchs, George Sangster, Marco N. Correia, Ricardo F. de Lima, Angelica Crottini

Supplementary material 7 Table S2. Details of song recordings Data type Database: song recordings Explanation note **Table S2.** Details of the recordings used for bioacoustic analyses. Recording codes - XC: Xeno-canto; IBC: The Internet Bird Collection (now under the Macaulay Library); AV: Avian Vocalizations Center (AVoCet). STP: São Tomé and Príncipe. https://binary.pensoft.net/file/762619This dataset is made available under the Open Database License (http://opendatacommons.org/licenses/odbl/1.0/). The Open Database License (ODbL) is a license agreement intended to allow users to freely share, modify, and use this Dataset while maintaining this same freedom for others, provided that the original source and author(s) are credited.Martim Melo, Bárbara Freitas, Philippe Verbelen, Sátiro R. da Costa, Hugo Pereira, Jérôme Fuchs, George Sangster, Marco N. Correia, Ricardo F. de Lima, Angelica Crottini

Supplementary material 8 Table S3. Song measurements Data type Statistics: Bioaucoustics. Explanation note **Table S3.** Measures taken for the songs in each recording used for bioacoustic analyses. Bioacoustic parameters - F1: frequency at start; F2: frequency at end; F3: frequency at 25% of total duration; F4: frequency at midpoint; F5: frequency at 75% of total duration; F6: frequency at maximum amplitude; F7: maximum frequency; F8: minimum frequency; DT1: total duration; DT2: time to maximum amplitude; DT3: time to maximum frequency; DT4: internote interval; DF1: frequency drop from start to end; DF2: frequency range; DFT1: slope from 25% to 75% of total duration; DFT2: slope from midpoint to end. https://binary.pensoft.net/file/762620This dataset is made available under the Open Database License (http://opendatacommons.org/licenses/odbl/1.0/). The Open Database License (ODbL) is a license agreement intended to allow users to freely share, modify, and use this Dataset while maintaining this same freedom for others, provided that the original source and author(s) are credited.Martim Melo, Bárbara Freitas, Philippe Verbelen, Sátiro R. da Costa, Hugo Pereira, Jérôme Fuchs, George Sangster, Marco N. Correia, Ricardo F. de Lima, Angelica Crottini

Supplementary material 9 Localities Data type excel file. Explanation note Coordinates of tissue sampling localities for *Otusbikegila* sp. nov. and *O.hartlaubi*. https://binary.pensoft.net/file/762621This dataset is made available under the Open Database License (http://opendatacommons.org/licenses/odbl/1.0/). The Open Database License (ODbL) is a license agreement intended to allow users to freely share, modify, and use this Dataset while maintaining this same freedom for others, provided that the original source and author(s) are credited.Martim Melo, Bárbara Freitas, Philippe Verbelen, Sátiro R. da Costa, Hugo Pereira, Jérôme Fuchs, George Sangster, Marco N. Correia, Ricardo F. de Lima, Angelica Crottini

Supplementary material 10 Table S4. Genomic regions, primers and amplification conditions Data type Database: Genomic regions and primers. Explanation note **Table S4.** Gene name, primer name, sequence, source, and amplification conditions used in the present study. PCR conditions start with temperature (in °C) of each step followed by the time in seconds. * primers used to amplify internal fragments (used for the amplification of DNA from toe-pad extractions). # custom-made primers used to amplify CYTB and ND2 fragments from *Otuscyprius*, *O.i.icterorhynchus*, *O.silvicola*, *O.spilocephalusvandewateri*, *O.s.vulpes*, *O.s.luciae*, and *O.s.hambroecki*. https://binary.pensoft.net/file/762622This dataset is made available under the Open Database License (http://opendatacommons.org/licenses/odbl/1.0/). The Open Database License (ODbL) is a license agreement intended to allow users to freely share, modify, and use this Dataset while maintaining this same freedom for others, provided that the original source and author(s) are credited.Martim Melo, Bárbara Freitas, Philippe Verbelen, Sátiro R. da Costa, Hugo Pereira, Jérôme Fuchs, George Sangster, Marco N. Correia, Ricardo F. de Lima, Angelica Crottini

Supplementary material 11 Table S5. PCA factor loadings of morphological variables Data type Statistics: Morphological. Explanation note **Table S5.** Factor loadings of morphological variables on the first two principal components for seven *Otus* taxa (*O.bikegila* sp. nov., *O.hartlaubi*, *O.senegalensissenegalensis*, *O.senegalensisfeae*, *O.pembaensis*, *O.scops*, *O.brucei*). Eigenvalues and percentage of variance explained by the respective components are given at the bottom of the table. Morphological measurements – Bilen: bill length from bill tip to where culmen enters feathers; Binares: bill length from the anterior end of the nares to the tip; Biwid: bill width; Tarlen: tarsus length; Wilen: wing length; Tailen: tail length. https://binary.pensoft.net/file/762623This dataset is made available under the Open Database License (http://opendatacommons.org/licenses/odbl/1.0/). The Open Database License (ODbL) is a license agreement intended to allow users to freely share, modify, and use this Dataset while maintaining this same freedom for others, provided that the original source and author(s) are credited.Martim Melo, Bárbara Freitas, Philippe Verbelen, Sátiro R. da Costa, Hugo Pereira, Jérôme Fuchs, George Sangster, Marco N. Correia, Ricardo F. de Lima, Angelica Crottini

Supplementary material 12 Table S6. Morphological differentiation Data type Statistics: Morphology. Explanation note **Table S6.** Results of Welch’s ANOVA and Games-Howell post-hoc comparisons performed with the morphometric variables of six *Otus* taxa (*O.bikegila* sp. nov., *O.hartlaubi*, *O.senegalensissenegalensis*, *O.senegalensisfeae*, *O.pembaensis*, *O.scops*). Superscript values indicate significance levels (* P < 0.05; ** P < 0.01; *** P < 0.005; **** P < 0.001) and NA (Not Available, significance level not possible to obtain due to low sample size). Some individuals had missing data, not allowing for the test performance (indicated by -). Morphological measurements – Bilen: bill length from bill tip to where culmen enters feathers; Biwid: bill width; Bidepth: bill depth at the anterior end of nares; Binares: bill length from the anterior end of the nares to the tip; Hebi: head+bill, from the tip of the bill to the opposite point on the back of the skull; Midt: middle toe length; Tarlen: tarsus length; Wilen: wing length; Tailen: tail length; Bolen: body length; SP: shortfall in relation to tip of longest primary (P1 is the closest to the body), SP10: shortfall of P10; SP9: shortfall of P9; SP5: shortfall of P5; SP4: shortfall of P4. https://binary.pensoft.net/file/762624This dataset is made available under the Open Database License (http://opendatacommons.org/licenses/odbl/1.0/). The Open Database License (ODbL) is a license agreement intended to allow users to freely share, modify, and use this Dataset while maintaining this same freedom for others, provided that the original source and author(s) are credited.Martim Melo, Bárbara Freitas, Philippe Verbelen, Sátiro R. da Costa, Hugo Pereira, Jérôme Fuchs, George Sangster, Marco N. Correia, Ricardo F. de Lima, Angelica Crottini

Supplementary material 13 Table S7. PCA factor loadings of bioacoustic variables Data type Statistics: Bioaucoustics. Explanation note **Table S7.** Factor loadings of bioacoustic variables on the four principal components in 10 *Otus* taxa (*O.bikegila* sp. nov., *O.hartlaubi*, *O.senegalensissenegalensis*, *O.senegalensisfeae*, *O.pembaensis*, *O.pamelae*, *O.scops*, *O.cyprius*, *O.brucei*, *O.ireneae*). Eigenvalues and percentage of variance explained by the respective components are given at the bottom of the table. Bioacoustic parameters - F1: frequency at start; F2: frequency at end; F3: frequency at 25% of total duration; F4: frequency at midpoint; F5: frequency at 75% of total duration; F6: frequency at maximum amplitude; F7: maximum frequency; F8: minimum frequency; DT1: total duration; DT2: time to maximum amplitude; DT3: time to maximum frequency; DT4: internote interval; DF1: frequency drop from start to end; DF2: frequency range; DFT1: slope from 25% to 75% of total duration; DFT2: slope from midpoint to end. https://binary.pensoft.net/file/762625This dataset is made available under the Open Database License (http://opendatacommons.org/licenses/odbl/1.0/). The Open Database License (ODbL) is a license agreement intended to allow users to freely share, modify, and use this Dataset while maintaining this same freedom for others, provided that the original source and author(s) are credited.Martim Melo, Bárbara Freitas, Philippe Verbelen, Sátiro R. da Costa, Hugo Pereira, Jérôme Fuchs, George Sangster, Marco N. Correia, Ricardo F. de Lima, Angelica Crottini

Supplementary material 14 Table S8. Bioacoustics differentiation Data type Statistics: Bioacoustics. Explanation note **Table S8.** Results of Welch’s ANOVA and Games-Howell post-hoc comparisons performed with the bioacoustic variables of 10 *Otus* taxa (*O.bikegila* sp. nov., *O.hartlaubi*, *O.senegalensissenegalensis*, *O.senegalensisfeae*, *O.pembaensis*, *O.pamelae*, *O.scops*, *O.cyprius*, *O.brucei*, *O.ireneae*). Superscript values indicate significance levels that are indicated with asterisks (* P < 0.05; ** P < 0.01; *** P < 0.005; **** P < 0.001) and NA (Not Available, significance level not possible to obtain due to low sample size). Bioacoustic parameters - F1: frequency at start; F2: frequency at end; F3: frequency at 25% of total duration; F4: frequency at midpoint; F5: frequency at 75% of total duration; F6: frequency at maximum amplitude; F7: maximum frequency; F8: minimum frequency; DT1: total duration; DT2: time to maximum amplitude; DT3: time to maximum frequency; DT4: internote interval; DF1: frequency drop from start to end; DF2: frequency range; DFT1: slope from 25% to 75% of total duration; DFT2: slope from midpoint to end. https://binary.pensoft.net/file/762626This dataset is made available under the Open Database License (http://opendatacommons.org/licenses/odbl/1.0/). The Open Database License (ODbL) is a license agreement intended to allow users to freely share, modify, and use this Dataset while maintaining this same freedom for others, provided that the original source and author(s) are credited.Martim Melo, Bárbara Freitas, Philippe Verbelen, Sátiro R. da Costa, Hugo Pereira, Jérôme Fuchs, George Sangster, Marco N. Correia, Ricardo F. de Lima, Angelica Crottini

Supplementary material 15 Table S9. Phylogeny: sequence partition and evolution models Data type Analysis: Partitioning scheme and selected models of evolution. Explanation note **Table S9.** Best-fitting partitioning scheme, and respective best model of sequence evolution, inferred with PartitionFinder2 for the dataset used to infer the phylogenetic affinities within the genus *Otus* (Suppl. material 2: Dataset 2: 12,925 bp). https://binary.pensoft.net/file/762627This dataset is made available under the Open Database License (http://opendatacommons.org/licenses/odbl/1.0/). The Open Database License (ODbL) is a license agreement intended to allow users to freely share, modify, and use this Dataset while maintaining this same freedom for others, provided that the original source and author(s) are credited.Martim Melo, Bárbara Freitas, Philippe Verbelen, Sátiro R. da Costa, Hugo Pereira, Jérôme Fuchs, George Sangster, Marco N. Correia, Ricardo F. de Lima, Angelica Crottini

Supplementary material 16 Table S10. Divergence times: sequence evolution models Data type Analysis: Selected models of evolution for divergence times. Explanation note **Table S10.** Best models of sequence evolution, inferred with MEGA X, for the markers used in divergence time analyses of the genus *Otus*. https://binary.pensoft.net/file/762628This dataset is made available under the Open Database License (http://opendatacommons.org/licenses/odbl/1.0/). The Open Database License (ODbL) is a license agreement intended to allow users to freely share, modify, and use this Dataset while maintaining this same freedom for others, provided that the original source and author(s) are credited.Martim Melo, Bárbara Freitas, Philippe Verbelen, Sátiro R. da Costa, Hugo Pereira, Jérôme Fuchs, George Sangster, Marco N. Correia, Ricardo F. de Lima, Angelica Crottini

## References

[B1] Atkinson P Peet N Alexander J (1991) The status and conservation of the endemic bird species of São Tomé and Príncipe, West Africa. Bird Conservation International 1(3): 255–282. 10.1017/S0959270900000629

[B2] Avise JC Ball Jr RM (1990) Principles of genealogical concordance in species concepts and biological taxonomy. In: Futuyma D Antonovics J (Eds) Oxford Surveys in Evolutionary Biology, vol.7. Oxford University Press, Oxford, 45–67.

[B3] Avise J Wollenberg K (1997) Phylogenetics and the origin of species. Proceedings of the National Academy of Sciences of the United States of America 94(15): 7748–7755. 10.1073/pnas.94.15.7748 9223259 PMC33696

[B4] Baker P Davis S Payne S Revill M (2003) On preparing animal skeletons: a simple and effective method. International Council for Archaeozoology 4: 4–15. http://www.alexandriaarchive.org/icaz/icaz_website_formembers/pdf/nspring03.pdf

[B5] Bruford MW Hanotte O Brookfield JFY Burke T (1992) Single-locus and multilocus DNA fingerprinting. In: Hoelzel AR (Ed.) Molecular genetic analysis of populations: a practical approach.IRL Press, Oxford, New York, 225–269.

[B6] Buchanan GM Donald PF Butchart SHM (2011) Identifying priority areas for conservation: A global assessment for forest-dependent birds. PLoS ONE 6(12): e29080. 10.1371/journal.pone.0029080 PMC3242781 22205998

[B7] Burke K (2001) Origin of the Cameroon line of volcanic-capped swells. The Journal of Geology 109(3): 349–362. 10.1086/319977

[B8] Cadena CD Zapata F (2021) The genomic revolution and species delimitation in birds (and other organisms): Why phenotypes should not be overlooked. Ornithology 138(2): 1–18. 10.1093/ornithology/ukaa069

[B9] Castanheira-Diniz A Cardoso-de-Matos G (2002) Carta de Zonagem Agro-Ecológica e da Vegetação de S. Tomé e Príncipe: 2 - Ilha do Príncipe. Garcia De Orta - Série de Botânica 15: 47–72.

[B10] Cataldo D (2017) Phenotypic differentiation in the Azorean woodpigeon (*Columbapalumbusazorica*). M.Sc. dissertation, University of Porto, Porto, Portugal. https://hdl.handle.net/10216/110672

[B11] Ceballos G Ehrlich PR Raven PH (2020) Vertebrates on the brink as indicators of biological annihilation and the sixth mass extinction. Proceedings of the National Academy of Sciences of the United States of America 117(24): 13596–13602. 10.1073/pnas.1922686117 32482862 PMC7306750

[B12] Clement M Posada D Crandall KA (2000) TCS: A computer program to estimate gene genealogies. Molecular Ecology 9(10): 1657–1659. 10.1046/j.1365-294x.2000.01020.x 11050560

[B13] Clements JF Schulenberg TS Iliff MJ Billerman SM Fredericks TA Gerbracht JA Lepage D Sullivan BL Wood CL (2021) The eBird/Clements checklist of Birds of the World: v. 2021. https://www.birds.cornell.edu/clementschecklist/download/ [accessed 10 November 2021]

[B14] Collar NJ Boesman P (2020) The taxonomic status of Annobón Scops Owl *Otusfeae* and Arabian Scops Owl *O.pamelae*. Bulletin of the African Bird Club 27: 159–167.

[B15] Dabney J Knapp M Glocke I Gansauge M-T Weihmann A Nickel B Valdiosera C Garcia N Pääbo S Arsuaga J-L Meyer M (2013) Complete mitochondrial genome sequence of a Middle Pleistocene cave bear reconstructed from ultrashort DNA fragments. Proceedings of the National Academy of Sciences of the United States of America 110(39): 15758–15763. 10.1073/pnas.1314445110 24019490 PMC3785785

[B16] Davis S Payne S (1992) 101 ways to deal with a dead hedgehog: notes on the preparation of disarticulated skeletons for zoo-archaeological use. Circaea 8: 95–104.

[B17] del Hoyo J [Ed.] (2020) All the Birds of the World. Lynx Edicións, Barcelona, 968 pp.

[B18] Dijkstra K-DB (2016) Natural history: Restore our sense of species. Nature 533(7602): 172–174. 10.1038/533172a 27172032

[B19] Drummond AJ Rambaut A (2007) BEAST: Bayesian evolutionary analysis by sampling trees. BMC Evolutionary Biology 7(1): e214. 10.1186/1471-2148-7-214 PMC2247476 17996036

[B20] Drummond AJ Suchard MA Xie D Rambaut A (2012) Bayesian Phylogenetics with BEAUti and the BEAST 1.7. Molecular Biology and Evolution 29(8): 1969–1973. 10.1093/molbev/mss075 22367748 PMC3408070

[B21] Exell AW (1944) Catalogue of the Vascular Plants of S. Tomé (with Príncipe and Annobón). British Museum (Natural History), London, [xi +] 428 pp.

[B22] Flint P Whaley D Kirwan GM Charalambides M Schweizer M Wink M (2015) Reprising the taxonomy of Cyprus Scops Owl Otus (scops) cyprius, a neglected island endemic. Zootaxa 4040(3): 301–316. 10.11646/zootaxa.4040.3.3 26624667

[B23] Freitas B Melo M Bom Jesus C Costa SR Santos Y Crottini A Lima RF (2022) The recently discovered Principe Scops-owl is highly threatened: distribution, habitat associations, and population estimates. Bird Conservation International. 10.1017/S0959270922000429

[B24] Fuchs J Pons J-M Pasquet E Raherilalao MJ Goodman SM (2007) Geographical structure of genetic variation in the Malagasy Scops-Owl inferred from mitochondrial sequence data. The Condor 109(2): 408–418. 10.1093/condor/109.2.408

[B25] Fuchs J Pons J-M Goodman SM Bretagnolle V Melo M Bowie RC Currie D Safford R Virani MZ Thomsett S Hija A Cruaud C Pasquet E (2008) Tracing the colonization history of the Indian Ocean scops-owls (Strigiformes: *Otus*) with further insight into the spatio-temporal origin of the Malagasy avifauna. BMC Evolutionary Biology 8(1): e197. 10.1186/1471-2148-8-197 PMC2483963 18611281

[B26] Gahr M (2000) Neural song control system of hummingbirds: Comparison to swifts, vocal learning (songbirds) and nonlearning (suboscines) passerines, and vocal learning (Budgerigars) and nonlearning (dove, owl, gull, quail, chicken) nonpasserines. The Journal of Comparative Neurology 426(2): 182–196. 10.1002/1096-9861(20001016)426:2<182::AID-CNE2>3.0.CO;2-M 10982462

[B27] Gascoigne A (2004) São Tomé, Príncipe, and Annobón Moist Lowland Forests. In: Burgess N D’Amico Hales J Underwood E Dinerstein E Olson D Itoua I Schipper J Ricketts T Newman K (Eds) Terrestrial Ecoregions of Africa and Madagascar: a Conservation Assessment).Island Press, Washington, 236–238.

[B28] Gill F Donsker D Rasmussen P [Eds] (2021) IOC World Bird List (v. 11.1). 10.14344/IOC.ML.11.1 [accessed 10 November 2021]

[B29] Griffiths R Double MC Orr K Dawson RJG (1998) A DNA test to sex most birds. Molecular Ecology 7(8): 1071–1075. 10.1046/j.1365-294x.1998.00389.x 9711866

[B30] Hall TA (1999) BioEdit: A user-friendly biological sequence alignment editor and analysis program for Windows 95/98/NT. Nucleic Acids Symposium Series 41: 95–98. http://jwbrown.mbio.ncsu.edu/JWB/papers/1999Hall1.pdf

[B31] Holt DW Berkley R Deppe C Enríquez PL Petersen JL Rangel Salazar JL Segars KP Wood KL Marks JS (2020) Mountain Scops-Owl (*Otusspilocephalus*), version 1.0. In: del Hoyo J, Elliott A, Sargatal J, Christie DA, de Juana E (Eds) Birds of the World. Cornell Lab of Ornithology, Ithaca. 10.2173/bow.mosowl2.01 [accessed 01 June 2021]

[B32] Holyoak DT (2001) Nightjars and their Allies. Oxford University Press, Oxford, 848 pp.

[B33] Jenni L Winkler R (1989) The feather-length of small passerines: A measurement for wing-length in live birds and museum skins. Bird Study 36(1): 1–15. 10.1080/00063658909476996

[B34] Jones PJ (1994) Biodiversity in the Gulf of Guinea: An overview. Biodiversity and Conservation 3(9): 772–784. 10.1007/BF00129657

[B35] Jones PJ Tye A (2006) The Birds of São Tomé and Príncipe, with Annobón: Islands of the Gulf of Guinea. British Ornithologists’ Union, Oxford, 172 pp.

[B36] Katoh K Standley DM (2013) MAFFT Multiple sequence alignment software version 7: Improvements in performance and usability. Molecular Biology and Evolution 30(4): 772–780. 10.1093/molbev/mst010 23329690 PMC3603318

[B37] Köhler G (2012) Color Catalog for Field Biologists. Herpeton, Offenbach, 49 pp.

[B38] König C Weick F Becking J-H (2008) Owls of the World, 2^nd^ edn. Yale University Press, New Haven, 528 pp.

[B39] Kumar S Stecher G Tamura K (2016) MEGA7: Molecular Evolutionary Genetics Analysis version 7.0 for bigger datasets. Molecular Biology and Evolution 33(7): 1870–1874. 10.1093/molbev/msw054 27004904 PMC8210823

[B40] Kumar S Stecher G Li M Knyaz C Tamura K (2018) MEGA X: Molecular Evolutionary Genetics Analysis across computing platforms. Molecular Biology and Evolution 35(6): 1547–1549. 10.1093/molbev/msy096 29722887 PMC5967553

[B41] Lambert K Chappel J (2001) Sea level change through the last glacial cycle. Science 292(5517): 679–686. 10.1126/science.1059549 11326090

[B42] Lambert FR Rasmussen PC (1998) A new scops owl from Sangihe Island, Indonesia. Bulletin of the British Ornithologists’ Club 118: 204–217. https://biostor.org/reference/144176

[B43] Lane DF Aponte Justiniano MA Terrill RS Rheindt FE Klicka LB Rosenberg GH Schmitt CJ Burns KJ (2021) A new genus and species of tanager (Passeriformes, Thraupidae) from the lower Yungas of western Bolivia and southern Peru. Ornithology 138(4): ukab059. 10.1093/ornithology/ukab059

[B44] Lanfear R Calcott B Ho SYW Guindon S (2012) PartitionFinder: Combined selection of partitioning schemes and substitution models for phylogenetic analyses. Molecular Biology and Evolution 29(6): 1695–1701. 10.1093/molbev/mss020 22319168

[B45] Lanfear R Frandsen PB Wright AM Senfeld T Calcott B (2016) PartitionFinder 2: New methods for selecting partitioned models of evolution for molecular and morphological phylogenetic analyses. Molecular Biology and Evolution 34: 772–773. 10.1093/molbev/msw260 28013191

[B46] Lê S Josse J Husson F (2008) FactoMineR: An *R* package for multivariate analysis. Journal of Statistical Software 25(1): 1–18. 10.18637/jss.v025.i01

[B47] Lerner HRL Meyer M James HF Hofreiter M Fleischer RC (2011) Multilocus resolution of phylogeny and timescale in the extant adaptive radiation of Hawaiian honeycreepers. Current Biology 21(21): 1838–1844. 10.1016/j.cub.2011.09.039 22018543

[B48] Madeira B (2018) Sexual dimorphism and reproductive phenology of common birds in São Tomé Island – conservation implications. M.Sc. dissertation. University of Lisbon, Lisbon. http://hdl.handle.net/10451/33859

[B49] Marcot BG Johnson DH (2003) Owls in mythology and culture. In: Duncan JR (Ed.) Owls of the World: Their Lives, Behavior and Survival.Key Porter Books, Toronto, 88–105.

[B50] Marks JS Cannings RJ Mikkola H (1999) Family Strigidae (Typical Owls). In: del Hoyo J Elliott A Sargatal J (Eds) Handbook of the Birds of the World – Volume 5.Lynx Edicións, Barcelona, 76–243.

[B51] Marshall JT (1978) Systematics of smaller Asian night birds based on voice. Ornithological Monographs 25: 1–58. 10.2307/40166757

[B52] McDonald JH (2014) Handbook of Biological Statistics. 3^rd^ Edn. Sparky House Publishing, Baltimore, 299 pp. https://www.biostathandbook.com/HandbookBioStatThird.pdf

[B53] Melo M Dallimer M (2008) The status of a rare and recently described endemic bird species (the Príncipe Thrush *Turdus* [*olivaceofuscus*] *xanthorhynchus*) and a search for an as yet undescribed ‘Owl’. Davis Expedition Fund Report.

[B54] Melo M Dallimer M (2009) Is there an undiscovered endemic scops owl *Otus* sp. on Príncipe Island? Malimbus 31: 109–115. http://malimbus.free.fr/articles/V31/31109115.pdf

[B55] Melo M Jones P Lima RF (2022) The avifauna of the Gulf of Guinea Oceanic Islands. In: Ceríaco LMP Lima RF Melo M Bell RC (Eds) Biodiversity of the Gulf of Guinea Oceanic Islands: Science and Conservation.Springer, Cham, 555–592. 10.1007/978-3-031-06153-0_21

[B56] Milá B Bruxaux J Friis G Sam K Ashari H Thébaud C (2021) A new, undescribed species of *Melanocharis* berrypecker from western New Guinea and the evolutionary history of the family Melanocharitidae. The Ibis 163(4): 1310–1329. 10.1111/ibi.12981

[B57] Miller MA Pfeiffer W Schwartz T (2010) Creating the CIPRES Science Gateway for inference of large phylogenetic trees. 2010 Gateway Computing Environments Workshop (GCE). IEEE, New Orleans, 1–8. 10.1109/GCE.2010.5676129

[B58] Mora C Tittensor DP Adl S Simpson AGB Worm B (2011) How many species are there on Earth and in the Ocean? PLoS Biology 9(8): e1001127. 10.1371/journal.pbio.1001127 PMC3160336 21886479

[B59] Morony JJ Bock WJ Farrand J (1975) Reference list of the birds of the world. American Museum of Natural History, New York, [x +] 207 pp. https://digitallibrary.amnh.org/handle/2246/6700

[B60] Muñoz-Pajares AJ Belluardo F Cocca W Crottini A (2019) PipeLogeny: An automated pipeline for phylogenetic reconstruction. https://sites.google.com/site/pipelogenydownload/ [accessed on 15 February 2021]

[B61] Múrias dos Santos A Cabezas MP Tavares AI Xavier R Branco M (2016) tcsBU: A tool to extend TCS network layout and visualization. Bioinformatics 32(4): 627–628. 10.1093/bioinformatics/btv636 26515821

[B62] Padial JM Miralles A De la Riva I Vences M (2010) The integrative future of taxonomy. Frontiers in Zoology 7(1): e16. 10.1186/1742-9994-7-16 PMC2890416 20500846

[B63] Pons J-M Kirwan GM Porter RF Fuchs J (2013) A reappraisal of the systematic affinities of Socotran, Arabian and East African scops owls (*Otus*, Strigidae) using a combination of molecular, biometric and acoustic data. The Ibis 155(3): 518–533. 10.1111/ibi.12041

[B64] R Core Team (2017) R: A language and environment for statistical computing. R Foundation for Statistical Computing, Vienna. https://www.R-project.org/

[B65] Rambaut A Drummond AJ Xie D Baele G Suchard MA (2018) Posterior summarization in Bayesian phylogenetics using Tracer 1.7. Systematic Biology 67(5): 901–904. 10.1093/sysbio/syy032 29718447 PMC6101584

[B66] Rasmussen PC Schulenberg TS Hawkins AFA Voninavoko R (2000) Geographical variation in the Malagasy Scops-Owl (*Otusrutilus* auct.): The existence of an unrecognized species on Madagascar and the taxonomy of other Indian Ocean taxa. Bulletin of the British Ornithologists’ Club 120: 75–102. http://biostor.org/reference/111890

[B67] Rheindt FE Prawiradilaga DM Ashari H Suparno Gwee CY Lee GWX Wu MY Ng NSR (2020) A lost world in Wallacea: Description of a montane archipelagic avifauna. Science 367(6474): 167–170. 10.1126/science.aax2146 31919216

[B68] Rohling EJ Fenton M Jorissen FJ Bertrand P Ganssen G Caulet JP (1998) Magnitudes of sea-level lowstands of the past 500,000 years. Nature 394(6689): 162–165. 10.1038/28134

[B69] Ronquist F Teslenko M van der Mark P Ayres DL Darling A Höhna S Larget B Liu L Suchard MA Huelsenbeck JP (2012) MrBayes 3.2: Efficient Bayesian phylogenetic inference and model choice across a large model space. Systematic Biology 61(3): 539–542. 10.1093/sysbio/sys029 22357727 PMC3329765

[B70] Rozas J Ferrer-Mata A Sánchez-DelBarrio JC Guirao-Rico S Librado P Ramos-Onsins SE Sánchez-Gracia A (2017) DnaSP 6: DNA sequence polymorphism analysis of large data sets. Molecular Biology and Evolution 34(12): 3299–3302. 10.1093/molbev/msx248 29029172

[B71] RStudio Team (2015) RStudio: Integrated development environment for R. RStudio, Inc., Boston. https://www.rstudio.com/products/rstudio/

[B72] Ryan P (2016) New scops owl on Príncipe. African Birdlife 5: 10. http://www.fitzpatrick.uct.ac.za/sites/default/files/image_tool/images/275/Publications/semi-popular/2016/AB05%2801%2910.pdf

[B73] Safford RJ (1993) Rediscovery, taxonomy and conservation of the Anjouan Scops Owl *Otuscapnodes* (Gurney 1889). Bird Conservation International 3(1): 57–74. 10.1017/S0959270900000782

[B74] Salter JF Oliveros CH Hosner PA Manthey JD Robbins MB Moyle RG Brumfield RT Faircloth BC (2020) Extensive paraphyly in the typical owl family (Strigidae). The Auk 137(1): ukz070. 10.1093/auk/ukz070

[B75] Sangster G (2018) Integrative taxonomy of birds: the nature and delimitation of species. In: Tietze DT (Ed.) Bird species: how they arise, modify, and vanish.Springer International Publishing, Cham, 9–37. 10.1007/978-3-319-91689-7_2

[B76] Sangster G King BF Verbelen P Trainor CR (2013) A new owl species of the genus *Otus* (Aves: Strigidae) from Lombok, Indonesia. PLoS ONE 8(2): e53712. 10.1371/journal.pone.0053712 PMC3572129 23418422

[B77] Stephens M Smith NJ Donnelly P (2001) A new statistical method for haplotype reconstruction from population data. American Journal of Human Genetics 68(4): 978–989. 10.1086/319501 11254454 PMC1275651

[B78] Templeton AR Crandall KA Sing CF (1992) A cladistic analysis of phenotypic associations with haplotypes inferred from restriction endonuclease mapping and DNA sequence data. III. Cladogram estimation. Genetics 132(2): 619–633. 10.1093/genetics/132.2.619 1385266 PMC1205162

[B79] Verbelen P Melo M Sangster G Spina F (2016) A 90-year-old mystery solved: A potentially new species of owl from Príncipe. Oryx 50(4): 581. 10.1017/S0030605316000831

[B80] von Humboldt A (1841) Preface to: Reisen in Guiana und am Orinoco in den Jahren 1835–1839 (Schomburgk, R. H., author). Georg Wigand, Leipzig, [xxiv +] 510 pp.

[B81] Warakagoda DH Rasmussen PC (2004) A new species of scops-owl from Sri Lanka. Bulletin of the British Ornithologists’ Club 124: 85–105. https://biostor.org/reference/106330

[B82] Widodo W Cox JH Rasmussen PC (1999) Rediscovery of the Flores Scops Owl *Otusalfredi* on Flores, Lesser Sunda Islands, Indonesia, and reaffirmation of its specific status. Forktail 15: 15–23. https://orientalbirdclub.org/wp-content/uploads/2012/09/Widodo-Flores.pdf

[B83] Winkler DW Billerman SM Lovette IJ (2020) Owls (*Strigidae*), version 1.0. In: Billerman SM, Keeney BK, Rodewald PG, Schulenberg TS (Eds) Birds of the World. Cornell Lab of Ornithology, Ithaca. 10.2173/bow.strigi1.01 [accessed 01 June 2021]

