# Supplementary material for: ﻿A new species of scops-owl (Aves, Strigiformes, Strigidae, Otus) from Príncipe Island (Gulf of Guinea, Africa) and novel insights into the systematic affinities within Otus
Source: Zookeys. 2022 Oct 30;1126:1–54. doi: 10.3897/zookeys.1126.87635 (PMC9836643; doi:10.3897/zookeys.1126.87635)
Supplement: Supplementary material 1 — Timeline [file zookeys-1126-001_article-87635__-s001.docx]

**Timeline of the discovery for science of the Principe Scops-Owl *Otus bikegila* sp. nov.**

In 1998, while studying the Grey Parrot *Psittacus erithacus princeps* Alexander, 1909 from Príncipe, Martim Melo collected testimonials from Ceciliano do Bom Jesus (known as Bikegila) describing two occasions, in the early 1990s, when parrot harvesters had found a bird unknown to them in the tree holes where they were looking for parrot chicks. Both these events took place in the southern forests of the island (Ribeira Porco and Focinho de Cão), the main parrot breeding site (Melo 1998). In one instance, Bikegila himself was present and the description of the bird fitted the overall appearance of a small owl. Sátiro da Costa was responsible for the second observation, and when presented with the color plates of the endemic birds of the Gulf of Guinea islands (Borrow and Demey 2001) immediately identified it as the Sao Tome Scops-Owl *Otus hartlaubi* (Giebel, 1872). He also reported that the elders told him it was a “Kitóli” (the name given on São Tomé Island to *O. hartlaubi*), and that it destroys parrot eggs to occupy the nesting cavity, suggesting that there was some local knowledge on the species (Melo 1998). In 2002, Martim Melo heard and recorded unknown calls in the area of Ribeira Porco. Notes of these calls were in the same frequency range as those of other scops-owls, but were distinct from those of any known species (Melo and Dallimer 2008, 2009), and therefore could qualify the putative population of Scops-Owl from Príncipe as a confirmed candidate species (sensu Vieites et al. 2009). In 2007-2008, a survey of the island was carried out to determine the distribution and abundance of the Principe Thrush *Turdus xanthorhynchus* Salvadori, 1901 (Dallimer et al. 2010), and to determine the source of the putative owl-like calls (Melo and Dallimer 2008, 2009). These calls were mapped and new recordings obtained, but the individuals emitting these calls remained undetected.

In 2009, in an effort to unveil independent evidence for the presence of an owl on Príncipe, Rita Covas (CIBIO-InBIO, Portugal), at the request of Martim Melo, unearthed a letter by José Correia in the archives of the American Museum of Natural History (AMNH), New York. In 1928-1929, Correia and his wife Virginia collected in the Gulf of Guinea for the AMNH. Their collection was the basis for the first synthesis on the origin of the birds of the islands (Amadon 1953). In a letter from Príncipe Island, dated 3 Oct 1928, he reports to Gordon Cushman Murphy that although he did not see any owl on Príncipe, some residents told him about its presence in the “wild forests”, where it could take them up to ten years before they could find one (Correia 1928). This information had no impact whatsoever, and the letter was archived with no efforts to pursue this lead. Similarly, we found out that the ornithologist René de Naurois, who did extensive work on the islands in the 1970s, never saw an owl on Príncipe, but did collect four third-party potential sightings, all from the northern part of the island (Naurois 1975, 1983). The first two were from airport workers, who described seeing on occasion a “coruja”, the Portuguese name used for medium-sized owls, including the Barn Owl *Tyto alba thomensis* (Hartlaub, 1852) resident on São Tomé. In the third instance, another airport employee brought Naurois a “coruja”, which turned out to be a vagrant Common Kestrel *Falco tinnunculus* Linnaeus, 1758. The last sighting was by the Príncipe administrator who reported on seeing a bird with a similar shape to the Eurasian Scops-Owl *O. scops* (Linnaeus, 1758) on the ground of the airport runway – a typical behavior of nightjars (Caprimulgidae) but unheard off in scops-owls. Pennant-winged Nightjars *Macrodipteryx vexillaria* (Gould, 1838) are recorded as probable migrants to the neighboring Bioko Island, and it is more likely that this record was of a vagrant nightjar. With hindsight, the species described in this paper is restricted to the old-growth forests of the south (see ‘Distribution’; Freitas 2019), and its presence in the north of the island at that time would be very unlikely as, by then, there were already no suitable forests left in the area.

These different lines of evidence suggested that a candidate species of scops-owl was present on Príncipe. This evidence was summarized, and a plea was made for birders visiting the island to make a special effort to find it (Melo and Dallimer 2009). This plea was finally headed by Philippe Verbelen, who has extensive experience in nocturnal birds (e.g., Verbelen 2010, Sangster et al. 2013, Gwee et al. 2017). Philippe Verbelen visited Príncipe in July 2016 and, together with Felipe Spina (Fauna & Flora International / Fundação Príncipe), Bikegila and Sátiro da Costa obtained the first evidence for the presence of a scops-owl on Príncipe: two rufous morph individuals were photographed on the 4^th^ and 5^th^ of July in the area of Ribeira Porco after descending from the canopy in response to playback (Ryan 2016, Verbelen et al. 2016a, b).

On May 29, 2017, in the Ribeira Porco area (1°33.02N 7°22.29E, Fig. 1 of main text), one individual was captured by Hugo Pereira and Ceciliano do Bom Jesus. The individual was sacrificed and stored to produce the holotype (see main paper).

**References**

Amadon D (1953) Avian systematics and evolution in the Gulf of Guinea. Bulletin of the American Museum of Natural History 100: 394–451. <http://hdl.handle.net/2246/1013>

Borrow N, Demey R (2001) Birds of Western Africa. Christopher Helm, London.

Correia JG (1928) AMNH Gulf of Guinea Expedition Correspondence: Letter to G. C. Murphy, 3 October 1928. Department of Ornithology Archives, American Museum of Natural History, New York.

Dallimer M, Melo M, Collar NJ, Jones PJ (2010) The Príncipe thrush *Turdus xanthorhynchus*: a newly split, Critically Endangered, forest flagship species. Bird Conservation International 20: 375–381. <https://doi.org/10.1017/S0959270910000390>

Freitas B (2019). The mystery Scops owl of Príncipe Island: combining evidence for a species description and assessment of its conservation status. M.Sc. dissertation, University of Porto, Porto. <https://hdl.handle.net/10216/124021>

Gwee CY, L. Christidis L, Eaton JA, Norman JA, Trainor CR, Verbelen P, Rheindt F (2017) Bioacoustic and multi-locus DNA data of *Ninox* owls support high incidence of extinction and recolonisation on small, low-lying islands across Wallacea. Molecular Phylogenetics and Evolution 109: 246–258. <https://doi.org/10.1016/j.ympev.2016.12.024>

Melo M. (1998) Differentiation between Príncipe Island and mainland populations of the African Grey Parrot *Psittacus erithacus* - First field expedition report: 28 October - 3 December 1998. Percy FitzPatrick Institute of African Ornithology, Cape Town. <https://doi.org/10.13140/RG.2.2.24492.28807>

Melo M, Dallimer M (2008) The status of a rare and recently described endemic bird species (the Príncipe Thrush *Turdus* [*olivaceofuscus*] *xanthorhynchus*) and a search for an as yet undescribed 'Owl’. Davis Expedition Fund Report. <https://www.researchgate.net/publication/242266807>

Melo M, Dallimer M (2009) Is there an undiscovered endemic scops owl *Otus* sp. on Príncipe Island? Malimbus 31: 109–115. <http://malimbus.free.fr/articles/V31/31109115.pdf>

Naurois R de (1975) Le “Scops” de I’lle de São Tomé *Otus hartlaubi* (Giebel). Bonner Zoologische Beiträge 26: 319–355. <http://biostor.org/reference/136423>

Naurois R de (1983) Falconidae, Psittacidae et Strigiformes des îles de São Tomé et Principe. Bonner Zoologische Beiträge 34: 429–451. <http://biostor.org/reference/136606>

Ryan P (2016) New scops owl on Príncipe. African Birdlife 5: 10. <http://www.fitzpatrick.uct.ac.za/sites/default/files/image_tool/images/275/Publications/semi-popular/2016/AB05%2801%2910.pdf>

Verbelen P (2010) First field observations of *Ninox (novaeseelandiae) rotiensis* on Roti island, Lesser Sundas, Indonesia. BirdingASIA 13: 85–89. <https://www.researchgate.net/publication/292608131_Asian_enigma_First_field_observations_of_Ninox_novaeseelandiae_rotiensis_on_Roti_island_Lesser_Sundas_Indonesia>

Verbelen P, Melo M, Sangster G, Spina F (2016a) A 90-year-old mystery solved: a potentially new species of owl from Príncipe. Oryx 50: 581. <https://doi.org/10.1017/S0030605316000831>

Verbelen P, Bom Jesus C, Sátiro, Spina F, Melo M (2016b) An unknown Scops-owl in the genus *Otus* discovered in the remote forests of Príncipe Island (Gulf of Guinea, West Africa). Arquipelago - Life and Marine Sciences Supplement 9: 536.

Vieites DR, Wollenberg KC, Andreone F, Köhler J, Glaw F, Vences M (2009). Vast underestimation of Madagascar's biodiversity evidenced by an integrative amphibian inventory. Proceedings of the National Academy of Sciences USA 106: 8267-8272. <https://doi.org/10.1073/pnas.0810821106>
