# Supplementary material for: ﻿A new species of scops-owl (Aves, Strigiformes, Strigidae, Otus) from Príncipe Island (Gulf of Guinea, Africa) and novel insights into the systematic affinities within Otus
Source: Zookeys. 2022 Oct 30;1126:1–54. doi: 10.3897/zookeys.1126.87635 (PMC9836643; doi:10.3897/zookeys.1126.87635)
Supplement: Supplementary material 2 — Alignment [file zookeys-1126-001_article-87635__-s002.docx]

**Concatenated sequences of the phylogenetic dataset*.***

[marker: nd2; length: 1037; no. of seqs: 51; new coordinates: 1-1037)]

Bubo_bubo_111 -----cccccatacaaaactactcgcctccataagcctcctcctaggaacaatcatcactacctcaagtaatcactgaataatagcctgaaccggactagaaatcaacaccctagccatcatccccctaatttcaaaatctcaccacccacgagccatcgaagccacaatcaaatacttcctagtacaagcagccgcctccgccctactactcttctcaagcataatcaacgcctgatcatcaggacaatgggacatcccccaaataaaccaccctataccctgcctactcataacaacagctattgcaataaaactgggactagtcccattccacttctggttcccagaagtcctccaaggaacatctctcatcaccgcactcctcctatcaacgttgataaaactccctccactcaccctcctcctcctaacatcacactcactaaaccctaccctactaactatcatagctatctcctcaacagccctaggaggatgaatgggcctaaaccaaacacaaacccgaaaaatcctagccttctcatccatctcccacctaggttgaataacaatcatcctactctactaccctaaactcaccctactaaccttctacctctacaccataataaccgccgccgtattcctgactctaaacacaaccaaaaccctgagcctaaagacaataataaccgcatgaacaaaggcccccatactaaatacaaccctcatactaaccctactatcactagcaggactaccacccctaactggcttcctgccaaaatgacttatcttacaagaactaaccaaccaagaactaaccccgctcgccacagctactgccatactatctctcctaggactatttttctacctacgactcgcctaccactcaacaatcaccctaccacccaactccacaacccacataaacctgtggcacactaacaacccaacaaacaccattatcgcctccctcacctccacatcaaccctactcctaccactctccccaataatcctagcctccat

Strix_aluco_129 -----cccccacacaaaacttcttacctccctaagcctcttattagggacaaccattgctatctcaagcaatcactgactaatagcctgagccggactagaaatcaacaccctagccattatccctataattgctaaatcgcaccaccctcgagccatcgaagccaccatcaaatacttcctagtacaagcaactgcctccgccctgctactcttctcaagcatgaccaatgcatgatcttcaggacagtgagacatcactcaaatagcctgcaccacaccctgcctccttataacaatggcaattgcaataaaactaggactagtcccattccacttctgattcccagaagtcctccaaggaacatccctcactaccgccctcctactatcaacactaataaaacttcccccactcaccctcctcctactgacatcacactcactaaaccccaccttactgatcaccatagccatctcctcaaccgccctaggaggctgaataggactaaaccaaacacaaactcgaaaaatcctagccttctcatccatctcacacttgggctgaatagccgctatcatcctataccaccccaaactcacactactaaccttctacctatacaccgttataaccaccaccgtattcctaaccttaaacacaaccaagaccatgaacctacaagcaataataattgcatgaacaaaagcccccgtactaaacacaaccctcatactaaccctactctcactggcaggactaccgcctctaaccggcttcctaccaaaatgacttatcctccaagaactaaccaaccaggaactaacccccacagctacaatcatcgctatactatcactcctagggttattcttctatctacgactcgcatactactcaacaatcacccttccaccaaactccacagcccatataaaacaatgacacaccaatagcccaacaaacactatcattgcctccctcacctcctcatcaatattactcctaccactctccccaataatcctagcctccat

Otus_bakkamoena_marathae_35 atgagcccctacacgaaactgctcacctccatgagcctccttctaggtacaactatcaccctctcaagcaatcactgagtgatagcctgaacagggctagaaattaatacactagccatcatccccataatctcaaaatcacatcacccccgagccattgaagccacaatcaaatacttcttagttcaagcaaccgcctccattatactactattctcaagcactacaaatgcatgatcttcaggacaatgagacatcacccaaataacccaccccatatcatgccttaccctaactgtagcaattgcaataaaactaggactagttccattccacttctggttcccagaagtcctccaaggaacaacactcactaccgcactcctactctcaacactaataaaactcccaccactcaccctcctcctactaacatcccactcactaaacccaaccctattaaccacaatagccatcctctcaactgctctgggtggatgaataggcctaaaccaaacacaaacccgaaaaatcttagccttctcatccatctcccacttaggctgaataatcgccatcatcctctactcccccaaactaaccctcctaaccttctgcctctatgccacaataacctcccccgtattcataacactcagcacaaccaaaactacaaacctaccaataataatgatctcatgaacaaaagcccccatactaaacacatccctcatactaaccctcctatcactggccgggcttcccccattaacaggcttcctaccaaaatggctcatcctacaagaactaatcaaccaaggactcacccccgaagccacaatcattgccatactatccctcttaagcctgttcttctacctacgcctagcatactattcaacaatcaccctcccaccaaactcctccacccacacgaaacagtgacatacctgcaactcaccaaacatcaccattgctattctaacctcaatatcaatctcactcctaccgctctccccaatgatcttagcctctat

_Otus_lempiji_CHIMERA --gagcccctacacgaaactactaacttccctaagcctacttctaggtacaaccattaccctctcaagcaaccactgagttatagcctgaacaggactagaaatcaacacactagctattatccccataatctcaaaatcacaccacccccgagccatcgaagccacaatcaaatacttcttagtacaagcaaccgcctccactatgctactattctcaagcactacaaatgcatggtcttcagggcaatgagacatcacccaaataactcaccccatatcatgcctcaccctaacaatagcaattgcaataaaactaggactagttccattccacttttgattcccagaagtcctccaaggaacatcactcactaccgcactcctactttcaacactaataaaactcccaccacttaccctcctcctactgacatcccactcactaaacccagccctattaaccacaatagctatcctctcaacagccctaggtggatgaataggcctaaaccaaacacaaacccgaaaagtcttagccttctcatccatctcccacttaggctgaataatcgccattatcctctactcccccaaactaaccctcttaaccttctgcctctacaccataataacttcctccgtattcataacattcaacacaaccaaaaccctaaacctaccaacaataataatctcatgaacaaaagcccccatactaaatacaaccctcatactaactctcctatcactagccgggcttcccccactaacaggcttcctgccaaaatgactcatcctacaagaactaaccaaccaagggctcacccccgaagccacaatcattgccatactatcactcttaagcctgttcttctacctacgcctagcatactattcaacaatcacccttccaccaaactcctccacccacacaaaacagtgacatacctgcaactcaccaaacatcaccattgccattctaacctcaacatcaatctcactcctaccgctctccccaatgatcttagcctctat

Otus_lettia_lettia_CHIMERA -tgagcccctacacgaaactactaacctccctaagcctccttctaggtacaaccattaccctctcaagcaaccactgagttatagcctgaacaggactagaaatcaacacactagctattatccccataatctcaaaatcacaccacccccgagccatcgaagccacaatcaaatatttcttagtacaagcaaccgcctccactatgctactattctcaagcactacaaatgcatggtcttcagggcaatgagacatcacccaaataactcaccccatatcatgcctcaccctaacaatagcaattgcaataaaactaggactagttccattccacttttgattcccagaagtcctccaaggaacatcactcactaccgcactcctactttcaacactaataaaactcccaccacttaccctcctcctactgacatcccactcactaaacccagccctattaaccacaatagctatcctctcaacagccctaggtggatgaataggcctaaaccaaacacaaacccgaaaagtcttagccttctcatccatctcccacctaggctgaataatcgccattatcctttactcccccaaactaaccctcttaaccttctgcctctacaccataataacttcctccgtattcataacattcaacacaaccaaaaccctaaacctaccaacaataataatctcatgaacaaaagcccccatactaaatacaaccctcatactaactctcctatcactagccgggcttcccccactaacaggcttcctgccaaaatggctcatcctacaagaactaaccaaccaagggctcacccctgaagccacaatcattgccatactatcactcttaagcctgttcttctacctacgcctagcatactattcaacaatcacccttccaccaaactcctccacccacacaaaacagtgacatacctgcaactcaccaaacatcaccattgccattctaacctcaacatcaatctcactcctaccgctctccccaatgatcttagcctctat

Otus_megalotis_CHIMERA ----gcccctacacgaaactacttacctccctcagcctccttctaggtacaaccatcaccctctcaagtaaccactgagtgatagcctgaacagggttagaaatcaatacactagctattatccctataatctcaaaatcacaccacccccgggccatcgaagctacaatcaaatatttcctagtgcaggcaaccgcatccatcatactacttttctcaagcactacaaatgcatggtcctcagggcaatgggacattacccaaataacccaccccacatcatgcctcatcctaacaacagcaattgcaatcaaactaggactagtcccatttcacttctgattcccagaagtcctccaagggacaacactcaccactgcactccttctctcaacactaataaaactcccaccactcaccctcctcctactaacatcccattcactaaacccaaccctattgaccacaatagccatcctctcaacagccctaggtggatgaatgggcctaaaccagacacaaacccgaaagatcctagctttctcatccatctcccacctaggctgaataatcgccatcatcctctactcccccaaactaacccttctaaccttctgtctctacaccacaataacctcctccgtattcataacactcaacacaaccaaaaccataagcctaccaacaatgatagtctcatgaacaaaagcccccgtactaaatacaaccctcatactaaccctcctatcactagccggactccctccactgacaggcttcctgccaaaatgatttattctacaagaactaaccaaccaagaactcacccccgaagccacaatacttgccatactatcccttctaagcctattcttctacttacggctagcgtactattcaacaatcaccctcccaccaaactcctccacccatatgaaacagtgacacacctgcaacccaccaaacactactattgccattctaacctcaatatcgatctcactcctaccgctctccccaatgatcttagcatctat

Otus_nigrorum_CHIMERA ----------------------ttacctccctcagcctcctcctaggtacaaccatcaccctctcaagtaaccactgagtaatagcctgaacaggactagaaattaatacactagctattatccctataatctcgaaatcacaccacccccgagccatcgaagctacaatcaaatacttcctagtgcaagcaaccgcatccatcatactacttttctcaagcactacaaacgcatgatcctcagggcaatgagatatcacccaaataacccatcccacatcatgcctcatcctaacaatagcaattgcaataaaactaggactagtcccatttcacttctgattcccagaagtcctccaaggaacaacacttaccaccgcactccttctctcaacactaataaaactcccaccactcaccctcctcctactaacatcccactcactaaacccaaccctattgaccacaatagccatcctctcaacagccctaggcggatgaataggcctaaaccaaacacaaactcgaaagatcctagccttctcatccatctcccacctaggctgaataatcgccatcatcctctactcccctaaactaacccttctaactttctgcctctacaccacaataacctcctccgtattcataacactcaacacaaccaaaaccacaaacctaccaacaataatagtctcatgaacaaaagcccccgtactaaacacaaccctcatactaaccctcctatcattagccggactcccaccattaacaggcttcctaccaaaatgatttatcctacaagaactaaccaaccaagaactcacccccgaagccacaatacttgccatactatccctcctaagcttattcttctacttacgactagcgtactattcaacaatcaccctcccaccaaactcctccacccatatgaaacagtgacacacctgcaacccaccaaacactactattgccattctaacctcaatatcaatctctctcctaccactctccccaatgatcttagcatctgt

Otus_everetti_CHIMERA atgagcccccacacgaaactacttacctccctcagcctcctcctaggtacaaccatcaccctctcaagtaaccactgagtaatagcctgaacaggactagaaatcaacacactagctattatccccatgatctcaaaatctcaccacccccgagccatcgaggctacaatcaaatacttcctagtgcaagcaaccgcatccatcatactactcttctcaagcactacaaatgcatgatcttcagggcagtgagatattacccaaataacccaccccacatcatgcctcatcctaacaatagcaattgcaataaaactaggactagtcccatttcacttctgattcccagaagtcctccaagggacaacactcaccagcgcactccttctctcaacactaataaaactcccaccactcaccctcctcctactaacatcccactcactaaacccaaccctattgaccctaatagccatcctctcaacagccctaggcggatgaataggcctaaaccagactcaaactcgaaagatcctggctttctcgtccatctcccacctgggctgaataatcgccgtcatcctctactcccctaaactaacccttctaaccttctgcctctacaccacaataacctcctctgtattcataacactcaacacaaccaaaaccacaagcctaccaacaataatagtctcatggacaaaagcccccgtactaaatacaaccctcatactaaccctcctatcattggccggacttcccccattgacaggcttcttaccaaaatgatttatcctacaagaactaaccaaccaagaactcacccccgaagccacaatgcttgccatactatcccttctaagcctattcttctacttacggctagcgtactattcaacaatcaccctcccgccaaactcctccacccatatgaaacagtgacacacctgcaacccaccaaacactactattgccattctaacctcaatatcaatctcactcctaccactctccccaatgatcttagcca----

Otus_semitorques_CHIMERA -tgagcccccacacaaaattacttacctccctcagcctccttctaggtacaaccatcaccctctcaagtaaccactgaataatagcctgaacaggactagaaatcaatacactagccattattcctataatctcaaaatcacatcaccctcgagccatcgaggctacaatcaaatacttcctagtacaagcaaccgcctccatcatactactcttctcaagcactacaaacgcatgatcctcagggcaatgagatatcacccaaataacccaccccacatcatgcctcatcctaacaatagcaattgcaataaaactaggactagttccatttcacttctgattcccagaagtccttcaggggacaacacttaccactgcactccttctctcaacactaataaaactcccacctctcaccctcctcctactgacatcccactcactaaacccaaccctactaaccacaatagccatcctctcaacagccctaggcggatgaataggcctaaaccaaacacaaactcgaaagatcctagctttctcatccatctcccacctaggctgaataaccgccatcatcctctactcccccaaactaacccttctaaccttctgcctctactccgcaataacctcctccgtattcataacactcaacacaaccaaaaccataaacctcccaacaataatgatctcgtgaacaaaagcccctgtactaaattcaaccctcatactaaccctcctatcattagctggactccccccactaacaggcttcctaccaaaatgacttatcctacaagagctaactaaccaagaactcacccccgaagccacaatcattaccatactatctcttctaagcctattcttctacttacgactagcatactactcaacaatcaccttaccaccaaactcctccacccacatgaaacagtgacacacctgcaacccaccaagcactactaccgccattctaacctcaatatcaatctcactcctaccactcaccccaatgatcttagcctctat

Otus_angelinae_29 atgagcccctacacgaaactacttacctccctcagcctccttctaggcacaaccatcaccctctcaagtaaccactgagtaatagcctgaacaggattagaaatcaatacactagctattattcctataatctcaaaatcacaccaccctcgagccatcgaggctacaatcaaatacttcctagtacaagcaaccgcctctatcatactacttctctcaagcactacaaacgcatgatcctcaggacagtgggatatcacccaaataacccaccctacatcatgcctcatcctaacaatagcaattgcaataaaactaggactagccccatttcacttctgattcccagaagtcctccaaggaacaacactcatcactgcactccttctctcaacactaataaaactcccacccctcaccctcctcctactaacatcccactcactaaacccaaccctactggccacaatagccatcctctcaacagccctaggtggatgaatgggcctgaaccaaacgcaaactcgaaagatcttagctttctcatccatctcccacctgggctgaataatcgccatcatcctctactcccccaaactaacccttctaaccttttgcctctataccacaataacctcctccgtattcataacgctcaacacaaccaaaaccataagcctaccaacaataatgatctcatgaacaaaagcccccgtactaaattcaaccctcatactaactctcctatcattagccggacttcccccgttgacaggcttccnnnnnnnnnnnnnnnnnnnnnnnnnnnnnnnnnnnnnnnnnnnnnnnnnnnnnnnnnnnnnnnnnnnnnnnnnnnnnnnnnnnnnnnnnnnnnnnnnnnnnnnnnnnnnnnnnnnnnnnnnnnnnnnnnnnnnnnnnnnntcctccacccgcatgaaacagtgacacacctgcaacccgccaaacacaactattgccattctaacctcaatatcaatctcactcctaccgctctccccaatgatcttagcctctat

Otus_spilocephalus_vandewateri_97 atgagcccatacacgaaactacttgcctccataagcctcctcctaggcacaaccatcactctctcgagcaaccactgagtgatagcctgaacaggactagaaatcaacacactagccatcatccccataatctcaaaatcacaccacccccgagctattgaagccacaatcaaatacttcctagtacaagcaaccgcctccaccatactactcttctcaagcactacaaatgcatggtcctcagggcaatgagacatcacccaaataacccaccccacatcatgccttctcctaacaacagcaattgcaataaaactaggactagtcccattccacttctgattcccagaagtcctacaaggaacatcactcaccaccgcactcctactctcaacacttataaaacttcccccgctcactctcctcctactaacatccaattcgctaaacccaatcttattaacctccatagctatcctcnnnnnnnnnnnnnnnnnnnnnnnnnnnnnnnnnnnnnnnnnnnnnnnnnnnnnnnnnnnnnnnnnnnnnnnnnnnnnnnnnnnnnnnnnnnnnnnnnnnnnnnnnnnnnnnnnnnnnnnnnnnnnnnnnnnnnnnnnnnnnnnnnnnnnnnnnnnnnnnnnnnnnnnnnnnnnnnnnnnnnnnnnnnnaactacaaacctgccaacaataatgatctcatgaacaaaagcacccgtactaaacacaaccctcatactaaccctcctgtcactagctggcctgccccctctaacaggcttcctnnnnnnnnnnnnnnnnnnnnnnnnnnnnnnnnnnnnnnnnnnnnnnnnnnnnnnnnnnnnnnnnnnnnnnnnnnnnnnnnnnnnnnnnnnnnnnnnnnnnnnnnnnnnnnnnnnnnnnnnnnnnnnnnnnnnnnnnnnnnnnnnnnnnnnnnnnnnnnnnnnnnnnnnnaacaacccaccaaacaccaccattgccattctaacctcaatatcgatcgcactcttgccgctctccccggtgatcttagccactat

Otus_spilocephalus_vulpes_96 atgagcccgtacacgaagctacttacctccataagcctccttctaggcacaaccatcactctctcaagcaaccactgggtgatagcctgaacaggactagaaatcaacacactagccatcatccccat---------------------------------------------------------------------------------------------------------------------------------------------------------------------------------------------------------------------------------------------------------------------------------------------------------------------------------------------------------------------------------------------------------------------------------------------------------------------------------------------------------------------------------------------------------------------------------------------------------------------------------------------------------------------------------------------------------------------------------------------------------------------------------------------------------------------------------------------------------------------------------------------------------------------------------------------

Otus_spilocephalus_spilocephalus_90 -----cccatacacgaaactacttacctctataagcctccttctaggcacagccatcactctctcaagcaaccactgagtgatggcctggacaggactagaaatcaacacactagccattatccccataatctcaaaatcacaccacccccgagctattgaagccacaatcaaatacttcctagtacaggcaaccgcctccaccatactactcttctcgagcactacaaatgcatgatcttcaggacaatgagacatcacccaaataacccaccctatatcatgcctcatcctaacaacagcaattgcaataaaactaggactagtcccattccacttctgatttccagaagtccttcaaggaacatcactcaccaccgcactcctgctctcaacacttataaaactaccaccactcgccctccttctactaacatccaactcactaagcccaatcctattaacttctatagctatctcctcaacagcccttggtggatgaatgggcctaaaccaaacacaagtccgaaagatcttagccttctcatccatctcccacctaggctgaatggttgccattatcctatactcccccaaactgaccctcctagccttctgcctctacaccacaataacctcctccgtgttcataacactcaacacaaccaaaaccacaaacctaccgacaataatgatctcatggactaaagcacctattctaaacacaaccctcatactaaccctcctatcactagctggcctcccccccttaacgggcttcctcccaaaatgactcattctacaagaactaaccaaccaaaaactcacccccgaagccacaatcattgccatactatccctcctaagcctcttcttctacctacgcctagcatactactcaacaatcaccctcccaccaaactccgccacccacacaaaacagtgacacaccagcaacccgccaaacaccaccattgccattctaacctcgatatcgatctcactcttgccgctctccccagtgatcttagccactat

Otus_spilocephalus_hambroecki_92 atgagcccctacacaaaactacttacctctataagcctccttctaggcacaaccatcactctctcaagcaaccactgagtgatggcctgagcaggactagagatcaacacactagccattatccccataatctcaaaatcacaccaccccnnnnnnnnnnnnnnnnnaatcaaatacttcctagtacaggcaaccgcctccaccatactactcttctcaagcactacaaatgcatgatcttcaggacagtgagacatcacccaaataacccacccaacatcatgcctaatcctaacaacagcaattgcaatgaaactaggactagtcccattccacttctgattcccagaagtccttcaaggaacatcactcaccaccgcactcctgctctcaacacttataaaactaccaccactcaccctccttctacttacatccaattcactaaacccaaccctattagcttctatagctatctccnnnnnnnnnnnnnnnnnnnnnnnnnnnnnnnnnnnnnnnnnnnnnnnnnnnnnnnnnnnnnnnnnnnnnnnnnnnnnnnnnnnnnnnnnnnnnnnnnnnnnnnnnnnnnnnnnnnnnnnnnnnnnnnnnnnnnnnnnnnnnnnnnnnacaataacctcctccgtgttcatatcactcaacacaaccaaaaccacaaacctaccaacaataatgatctcatgggctaaagcacccatactaaacacaaccctcatactaaccctcctatcactagccggccttcctcccttaacgggcttcctcccaaaatgactcattctacaagaactaaccaaccaaaaactcacccccgaagccacaatcattgccatactatccctcctaagcctcttcttctacctacgcctagcataccactcaacaatcacccttccgccaaactccaccacccacacaaaacagtgacacaccagcaacccgccaaacaccaccattgccattctaacctcgatatcgatctcactcttgccgctctccccagtgatcttagccactat

_Otus_spilocephalus_latouchi_CHIMERA --gagcccgtacacaaaactacttacctctataagcctccttctaggcacaaccatcactctctcaagcaaccactgagtgatggcctgagcaggactagagatcaacacactagccatcatccccataatctcaaaatcacaccacccccgagctattgaagctacaatcaaatacttcctggtacaggcaaccgcctccaccatactactcttctcaagcactacaaatgcatgatcttcaggacaatgagacattacccaaataacccacccaacatcatgcctcatcctaacaacagcaattgcaatgaaactaggactagtcccattccacttctgattcccagaagtccttcaaggaacatcactcaccaccgcactcctactctcaacactcataaaactaccaccactcaccctccttctactaacatccaattcactaaacccaatcctattaacttctatagctatctcctcaacagccctgggtggatgaatgggtttaaaccaaacacaagtccgaaagatcctagccttctcatccatctcccacctaggctgaatagttgccattatcctatactcccccaaactaaccctcctaaccttctgcctctacaccacaataacctcctccgtattcatatcactcaacacaaccaaaaccacaaacctaccaacaataatgatctcatggactaaagcacccatactaaacacaaccctcatgttaaccctcctatcactagccggccttccccccctaacaggcttcctcccaaaatgacttattctgcaagaactaaccaaccaaaaactcacccccgaagccacaatcattgccatactatccctcctaagcctcttcttctacctacgcctagcataccactcaacaatcacccttccaccaaactctaccacccacacaaaacagtgacacaccagcaacccaccaaacaccaccattgccattctaacctcaatatcgatctcactcttaccgctctccccagtgatcttagccactat

Otus_hartlaubi_13 ---------cacactaaactacttacctcactaagcctcctcctgggaacaaccatcaccatctcgagcaatcactgagtaatagcctgaacagggttagaaatcaacacactagcaatcatccctataatctcaaaatcacaccatccccgagccattgaagccaccatcaaatacttcctagtacaagcaaccgcctccatcatactactcttctcaagcaccataaatgcatgatcctcaggacaatgagacatcacacaaataactcaccctacgtcatgccttatcctaacaacagccattgcaataaaactaggactagtcccattccacttttgactcccagaagtcctccaaggaacatcactcaccaccgcactcctactttcaacactaataaaactcccacccctcaccctcctcctactaacctcccactcactaaacccaaccctactaaccactatggccatctcctcaacagccttaggaggatgaataggcctaaaccaaacacaaatccgaaaaatcttagccttctcatccatctcccacctaggctgaataactgctatcaccctctactcccccaaactggccctcctaactttctgcctctacgttacaataaccacctctgtatttataacattcaacacaaccaaaaccacaaacctatcaaccataataacctcatgaaccaaagcccccatactcaatacaaccctcatactaaccctcctttcactagccggcctcccacccctatcgggtttcctaccaaaatgactcatcctacaagaactagtcaatcaagaactcacccctaaagccacaatcattgctctactatcacttctaggcctattcttctacctacgcctagcgtactactcaacaatcaccctaccaccaaactcctccactcacacaaaacaatgacacaccaacaacccaacaacaaccctcattgccattctcacctcaatatcaatcgcactcctgccgctctccccggtgatcttagccactat

Otus_hartlaubi_15 ----------acactaaactacttacctcactaagcctcctcctgggaacaaccatcaccatctcgagcaatcactgagtaatagcctgaacagggttagaaatcaacacactagcaatcatccctataatctcaaaatcacaccatccccgagccattgaagccaccatcaaatacttcctagtacaagcaaccgcctccatcatactactcttctcaagcaccataaatgcatgatcctcaggacaatgagacatcacacaaataactcaccctacgtcatgccttatcctaacaacagccattgcaataaaactaggactagtcccattccacttttgattcccagaagtcctccaaggaacatcactcaccaccgcactcctactttcaacactaataaaactcccacccctcaccctcctcctactaacctcccactcactaaacccaaccctactaaccactatggccatctcctcaacagccttaggaggatgaataggcctaaaccaaacacaaatccgaaaaatcttagccttctcatccatctcccacctaggctgaataactgctatcaccctctactcccccaaactggccctcctaactttctgcctctacgttacaataaccacctctgtatttataacattcaacacaaccaaaaccacaaacctatcaaccataataacctcatgaaccaaagcccccatactcaatacaaccctcatactaaccctcctttcactagccggcctcccacccctatcgggtttcctaccaaaatgactcatcctacaagaactagtcaatcaagaactcacccctaaagccacaatcattgctctactatcacttctaggcctattcttctacctacgcctagcgtactactcaacaatcaccctaccaccaaactcctccactcacacaaaacaatgacacaccaacaacccaacaacaaccctcattgccattctcacctcaatatcaatcgcactcctgccgctctccccgg----------------

Otus_hartlaubi_16 -----------cactaaactacttacctcactaagcctcctcctgggaacaaccatcaccatctcgagcaatcactgagtaatagcctgaacagggttagaaatcaacacactagcaatcatccctataatctcaaaatcacaccatccccgagccattgaagccaccatcaaatacttcctagtacaagcaaccgcctccatcatactactcttctcaagcaccataaatgcatgatcctcaggacaatgagacatcacacaaataactcaccctacgtcatgccttatcctaacaacagccattgcaataaaactaggactagtcccattccacttttgattcccagaagtcctccaaggaacatcactcaccaccgcactcctactttcaacactaataaaactcccacccctcaccctcctcctactaacctcccactcactaaacccaaccctactaaccactatggccatctcctcaacagccttaggaggatgaataggcctaaaccaaacacaaatccgaaaaatcttagccttctcatccatctcccacctaggctgaataactgctatcaccctctactcccccaaactggccctcctaactttctgcctctacgttacaataaccacctctgtatttataacattcaacacaaccaaaaccacaaacctatcaaccataataacctcatgaaccaaagcccccatactcaatacaaccctcatactaaccctcctttcactagccggcctcccacccctatcgggtttcctaccaaaatgactcatcctacaagaactagtcaatcaagaactcacccctaaagccacaatcattgctctactatcacttctaggcctattcttctacctacgcctagcgtactactcaacaatcaccctaccaccaaactcctccactcacacaaaacaatgacacaccaacaacccaacaacaaccctcattgccattctcacctcaatatcaatcgcactcctgccgctctccccggtgatcttag-------

Otus_hartlaubi_14 ---------------aaactacttacctcactaagcctcctcctgggaacaaccatcaccatctcgagcaatcactgagtaatagcctgaacagggttagaaatcaacacactagcaatcatccctataatctcaaaatcacaccatccccgagccattgaagccaccatcaaatacttcctagtacaagcaaccgcctccatcatactactcttctcaagcaccataaatgcatgatcctcaggacaatgagacatcacacaaataactcaccctacgtcatgccttatcctaacaacagccattgcaataaaactaggactagtcccattccacttttgattcccagaagtcctccaaggaacatcactcaccaccgcactcctactttcaacactaataaaactcccacccctcaccctcctcctactaacctcccactcactaaccccaaccctactaaccactatggccatctcctcaacagccttaggaggatgaataggcctaaaccaaacacaaatccgaaaaatcttagccttctcatccatctcccacctaggctgaataactgctatcaccctctactcccccaaactggccctcctaactttctgcctctacgttacaataaccacctctgtatttataacattcaacacaaccaaaaccacaaacctatcaaccataataacctcatgaaccaaagcccccatactcaatacaaccctcatactaaccctcctttcactagccggcctcccacccctatcgggtttcctaccaaaatgactcatcctacaagaactagtcaatcaagaactcacccctaaagccacaatcattgctctactatcacttctaggcctattcttctacctacgcctagcgtactactcaacaatcaccctaccaccaaactcctccactcacacaaaacaatgacacaccaacaacccaacaacaaccctcattgccattctcacctcaatatcaatcgcactcctgccgctctccccggtgatcttagcca----

Otus_feae_45 atgagcccacacactaaactacttacctcactaagcctcctcctaggaacaactatcaccatctcgagcaatcactgagtaatagcctgaacaggattagaaatcaacacactagcaatcatccctataatctcaaaatcacaccacccccgagccattgaagccaccatcaaatacttcctagtgcaagcaaccgcctccatcatactactcttctcaagcaccataaatgcatgatcctcaggacaatgagacatcacacaaataacccaccccacgtcatgcctcatcctaacaacagccattgcaataaaactaggactagtcccattccacttttgattccctgaagtcctccaaggaacatcactcaccactgcactcctactttcaacactaataaaactcccacccctcaccctcctcctactaacctcccactcactaaatccaaccctactaaccaccatggccatctcctcaacagccctgggaggatgaataggcctaaaccaaacacaaatccgaaagatcttagccttctcatccatctcccacctaggctgaatagccgctatcatcctctactcccctaaactaaccctcctaaccttctgcctctacgtcacaataaccacctctgtatttataacattcaacacaaccaaaaccacaaacctaccaaccataataacctcatgagccaaagcccccatactcaatacaactctcatactaaccctcctttcactagctggcctcccacccctatcgggcttcctaccaaaatgactcatccttcaagaactagtcaatcaagaactcacccctgaagccacaatcattgctatactatcacttttaagcctatttttctacctacgcctagcgtactactcaacaatcactctaccaccaaactcctccacccacacaaaacaatgacacaccaacaacccaacaacaaccctcattgccattctcacctcaatatcaatcgcactcctgccgctctccccggtgatcttagccactat

Otus_senegalensis_17 atgagcccacacactaaactacttacctcactaagcctcctcctaggaacaactatcaccatctcgagcaatcactgagtaatagcctgaacaggattagaaatcaacacactagcaatcatccctataatctcaaaatcacaccatccccgagccattgaagccaccatcaaatacttcctagtacaagcaaccgcctccatcatactactcttctcaagcaccataaatgcatgatcctcaggacaatgagacatcacacaaataacccaccccacgtcatgcctcatcctaacaacagccattgcaataaaactaggactagtcccattccacttttgattccctgaagtcctccaaggaacatcactcaccactgcactcctactttcaacactaataaaactcccacccctcaccctcctcctactaacctcccactcactaaatccaaccctactaaccactatggccatctcctcagcagccctgggaggatgaataggcctaaaccaaacacaaatccgaaagatcttagccttctcatccatctcccacctaggctgaatagccgctatcatcctctactcccctaaactaaccctcctaaccttctgcctctacgtcacaataaccacctctgtatttataacattcaacacaaccaaaaccacaaacctaccaaccataataacctcatgaaccaaagcccccatactcaatacaactctcatactaaccctcctttcactagctggcctcccacccctatcgggcttcctaccaaaatgactcatccttcaagaactagtcaatcaagaactcacccctgaagccacaatcattgctatactatcacttttaagcctatttttctacctgcgcctagcgtactactcaacaatcactctaccaccaaactcctccactcacacaaaacaatgacacaccaacaacccaacaacaaccctcattgccattctcacctcaatatcaatcgcactcctgccgctctccccggtgatcttagccactat

Otus_pamelae_74 ------ccacacactaaactacttacctcactaagcctcctcctagggacaaccatcaccatctcgagcaatcactgagtaatagcctgaacaggattagaaatcaacacactagcaattatccctataatctcaaaatcacaccacccccgagccatcgaagccaccatcaaatacttcctagtacaagcaaccgcctccatcatactactcttctcaagcaccacaaatgcatgatcctcaggacaatgagacatcacacaaataacccaccccatgtcatgcctcatcctaacaacagccattgcaataaaactaggactagtcccattccacttttgattcccagaagttctccaaggaacatcactcaccaccgcactcctactttcaacactaataaaactcccacccctcaccctcctcctactaacctcccactcactaaacccaaccctgctaaccaccatagccatctcctcaacagccctaggaggctg---------------------------------------------------------------------------------------------------------------------------------------------------------------------------------------------------------------------------------------------------------------------------------------------------------------------------------------------------------------------------------------------------------------------------------------------------------------------------------------------------------------------------------------------------------

Otus_sp_nov_1 -----cccgcacactaaactacttacctcactaagcctcctcctaggaacaaccatcaccatctcgagcaatcactgagtaatagcctgaacaggattagaaatcaacacactagcaatcatccctataatctcaaaatcacaccatccccgagccattgaagccaccatcaaatacttcctagtacaagcaaccgcctccatcatactactcttctcaagcaccacaaatgcatgattctcaggacaatgagacatcacacaaataactcaccccacgtcatgcctcatcctaacaacagctattgcaataaaactaggactagtcccattccacttttgattcccagaagtactccaaggaacatcactcgccaccgcactcctactttcaacactaataaaactcccacccctcaccctcctcctactaacctcccactcactaaacccaaccctactaaccactatggccatctcctcaacagccctagggggatgaatgggcctaaaccaaacacaaatccgaaaaatcttagccttctcatccatctcccacctaggctgaataaccgctatcaccctctactcccccaaactaaccctcctaaccttctgcctctacattacaataaccacctccgtatttataacatttaacgtaactaaaaccacaaacctaccaaccataataacctcgtgaaccaaagcccccatacttaatacaaccctcatactaaccctcctctcactagccggcctcccacccctatcaggcttcctaccaaaatgactcatcctacaagaactagtcaatcaggaactcacccctgaagc---------------------------------------------------------------------------------------------------------------------------------------------------------------------------------------------------------

Otus_sp_nov_2 ---------------aaactacttacctcactaagcctcctcctaggaacaaccatcaccatctcgagcaatcactgagtaatagcctgaacaggattagaaatcaacacactagcaatcatccctataatctcaaaatcacaccatccccgagccattgaagccaccatcaaatacttcctagtacaagcaaccgcctccatcatactactcttctcaagcaccacaaatgcatgattctcaggacaatgagacatcacacaaataactcaccccacgtcatgcctcatcctaacaacagctattgcaataaaactaggactagtcccattccacttttgattcccagaagtactccaaggaacatcactcgccaccgcactcctactttcaacactaataaaactcccacccctcaccctcctcctactaacctcccactcactaaacccaaccctactaaccactatggccatctcctcaacagccctagggggatgaatgggcctaaaccaaacacaaatccgaaaaatcttagccttctcatccatctcccacctaggctgaataaccgctatcaccctctactcccccaaactaaccctcctaaccttctgcctctacattacaataaccacctccgtatttataacatttaacgtaactaaaaccacaaacctaccaaccataataacctcgtgaaccaaagcccccatacttaatacaaccctcatactaaccctcctctcactagccggcctcccacccctatcaggcttcctaccaaaatgactcatcctacaagaactagtcaatcaggaactcacccctgaagccacaatcattgctatattatcactcttaggcctatttttctacctacgcctagcgtactactcaacaatcaccctaccaccaaactcctccactcacacaaaacaatgacacaccaacaacccaacaacaaccctcattgccattctcacctcaatatcaatcgcactcctgccgctctccccggtgatctta--------

Otus_sp_nov_3 --------------------------------------------------------------------------------------------------------------------------------------------------------------------------------------------------------------------------------------------------------------------------------------------------------------------------------------------------------------------------------------------------------------------------------------------------------ctcactaaacccaaccctactaaccactatggccatctcctcaacagccctagggggatgaatgggcctaaaccaaacacaaatccgaaaaatcttagccttctcatccatctcccacctaggctgaataaccgctatcaccctctactcccccaaactaaccctcctaaccttctgcctctacattacaataaccacctccgtatttataacatttaacgtaactaaaaccacaaacctaccaaccataataacctcgtgaaccaaagcccccatacttaatacaaccctcatactaaccctcctctcactagccggcctcccacccctatcaggcttcctaccaaaatgactcatcctacaagaactagtcaatcaggaactcacccctgaagccacaatcattgctatattatcactcttaggcctatttttctacctacgcctagcgtactactcaacaatcaccctaccaccaaactcctccactcacacaaaacaatgacacaccaacaacccaacaacaaccctcattgccattctcacctcaatatcaatcgcactcctgccgctctccccggtgatctta--------

Otus_sp_nov_4 ----------------------------------------------------------------------------------------------------------------------------------------------------------------------------------------------------------------------------------------------------------------------------------------------------------------------------------------------------------------------------------------------------------------------------------------------------------------------------taaccactatggccatctcctcaacagccctagggggatgaatgggcctaaaccaaacacaaatccgaaaaatcttagccttctcatccatctcccacctaggctgaataaccgctatcaccctctactcccccaaactaaccctcctaaccttctgcctctacattacaataaccacctccgtatttataacatttaacgtaactaaaaccacaaacctaccaaccataataacctcgtgaaccaaagcccccatacttaatacaaccctcatactaaccctcctctcactagccggcctcccacccctatcaggcttcctaccaaaatgactcatcctacaagaactagtcaatcaggaactcacccctgaagccacaatcattgctatattatcactcttaggcctatttttctacctacgcctagcgtactactcaacaatcaccctaccaccaaactcctccactcacacaaaacaatgacacaccaacaacccaacaacaaccctcattgccattctcacctcaatatcaatcgcactcctgccgctctccccggtgatctta--------

Otus_scops_5 atgagcccacacactaagctacttacctcactaagcctcctcctaggaacaaccatcaccatctcgagcaatcactgagtaatagcctgaacagggttagaaatcaacacactagcaattatccccataatctcaaaatcacaccacccccgagccatcgaagccaccatcaaatacttcctagtacaagcaaccgcctccatcatactactcttctcaagcaccataaatgcatggtcctcaggacaatgagacatcacacaaataactcaccccatgtcatgcctcatcctaacaacagccattgcaataaaactaggactagtcccattccacttttgattcccagaagtcctccaaggaacatcactcaccaccgcactcctactttcaacccttataaaactcccacccctcaccctccttatactaacctcccactcacttaacccaaccctactaaccactatagccatctcctcaacagccctaggaggatgaataggcctaaaccaaacacaaatccgaaaaatcttagccttctcatccatctcccacctaggctgaataaccgctaccatcctctactcccccaaactaacccttctaaccttcttcctctacattacaataaccacctccgtatttataacattcaacacaactaaaaccacaaacttaccaaccataataacctcctggaccaaagcccccatacttaatacaaccctcatactaaccctcctctcactagccggcctcccacccctatcaggtttcctaccaaaatgactcatcctacaagaactagtcaatcaagaactcactcctgaagccacaatcattgccatactatcactcttaagcctatttttctacctacgcctagcgtactactcaactatcaccctaccaccaaactcctccactcacacaaaacaatgacacaccaacacccaaacaacaacccccattgctattctcacctcaatatcaatcgcactcctgccgctctccccggtgatcttagccactat

_Otus_scops_JF5337_F --gagcccacacactaagctacttacctcactaagcctcctcctaggaacaaccatcaccatctcgagcaatcactgagtaatagcctgaacagggttagaaatcaacacactagcaattatccccataatctcaaaatcacaccacccccgagccatcgaagccaccatcaaatacttcctagtacaagcaaccgcctccatcatactactcttctcaagcaccataaatgcatggtcctcaggacaatgagacatcacacaaataactcaccccatgtcatgcctcatcctaacaacagccattgcaataaaactaggactagtcccattccacttttgattcccagaagtcctccaaggaacatcactcaccaccgcactcctactttcaacccttataaaactcccacccctcaccctccttatactaacctcccactcacttaacccaaccctactaaccactatagccatctcctcaacagccctaggaggatgaataggcctaaaccaaacacaaatccgaaaaatcttagccttctcatccatctcccacctaggctgaataaccgctatcatcctctactcccccaaactaacccttctaaccttcttcctctacattacaataaccacctccgtatttataacattcaacacaactaaaaccacaaacttaccaaccataataacctcctggaccaaagcccccatacttaatacaaccctcatactaaccctcctctcactagccggcctcccacccctatcaggtttcctaccaaaatgactcatcctacaagaactagtcaatcaagaactcactcctgaagccacaatcattgccatactatcactcttaagcctatttttctacctacgcctagcgtactactcaactatcaccctaccaccaaactcctccactcacacaaaacaatgacacaccaacacccaaacaacaacccccattgctattctcacctcaatatcaatcgcactcctgccgctctccccggtgatcttagccactat

Otus_pembaensis_24 atgagtccacacaccaaactacttacctcactaagcctcctcctgggaacaaccatcaccatctcgagcaatcactgagtaatagcctgaacagggttagaaatcaacacactagcaatcatccctataatctcaaaaccacaccatccccgagccattgaagccaccatcaaatacttcctagtacaagcagctgcctccatcatactactcttctcaagcaccataaatgcatgatcctcaggacaatgagacatcacacaaataactcaccccacatcatgcttcatcctaacaacagccattgcaataaaactaggactagtcccattccacttttgattcccagaagtcctccaaggaacatcactcaccgccgcactactcctttcaacactaataaaactcccacccctcaccctcctcctactaacctcccactcactaaacccaaccctgctaaccactatggccatctcctcaacagccctaggaggatgaataggcctaaaccaaacacaaatccgaaaactcttagccttctcatccatctcccacctaggatgaataaccgctatcatcctctactcccccaaactaaccctcctaaccttctgcctctacgttacaataaccacctctgtatttctaacattcaacacaactaaaaccacaaacctaccaaccataataacctcatgaaccaaagcccccatactcaatacaaccctcatactaaccctcctctcactagccggcctcccacccctatcaggcttcttaccaaaatgacttatcctacaagagctaaccaatcaagaactcacccctgaagccacaatcattgctatactatcacttttaagcctatttttctacctacgcctagcgtactactcaacaatcaccctaccaccaaattcctcccctcacacaaaacaatgatacaccaacaacccaacaacaaccctcgttgccattctcacctcaatatcaatcgcactcctgccgctctccccggtgatcttagccactat

Otus_pembaensis_25 atgagtccacacaccaaactacttacctcactaagcctcctcctgggaacaaccatcaccatctcgagcaatcactgagtaatagcctgaacagggttagaaatcaacacactagcaatcatccctataatctcaaaaccacaccatccccgagccattgaagccaccatcaaatacttcctagtacaagcagctgcctccatcatactactcttctcaagcaccataaatgcatgatcctcaggacaatgagacatcacacaaataactcaccccacatcatgcttcatcctaacaacagccattgcaataaaactaggactagtcccattccacttttgattcccagaagtcctccaaggaacatcactcaccgccgcactactcctttcaacactaataaaactcccacccctcaccctcctcctactaacctcccactcactaaacccaaccctgctaaccactatggccatctcctcaacagccctaggaggatgaataggcctaaaccaaacacaaatccgaaaactcttagccttctcatccatctcccacctaggatgaataaccgctatcatcctctactcccccaaactaaccctcctaaccttctgcctctacgttacaataaccacctctgtatttctaacattcaacacaactaaaaccacaaacctaccaaccataataacctcatgaaccaaagcccccatactcaatacaaccctcatactaaccctcctctcactagccggcctcccacccctatcaggcttcttaccaaaatgacttatcctacaagagctaaccaatcaagaactcacccctgaagccacaatcattgctatactatcacttttaagcctatttttctacctacgcctagcgtactactcaacaatcaccctaccaccaaattcctcccctcacacaaaacaatgatacaccaacaacccaacaacaaccctcgttgccattctcacctcaatatcaatcgcactcctgccgctctccccggtgatcttagccactat

_Otus_longicornis_CHIMERA --gagcccacatacaaaactactcacctcactaagcctcctcctgggaacaaccatcaccatctcgagcaatcactgagtaatggcctgagcaggactagaaatcaacacactagcaattatccccataatctcaaaatcacatcacccccgagccattgaagccaccattaaatacttcctagtacaagcaaccgcttccatcatattactcttctcaagcaccacaaacgcatgatcctcaggacaatgagacatcacacaaataacccaccccacatcatgcctcatcctaacaacagctattgcaataaaactaggactagtcccattccacttttgattcccggaagtcctccaaggcacatcacttaccacagcactcctactctcaacactaataaaactcccaccacttactctcctccttctaacctcccactcactaaacccaaccctactaaccaccatagctatctcctcaacagccctaggaggatgaataggcctaaaccaaacacaaatccgaaaaatcctagccttctcatccatctcccacctaggctgaataactgctatcatcctctactcccccaaactaaccctcctgaccttctacctctacattacaataaccacctccgtattcataacattcaacacaactaaaaccacaaacttaccaactataataacctcatgaactaaagcccccatacttaacacaaccctcatactaactctcctctcattagccggactcccacccctatcaggcttcctaccaaaatgacttatcctacaagaactagttaatcaagaactcacccccgaagccacaatcattgccatattatcactcctaggactattcttctacctacgcctagcatactactcaacaatcaccctaccaccaaactcctccacccacacaaaacaatgatacaccaacaatccaacaacaacccccattgccattctcacctcaatatcaatttcactcttgccgctctccccagtgatcttagccgctat

Otus_mirus_CHIMERA atgagcccacatacaaaactactcacctcactaagcctcctcctgggaacaaccatcaccatctcgagcaatcactgagtaatagcctgaacaggactagaaatcaatacactagcaattatccccataatctcaaaatcacatcacccccgagccatcgaagccaccattaaatacttcctagtacaagcaaccgcttccatcatattactcttctcaagcaccacaaacgcatgatcttcaggacaatgggacatcacacaaataacccaccccacatcatgcctcatcctaacaacagctattgcaataaaactaggactagtcccattccacttttgattcccggaagtcctccaaggcacatcacttaccaccgcactcctactttcaacactaataaaactcccaccacttactctcctccttctaacctcacactcactaaacccaaccctactagccaccatagctatctcctcaacagccctaggaggatgaataggcctaaaccaaacacaaatccgaaaaatcctagccttctcgtccatctcccacctgggctgaataaccgctatcatcctctactcccccaaactaaccctcctaaccttctgcctatacattacaataaccacctccgtattcataacgttcaacacaactaaaaccacaaacttaccgaccataataacctcatgaactaaagcccccatacttaacacaaccctcatactaactctcctctcactagccggacttccacccctatcaggcttcctaccaaaatgacttatcctacaagaactagttaaccaagaactcaccccagaagccacagtcattgccatactatcactcctaggactattcttctacctacgcctagcatactactcaacaatcaccctgccaccaaactcctccacccacacaaaacagtgatgcaccaacaatccaacaacaacccccattgccattctcacctcaatatcaatttcactcttgccgctctccccaatgatcttagccgctat

Otus_mayottensis_60 -----cccacatacaaaactactcacctcactaagcctcctcctaggaacagccatcaccatctcgagcaaccactgagtaatggcctgaacaggactagagatcaatacactagcaattatccccataatctcaaaatcacaccacccccgagccatcgaagccaccattaagtacttcctagtacaagcaaccgcctccatcatactactcttctcaagcaccacaaacgcatgatcctcaggacaatgagacatcacacagataacccaccccacatcatgcctcattctaacaacagctattgcaataaaactaggactagccccattccacttctgattcccagaagtcctccaaggcacatcacttaccaccgcactcctactctctacactaataaaactcccaccgctcactctcctcctactaacctctcactcactaaactcaaccctactaactaccatagctatctcctcaacagccctgggaggatgaataggcctaaaccaaacacaaatccgaaaaatcctagccttctcatccatctcccacctgggctgaataactgctatcatcctctactcccccaaactaaccctcctaaccttctacctctacgttacaataactacctcagtattcataacattcaacacaaccaaaaccataaacttatcaactataataacctcatgaactaaagcccccatacttaatacaaccctcatactaactctcctctcactagccggactcccacccctatcaggcttcctaccaaaatgactcatcctacacgaactagttaaccaagaactaacccccaaagccacaatcatcgccatactatcactcctaggactattcttctacctacgcctagcatactactcaacaatcaccctaccaccaaactcctccacccacacaaaacaatgacacaccaacaacccaacaacaacccctattgccattctcacctcaatatcaatctcactcttgccgctctccccaatgatcttagccactat

Otus_capnodes_40 -----tccacatacaaaactactcacctcactaagcctcctcctaggaacagccatcaccatctcgagcaaccactgagtaatggcctgaacagggctagaaatcaatacactagcaattatccccataatctcaaaatcacatcacccccgagccatcgaagccaccattaaatacttcctagtacaagcaaccgcctccatcatattactcttctcaagcaccacaaacgcatgatcctcaggacaatgagacatcacacaaataactcaccctatatcatgcctcatcctaacaacagccattgcaataaaactaggactagctccattccacttctgattcccggaagtcctccaaggcacgtcacttaccaccgcactcctactctcaacactaataaaactcccaccgctcactctcctcctactaacctcccactcgctaaacccagccctactaactaccatggccatctcctcaacagccctaggaggatgaataggcctaaatcaaacacaaatccgaaaaatcctagccttctcatccatctcccacttgggctgaataaccgctatcatcctctactcccccaaactaaccctcctagccttctacctctacattacaataaccacctccgtattcataacattcaacacaaccaaaaccacaaacctatcaactataataacctcatgaaccaaagctcccatactcaatacaaccctcatactaactctcctctcactagccggactcccacccctatcaggcttcctaccaaaatgacttatcctacaagaactagtcaaccaagaactaacccccgaagccacaatcattgccatactatcactcctaggattattcttctacctacgcctagcatactactcaacaatcaccctaccaccaaactcctccacccacacaaaacagtgacacaccaacaacccaacaacaacccccattgccattctcacctcaatatcaatctcactcttgccgctctccccagtgatcttaaccgctat

_Otus_madagascariensis_CHIMERA --gagcccacatacaaaactactcacctcactaagcctcctcctaggaacaaccatcactatctcgagcaaccactgagtaatggcctgaacaggactagaaatcaacacattagcaattatccccataatctcaaaatcacatcatccccgagccatcgaagccaccattaaatacttcctagtacaagcaaccgcctccatcatactactcttctcaagcatcacaaacgcatgatcctcgggacaatgagacatcacacaattaactcaccccacatcatgcctcatcctaacagcagctattgcaataaaactaggactaaccccatttcacttttgatttccggaagtcctccaaggcacatcacttaccaccgcactcctactctcaacactaataaaactaccaccactcactctcctcctactaacctcccactcactaaacccaactctactaactaccatggctatctcctcagcagccttgggaggatgaataggcctaaaccaaacacaaatccgaaaaatcctagccttctcatccatctcccacctaggctgaataactgctatcatcctctactcccccaaactaaccctcctaaccttctacctctacattacaataaccacctccgtattcataacattcaacacaactaaaaccacaaacctatcaactataataacctcatgaactaaaacccccgtacttaatacaaccctcatactaactctcctctcactagccggactcccacccctatcaggcttcctaccaaaatgacttatcttacaagaacttgttaaccaagaactaacccccaaagccacaatcattgccatattatcactcctaggattattcttctacctacgcctagcatactactcaacaatcaccctaccaccaaactcctccacccacacaaaacaatgacacaccaacaacccaacaacaactcccattgccattctcacctcaatatcaatctcactcttgccgctctccccagtgatcttagccactat

Otus_pauliani_76 atgagcccacatacaaaactactcacctcactaagcctcctcctaggaacagctatcaccatctcaagcaatcactgagtaatggcctgaacaggactagaaatcaatacactagcaattatccccataatctcaaaatcacatcacccccgagccatcgaagccgccattaaatacttcctagtacaagcaaccgcctccatcatattactcttctcaagcaccacaaacgcatgatcctcaggacaatgagacatcacacaaataactcaccctacatcatgcctcatcctaacaacagctattgcaataaaactaggactagtcccattccacttttgattcccagaagtcctccaaggcacatcacttaccaccgcactcctgctctcaacactaataaaacttccaccgctcactctcctcctactaacctcccactcactaaacccaaccctactaactaccatggccatctcctcaacagccctaggagggtgaatgggcctaaaccaaacacaaatccgaaaaatcctagccttctcatccatctcccatctaggctgaatatctgctatcatcctctactcccccaaactaaccctcctaaccttctacctctacgccacaataaccacctctgtattcataacattcaacacaactaaaaccacaaacttatcaactataataacctcatgagctaaagcccccatacttaacacaacccttatagtaaccctcctctcgctagccggactcccacccctatcaggcttcctaccaaaatgacttatcctacaagaactagtcaaccaagaactaacccccgaagccacaaccattgccatattgtcactcctaggactattcttctacctacgcctagcatactactcaacaatcaccctaccaccaaactcctccacccacacaaaacaatgacacaccaacaacccgacaacagcccccattgccattctcacctcaatatcaatctcactcttgccgctctccccggtgatcttagccactat

Otus_insularis_47 -----cccccatacaaaactactcacctcactaagcctcctcctaggaacaagcatcaccatctcgagcaaccactgagtaatagcctgaacaggactagaaatcaacacactagcaattatccccataatctcaaaatcacatcaccctcgagccatcgaagccaccattaaatacttcctagtacaagcaaccgcctccattatactactcttctcaagcaccataaacgcatgatcctcaggacaatgagacatcacacaaataacccaccccacatcatgtctcatcctaacaacagctattgcaataaaactaggactagtcccattccacttttgattcccagaagtcctccaaggtacatcacttaccaccgcactcctactctcgacactaataaaactcccaccactcaccctcctcctactaacctcccactcactaagcccaaccttactaactactatagctgtctcctcaacagccttgggaggatgaataggcctaaaccaaacacaaatccgaaaaatcctagccttctcatccatctcccacttaggctgaataactgctatcatcctctactcccctaaactaaccctcctaaccttctacctctatattacaataaccacttccgtattcataacattcaacacaactaaaaccataaacttatcaactataataacctcatgaaccaaagcccccatacttaacactaccctcatactaaccctcctctcactagccggactcccacccctatcaggcttcctaccaaaatgactcatcctacaagaactagttaaccaagaactaacccccgaagccacaatcattgccatactatcactcctaggactattcttctacctacgcctagcatactactcaacaatcaccctgccaccaaactcctccacccacataaaacaatgacacaccaacaacccaacaacaacccccattgccattctcacttcaatatcaatctcactcttgccgctctccccagtgatcttagccactat

Otus_socotranus_89 ------ccccatacaaaactacttacctcactaagcctcctcctaggaacaagcatcaccatctcgagcaaccactgagtaatagcctgaacaggactagaaatcaacacactagcaatcatccctataatttcaaaatcacatcaccctcgagccatcgaagctaccattaaatacttcctagtacaagcaaccgcctccatcatactactcttctcaagcaccataaacgcatgatcctcaggacaatgagacatcacacaaataactcaccctacatcatgtctcatcctaacaacagctattgcaataaaactaggactagttccattccacttttgattcccggaagtcctccaaggcacatcacttaccaccgcactcctactctcgacactaataaaactcccaccgctcaccctcctcctactaacctcccactcactaaacccaactttactaactactatagctgtctcctcaacagccctaggaggatgaataggcctaaaccaaacacaaatccgaaaaatcctagccttctcatccatctcccacctaggttgaataactgccatcatcctctactcccctaaactaaccctcctgaccttctacctctacatcacaataaccacttccgtattcataacattcaacacaaccaaaaccacaaacttatcaactataataacctcatgaactaaagcccccatactcaactctacccttatactaactctcctctcactagccggactcccacccctatcgggcttcctaccaaaatgatttatcctacaagaactagttaaccaagaactaacccccgaagccacaatcattgccatactatcactcctaggattattcttctacctacgcctagcatactactcaacaatcaccctaccaccaaactcctccacccacacgaaacaatgatacaccaacaacccaacaacaacccccattgccattctcacctcaatatcaatctcactcttgccgctctccccagtgatcttagccactat

_Otus_sunia_CHIMERA --gagcccccatacaaaactactcacctcactaagcctcctcctaggaacaagcatcaccatctcgagcaaccactgagtaatagcctgaacaggactagaaatcaacacactagcaatcatccccataatctcaaaatcacaccaccctcgagccattgaagccaccattaaatacttcctagtacaagcaaccgcctccatcatactactcttctcaagcaccataaacgcatgatcctcaggacaatgagacatcacacaaataactcaccccacatcatgcctcatcctaacaacagccattgcaataaaactaggactagtcccattccacttctgattcccggaagtcctccaaggcacatcacttaccaccgcactcctactctccacactaataaaactcccaccactcaccctcctcctactaacatcctactcactaaacccaaccttattaactactatagctgtctcctcaacagccctgggaggatggataggcctaaaccaaacacaaatccgaaaaatcctagccttctcatccatctcccacctaggctgaataactgctatcatcctctactcccctaaactaaccctcctaaccttctgcctttacattacaataaccacttccgtattcataacattcaacacaaccaaaaccacaaacctatcaaccttaataatctcatgaaccaaagcccccatacttaacactaccctcatactaaccctcctctcactagccggactcccacctctatcaggcttcctaccaaaatgactcatcctacaagaactagttaaccaagaactaacccctgaagccacaattatcgctatactatcactcctaggattattcttctacctacgcctaacatactactcaacaatcaccctaccaccaaactcctccacccacacaaaacaatgacacaccaataacccaacaacaacccccattgccattctcacctcaatatcaatctcactcctgccgctctccccaatgatcttagccgctat

Otus_moheliensis_CHIMERA -----ccctcatacgaaactactaacctcactaagcctcctcctaggaacaaccatcaccatctcgagcaatcactgaataatagcctgaacaggactagaaatcaatacactagcaattatccccataatctcaaaatcacatcacccccgagccatcgaagccaccattaagtgcttcctagtacaagcaaccgcctccatcatattactcttctcaagcaccataaacgcatgatcctcaggacaatgagacatcacacaaataactcatcccacatcatgcctcatcctaacaacagccattgcaataaaactaggactagttccattccacttttgattcccagaagtcctccaaggcacgtcactcaccaccgcactcctgctctcaacactattaaaactcccaccgctcactctcctcctactaacctcccattcactaaacccaaccctactagctaccatagctatctcctcaacagccctaggaggatgaataggcctaaaccaaacacaaatccgaaaaatcctagccttctcatccatctcccacctgggatgaataactgctatcatcctctactccccaaaattaaccctcctaaccttctacctctacgttacaataaccgcctccgtactcataacattcaacacaactaaaaccacaaatttatcaactataataacctcatgaactaaagcccccatactcaacacaacccttatattaactctcctctcactagccggactcccacccctatcaggcttcctaccaaaatgacttatcctacaagaactagttaaccaagaactgacccccgaagccacaatcatcgccatactgtcactcctgggattatttttctacctacgcctagcatactactcaacaatcaccctgccaccaaactcctccactcacgcaaaacaatgacacaccaacaacccaacaacaacccccattgccattctcacctcaatatcaatctcactcttgccgctctccccagtgatcttagcctctat

Otus_brucei_CHIMERA ------ccacatacaaaattactcaccacactaagcctcctcctaggaacaaccatcaccatctcgagcaatcactgagtaatagcctgaacgggattagaaatcaatacactagcaatcatccctatgatctcaaaatcacatcacccccgagccatcgaagccaccatcaaatacttcttagtacaagcaactgcctccatcatactactcttctcaagcaccataaacgcatgatcctcaggacaatgagacattacacaaataacccaccccgcatcatgcctcatcctaacaacagccattgcaataaaactaggactagtcccattccacttttgattcccagaagtcctccaaggaacatcactcaccaccgcacttttactctcaacgctaataaaactcccaccactcaccctcctcctactgacctcccactcactaaaccccaccctactaaccaccatggctatctcatcgacagccctaggaggatg---------------------------------------------------------------------------------------------------------------------------------------------------------------------------------------------------------------------------------------------------------------------------------------------------------------------------------------------------------------------------------------------------------------------------------------------------------------------------------------------------------------------------------------------------------

Otus_icterorhynchus_holerythrus_27 ------------------ctactcacttcgttaagccttctcctagggacaaccatcaccatctcaagcaatcactgagtaatagcctgaacaggattagaaatcaacacacttgcaattatccctataatctcaaaatcacaccatccccgagccatcgaagccactatcaaatacttcctagtacaagcaaccgcttccattatactactcttctcaagcactacaaacgcatggtcctcaggacaatgagacatcacccaaataactaaccccacatcatgcctcatcctaacagtagcaattgcaataaaactaggactagtcccattccacttctgatttccagaagtccttcaaggaacttcactcactaccgcactcctactctcaacactaataaaactcccaccactagccctcctcctactaacctcccactcactaaacccaaccctactaaccaccatagctatctcctcaacagccctgggaggatgaatgggcctaaaccaaac---------------------------------------------------------------------------------------------------------------------------------------------------------------------------------------------------------------------------------------------------------------------------------------------------------------------------------------------------------------------------------------------------------------------------------------------------------------------------------------------------------------------------------------

Otus_icterorhynchus_CHIMERA ---------------------------------------------gggacaaccatcaccatctcaagcaaccactgagtgatagcctgaacaggattagaaattaatacactagcaatcatccccataatctcaaaatcacaccacccccgagccatcgaggccactatcaaatacttcctagtacaagcaaccgcttccatcatactactcttctcaagcaccacaaacgcatgatcctcaggacaatgagacatcacccaaataaccaaccccacatcatgcctcatcctaacagtagcaattgcaataaagttaggactagtcccattccacttctggttcccagaagtccttcaaggaacatcacttaccaccgctctcctactctcaacactaataaaactcccaccactagccctcctcttactaacctcccactcactaaacccaaccctactaaccaccatagctatcgcctcaacagccctgggaggatgaatgggcctaaacca------------------------------------------------------------------------------------------------------------------------------------------------------------------------------------------------------------------------------------------------------------------------------------------------------------------------------------------------------------------------------------------------------------------------------------------------------------------------------------------------------------------------------------------

Otus_ireneae_49 -tgagcccgtacacaaaactgcttacctccctaagcctcctcctggggacagccatcgccatctcaagcaaccactgggcaatagcctgaacaggattagaaatcaacacactagccatcatccccctaatctcaaaatcacatcacccccgagccatcgaagcctcaatcaagtacttcctagtacaagcaactgcttcaaccctactactcttctcaagcaccaccaatgcatggtcctcaggacagtgggacatcacccagataacccaccctacgtcctgccttatcctaacatcagcaattgcaataaaattaggattagtaccattccacttctgattcccagaggtcctccaaggagcacccctcaccaccgcactcttgctctcaacgctaatgaaactccccccactcgccctacttctactaacatcccactccctgaacccaaccctgctagccaccatagctatttcatcggcggccctggggggatgaataggcttgaaccaaacacaaatccgaaaaattctagccttctcatccatctcacacttaggctgaataatcgccatcatcctctactcccccaagctaaccctcctaaccttctgcctatacactacaataatcaccaccgtattcatggcctttaacataaccaaagccacaagcttatcaacaataataatctcatgaacaaaagcccccatactgaacacaacactcatactaaccttactatcactagccggacttccccccctaacaggatttctaccaaaatgactcatcctacaagaacttaccaaccaggaactcaccctagaggccttaaccatcgctatgctatcgctcctaggactgttcttctacctacgcctagcataccacgcaacaatcaccctaccgccaaacccttcaacccacatcaaacaatgacacatcatcaaccgaactaataccaccattgccattctcacttcaatatcgatctcactcctgccactctccccaatgatatcagccactat

Otus_rutilus_78 -----cccacatacaaaactactcacctcactaagcctcctcctaggaacaaccatcactatctcgagcaaccactgagtaatagcctgaacaggactagaaatcaacacattagcaattatccccataatctcaaaatcacatcatccccgagccatcgaagccaccattaaatacttcctagtacaagcaaccgcctccatcatactactcttctcaagcatcacaaacgcatgatcctcgggacaatgagacatcacacaattaactcaccccacatcatgcctcatcctaacagcagctattgcaataaaactaggactaaccccatttcacttttggtttccggaagtcctccaaggcacatcacttaccaccgcactcctactctcaacactaataaaactcccaccactcactctcctcctactaacctcccactcactaaacccaactctactaactaccatggctatctcctcaacagccttgggaggatgaataggcctaaaccaaacacaaatccgaaaaatcctagccttctcatccatctcccacctaggctgaataactgctatcatcctctactcccccaaactaaccctcctaaccttctacctctacattacaataaccacctccgtattcataacattcaacacaactaaaaccacaaacctatcaactataataacctcatgaactaaaacccccgtacttaatacaaccctcatactaactctcctctcactagccggactcccacccctatcaggcttcctaccaaaatgacttatcttacaagaacttgttaaccaagaactaacccccaaagccacaatcattgccatattatcactcctaggattattcttctacctacgcctagcatactactcaacaatcaccctaccaccaaactcctccacccacacaaaacaatgacacaccaacaacccaacaacaactcccattgccattctcacctcaatatcaatctcactcttgccgctctccccagtgatcttagccactat

Otus_scops_scops_8 -----------------------------------------------------------------------------------------------------------------------------------------------------------------------------------------------------------------------------------------------------------------------------------------------------------------------------------------------------------------------------------------------------------------------------------------------------------------------------------------------------------------------------------------------------------------------------------------------------------------------------------------------------------------------------------------------------------------------------------------------------------------------------------------------------------------------------------------------------------------------------------------------------------------------------------------------------------------------------------------------------------------------------------------------------------------------------

Otus_scops_scops_9 -----------------------------------------------------------------------------------------------------------------------------------------------------------------------------------------------------------------------------------------------------------------------------------------------------------------------------------------------------------------------------------------------------------------------------------------------------------------------------------------------------------------------------------------------------------------------------------------------------------------------------------------------------------------------------------------------------------------------------------------------------------------------------------------------------------------------------------------------------------------------------------------------------------------------------------------------------------------------------------------------------------------------------------------------------------------------------

Otus_spilocephalus_luciae_94 -----------------------------------------------------------------------------------------------------------------------------------------------------------------------------------------------------------------------------------------------------------------------------------------------------------------------------------------------------------------------------------------------------------------------------------------------------------------------------------------------------------------------------------------------------------------------------------------------------------------------------------------------------------------------------------------------------------------------------------------------------------------------------------------------------------------------------------------------------------------------------------------------------------------------------------------------------------------------------------------------------------------------------------------------------------------------------

Otus_silvicola_87 -----------------------------------------------------------------------------------------------------------------------------------------------------------------------------------------------------------------------------------------------------------------------------------------------------------------------------------------------------------------------------------------------------------------------------------------------------------------------------------------------------------------------------------------------------------------------------------------------------------------------------------------------------------------------------------------------------------------------------------------------------------------------------------------------------------------------------------------------------------------------------------------------------------------------------------------------------------------------------------------------------------------------------------------------------------------------------

_Otus_cyprius_CHIMERA -----------------------------------------------------------------------------------------------------------------------------------------------------------------------------------------------------------------------------------------------------------------------------------------------------------------------------------------------------------------------------------------------------------------------------------------------------------------------------------------------------------------------------------------------------------------------------------------------------------------------------------------------------------------------------------------------------------------------------------------------------------------------------------------------------------------------------------------------------------------------------------------------------------------------------------------------------------------------------------------------------------------------------------------------------------------------------

Otus_elegans_CHIMERA -----------------------------------------------------------------------------------------------------------------------------------------------------------------------------------------------------------------------------------------------------------------------------------------------------------------------------------------------------------------------------------------------------------------------------------------------------------------------------------------------------------------------------------------------------------------------------------------------------------------------------------------------------------------------------------------------------------------------------------------------------------------------------------------------------------------------------------------------------------------------------------------------------------------------------------------------------------------------------------------------------------------------------------------------------------------------------

[marker: nd3; length: 385; no. of seqs: 51; new coordinates: 1038-1422)]

Bubo_bubo_111 ataaacacaatctcactcatactcgtcacctccctcaccctaagcaccatcctagtcgtattaaacttttgacttgcccaaatgaactccgaccaagaaaaactatccccatacgaatgcggatttgacccactaggctccgctcgactcccattctcagtccgattcttcctagtagccatcctgttcctcctattcgacctagaaatcgcacttctactccccctcccctgggccattcaacttcaatccccaattaccactctaacctgaaccttcgccataatcaccctcctcaccctgggcttaatctacgaatgaacacagggcggcctagaatgggccgaataa----------------------------------

Strix_aluco_129 ataaacacaatctcactcatgttcaccacctccctcatcctaagcgtcaccttaatcacactaaacctctgactcacccaaataaaccccgaccaagaaaaactatccccatacgaatgtggcttcgaccccctaggatctgcccgactcccattttcaattcgattcttcctagtagctatcctgttcctcctattcgacctagaaatcgcactcctactccccctcccatgggctattcaactacaatccccaattgccactctaacctgaaccttcatcataattaccctactcaccctaggcttaatctatgagtgaatgcaaggcggcctggaatgagctgaataa----------------------------------

Otus_bakkamoena_marathae_35 -------------------------------------------------------------------------------------------------------------------------------------------------------------------------------------------------------------------------------------------------------------------------------------------------------------------------------------------------------------------------------------------------

_Otus_lempiji_CHIMERA ataaacacaatctcacttatactcttaacctccctaaccttaagcacaatcctaatcatactaaacttctgactcgctcaaacttacaccgaccaagaaaaactaaccccatacgaatgcggcttcgaccctctagggtctgcccgactaccattctcagtacgattcttcctagtagctatcttattcctcctatttgacttagaaatcgcacttctacttcccctcccatgagccactcaactacaatccccaactaccaccctaacctgaaccttcaccatacttaccctgctcaccctcggcctaatctacgaatgaatccaagggggcctagaatgggcagaataa----------------------------------

Otus_lettia_lettia_CHIMERA ataaacacaatctcacttatactcttaatctccctaaccttaagcacaatcttaatcatactaaacttctgactcgctcaaacttacaccgaccaagaaaaactatccccatacgaatgcggcttcgaccccctaggatctgcccgactaccattctcagtacgattcttcctagtagctatcttattcctcctatttgacttagaaatcgcacttctacttcccctcccatgagccactcaactacaatccccaactaccaccctaacctgaaccttcaccatacttaccctgctcaccctcggcctaatctacgaatgaatccaaggaggcctagaatgggcagaataa----------------------------------

Otus_megalotis_CHIMERA ---------atctcactcatatttctaatctccctgaccctaagcacaattctaatcacactaaacttctgactcgcccaaacctacactgaccaagaaaaactatccccatacgagtgtggctttgaccccctaggatccgcccgactaccattctcagtacgattcttcctagtagctatcttattcctcctattcgatttagaaatcgcacttctactccccctcccatgagccacccaactacaatccccaaccactaccctaacctgaaccttcactgtaatcactctacttaccctaggcctaatttatgaatgaattcaggggggcctagaatgggcagaataa----------------------------------

Otus_nigrorum_CHIMERA -------------------------------------------------------------------------------------------------------------------------------------------------------------------------------------------------------------------------------------------------------------------------------------------------------------------------------------------------------------------------------------------------

Otus_everetti_CHIMERA -------------------------------------------------------------------------------------------------------------------------------------------------------------------------------------------------------------------------------------------------------------------------------------------------------------------------------------------------------------------------------------------------

Otus_semitorques_CHIMERA ataaacacaatctcactcatatttttaatctccctgaccctaagtacaattctaatcacactaaacttctgactcgcccaaacctacactgatcaagaaaaactatccccatacgaatgtggctttgaccccctaggatctgcccgactgccattctcagtacgattcttcctagtagctatcttattcctcctattcgacttagaaatcgcacttctactccccctcccatgagccacccaactacaatccccaaccaccaccttggcctgaaccttcactataatcaccctgcttaccctaggcctaatttatgaatgaatccaagggggcttagagtgggcagaataa----------------------------------

Otus_angelinae_29 -------------------------------------------------------------------------------------------------------------------------------------------------------------------------------------------------------------------------------------------------------------------------------------------------------------------------------------------------------------------------------------------------

Otus_spilocephalus_vandewateri_97 -------------------------------------------------------------------------------------------------------------------------------------------------------------------------------------------------------------------------------------------------------------------------------------------------------------------------------------------------------------------------------------------------

Otus_spilocephalus_vulpes_96 -------------------------------------------------------------------------------------------------------------------------------------------------------------------------------------------------------------------------------------------------------------------------------------------------------------------------------------------------------------------------------------------------

Otus_spilocephalus_spilocephalus_90 -------------------------------------------------------------------------------------------------------------------------------------------------------------------------------------------------------------------------------------------------------------------------------------------------------------------------------------------------------------------------------------------------

Otus_spilocephalus_hambroecki_92 -------------------------------------------------------------------------------------------------------------------------------------------------------------------------------------------------------------------------------------------------------------------------------------------------------------------------------------------------------------------------------------------------

_Otus_spilocephalus_latouchi_CHIMERA ataaacacaatttctctcatattcttaatctccctaaccctaagcacaattctaattatactgaacttctgactcgcccaagtccaaaccgaccaagaaaaactatccccatacgaatgcggctttgaccccctaggatctgcccggctaccattctcagtccgattcttcctagtagccatcctattcctcctatttgacctagaaattgcactcctgctccccctcccatgagccactcaactacaatcccccaccaccaccctaacttgaaccttcaccatgctcaccctactcaccctaggcctaatctatgaatgaatccaagggggcctagaatgagcagaataa----------------------------------

Otus_hartlaubi_13 ataaacacaatctccctcatactcctaatctccctagccctaagcacaattctaattatactaaatttcggactcgcccaaatccacactgaccaagaaaagctatccccatatgaatgtggctttgaccctctaggatctgctcggctaccattctcagtccgattcttcctagtagctatcctattcctcctattcgacctagaaattgcactcttactccccctcccatgagccacccagctacaatccccagccactaccctaacatgaacctccgtcataattaccctactcaccctaggcctgatctatgaatgaattcaagggggcctagaatgagccgaataa----------------------------------

Otus_hartlaubi_15 -----------------catactcctaatctccctagccctaagcacaattctaattatactaaatttcggactcgcccaaatccacactgaccaagaaaagctatccccatatgaatgtggctttgaccctctaggatctgctcggctaccattctcagtccgattcttcctagtagctatcctattcctcctattcgacctagaaattgcactcttactccccctcccatgagccacccagctacaatccccagccactaccctaacatgaacctccgtcataattaccctactcaccctaggcctgatctatgaatgaattcaagggggcctagaatgagccgaataaccagaaagttagtctaactaagacagttgatttc

Otus_hartlaubi_16 ----------tctccctcatactcctaatctccctagccctaagcacaattctaattatactaaatttcggactcgcccaaatccacactgaccaagaaaagctatccccatatgaatgtggctttgaccctctaggatctgctcggctaccattctcagtccgattcttcctagtagctatcctattcctcctattcgacctagaaattgcactcttactccccctcccatgagccacccagctacaatccccagccactaccctaacatgaacctccgtcataattaccctactcaccctaggcctgatctatgaatgaattcaagggggcctagaatgagccgaataaccagaaagttagtctaactaagacagttgatttc

Otus_hartlaubi_14 -------------------------------------------gcacaattctaattatactaaatttcggactcgcccaaatccacaccgaccaagaaaagctatccccatatgaatgtggctttgaccccctaggatctgctcggctaccattctcagtccgattcttcctagtagctatcctattcctcctattcgacctagaaattgcactcttactccccctcccatgagccacccagctacaatccccagccactaccctaacatgaacctccgtcataattaccctactcaccctaggcctgatctatgaatgaattcaagggggcctagaatgagccgaataaccagaaagttagtctaactaagacagttgatttc

Otus_feae_45 ----------------------------------------------------------------------gactcgcccaaatccacgccgaccaagaaaaactatccccatatgaatgtggctttgaccctctaggatccgctcgactaccattctcagtccgattcttcctagtagccatcctattcctcctattcgacctagaaattgcactcttacttcccctcccatgagccacccaactacaatccccagccactaccctaacatgaacttctgccataattaccctactcaccctaggcctgatctatgaatgaattcaagggggcctagaatgagccgaataa----------------------------------

Otus_senegalensis_17 ataaacacaatctccctcatactcctaatctccctcgccctgagcacaattctaattatactaaatttcggactcgcccaaatccacgccgaccaagaaaaactatccccatatgaatgtggctttgaccctctaggatccgctcgactaccattctcagtccgattcttcctagtagccatcctattcctcctattcgacctagaaattgcactcttacttcccctcccatgagccacccaactacaatccccagccactaccctaacatgaacttctgccataattaccctactcaccctaggcctgatctatgaatgaattcaagggggcctagaatgagccgaataa----------------------------------

Otus_pamelae_74 -taaacacaatctccctcatactcctaatctccctagccctaagcacaattctaattatactaaatttcggactcgcccaaatccataccgaccaagaaaaactatccccatacgaatgtggctttgaccctctaggatccgctcgacttccattctcagtccgattcttcctagtagctatcctattcctcctattcgacctagaaattgcactcctactccccctcccatgagccacccaactacaatccccaaccactaccctaacatgaacttctgttataatcaccctgctcaccctaggcctaatctacgaatgaattcaagggggcctagaatgagccgaatag----------------------------------

Otus_sp_nov_1 -----------ctccctcatactcctaatctccctagccctaagcacaattctaatcatgctaaatttcggactcgcccaaatccacaccgaccaagaaaagctatctccatatgaatgcggctttgaccctctaggatccgctcgactaccattctcagtccgattcttcctagtagccatcctattcctcctattcgacctagaaattgcactcttactccccctcccatgagccacccaactacaatccccaaccactaccctaacatgaacctccatcataattaccctgctcaccctaggcctgatctatgaatgagttcaggggggcctagaatgagccgaataaccagaaagttagtctaactaagacagttgatttc

Otus_sp_nov_2 -----------ctccctcatactcctaatctccctagccctaagcacaattctaatcatgctaaatttcggactcgcccaaatccacaccgaccaagaaaagctatctccatatgaatgcggctttgaccctctaggatccgctcgactaccattctcagtccgattcttcctagtagccatcctattcctcctattcgacctagaaattgcactcttactccccctcccatgagccacccaactacaatccccaaccactaccctaacatgaacctccatcataattaccctgctcaccctaggcctgatctatgaatgagttcaggggggcctagaatgagccgaataaccagaaagttagtctaactaagacagttgatttc

Otus_sp_nov_3 -----------ctccctcatactcctaatctccctagccctaagcacaattctaatcatgctaaatttcggactcgcccaaatccacaccgaccaagaaaagctatctccatatgaatgcggctttgaccctctaggatccgctcgactaccattctcagtccgattcttcctagtagccatcctattcctcctattcgacctagaaattgcactcttactccccctcccatgagccacccaactacaatccccaaccactaccctaacatgaacctccatcataattaccctgctcaccctaggcctgatctatgaatgagttcaggggggcctagaatgagccgaataaccagaaagttagtctaactaagacagttgatttc

Otus_sp_nov_4 -----------ctccctcatactcctaatctccctagccctaagcacaattctaatcatgctaaatttcggactcgcccaaatccacaccgaccaagaaaagctatctccatatgaatgcggctttgaccctctaggatccgctcgactaccattctcagtccgattcttcctagtagccatcctattcctcctattcgacctagaaattgcactcttactccccctcccatgagccacccaactacaatccccaaccactaccctaacatgaacctccatcataattaccctgctcaccctaggcctgatctatgaatgagttcaggggggcctagaatgagccgaataaccagaaagttagtctaactaagacagttgatttc

Otus_scops_5 ataaacacaatctccctcatactccttatctccctagccctaagcacaattctaattatactaaatttcggactcgcccaaatccgcactgaccaagaaaaactatccccatacgaatgtggctttgaccctctaggatccgctcgactaccattctcagtccgattcttcctagtagccatcctattcctcctattcgacctagaaattgcactcctactccccctcccatgggccacccaactacaatctccagccactaccctaacatgaacttctgttataattaccctactcaccctaggcctaatctatgagtgaactcaagggggcctagaatgagccgaataa----------------------------------

_Otus_scops_JF5337_F ataaacacaatctccctcatactccttatctccctagccctaagcacaattctaattatactaaatttcggactcgcccaaatccgcactgaccaagaaaaactatccccatacgaatgtggctttgaccctctaggatccgctcgactaccattctcagtccgattcttcctagtagccatcctattcctcctattcgacctagaaattgcactcctactccccctcccatgggccacccaactacaatctccagccactaccctaacatgaacttctgttataattaccctactcaccctaggcctaatctatgagtgaactcaagggggcctagaatgagccgaataa----------------------------------

Otus_pembaensis_24 ataaacacaatctccctcatactcctaatctccctagccctaagcacgattctaattatgctaaatttcggactcgcccaaacccacaccgaccaagaaaaactatccccatatgaatgtggctttgaccccctaggatccgctcgactaccattctcagtacgattcttcctagtagccatcctattcctcctattcgacctagaaattgcactcttactccccctcccatgagctacccaactacaatccccagccaccaccctaacatgaacttttgtcataattaccctactcaccctaggcctgatctatgaatgaattcaagggggcctagaatgagccgaatag----------------------------------

Otus_pembaensis_25 ataaacacaatctccctcatactcctaatctccctagccctaagcacgattctaattatgctaaatttcggactcgcccaaacccacaccgaccaagaaaaactatccccatatgaatgtggctttgaccccctaggatccgctcgactaccattctcagtacgattcttcctagtagccatcctattcctcctattcgacctagaaattgcactcttactccccctcccatgagctacccaactacaatccccagccaccaccctaacatgaacttttgtcataattaccctactcaccctaggcctgatctatgaatgaattcaagggggcctagaatgagccgaatag----------------------------------

_Otus_longicornis_CHIMERA ataaacacaatctccctcatactcctaatctccctagccctaagcacagctttaatcacactaaacttctgacttgcccaaatccacaccgaccaagaaaaactatccccatacgaatgtggctttgaccccctaggatcagcccgactaccattctcagtccgattcttcctagtagccatcctattcctcctgttcgacctagaaattgcactcctactccccctcccatgagccacccaactacaatctccaacctctaccctgacatgaactgccatcataatcaccctactcaccctaggcctaatctacgaatgaattcaaggaggcctcgaatgagctgaataa----------------------------------

Otus_mirus_CHIMERA ataaacacaatctccctcatactcctaatctccctagccctaagcacagctctaattacactaaacttctgacttgcccaaatccacaccgaccaagaaaaactatccccatacgaatgtggcttcgaccccctaggatcagcccgactaccattctcagtccgattcttcctagtagctatcctattcctcctgttcgacctagaaattgcactcctactccccctcccatgagccacccaactacaatctccaacctctaccctagcatgaactaccatcataattaccctgcttaccctaggcctaatctatgaatgaattcaaggaggcctcgaatgagctgaataa----------------------------------

Otus_mayottensis_60 ataaacacaatctccctcatactcctaatctccctagccctaagcacgattctaatcacactaaacttctgacttgcccaaattcacaccgaccaagaaaaactatccccatacgaatgcggctttgaccccctaggatcagcccgactcccattctcagtccgattcttcctagtagccatcctattcctcctattcgacctagaaattgcactcctgctccccctcccatgagccacccaactacaatctccaacctctaccctaacatgaactgccattatactcaccctacttactctgggcctaatctatgagtgaattcaagggggcctagagtgagctgaataa----------------------------------

Otus_capnodes_40 ataaacacaatctccctcatactcctaatctccctagccctaagcacaattctaatcacactaaacttctgacttgcccaaacccacaccgaccaagaaaaactatccccatacgaatgtggcttcgaccccctgggatcagcccgactaccattctcagtccgattcttcctagtagccatcctattcctcctattcgacctagaaattgcgctcctactcccccttccatgagccacccaactacaatctccaacctctaccctaacatgaactgccattataatcaccctgcttaccatgggcctaatctatgaatgaattcaaggaggcctagaatgagccgaataa----------------------------------

_Otus_madagascariensis_CHIMERA ataaacacaatctccctcatactcctaatctccctagccctaagcacaattctaatcacactaaacttctgacttgcccaaatccacaccgaccaagaaaaactatccccgtatgaatgtggctttgaccccctgggatcagcccgactaccattctcagtccgattcttcctagtagccatcctattccttctatttgacctggaaattgcactcttactccccctcccatgagccacccaactacaatctccaacctctaccctagcatgaactgccattataatcaccctgcttaccctgggcctaatctatgaatgaattcaagggggcctagaatgagccgaataa----------------------------------

Otus_pauliani_76 ataaacacaatatccctcatactcctaatctccctagccctaagcataattctaatctcactaaacttctgatttgcccaaatccacaccgaccaagaaaaactgtccccatatgaatgcggctttgaccccctgggatcagcccgactaccattctcagtccgattcttcctagtagccatcctattcctcctattcgacctagaaattgcacttctacttcccctcccatgagccacccaactacaatctccaacctctaccctagcatgaactgccattataatcactctacttaccctaggcctaatctatgaatgaattcaagggggcctagagtgggccgagtaa----------------------------------

Otus_insularis_47 ataaacacaatctccctcatactcctaatctccacagccctaagcacaattctaatcgcactaaacttctgacttgcccaaacccataccgaccaagaaaaactatccccatacgaatgtggcttcgaccccctaggatctgcccgactaccattctcaatccgattcttcctagtagccatcctattcctcctattcgatctagaaattgcactcctactccccctcccatgagccacccaactacaatctccaacctctaccctaacatgaactgccattataatcaccctgcttaccctaggcctaatctatgaatgaattcagggaggcctagagtgagccgaatag----------------------------------

Otus_socotranus_89 -taaacacaatctccctcatactcctaatctccacagccctaagtacaatcctaatcgcattaaacttctgacttgcccaaatccataccgaccaagaaaaactatccccatacgagtgtggcttcgaccccctaggatctgctcgactaccattctcagtccgattcttcctagtagccatcctattcctcctattcgatctagaaattgcactcctactccccctcccatgagccacccaactacaatctccaacctctaccctaacatgaactgccattataattaccctgcttaccctaggcctaatctatgaatgaattcaaggaggcctagaatgagccgaataa----------------------------------

_Otus_sunia_CHIMERA ataaacacaatctccctcatactcctaatctccacagccctaagcacaattctaatcgtactaaacttctgacttgcccaaatccataccgaccaagaaaaactatccccatacgaatgtggcttcgaccccctaggatctgcccgactgccattctcagtccgattcttcctagtagccatcctattcctcctgttcgatctagaaattgcactcctactccccctcccatgagccacacaactacaatctccaacctctaccctaacatgaactgccatcataatcaccctgcttaccttaggcctaatctatgaatgaattcaagggggcttagagtgagccgaataa----------------------------------

Otus_moheliensis_CHIMERA ------------tccttcatactcctaatctccctagccctaagcacaattctaatcacactaaacttctgactcgcccaaatccgcaccgaccaagaaaaactatccccatacgaatgtggctttgaccccctaggatcagcccgactaccattctcagtccgattcttcctagtagctatcctattcctcctattcgacctagaaattgcactcctactccccctcccatgagctacccaactacaatctccaacctctaccctaacatgaactgccatcataatcactctgcttaccctgggcctggtctatgaatgaattcaagggggcctagagtgagccgaataaccagaaagttagtctaactaagacagttgatttc

Otus_brucei_CHIMERA -taaacacaatctccctcatactcctaatctccctagccttaagcacaggtctaattacactaaacttctgactcgcccaaacccacaccgaccaagaaaaactatccccatacgaatgcggctttgaccccctagggtcagctcgactaccattctcagttcgattcttcctagtagcaatcctattcctcctattcgacctagaaattgcactcctactccccctcccatgagccacccagctacaatccccaaccactaccctaacatgaacttccattataattaccctactcaccctaggcctaatctatgaatgaattcaagggggcttagaatgagccgaataa----------------------------------

Otus_icterorhynchus_holerythrus_27 ------------------atactcctagtctccttagccttaagcacaattctaatcgcactaaacttctgactcgcccaaattcgcactgaccaagaaaaactatccccatacgaatgcggcttcgaccctctgggatcagcccggctaccattctcagtccggttcttcctagtagccatcctattccttctattcgacttggaaattgctctcctactccccctcccatgagctacccaactacaatccccaaccaccaccttaacctgaacttccattatactcgccctactcaccctaggcctaatctacgaatgaattcaagggggcctagaatgggccgaataaccagaaagttagtctaactaagacagttgatttc

Otus_icterorhynchus_CHIMERA -------------------------------------ccctaagcacaattctaatcgcactaaacttctgactcgcccaaattcacaccgaccaagaaaaactatccccatacgaatgcggcttcgaccctctggggtcagcccggctgccattctcagtccgattcttcctagtagccatcctattccttctattcgacttggaaattgctctcctactccccctcccatgagctactcaactacaatccccaaccaccaccctaacctgaacttccattatactcgccctactcaccctgggcctaatttacgaatgaattcaaggaggcctagaatgagccgaataaccagaaagttagtctaaccaagacagttgatttc

Otus_ireneae_49 ataaacacaatcacactcatattctcagtctccctgaccctaagcgcaattctaatcacactaaacctctgactcgcccaagtaaacactgaccaagaaaagctatccccatacgaatgtggcttcgaccccctgggatccgctcgactcccattctcagttcgattcttcctagtagccattctattcctcctgtttgacctagagatcgcgcttctactcccccttccatgagccacccagctacaatccccaaccaccacactaacctgaaccttcaccataattgccctcctcaccttaggcctcacctacgaatgaacccaaggaggcctagaatgggccgaataa----------------------------------

Otus_rutilus_78 ataaacacaatctccctcatactcctaatctccctagccctaagcacaattctaatcacactaaacttctgacttgcccaaatccacaccgaccaagaaaaactatccccgtatgaatgtggctttgaccccctgggatcagcccgactaccattctcagtccgattcttcctagtagccatcctattccttctatttgacctggaaattgcactcttactccccctcccatgagccacccaactacaatctccaacctctaccctagcatgaactgccattataatcaccctgcttaccctgggcctaatctatgaatgaattcaagggggcctagaatgagccgaataa----------------------------------

Otus_scops_scops_8 ataaacacaatctccctcatactccttatctccctagccctaagcacaattctaattatactaaatttcggactcgcccaaatccgcactgaccaagaaaaactatccccatacgaatgtggctttgaccctctaggatccgctcgactaccattctcagtccgattcttcctagtagccatcctattcctcctattcgacctagaaattgcactcctactccccctcccatgggccacccaactacaatctccagccactaccctaacatgaacttctgttataattaccctactcaccctaggcctaatctatgagtgaactcaagggggcctagaatgagccgaataaccagaaagttagtctaactaagacagttgatttc

Otus_scops_scops_9 --------------------------------------------cacaattctaattatactaaatttcggactcgcccaaatccgcactgaccaagaaaaactatccccatacgaatgtggctttgaccctctaggatccgctcgactaccattctcagtccgattcttcctagtagccatcctattcctcctattcgacctagaaattgcactcctactccccctcccatgggccacccaactacaatctccagccactaccctaacatgaacttctgttataattaccctactcaccctaggcctaatctatgagtgaactcaagggggcctagaatgagccgaataaccagaaagttagtctaactaagacagttgatttc

Otus_spilocephalus_luciae_94 -------------------------------------------------------------------------------------------------------------------------------------------------------------------------------------------------------------------------------------------------------------------------------------------------------------------------------------------------------------------------------------------------

Otus_silvicola_87 -------------------------------------------------------------------------------------------------------------------------------------------------------------------------------------------------------------------------------------------------------------------------------------------------------------------------------------------------------------------------------------------------

_Otus_cyprius_CHIMERA -------------------------------------------------------------------------------------------------------------------------------------------------------------------------------------------------------------------------------------------------------------------------------------------------------------------------------------------------------------------------------------------------

Otus_elegans_CHIMERA -------------------------------------------------------------------------------------------------------------------------------------------------------------------------------------------------------------------------------------------------------------------------------------------------------------------------------------------------------------------------------------------------

[marker: atp6; length: 684; no. of seqs: 51; new coordinates: 1423-2106)]

Bubo_bubo_111 atgaacttaagcttcttcgaccaattcataagcccccacctcataggaatccccctcaccctactctcactactacttccaaccctactcctccccgcaccaaacaaccggtgagtcactaatcgcctatctacccttcagctatgactcctaaacaccatcacaaaacaactaataaccccactaaacaaaaaagcccacaaatgggccctaatcctaacctccctaataacaatacttcttgcaatcaacctcctaggccttctaccgtacacattcacccctaccacccagctctccataaacatagcactagccttcccactatggctagctaccctacttacgggcctacgaaaccaaccttcaatctccatcggacacctcctaccagaaggcacccccacaccactaatcccagccctaatcctaattgaaactaccagcctcctcatccgccccttagccctaggtgtccgcctaacagcaaacctgacagcaggacacctccttattcaactcatctcaactgccgttatagccctcctaccaatcataccagtgatatccactctaaccgcaataatcctattcctcctaaccatcctagagatcgcagtagccataatccaagcctacgtctttgttctcttattaaccctatatctacaagaaaacatctaa

Strix_aluco_129 atgaacctaagcttcttcgaccaattcataagtccctgcctcataggaatcccccttactctactctcaatactacttccaactttactactccccgcaccaaacaaccgatgagtaccgaaccgtctatctaccatccaactatgacttctaaacaccatcacaaaacaactaataaccccattaaataaaaaagctcacaaatgagccctaatcctaacttccctgataacaatactcctcatgatcaaccttctaggactactaccttacacattcacccccaccacccaactgtccataaacatagcactagccttcccactctgactagccaccctgctcacaggcctgcgaaatcaaccctcaatttccctaggacacctcttgccagaaggtaccccaacaccactaatcccagccctaatcctaatcgaaacaactagcctcctcatccgccccctagccctaggtgtccgcctaacggcgaacctaacagcaggacacctcctcattcaactcatctcaacagccactatagccctcctgccgatcttaccagtagtctccgccttaactgcgataatcctactcctccttaccatcctagaggtcgcagtggctataatccaagcatatgtctttgtcctcctattaagtctgtacttacaagaaaacatctaa

Otus_bakkamoena_marathae_35 ------------------------------------------------------------------------------------------------------------------------------------------------------------------------------------------------------------------------------------------------------------------------------------------------------------------------------------------------------------------------------------------------------------------------------------------------------------------------------------------------------------------------------------------------------------------------------------------------------------------------------------------------------------------------------------------------------------

_Otus_lempiji_CHIMERA atgaacctaagcttcttcgaccaattcataagcccttgcctactgggagtacccctcaccttactatcagtcctatttccaacactcctactaccagcaccaaacaaccgatgaatcactaaccgcttatctactctacaactctgattcttaaacaccaccacaaaacaactactaaccccattaaacaaaaagggccacaagtgagccctagtcctaacctccctaataacaatgttactaataatcaacctactgggcctgttaccatacacattcacccctaccacccaactatccataaacatagcactggccttcccactctgaatagccaccctccttacaggcctacgaaatcaaccttcaacctccctaggacacctcctgccagaaggcaccccaacaccactaatcccagccctcattctgatcgaaacaaccagcctactcatccgccctttagccctaggagtccgcttaacagcaaacctaacagcaggacacctactaattcaactcatctcgactgccgtcatggcccttcttcccgtcctcccagcagtgtctgccctaaccgcaataatcctactccttctcactatcctagaggtcgcagtagccataattcaagcatacgtatttgtcctcctactaaccttatacttacaagagaacatctaa

Otus_lettia_lettia_CHIMERA atgaacctaagcttcttcgaccaattcataagcccttgcctactgggagtacccctcaccttactatcaatcctatttccaatactcctactaccagcaccaaacaaccgatgaatcactaaccgcttatccactctacaactctgactcttaaacaccaccacaaaacaactactaaccccattaaacaaaaaaggccacaaatgagccctagtcctaacctccctaataacaatgttactaataatcaacctactgggcctgttaccatacacattcacccctaccacccaactatccataaacatagcactggccttcccactctgaatagccaccctccttacaggcctacgaaaccaaccttcaacctccctaggacacctcctgccagaaggcaccccaacaccactaatcccagccctcattctaatcgaaacaaccagcctactcatccgccccttagccctaggagtccgcttaacagcaaacctaacagcaggacacctactaattcaactcatctcgactgccgtcatggcccttcttcccgtcctcccagcagtgtctgccctaaccgcaataatcctactccttctcactatcctagaggtcgcagtagccataattcaagcatacgtatttgtcctcctactaaccttatacttacaagagaacatctaa

Otus_megalotis_CHIMERA atgaacttaagctttttcgaccaattcataagcccatgccttttaggagtacccctcaccctactatcaatcctattcccagctctcctactaccaacacctaacaaccgatgaatcaccaaccgcttatccaccctacaactatgattcttaaacaccactacaaaacaactactaaccccattaaacaaaaaaggccacaaatgagccctaatcctaacctccctaataacgatacttctgacaatcaatctactgggcctactaccatatacattcacccccaccacccaactatccataaacatggcactagccttcccactctgaatagccaccctcctcacaggcctacgaaaccaaccttcaacctccttaggacacctcctgccagaaggtacccccacaccactaatccctgcccttattctaatcgaaacaactagcctactcatccgtcccctagctctaggagtccgcctaactgcaaacctaacagcagggcacctactaatccaactcatctcaacagccgttatagcccttctcccaatcctaccagcagtatctgccctaactgcaataatcttacttcttcttaccatcctagaagtcgcagtagctataatccaagcatacgtatttgttctcctactaaccctatacttacaagaaaacatctaa

Otus_nigrorum_CHIMERA ------------------------------------------------------------------------------------------------------------------------------------------------------------------------------------------------------------------------------------------------------------------------------------------------------------------------------------------------------------------------------------------------------------------------------------------------------------------------------------------------------------------------------------------------------------------------------------------------------------------------------------------------------------------------------------------------------------

Otus_everetti_CHIMERA ------------------------------------------------------------------------------------------------------------------------------------------------------------------------------------------------------------------------------------------------------------------------------------------------------------------------------------------------------------------------------------------------------------------------------------------------------------------------------------------------------------------------------------------------------------------------------------------------------------------------------------------------------------------------------------------------------------

Otus_semitorques_CHIMERA atgaacttaagcttcttcgaccaattcatgagcccatgccttctaggagtacccctcaccctactatcagttctattcccagctctcctactaccagcacccaataaccgatgaatcaccaaccgtttatctaccctacaattatgattcttaaacacgactacaaaacaactactaaccccactaaacaaaaaaggccataaatgagccctaatcctaacctccctaataacaatactcctaataatcaacctactaggcctgctaccatacacattcacccccaccacccaactatccataaacatagcactagccttcccactctgaatagccaccctcctcacaggcctgcgaaaccaaccttcaacctccctaggacacctcctgccagaaggcaccccaacaccactaatcccagccctaattttaatcgaaacaaccagcctactcattcgccccctagccttaggagtccgcctaacagcaaacctaacagcagggcacctactaatccaactcatttcaacagccgttatagccctcctcccaatcctaccagcagtatctgccctaactgctataatcttactccttcttaccatcttagaggtcgcagtagccataatccaagcatatgtatttgttctcctactaaccttatacttacaagaaaacatctaa

Otus_angelinae_29 ------------------------------------------------------------------------------------------------------------------------------------------------------------------------------------------------------------------------------------------------------------------------------------------------------------------------------------------------------------------------------------------------------------------------------------------------------------------------------------------------------------------------------------------------------------------------------------------------------------------------------------------------------------------------------------------------------------

Otus_spilocephalus_vandewateri_97 ------------------------------------------------------------------------------------------------------------------------------------------------------------------------------------------------------------------------------------------------------------------------------------------------------------------------------------------------------------------------------------------------------------------------------------------------------------------------------------------------------------------------------------------------------------------------------------------------------------------------------------------------------------------------------------------------------------

Otus_spilocephalus_vulpes_96 ------------------------------------------------------------------------------------------------------------------------------------------------------------------------------------------------------------------------------------------------------------------------------------------------------------------------------------------------------------------------------------------------------------------------------------------------------------------------------------------------------------------------------------------------------------------------------------------------------------------------------------------------------------------------------------------------------------

Otus_spilocephalus_spilocephalus_90 atgaacctaagcttcttcgaccaattcataagcccatgcctcctaggagtgcccctcactctactatcaatactatttccgaccctactactcccggcacctaacaaccgatgagtcaccaatcgcctatccaccctacaactatgactcctaaacaccaccacaaaacaactactaacccccttaaacaaaaagggacacaaatgagccctggtcctaacctccctaataacaatattactaataattaacctactaggcctactaccatacacattcacccctactactcaactatccataaatatggcactggccttcccactctgaatagccaccctccttacaggcctacgaaatcagccctcaacctccctagggcatctactgccagaaggcaccccaaccccactaattccagccctaatcctaatcgaaacaaccagcctactcatccgccccctagccctaggagtccgcctaacagcaaacctaacagcaggacacctactgattcaactcatctcaacagccgtcatggccctccttccaatcctaccagcagtatctgccctcaccgcaataatcctgctccttcttaccgtcctagaggtcgcagtagccataatccaagcatacgtctttgtcctcctactaaccctatacttacaagaaaacatctaa

Otus_spilocephalus_hambroecki_92 ------------------------------------------------------------------------------------------------------------------------------------------------------------------------------------------------------------------------------------------------------------------------------------------------------------------------------------------------------------------------------------------------------------------------------------------------------------------------------------------------------------------------------------------------------------------------------------------------------------------------------------------------------------------------------------------------------------

_Otus_spilocephalus_latouchi_CHIMERA atgaacctaagctttttcgaccaattcataagcccatgcctcctaggagtacccctcaccctactatcagtactattcccaaccttactactcccagcacccaacaaccgatgaattaccaaccgcctatccaccctacaactatgactcctaaacaccaccacaaaacaactgttaacccccttaaacaaaaagggacataagtgggccctaatcctaacctccctaataacaatactactaataattaacctactaggcctactaccatatacattcacccccactactcaactatccataaacatggcactggctttcccactctgaatagccaccctccttacaggtctacgaaatcagccctcaacctctctaggacacctactgccagaaggcaccccaaccccactaattccagccctaatcctaatcgaaacaaccagcctactcatccgccccctggccctgggagtccgcctaacagcaaacctaacagcaggacacctgctgatccaactcatctcaacagccgtcatggccctccttccaatcctaccagcagtatctgccctcaccgcaataatcctgctccttctcactattctagaggtcgcagtagccataatccaagcatacgtctttgtcctcctactaaccctatacttacaagaaaacatctaa

Otus_hartlaubi_13 atgaacctaagcttctttgatcaattcataagcccctgcttccttggagtacccctcatcctactatcaatactattcccaaccctcctgctcccagcaccaaacaaccgatgaatcaccaaccgcctatccacccttcaactatgactcctaaacaccattacaaaacaactgctaaccccactagacaagaagggccacaaatgagccataatcctaacctccctaataatgatactactgataattaacctcctaggccttctaccatacacatttacccccaccacccaactatccataaacatagcactagccttcccactctgaatagctaccctactcacaggcttacgaaaccaaccttcaatctccctaggccacctcttacctgaaggcactcccactccactaatccccgccctaatcctaatcgaaactaccagcctactaattcgccccctagccctcggagtccgtctaaccgcaaacctaacagcaggacaccttctaatccaactcatctcaacagctgtcatggccctactaccaatcctaccaacagtatcagccctaactgcaataatcctcctcctactcaccatcctagaagttgcagtagccataatccaggcatacgtattcgtcctcctactaagcctatacttacaagaaaacatctaa

Otus_hartlaubi_15 ------------------------------------------------------------------------------------------------------------------------------------------------------------------------------------------------------------------------------------------------------------------------------------------------------------------------------------------------------------------------------------------------------------------------------------------------------------------------------------------------------------------------------------------------------------------------------------------------------------------------------------------------------------------------------------------------------------

Otus_hartlaubi_16 -----------------------------------------------------------------------------------------------------------ccgatgaatcaccaaccgcctatccacccttcaactatgactcctaaacaccattacaaaacaactgctaaccccactagacaagaagggccacaaatgagccataatcctaacctccctaataatgatactactgataattaacctcctaggccttctaccatacacatttacccccaccacccaactatccataaacatagcactagccttcccactctgaatagctaccctactcacaggcttacgaaaccaaccttcaatctccctaggccacctcttacctgaaggcactcccactccactaatccccgccctaatcctaatcgaaactaccagcctactaattcgccccctagccctcggagtccgtctaaccgcaaacctaacagcaggacaccttctaatccaactcatctcaacagctgtcatggccctactaccaatcctaccaacagtatcagccctaactgcaataatcctcctcctactcaccatcctagaagttgcagtagccataatccaggcatacgtattcgtcctcctactaagcctatacttacaagaaaacatctaa

Otus_hartlaubi_14 ------------------------------------------------------------------------------------------------------------------------------------------------------------------------------------------------------------------------------------------------------------------------------------------------------------------------------------------------------------------------------------------------------------------------------------------------------------------------------------------------------------------------------------------------------------------------------------------------------------------------------------------------------------------------------------------------------------

Otus_feae_45 ------------------------------------------------------------------------------------------------------------------------------ctatccacccttcaactatgactcctaaacaccattacaaaacaactgctaaccccactagacaagaagggccacaaatgagccataatcctaacctctctgataacaatactactgataatcaacctcctaggccttctaccatacacatttacccccaccacccaactatccataaacatagcactagccttcccactctgaatagccaccctactcacaggcttacgaaaccaaccttcaatctccctaggccaccttttacccgaaggcactcccacgccactaatccccgccctaatcctaatcgaaaccaccagcctactaattcgccccctagccctcggagtccgtctaaccgcaaacctaacagcaggacaccttctaatccaactcatctcaacagctgtcatagccctactaccgatcctaccaacagtatcagccctaactgcaataatcctcctcctactcaccatcctagaagttgcagtagccataatccaggcatacgtattcgtcctcctactaagcctttacttacaagaaaacatctaa

Otus_senegalensis_17 atgaacctaagcttcttcgatcaattcataagcccttgcttccttggagtacccctcatcctactatcaatactatttccaaccctcctgctcccagcaccaaacaaccgatgaatcaccaaccgcctatccactcttcaactatgactcctaaacaccattacaaaacaactgctaaccccactagacaagaagggccacaaatgagccataatcctaacctctctgataacaatactactgataatcaacctcctaggccttctaccatacacatttacccccaccacccaactatccataaacatagcactagccttcccactctgaatagccaccctactcacaggcttacgaaaccagccttcaatctccctaggccaccttttacctgaaggcacccccactccactaatccccgccctaatcctaatcgaaaccaccagcctactaattcgccccctagccctcggagtccgtctaaccgcaaacctaacagcaggacaccttctaatccaactcatctcaacagctgtcatagccctactaccgatcctaccaacagtatcagccctaactgcaataatcctcctcctactcaccatcctagaagttgcagtagccataatccaggcatacgtattcgtcctcctactaagcctatacttacaagaaaatatctaa

Otus_pamelae_74 ------------------------------------------------------------------------------------------------------------------------------------------------------------------------------------------------------------------------------------------------------------------------------------------------------------------------------------------------------------------------------------------------------------------------------------------------------------------------------------------------------------------------------------------------------------------------------------------------------------------------------------------------------------------------------------------------------------

Otus_sp_nov_1 ------------------------------------tgcttccttggagtacccctcatcctactatcaatactattcccaaccctcctgctcccagcaccaaacaaccgatgaatcaccaaccgcctatccacccttcaactatgactcctaaacaccactacaaaacaactgctaaccccactagacaagaagggccacaaatgagccataatcctaacctccttgataacaatactactgataatcaacctcctaggccttctaccatacacatttacccccaccacccaactatccataaacatagcactagccttcccactctgaatggctactctactcacaggcttacgaaaccaaccttcaatctccctagggcacctcttacctgaaggcactcccactccactaatccccgccctaatcctaatcgaaaccactagcctactaattcgccccctagccctcggagtccgcttaaccgcaaacctaacagcaggacatcttctaatccaactcatctcaacagctgtcatagccctactaccaatcctaccaacagtatcagccctaactgcaataatcctcctcctactcaccatcctagaagttgcagtagccataatccaggcatacgtattcgtcctcttactaagcctgtacttacaagaaaacatctaa

Otus_sp_nov_2 -------------------------------------------------tacccctcatcctactatcaatactattcccaaccctcctgctcccagcaccaaacaaccgatgaatcaccaaccgcctatccacccttcaactatgactcctaaacaccactacaaaacaactgctaaccccactagacaagaagggccacaaatgagccataatcctaacctccttgataacaatactactgataatcaacctcctaggccttctaccatacacatttacccccaccacccaactatccataaacatagcactagccttcccactctgaatggctactctactcacaggcttacgaaaccaaccttcaatctccctagggcacctcttacctgaaggcactcccactccactaatccccgccctaatcctaatcgaaaccactagcctactaattcgccccctagccctcggagtccgcttaaccgcaaacctaacagcaggacatcttctaatccaactcatctcaacagctgtcatagccctactaccaatcctaccaacagtatcagccctaactgcaataatcctcctcctactcaccatcctagaagttgcagtagccataatccaggcatacgtattcgtcctcttactaagcctgtacttacaagaaaacatctaa

Otus_sp_nov_3 ------------------------------------------------------------------------------------------------------------------------------------------------------------------------------------------------------------------------------------------------------------------------------------------------------------------------------------------------------------------------------------------------------------------------------------------------------------------------------------------------------------------------------------------------------------------------------------------------------------------------------------------------------------------------------------------------------------

Otus_sp_nov_4 -------------------------------------------------------------------------------------------------------------------------------------------------------------------------------------------------------------------------------------------------------------------------------------------------------tatccataaacatagcactagccttcccactctgaatggctactctactcacaggcttacgaaaccaaccttcaatctccctagggcacctcttacctgaaggcactcccactccactaatccccgccctaatcctaatcgaaaccactagcctactaattcgccccctagccctcggagtccgcttaaccgcaaacctaacagcaggacatcttctaatccaactcatctcaacagctgtcatagccctactaccaatcctaccaacagtatcagccctaactgcaataatcctcctc------------------------------------------------------------------------------------------

Otus_scops_5 atgaacctaagcttctttgaccaattcataagcccctgcttcctcggagtacccctcatcctactatctgtactattcccaaccctcctgctcccagcaccaaacaaccgatgaatcaccaaccgcctatccacccttcaactatgactcctaaacaccactacaaaacaactactaaccccactagacaagaagggccacaaatgagctataatcctaacctccctgatgacgatactactgataatcaacctcctaggccttctaccatacacatttaccccgactacccaactatccataaacatagcactagccttcccactctgaatggctaccctactcacaggcttacgaaaccaaccctcaatctccctaggacacctcttacccgaaggcacccccactccactaatccccgccctaatcctaatcgaaaccaccagcctactaattcgccccctagccctcggagtccgtctaaccgcaaacctaacagcaggacaccttctaatccaactcatctcaacagctgtcatagccctactaccgatcctaccaatagtatcagccctaactgcaataatcctcctcctactcaccatcctagaggttgcagtagccataatccaggcatacgtattcgttctcctactaagcctatacttacaagaaaacatctaa

_Otus_scops_JF5337_F atgaacctaagcttctttgaccaattcataagcccctgcttcctcggagtacccctcatcctactatctgtactattcccaaccctcctgctcccagcaccaaacaaccgatgaatcaccaaccgcctatccacccttcaactatgactcctaaacaccactacaaaacaactactaaccccactagacaagaagggccacaaatgagctataatcctaacctccctgatgacgatactactgataatcaacctcctaggccttctaccatacacatttaccccaactacccaactatccataaacatagcactagccttcccactctgaatggctaccctactcacaggcttacgaaaccaaccctcaatctccctaggacacctcttacccgaaggcacccccactccactaatccccgccctaatcctaatcgaaaccaccagcctactaattcgccccctagccctcggagtccgtctaaccgcaaacctaacagcaggacaccttctaatccaactcatctcaacagctgtcatagccctactaccgatcctaccaatagtatcagccctaactgcaataatcctcctcctactcaccatcctagaggttgcagtagccataatccaggcatacgtattcgttctcctactaagcctatacttacaagaaaacatctaa

Otus_pembaensis_24 ---------------------------ataagcccttgcttccttggagtacccctcatcctactatcagtactattcccaaccctcctgctcccagcaccaaacaaccgatgagtcaccaaccgcctatccacccttcaactatgactcctaaacaccattacaaagcaactgctaaccccactagacaagaagggccacaaatgagccataatcctaacctccctgataacaatactactgataatcaacctcctaggccttctaccatacacatttacccccaccacccaactatccataaacatagcactagccttcccactctgaatagctaccctactcacaggtttacgaaaccaaccttcaatctccctaggccacctcttacctgaaggcacccccactccactaatccccgccctaatcctaatcgaaaccaccagcctactaattcgccccctagccctcggagtccgtctaaccgcaaacctaacagcaggacaccttctaatccaactcatctcaacagctgtcatagccctactaccgatcctaccaacagtatcagccctaactgcaataatcctcctcctactcaccatcctagaagttgcagtagccataatccaggcatacgtattcgtcctcctactaagcctgtacttacaagaaaacatctaa

Otus_pembaensis_25 ---------------------------ataagcccttgcttccttggagtacccctcatcctactatcagtactattcccaaccctcctgctcccagcaccaaacaaccgatgagtcaccaaccgcctatccacccttcaactatgactcctaaacaccattacaaagcaactgctaaccccactagacaagaagggccacaaatgagccataatcctaacctccctgataacaatactactgataatcaacctcctaggccttctaccatacacatttacccccaccacccaactatccataaacatagcactagccttcccactctgaatagctaccctactcacaggtttacgaaaccaaccttcaatctccctaggccacctcttacctgaaggcacccccactccactaatccccgccctaatcctaatcgaaaccaccagcctactaattcgccccctagccctcggagtccgtctaaccgcaaacctaacagcaggacaccttctaatccaactcatctcaacagctgtcatagccctactaccgatcctaccaacagtatcagccctaactgcaataatcctcctcctactcaccatcctagaagttgcagtagccataatccaggcatacgtattcgtcctcctactaagcctgtacttacaagaaaacatctaa

_Otus_longicornis_CHIMERA atgaacctaagcttcttcgaccaattcataagcccctgcttccttggagtgcccctcatcctgctatcaatactattcccaaccctcctgctcccagcaccaaacaaccgatgaatcaccaaccgtctatccacccttcaactatgactcctaaacaccattacaaaacaactactaaccccattagacaaaaagggccacaaatgagctgtaatcctaacctccctaataacaatactactaacaattaacctcctaggcctactaccatatacatttacccccaccacccaactatccataaacatagcactagccttcccactctgaatagccaccctactcacaggcctacggaaccaaccttcaatctccctaggacacctcttacccgaaggcacccctaccccactaatccccgccctaattctaatcgaaacaaccagcctactcatccgccccctagccctaggagtccgtctaaccgcaaacctaacagcaggacaccttctaatccaactcatctcaacagctgtcatggccctgctacccatcctgccaatagtatcagccctgactgcaataatcctcctcctactcaccatcctagaggtcgcagtagccataatccaggcatacgtattcgtcctcctactaagcctatatttacaagaaaacatctaa

Otus_mirus_CHIMERA ------------------------------------------------------------------------------------------------------------------------------------------------------------------------------------------------------------------------------------------------------------------------------------------------------------------------------------------------------------------------------------------------------------------------------------------------------------------------------------------------------------------------------------------------------------------------------------------------------------------------------------------------------------------------------------------------------------

Otus_mayottensis_60 atgaacctaagcttcttcgaccaattcataagcccctgcttccttggagtacccctcatcctactatcagtactattcccagccctcctgctcccagcaccaaacaaccgatgaatccccaaccgcctatccactcttcaactatgactcctaaacaccattacaaaacaactactaaccccactagataaaaaaggccacaaatgagccataatcctaacctccctaataacaatgcttctaacaatcaacctcctaggcctgctaccatatacatttacccccaccacccaactatccataaacatagcactagccttcccactctgaatagccaccctactcacaggcctacgaaaccaaccttcaatctccctaggacacctcttacctgaaggaacccctaccccactaatccccgccctgattctaatcgaaaccaccagcctactcattcgccccctagctttaggagtccgcctaactgcaaacctaacagcaggacacctcctaatccaactcatctcaacagccgtcatagccctactacccatcctaccaatagtatcagtcctgactgcaataatcctactcctactcacaatcctagaaatcgcagtagccataatccaggcatacgtattcgtcctcctactaagcctatacttacaagaaaacatctaa

Otus_capnodes_40 atgaacctaagctttttcgaccaattcataagcccctgcttccttggagtacccctcatcctgctatcaatactattcccaaccctcctgctcccagcaccaaacaaccgatgaatcaccaaccgcctatccactctacaactatgactcctaaacaccactacaaaacaactactaaccccactagataaaaaaggccacaaatgagctataatcttaacctccctaataacaatgctactgacaatcaacctcctaggcctactaccatatacattcacccccaccacccaactatccataaacatagcactagccttcccactctgaatagctactctactcacaggcttacgaaaccaaccttcaatctccctaggacacctcttacctgagggaacccctaccccactaatccccgccctaattctaatcgaaactaccagcctactcattcgccccctagccctaggagtccgcctaactgcaaacctaacagcaggacaccttctaatccaactcatctcaacagccgtcatagccttactacccatcctaccagtagtatcagccctgactgcaataatcctactcctactcaccatcctagaggtcgcagtagccataatccaggcatacgtattcgtcctcctactaagcttatacttacaagaaaacatctaa

_Otus_madagascariensis_CHIMERA atgaacctaagcttcttcgaccaattcataagcccctgcttccttggagtacccctcatcctactatcagtactatttccagccctcctgctcccagcaccaaacaaccgatgagtcaccaaccgcctatccacccttcaactatgactcctaaacaccattacaaaacaactactaaccccattagataagaaaggccacaaatgagctataatcctaacttccctaataataatgctactaacaatcaacctcctgggcctactaccatatacatttacccccaccacccaactatccataaacatagcactagccttcccactctgaatagccaccctactcacaggcttacgaaatcaaccttcaatctccctaggacacctcctacctgaaggaacccccaccctactaatccccgccctaattctaatcgaaactaccagcctactcattcgccccctagccttaggagtccgcctaactgcaaacctaacagcaggacaccttctaatccaactcatctcaacagccgttatagctctactacccatcctaccaatagtatcggccctgactgcaataatcctactcctactcaccgtcctagaggtcgcagtagctataatccaggcatatgtattcgtcctcctactgagcctatacttacaagaaaacatctaa

Otus_pauliani_76 ------------------------------------------------------------------------------------------------------------------------------------------------------------------------------------------------------------------------------------------------------------------------------------------------------------------------------------------------------------------------------------------------------------------------------------------------------------------------------------------------------------------------------------------------------------------------------------------------------------------------------------------------------------------------------------------------------------

Otus_insularis_47 atgaacctaagcttcttcgaccaattcataagcccctccttccttggagtacccctcatcctactatcaatactattcccagccctactgctcccaataccaaacaaccgatgaatcaccaaccgcctatccaccctccagctatgactcctaaacaccattacaaaacaactactaaccccattaaataagaaaggccacaaatgagccataatcctaacctccctaataactatgctactgacaatcaacctcctaggcttactaccgtatacatttacccccaccacccaactatccatgaacatagcactagccttcccactctgaatggccaccctactcacaggcttacgaaaccaaccttcaatctccctaggacacctcctacctgaaggaacccccaccccactaatccccgccctaattctaatcgaaactaccagcctactcattcgccccctagccttaggggtccgtctaactgcaaatcttacagcaggacacctcttaatccaactcatctcaacagccgtcatagccctactacccgtcctaccagtagtatcagccctgactgcaataatcctactcctactcaccatcctagaggtcgcagtagccataatccaggcatacgtattcgtcctcctactaagcctatacttacaagaaaacatctaa

Otus_socotranus_89 ------------------------------------------------------------------------------------------------------------------------------------------------------------------------------------------------------------------------------------------------------------------------------------------------------------------------------------------------------------------------------------------------------------------------------------------------------------------------------------------------------------------------------------------------------------------------------------------------------------------------------------------------------------------------------------------------------------

_Otus_sunia_CHIMERA atgaacctaagcttcttcgaccaattcataagcccctgcttccttggagtacccctcatcctactatcaatactattcccagccctgctgctcccgacaccaaacaaccgatgaatcaccaaccgcctatccaccctccaactatgactcctaaacaccattacaaaacaactactaaccccattaaataagaaaggccacaaatgagccataatcctaacctccctaataactatgttactgacaatcaacctcctaggcttactaccgtatacatttacccctaccacccaactatccatgaacatagcactagccttcccactctgaatagccaccctactcacaggcttacgaaaccaaccttcaatctccctaggacacctcctacctgaaggaacccccaccccactaatccccgccctaattctaatcgaaactaccagtctactcattcgccccctagccttaggagtccgtctaaccgcaaatcttacagcagggcacctcttaatccaactcatctcaacagccgtcatagccctactacccatcctaccagtagtatcagccctaactgcaataatcctactcctactcaccatcctagaggtcgcagtagccataatccaggcatacgtatttgtcctcctactaagcctatacttacaagaaaacatctaa

Otus_moheliensis_CHIMERA ------------------------------------------------------------------------------------------------------------------------------------------------------ctaaacaccattacaaaacaactactaaccccattaaataacaaaggccacaaatgagctataatcctaacctcacttataacaatgctactaacaatcaacctcctaggcctcctaccatacacattcacccccaccacccaactatccataaacatagcactagccttcccactctgaatagccaccctactcacaggcttacgaaaccaaccttcaatctccctaggacacctcttacctgaaggaacccctaccccactaatccccgccttaattctaatcgaaactaccagcctactcattcgccccctagccttaggagtccgcctaactgcaaacctaacagcaggacacctcctaatccaactcatctcaacagccgttatagccctactacccatcctaccaatagtatcagccctgactgcaataatcctactcctactcaccatcctagaggtcgcagtagccataatccaagcatacgtattcgtcctcttactaagcctatacttacaagaaaacatctaa

Otus_brucei_CHIMERA ------------------------------------------------------------------------------------------------------------------------------------------------------------------------------------------------------------------------------------------------------------------------------------------------------------------------------------------------------------------------------------------------------------------------------------------------------------------------------------------------------------------------------------------------------------------------------------------------------------------------------------------------------------------------------------------------------------

Otus_icterorhynchus_holerythrus_27 ------------------------------------------------------------------------------------------------------------------------------------------------------------------------------------------------------------------------------------------------------------------------------------------------------------------------------------------------------------------------------------------------------------------------------------------------------------------------------------------------------------------------------------------------------------------------------------------------------------------------------------------------------------------------------------------------------------

Otus_icterorhynchus_CHIMERA ------------------------------------------------------------------------------------------------------------------------------------------------------------------------------------------------------------------------------------------------------------------------------------------------------------------------------------------------------------------------------------------------------------------------------------------------------------------------------------------------------------------------------------------------------------------------------------------------------------------------------------------------------------------------------------------------------------

Otus_ireneae_49 atgaacctaagcttcttcgaccaattcacaagcccctgcctcctcggagtgcctctcatcctactctcaatgctatttccaaccctactactcccagcccaaaacaatcgatggatcactaaccgcctatccaccctacaactctggttcctaaacaccactacaaagaaactaataactccccttgacaaaaagggccacaagtgagccctaatcttaacctccctaatgacaatactactaataatcaacctcctaggcctgctaccatacacatttacccccaccacccaactatccataaacctagcactggcctttccactctgaatagccaccctacttacaggcctgcgaaaccagccttcaatctcattaggacacctcctgccagaaggcacccccaccccactcatcccagctctaattatgatcgaaaccaccagccttctcattcgccctctggccctcggagttcgactcacagccaacctaacagcaggacacctcctaattcaacttatctcaacagccatcatagccctactgccagtcctaccaatagtatctgccctaactgcgataattctactcctactaaccctactagagatcgcagttgccataatccaggcatacgtgttcgtcctccttctaagcctatacttacaagaaaacatctaa

Otus_rutilus_78 atgaacctaagcttcttcgaccaattcataagcccctgcttccttggagtacccctcatcctactatcagtactatttccagccctcctgctcccagcaccaaacaaccgatgagtcaccaaccgcctatccacccttcaactatgactcctaaacaccattacaaaacaactactaaccccattagataagaaaggccacaaatgagctataatcctaacttccctaataataatgctactaacaatcaacctcctgggcctactaccatatacatttacccccaccacccaactatccataaacatagcactagccttcccactctgaatagccaccctactcacaggcttacgaaatcaaccttcaatctccctaggacacctcctacctgaaggaacccccaccctactaatccccgccctaattctaatcgaaactaccagcctactcattcgccccctagccttaggagtccgcctaactgcaaacctaacagcaggacaccttctaatccaactcatctcaacagccgttatagctctactacccatcctaccaatagtatcggccctgactgcaataatcctactcctactcaccgtcctagaggtcgcagtagctataatccaggcatatgtattcgtcctcctactgagcctatacttacaagaaaacatctaa

Otus_scops_scops_8 ------------------------------------------------------------------------------------------------------------------------------------------------------------------------------------------------------------------------------------------------------------------------------------------------------------------------------------------------------------------------------------------------------------------------------------------------------------------------------------------------------------------------------------------------------------------------------------------------------------------------------------------------------------------------------------------------------------

Otus_scops_scops_9 ------------------------------------------------------------------------------------------------------------------------------------------------------------------------------------------------------------------------------------------------------------------------------------------------------------------------------------------------------------------------------------------------------------------------------------------------------------------------------------------------------------------------------------------------------------------------------------------------------------------------------------------------------------------------------------------------------------

Otus_spilocephalus_luciae_94 ------------------------------------------------------------------------------------------------------------------------------------------------------------------------------------------------------------------------------------------------------------------------------------------------------------------------------------------------------------------------------------------------------------------------------------------------------------------------------------------------------------------------------------------------------------------------------------------------------------------------------------------------------------------------------------------------------------

Otus_silvicola_87 ------------------------------------------------------------------------------------------------------------------------------------------------------------------------------------------------------------------------------------------------------------------------------------------------------------------------------------------------------------------------------------------------------------------------------------------------------------------------------------------------------------------------------------------------------------------------------------------------------------------------------------------------------------------------------------------------------------

_Otus_cyprius_CHIMERA ------------------------------------------------------------------------------------------------------------------------------------------------------------------------------------------------------------------------------------------------------------------------------------------------------------------------------------------------------------------------------------------------------------------------------------------------------------------------------------------------------------------------------------------------------------------------------------------------------------------------------------------------------------------------------------------------------------

Otus_elegans_CHIMERA ------------------------------------------------------------------------------------------------------------------------------------------------------------------------------------------------------------------------------------------------------------------------------------------------------------------------------------------------------------------------------------------------------------------------------------------------------------------------------------------------------------------------------------------------------------------------------------------------------------------------------------------------------------------------------------------------------------

[marker: cytb2; length: 1143; no. of seqs: 51; new coordinates: 2107-3249)]

Bubo_bubo_111 ---------------------------------------------------------------------------------------------------ttcggatccctactaggaatctgcctaacagtacagatcattactggcctcctcctcgccgcacactacacagctgactcaaccctagcctttgcatccgtatcacacacctgccgaaacgtacaatacggctgattactccgcaacctccatgcaaacggggcctcattcttttttatctgcatctacctgcatatcggacgtggcctatactacggctcctacctatacaaagaaacatgaaacacaggtgttatcctcctactcacccttatagcaactgccttcgtaggctacgttctcccgtgaggccaaatatcattctgaggggcaaccgtcatcaccaacctattctcggccatcccctacatcggccaaaccctcgtggaatgggcctgaggtggattctcagtagacaacccaacactgacccgatttttcgccctacacttcctcctccccttcttaattgcaagtctcacactcatccacctcaccttcctccacgactcaggctcaaacaacccactaggaatcacatcagactgcgacaaaatcccattccacccctacttctctataaaagacaccctaggacttgcactaatgctcctccccctaacaaccttagccttattctcacccaccctactcggcgacccagaaaactttacaccagcaaaccccctagtcacccctccccacatcaaaccagaatggtacttcctatttgcctacgccattttacgctcaatccccaataaactaggaggagtactagctctagccgcttccgtactgatcttactcttagtcccactcctccacaaatccaaacaacgtgcaatgaccttccgcccactctcccaatttctcttctgaaccctagctgccaacctactaatcctaacatgagtaggcagccaaccagtagaacacccatttatcattatcggccagctggcctccattacctacttcacaacaatcctcatccttttccctacagttagcaccctagaaaacaaactactcaactac---

Strix_aluco_129 ----------------------------------------------------------------------------------------------------------------------tctgcttagtaacccaaatcatcaccggcctcctccttgccgcccactatacagccgattccgccctagccttcacgtccgtatcacatacctgccgaaatgtacagtacggttgactactccgtaacctccatgcaaacggagcctcactcttcttcatctgtgtctacctacacatcggacgaggcctatactatggctcctacctgtacaaagaaacctgaaacacaggtgtcattcttttactgacccttatagcaaccgccttcgtaggctacgtcctcccatgaggccaaatatcattctgaggggcaaccgtcattactaatctgttttcagccatcccctatatcggccagaccctcgtagaatgagcctggggcggattctcagtagacaatccaacactaacccgattcttcgccctacacttcctcctccccttcataattgcaggcctnacactanttcacctaaccttcctccacgactcaggctcaaacaaccccctagggatctcatcggactgtgacaaaatcccattccacccctacttctctataaaagacaccctaggactggcattaatactaccacccctaataaccctagctctattcacgcctaacctgcttggcgacccagaaaattttacaccagcaaaccctctaatcaccccaccccacattaaaccagagtggtacttcctgtttgcctacgccatcctacgctcaatcccgaacaaactaggaggagtacttgcactagctgcctccgtactaatcctactcttaattcccctcctccataagtccaaacaacgcacaataaccttccgcccactctcccaactccttttctgaactctagtcactaacctcctaattctaacatgagtagg----------------------------------------------------------------------------------------------------------------------------

Otus_bakkamoena_marathae_35 ---------------------------------------------------------------------------------------------------------------------------------------------------------------------------------------------------------------------------------------------------------------------------------------------------------------------------------------------------------------------------------------------------------------------------------------------------------------------------------------------------------------------------------------------------------------------------------------------------------------------------------------------------------------------------------------------------------------------------------------------------------------------------------------------------------------------------------------------------------------------------------------------------------------------------------------------------------------------------------------------------------------------------------------------------------------------------------------------------------------------------------------------------------------------------------------

_Otus_lempiji_CHIMERA ---------------------------------------------------------------------------------------------------ttcggatccctcctaggaatctgccttataacccaaatcatcactggccttctcctagccgcacactacacagccgactcaaccctagctttctcatccgtagcacacacctgccgaaatgtacaatacggctgactactccgaaacctccatgcaaacggagcctcattcttcttcatctgcatctaccttcatatcggacgaggcctctactatggctcctacctctacaaagaaacctgaaacacaggtgttatcctactactgaccctcatagccaccgccttcgtaggatatgtcctgccatgaggccaaatgtcattctgaggggctaccgtcatcactaacctattctcagctgtaccatacatcggacagacccttgtagaatgagcctgaggcggattctcagtagacaacccaacactgactcggttctttgccctccacttcctccttccattcatcattgcaggactaacactcatccacctcactttccttcacgaatcaggctcaaacaaccctctagggatcacatcagactgcgacaaaatcccattccacccctacttttcaataaaagacacactaggcctagcgttactactactacccctaacaaccctggccttattctcacctaacctcctaggagaccccgaaaacttcacacctgctaatccactagttacccctccccatatcaaaccagaatgatacttcctgtttgcctacgccatcctacgctcaatccctaacaaactagggggtgtactagcactcgccgcctccgtattagtcttattcttaattcctctactgcataaatccaaacaacgagcaataaccttccgcccactttcccaactcctattctgagtactaactactaacctactgattctaacatgagtaggcagccaaccagtagaacatcccttcat-------------------------------------------------------------------------------------------------

Otus_lettia_lettia_CHIMERA -----------------------------------------------------------------------------------------------------------------------ctgcttagtaacccaaatcatcactggccttctcctagccgcacactacacagccgactcaaccctagctttctcatccgtagcacacacctgccgaaatgtacaatacggctgactactccgaaacctccatgcaaacggagcctctttcttcttcatctgcatctaccttcatatcggacgaggcctctactatggctcctacctctacaaagaaacctgaaacacaggtgttatcctactactaaccctcatagccaccgccttcgtaggatatgtcttaccatgaggccaaatgtcattctgaggggctaccgtcatcactaacctattctcagctgtaccatacatcggacagacccttgtagaatgagcctgaggcggattttcagtagacaacccaacactgacccggttctttgccctccacttcctcctccccttcatcattgcaggactaacactcatccacctcaccttcctccacgaatcaggctcaaacaaccctctagggatcacatcanactgcgacaaaatcccattccacccctacttttcaataaaagacacactaggcctagcgttactactactacccctaacaaccctggccttattctcacctaacctcctaggagaccccgaaaacttcacacctgctaacccactagttacccctccccatatcaaaccagaatgatacttcctgtttgcctacgccatcctacgctcaatccccaacaaactaggaggtgtactagcactcgccgcctccgtattagtcttattcctaattcctctactgcataaatccaaacaacgagcaataaccttccgcccactttcccaaatcctattctgactactaactactaacctactgattctaacatgagtagg----------------------------------------------------------------------------------------------------------------------------

Otus_megalotis_CHIMERA ----------------------------------------------------------------------------------------------------------------------tctgcctggcaacccaaatcatcaccggccttctcctagctgcacactacacagccgattcaactctagccttctcatccgtagcccacacctgccgaaatgtacaatatggctgactactccgaaacctccatgcaaacggagcctcattcttcttcatctgcatctaccttcacatcggacgaggcctctactatggttcctacctctacaaagaaacctgaaacacaggtgtcatcctattactaaccctcatagccaccgccttcgtcggatacgtcctaccatgaggtcaaatatccttttgaggtgctactgtcatcaccaacctattctcagctgtaccttacatcggacaaaccctcgtagaatgagcctgaggtggattctcagtagataacccaacactaanccgattcttcgcyytacacttcctcctcccattcatnnttgcaggcttaacactcattcaccttaccttcctccacgaatcaggctcaaacaacccactcggaatctcatcagactgtgacaaaatcccattccacccctacttctcaataaaggacacactaggcctagcattactactattacccctaacaaccttagccttattctcacctaacctccttggagaccctgaaaacttcacaccagctaatccactagtcactcccccccacatcaaaccagaatgatacttcctatttgcctacgccatcctacgctcaatccccaacaaactaggaggggtactagcacttgccgcttccgtactagtcctattcttaatccccctactacacaaatccaaacaacgagcaataaccttccgcccactctcccaactcctattctgaatactaaccgccaacctacttattctaacatgggtggg----------------------------------------------------------------------------------------------------------------------------

Otus_nigrorum_CHIMERA ------------------------------------------------------------------------------------------------------------------------------------caaatcatcaccggccttctcctagctgcacattacacagccgattcaaccctagccttctcatccgtagctcacacctgccgaaatgtacaatatggctgactactccgaaacctccatgcaaacggagcctcattcttcttcatctgcatctaccttcacatcggacgaggcctctactacggctcctacctctacaaagaaacctgaaatacaggcgtcatcctgctactgaccctcatagccaccgccttcgtaggatatgtcctaccatggggtcaaatatcattttgaggagccactgtcatcaccaacctgttctcagctgtaccttacatcggacaaaccctcgtagaatgagcttggggcggattctcagtagacaacccgacactgacccgattcttcgccctccacttccttctcccattcatcattgcaggcttaacactcattcaccttaccttcctccacgagtcaggctcaaacaacccactcggaatctcatcagactgtgacaaaatcccattccacccctacttctcaacaaaggacatactaggcctagcattactactattacccctaacaaccttagccttattttcacctaacctccttggagaccccgaaaactttacaccagctaatccactagtcactcccccccatatcaaaccagaatgatacttcctatttgcctatgccatcctacgctcaatccccaacaaattaggaggagtactggcactcgccgcttccgtactagtcctattcttaatccccctactacacaaatccaaacaacgagcaataaccttccgcccactctcccaactcctattctgaatactaactgccaacctacttattttaacatgagtaggtagccaaccagtagaacaccccttcattattatcggacaactagcctcaaccacctacttcacaacaatcctcatcctctttcccgcaatcagcaccctagagaacaaactactcaactac---

Otus_everetti_CHIMERA ------------------------------------------------------------------------------------------------------------------------------------ccaatcatcaccggccttctcctagctgcacactacacagccgactcaaccctagccttctcatccgtagcgcacacttgccgaaatgtacaatatggctgactactccgaaacctccatgcaaacggagcctcattcttcttcatctgcatctaccttcacatcggacgaggcctctactatggctcctacctctacaaagaaacctgaaacacaggtgtcatcctattactgaccctcatagccaccgccttcgtaggatacgtcctaccatgaggtcaaatatcattttgaggggctactgtcatcaccaatctgttctcagctgtaccttatatcggacaaaccctcgtagaatgagcctggggtggattctcagtagataacccaacactaacccgattcttcgccctccatttcctcctcccattcatcattgcaggcttaacactcattcaccttaccttcctccacgaatcaggctcaaacaacccactcggaatctcatcagactgtgacaaaatcccattccacccctacttctcaataaaggacatactaggtctagcactactactattacccctaacaaccttagccttattttcacctaacctccttggagaccccgaaaacttcacaccagctaacccactagtcactcccccccatatcaaaccagagtgatacttcctatttgcctacgccatcctacgctcaatccccaacaaattaggaggagtactggcacttgccgcttccgtactagtcctattcttaatccccctactacacaaatccaaacaacgagcaataaccttccgtccactctcccaactcctattctgaatactaactgccaacctactcattctaacatgagttggtagccagccagtagaacaccccttcattattatcggacaactagcctcaattacctacttcacaacaatcctcatcctctttcccacaatcagcaccctagagaacaaactactcaactac---

Otus_semitorques_CHIMERA ----------------------------------------------------------------------------------------------------------------------tctgcttagtaacccaaatcatcaccggccttctcttagctgcacactacacagccgattcaaccctagccttctcatctgtagctcacacctgccgaaatgtacagtacggctgactactccgaaacctccatgcaaacggagcctcattcttcttcatctgcatctacctccacatcggacggggcctctactacggctcctacctctacaaagaaacctgaaatacaggggtcatcctactactaaccctcatagccaccgccttcgtaggatatgttctaccatgaggtcagatatcattctgaggggctactgttattaccaacctgttctcagccgtaccctacatcggacaaaccctcgtagaatgagcctgaggtggattctcagtagacaacccaacactaacccgattcttcgccctccacttcctcctccccttcatcattgcaggcttaacactcattcaccttaccttccttcacgaatcaggctcaaacaacccactcggagtctcatcagactgcgacaaaatcccattccacccttacttttcagcaaaagatacactaggcctagcattactactactacctctgacaaccctagccttattttcaccaaacctccttggagaccccgaaaacttcacaccagctaaccctctaatcactcccccccatatcaaaccagagtggtacttcctatttgcctacgccatcctacgctcaatccctaacaaactaggaggagtactggcacttgccgcctccgtactagtcctattcttaatccccctgctacacaaatccaaacagcgagcaataaccttccgccctctctcccaactcctattctgaacactaaccgccaacctacttatccttacgtgagtagg----------------------------------------------------------------------------------------------------------------------------

Otus_angelinae_29 ---------------------------------------------------------------------------------------------------------------------------------------------------------------------------------------------------------------------------------------------------------------------------------------------------------------------------------------------------------------------------------------------------------------------------------------------------------------------------------------------------------------------------------------------------------------------------------------------------------------------------------------------------------------------------------------------------------------------------------------------------------------------------------------------------------------------------------------------------------------------------------------------------------------------------------------------------------------------------------------------------------------------------------------------------------------------------------------------------------------------------------------------------------------------------------------

Otus_spilocephalus_vandewateri_97 ---------------------------------------------------------------------------------------------------ttcggatccctccttggaatctgcctaacaacccaaattattacaggccttctcctagccgcacactacacagccgactcaaccctggccttctcatctgtagcacacacctgccgaaacgttcaatatggctgactactccgaaacctccatgcaaacggagcctcactcttcttcatctgcatctacctccacatcggacgaggcctctactatggatcttacctctacaaggaaacctgaaacacaggtgtcatcctactgctaaccctcatagccaccgcatttgtggggtatgtcctgccatggggccaaatatcattttggggggctaccgtcatcactaacctattctcggccgtaccctacatcggtcagacccttgtagaatgagcctgaggtggattttcagtagacaaccccacactaacccgattcttcgccctccacttcctcctcccctttatcattgcagggctaacactcatccaccttaccttcctccacgaatcaggctctaataacccactcggaatcacatcagactgcgacaaaatcccattccacccatacttctccacaaaagactcactgggcctagtactccttctactacccctaacaaccctagccctattctcacccaacctcctcgga---------------------------------------------------------------------------------------------------------------------------------------------------------------------------------------------------------------------------------------------------------------------------------------------------------------------------------------------------------------------------------------------------

Otus_spilocephalus_vulpes_96 -------------------------------------------------------------------------------------------------------------------------------------------------------------------------------------------------------------------------------------------------gaaacctccatgcaaacggagcctcattcttcttcatctgcatctacctccacatcggacgaggcctctactacggatcttatctctacaaagaaacctgaaacacaggtgtcatcctactgctaaccctcnnnnnnnnnnnnnnnnnnnnnnnnnnnnnnnnnnnnnnnnnnnnnnnnnnnnnnnnnnnnnnnnnnnnnnnnnnnnnnnnnnnnnnnnnnnnnnnnnnnnnnnnnnnnnnnnnnnnnnnnnnnnnnnnnnnnnnnnnnnnnnnnnnnnnnnnnnnnnnnnnnnnnnnnnnnnnnnnnnnnnnnnnnnnnnnnnnnnnnnnnnnnnnnnnnnnnnnnnnnnnnnnnnnnnnnnnnnnnnnnnnnnccaataacccactcggaatcgcatcagactgcgacaaaatcccattccacccatacttctccataaaagactcactgggcctagtactccttctactgcccctaacaaccctagccctattctcacccaacctcctcgga---------------------------------------------------------------------------------------------------------------------------------------------------------------------------------------------------------------------------------------------------------------------------------------------------------------------------------------------------------------------------------------------------

Otus_spilocephalus_spilocephalus_90 ----------------------------------------------------------------------------------------------------------------------tctgcttagnaacccaaatcatcactggccttctcctagccgcgcactacacagccgactcaaccctagccttctcatccgtagcacacacctgccgaaacgtccaatacggctgactacttcgaaacctccatgcaaacggagcctcattcttcttcatctgcatctacctccacatcggacgaggcctctactatggatcttacctctataaagaaacctggaacacaggtgttatcctactactaaccctcatagctaccgcattcgtaggatatgtcctgccatgaggccaaatatcattttggggggctaccgtcatcactaacctattctcggccgtaccctacatcggtcagacccttgtagaatgggcctgaggcggattttcagtagacaaccccacactaacccgattcttcgccctccacttcctcctcccctttatcattgccggactaacactcatccaccttaccttcctccacgaatcaggctccaataacccactcggaattacatcagactgcgataaaatcccattccacccctacttctccttaaaagactcactgggcctagtactccttctactacccctaacaaccctagctctattctcacccaacctcctcggagaccctgaaaacttcaccccagcgaacccactagtaactcccccccacatcaaaccagagtgatacttcctgttcgcctacgccatcctacgctcaatcccaaacaaactaggaggagtgctggcactcgctgcctccgtactagtcctattcctaatccccctgctacataaatccaaacaacgagcaatgaccttccgcccgctctcccaactcctattttgagccctaaccaccaacctccttattctaacgtgagtggg----------------------------------------------------------------------------------------------------------------------------

Otus_spilocephalus_hambroecki_92 ---------------------------------------------------------------------------------------------------ttcgggtccctccttggaatctgcctaataacccaaatcaccactggccttctcctagccgcacactacacagccgactcagccttagccttctcatccgtagcacacacttgccgaaacgtccaatacggctgactactccgaaacctccatgcaaacggagcctcattcttcttcatctgcgtctacctccacatcggacgaggcctctactatggatcctacctctacaaagaaacctggaacacaggcgttatcctactactaaccctcatagccaccgcattcgtagggtatgtcctgccatgaggccaaatatcattttggggggctaccgtcatcactaacctactctcagccgtaccttacatcggtcagaccctcgtagaatgggcctgaggcggattttcagtagacaaccccacactaacccgattcttcgccctccacttcctcctcccctttatcattgcagggctaacactcatccacctcaccttcctccacgaatcaggctccaacaacccactcggaattacatcagactgcgacaaaatcccattccacccctacttctccttaaaagactcactgggcctagtacttcttctactacccctaacaaccctagctatgttctcacccaacctcctcgga---------------------------------------------------------------------------------------------------------------------------------------------------------------------------------------------------------------------------------------------------------------------------------------------------------------------------------------------------------------------------------------------------

_Otus_spilocephalus_latouchi_CHIMERA ---------------------------------------------------------------------------------------------------ttcgggtccctccttggaatctgcctaataacccaaatcattactggccttctcctggccgcacactacacagccgactcaaccttagccttctcatccgtagcacacacttgccgaaacgtccaatacggctgactactccgaaacctccatgctaacggagcctcattcttcttcatctgcatctaccttcacatcggacgaggcctctactatggatcctacctctataaagaaacctggaacacaggcgttatccttctactaaccctcatagccaccgcattcgtaggatatgtcctgccatggggccaaatatcattttggggggctaccgtcatcactaacctattctcagccgtaccctacatcggccagacccttgtagaatgagcctgaggcggattctcagtagacaaccccacactaacccgattcttcgccctccacttcctcctcccctttatcattgcagggctaacactcatccacctcaccttcctccacgaatcaggctccaacaacccactcggaattacatcagactgcgacaaaatcccattccacccctacttctccttaaaagatttactgggcctagtactccttctactacccctaacaaccctagctttattctcacccaacctcctcggagaccccgaaaacttcaccccagcgaacccactggtaactcctcctcacatcaaaccagagtggtacttcctgttcgcctacgccatcctacgctcaatcccaaacaaactaggaggagtgctggcactcgctgcctctgtactagtcctattcctaatccccctgctacataaatccaaacaacgagcaatgaccttccgcccgctctcccaactcctattttgagctctagccaccaacctcctcattctaacatgagtaggca--------------------------------------------------------------------------------------------------------------------------

Otus_hartlaubi_13 ----------------------------------------------------------------------------------------------------------------------tctgcttagtaacccaaatcatcactggcctcctcctagccgcacattacacagctgattccaccctagccttctcatccgtagcacatacctgccgaaacgtacaatacggttgactactccgaaacctccatgcaaacggagcctcattcttcttcatctgtatctaccttcacatcggacggggcctctactatggctcctacctctacaaagaaacctgaaacacaggtgtcatcttattgttaaccctaatagcaaccgccttcgtagggtacgtgctgccatgaggccaaatatcattttgaggggctactgtcatcaccaacctcttttcagccgtaccctacatcggacaaacccttgtagaatgagcctgaggaggattttcagtggacaaccccacactaactcgattcttcgccctccacttcctcctccccttcatcattnnnnnnnnnnnnnnnnnncacctnaccttcctccacgaatcaggctcaaacaaccccctcggaatcacatcagattgcgacaaaatcccattccacccctacttctcaataaaagacatcctaggcttagcactaatactcctacccctaacaaccctagccctattctgacccaatctcctcggagaccccgaaaacttcacaccagctaacccgctagttaccccccctcatattaaaccagaatgatacttcctattcgcctacgccatcctacgctcaatccccaacaaactaggaggagtacttgcactcgccgcctccgtactagtcttattcctaatcccactactccacaaatccaaacagcgagcaataaccttccgcccactctcccaactcctattttgaaccctagccaccaacctacttatcctaacatgagtagg----------------------------------------------------------------------------------------------------------------------------

Otus_hartlaubi_15 ---------------------------------------------------------------------------------------------------------------------------------------------------------------------------------------------------------------------------------------------------------------------------------------------------------------------------------------------------------------------------------------------------------------------------------------------------------------------------------------------------------------------------------------------------------------------------------------------------------------ctcaccttcctccacgaatcaggctcaaacaaccccctcggaatcacatcagattgcgacaaaatcccattccacccctacttctcaataaaagacatcctaggcttagcactaatactcctacccctaacaaccctagccctattctgacccaatctcctcggagaccccgaaaacttcacaccagctaacccgctagttaccccccctcatattaaaccagaatgatacttcctattcgcctacgccatcctacgctcaatccccaacaaactaggaggagtacttgcacttgccgcctccgtactagtcttattcctaatcccactactccacaaatccaaacagcgagcaataaccttccgcccactctcccaactcctattttgaaccctagccaccaacctacttatcctaacatgagtagg----------------------------------------------------------------------------------------------------------------------------

Otus_hartlaubi_16 ---------------------------------------------------------------------------------------------------------------------------------------------------------------------------------------------------------------------------------------------------------------------------------------------------------------------------------------------------------------------------------------------------------------------------------------------------------------------------------------------------------------------------------------------------------------------------------------------------------------ctcaccttcctccacgaatcaggctcaaacaaccccctcggaatcacatcagattgcgacaaaatcccattccacccctacttctcaataaaagacatcctaggcttagcactaatactcctacccctaacaaccctagccctattctgacccaatctcctcggagaccccgaaaacttcacaccagctaacccgctagttaccccccctcatattaaaccagaatgatacttcctattcgcctacgccatcctacgctcaatccccaacaaactaggaggagtacttgcactcgccgcctccgtactagtcttattcctaatcccactactccacaaatccaaacagcgagcaataaccttccgcccactctcccaactcctattttgaaccctagccaccaacctacttatcctaacatgagtagg----------------------------------------------------------------------------------------------------------------------------

Otus_hartlaubi_14 ---------------------------------------------------------------------------------------------------------------------------------------------------------------------------------------------------------------------------------------------------------------------------------------------------------------------------------------------------------------------------------------------------------------------------------------------------------------------------------------------------------------------------------------------------------------------------------------------------------------ctcaccttcctccacgaatcaggctcaaacaaccccctcggaatcacatcagattgcgacaaaatcccattccacccctacttctcaataaaagacatcctaggcttagcactaatactcctacccctaacaaccctagccctattctgacccaatctcctcggagaccccgaaaacttcacaccagctaacccgctagttaccccccctcatattaaaccagaatgatacttcctattcgcctacgccatcctacgctcaatccccaacaaactaggaggagtacttgcacttgccgcctccgtactagtcttattcctaatcccactactccacaaatccaaacagcgagcaataaccttccgcccactctcccaactcctattttgaaccctagccaccaacctacttatcctaacatgagtagg----------------------------------------------------------------------------------------------------------------------------

Otus_feae_45 --------------------------------------------------------------------------------------------------------------------------------------------------------------------------------------------------------------------------------------------------------------------------------------------------------------------------------------------------------------------------------------------------------------------------------------------------------------------------------------------------------------------------------------------------------------------------------------------------------------------ctttctccatgaatcaggctcaaacaaccccctcggaatcacatcagactgcgacaaaatcccattccacccctacttctcaacaaaagacatcctaggcttagcactaatactcctacccttaacaaccctagccctgttctgacctaacctcctcggagaccccgaaaacttcacaccagctaacccgctagttaccccccctcatatcaaaccagaatgatacttcctattcgcctatgccatcctacgctcaatccccaacaagctagggggagtacttgcactcgctgcctctgtactaatcttattcctaatcccactactccacaaatccaaacaacgagcaataaccttccgcccactctcccaactcctattttgaactctagccaccaaccttctcatcctaacatgagtagg----------------------------------------------------------------------------------------------------------------------------

Otus_senegalensis_17 ----------------------------------------------------------------------------------------------------------------------tctgcctaataacccaaatcatcactggcctcctcctagccgcacattatacagctgattccaccctagccttctcatccgtagcacatacctgccgaaatgtacaatacggctgactactccgaaacctccatgcaaacggagcctcattcttcttcatctgtgtctaccttcacattggacgaggcctctactatggctcctacctctacaaagaaacctgaaatacgggtgtcatcctactgctaactctaatagcaaccgcctttgtagggtacgtgctgccatgaggccaaatatcattttgaggggccactgttatcaccaacctcttttcagccgtaccctacatcggacaaacccttgtagaatgagcctgagggggattttcagtagacaaccccacactaactcgattcttcgcccttcacttcctcctcccctttatcattgcaggactaaccctcatccatctcacctttctccatgaatcaggctcaaacaaccccctcggaatcacatcagactgcgacaaaatcccattccacccctacttctcaacaaaagacatcctaggcttagcactaatactcctacccttaacaaccctagccctgttctgacctaacctcctcggagaccccgaaaacttcacaccagctaacccgctagttaccccccctcatatcaaaccagaatgatactttctattcgcctatgccatcctacgctcaatccccaacaagctagggggagtacttgcactcgctgcctctgtactaatcttattcctaatcccactactccacaaatccaaacaacgagcaataaccttccgcccactctcccaactcctattttgaactctagccaccaaccttcttatcctaacatgagtagg----------------------------------------------------------------------------------------------------------------------------

Otus_pamelae_74 ---------------------------------------------------------------------------------------------------------------------------------------------------------------------------------------------------------------------------------------------------------------------------------------------------------------------------------------------------------------------------------------------------------------------------------------------------------------------------------------------------------------------------------------------------------------------------------------------------------------------------------------------------------------------------------------------------------------------------------------------------------------------------------------------------------------------------------------------------------------------------------------------------------------------------------------------------------------------------------------------------------------------------------------------------------------------------------------------------------------------------------------------------------------------------------------

Otus_sp_nov_1 ---------------------------------------------------------------------------------------------------------------------------------------------------------------------------------------------------------------------------------------------------------------------------------------------------------------------------------------------------------------------------------------------------------------------------------------------------------------------------------------------------------------------------------------------------------------------------------------------------------------ctcaccttcctccacgaatcaggctcaaacaaccccctcggaatcacatcagactgcgacaaaatcccattccacccctacttctcaacaaaagacatcctaggcttagcactaataatcctacccttaacaaccctagccttgttctgacctaacctcctcggagaccccgaaaacttcacaccagctaacccgctaattaccccccctcatatcaaaccagaatgatacttcctattcgcctacgccatcctacgctcaatccccaacaaactagggggagtacttgcacttgccgcctccgtactagtcctattcctaatcccactactccacaaatccaaacaacgagcaataaccttccgcccactctcccaactcctattttgagccctagccactaacctactcgtcctaacatgggtagg----------------------------------------------------------------------------------------------------------------------------

Otus_sp_nov_2 ---------------------------------------------------------------------------------------------------------------------------------------------------------------------------------------------------------------------------------------------------------------------------------------------------------------------------------------------------------------------------------------------------------------------------------------------------------------------------------------------------------------------------------------------------------------------------------------------------------------ctcaccttcctccacgaatcaggctcaaacaaccccctcggaatcacatcagactgcgacaaaatcccattccacccctacttctcaacaaaagacatcctaggcttagcactaataatcctacccttaacaaccctagccttgttctgacctaacctcctcggagaccccgaaaacttcacaccagctaacccgctaattaccccccctcatatcaaaccagaatgatacttcctattcgcctacgccatcctacgctcaatccccaacaaactagggggagtacttgcacttgccgcctccgtactagtcctattcctaatcccactactccacaaatccaaacaacgagcaataaccttccgcccactctcccaactcctattttgagccctagccactaacctactcgtcctaacatgggtagg----------------------------------------------------------------------------------------------------------------------------

Otus_sp_nov_3 ---------------------------------------------------------------------------------------------------------------------------------------------------------------------------------------------------------------------------------------------------------------------------------------------------------------------------------------------------------------------------------------------------------------------------------------------------------------------------------------------------------------------------------------------------------------------------------------------------------------ctcaccttcctccacgaatcaggctcaaacaaccccctcggaatcacatcagactgcgacaaaatcccattccacccctacttctcaacaaaagacatcctaggcttagcactaataatcctacccttaacaaccctagccttgttctgacctaacctcctcggagaccccgaaaacttcacaccagctaacccgctaattaccccccctcatatcaaaccagaatgatacttcctattcgcctacgccatcctacgctcaatccccaacaaactagggggagtacttgcacttgccgcctccgtactagtcctattcctaatcccactactccacaaatccaaacaacgagcaataaccttccgcccactctcccaactcctattttgagccctagccactaacctactcgtcctaacatgggtagg----------------------------------------------------------------------------------------------------------------------------

Otus_sp_nov_4 ---------------------------------------------------------------------------------------------------------------------------------------------------------------------------------------------------------------------------------------------------------------------------------------------------------------------------------------------------------------------------------------------------------------------------------------------------------------------------------------------------------------------------------------------------------------------------------------------------------------ctcaccttcctccacgaatcaggctcaaacaaccccctcggaatcacatcagactgcgacaaaatcccattccacccctacttctcaacaaaagacatcctaggcttagcactaataatcctacccttaacaaccctagccttgttctgacctaacctcctcggagaccccgaaaacttcacaccagctaacccgctaattaccccccctcatatcaaaccagaatgatacttcctattcgcctacgccatcctacgctcaatccccaacaaactagggggagtacttgcacttgccgcctccgtactagtcctattcctaatcccactactccacaaatccaaacaacgagcaataaccttccgcccactctcccaactcctattttgagccctagccactaacctactcgtcctaacatgggtagg----------------------------------------------------------------------------------------------------------------------------

Otus_scops_5 ----------------------------------------------------------------------------------------------------------------------tctgcttaacaacccaaatcatcactggcctcctcctagccgcacattacacagctgactccaccctagccttctcatccgtagcacatacatgccgaaatgtacaatacggctgactactccgaaaccttcatgcaaacggagcctcattcttcttcatctgtatctaccttcacatcggacgaggcctctactatggctcctacctctacaaagaaacctgaaatacaggtgtcatcttactactaactctaatagcaaccgcctttgtaggatatgtactgccatgaggacaaatatcattttgaggagctaccgtcatcaccaacctcttttcagccgtaccctacatcggacaaacccttgtagagtgggcctgaggaggattttcagtagacaaccccacactaactcgattctttgcccttcacttcctcctcccctttatcattgcaggactaaccctcatccatctcacctttctccacgaatcaggctcaaacaaccctctcggaattacatccgactgcgataaaatcccattccacccctacttctcaataaaagacatcctaggcctagcactaatactcctacccttaacaaccctagccttattctgacccaatctccttggagaccccgaaaacttcacaccagccaacccactagttaccccccctcatatcaaaccagaatgatacttcctattcgcctacgccatcctacgctcaatccccaacaaactaggaggagtacttgcacttgccgcctccgtactagtcctattcctaatcccactactccacaaatccaaacaacgagcaataaccttccgcccactctcccaactcctattttgaaccctagccaccaacctactcgtcctaacatgggtagg----------------------------------------------------------------------------------------------------------------------------

_Otus_scops_JF5337_F ---------------------------------------------------------------------------------------------------tttggatccctccttggcatctgcttaacaacccaaatcatcactggcctcctcctagccgcacattacacagctgactccaccctagccttctcatccgtagcacatacatgccgaaatgtacaatacggctgactactccgaaaccttcatgcaaacggagcctcattcttcttcatctgtatctaccttcacatcggacgaggcctctactatggctcctacctctacaaagaaacctgaaatacaggtgtcatcttactactaactctaatagcaaccgccttcgtaggatatgtactgccatgaggacaaatatcattttgaggagctaccgtcatcaccaacctcttttcagccgtaccctacatcggacaaacccttgtagagtgggcctgaggaggattttcagtagacaaccccacactaactcgattctttgcccttcacttcctcctcccctttatcattgcaggactaaccctcatccatctcacctttctccacgaatcaggctcaaacaaccctctcggaattacatccgactgcgataaaatcccattccacccctacttctcaataaaagacatcctaggcctagcactaatactcctacccttaacaaccctagccttattctgacccaatctccttggagaccccgaaaacttcacaccagccaacccactagttaccccccctcatatcaaaccagaatgatacttcctattcgcctacgccatcctacgctcaatccccaacaaactaggaggagtacttgcacttgccgcctccgtactagtcctattcctaatcccactactccacaaatccaaacagcgagcaataaccttccgcccactctcccaactcctattttgaaccctagccaccaacctactcgtcctaacatgggtaggtagccaaccagtagaacatcccttcatcatcatcggccaactagcttcaatcacctacttcacaacaattctaatcctcttcccagcaatcagtaccctagaaaacaaactacttaactac---

Otus_pembaensis_24 ----------------------------------------------------------------------------------------------------------------------------------cccaaatcatcactggcctcctcctagccgcacattacacagctgattccaccctagccttctcatccgtagcacatacttgccgaaacgtacaatacggctggctactccgaaacctccatgcaaacggagcctcattcttcttcatctgcatttaccttcacatcggacgaggcctctactatggctcctacctctacaaagaaacctgaaacacaggtgtcatcttactactaactctaatagcaaccgccttcgtagggtatgtgctgccatgaggccaaatatcattttgaggggctactgttatcaccaacctcttttcagccgtaccctacatcggacaaacccttgtagaatgagcctgagggggattttcagtagacaaccccacactaactcgattcttcgcccttcacttcctccnnnnnnnnnnnnnnnnnnnnnnnncccttatccatctcacctttctccacgaatcaggctcaaacaaccccctcggaatcgcatcagactgcgacaaaatcccattccncccctacttctcaataaaagacatcctaggcctagcactaatactcctacccttaacaaccctagccctgttctgacctaacctcctcggagaccccgaaaacttcacnccagccaacccactagttactccccctcatatcaaaccagaatgatacttcctattcgcctacgccatcctacgctcaatccccaacaaactaggaggagtacttgcacttgccgcctccgtactagtcttattcctaatcccactacttcacaaatccaaacaacgagcaataaccttccgcccactctcccaactcctattttgaaccctaaccaccaacctacttatcctaacatgagtagg----------------------------------------------------------------------------------------------------------------------------

Otus_pembaensis_25 ----------------------------------------------------------------------------------------------------------------------------------cccaaatcatcactggcctcctcctagccgcacattacacagctgattccaccctagccttctcatccgtagcacatacttgccgaaacgtacaatacggctggctactccgaaacctccatgcaaacggagcctcattcttcttcatctgcatttaccttcacatcggacgaggcctctactatggctcctacctctacaaagaaacctgaaacacaggtgtcatcttactactaactctaatagcaaccgccttcgtagggtatgtgctgccatgaggccaaatatcattttgaggggctactgttatcaccaacctcttttcagccgtaccctacatcggacaaacccttgtagaatgagcctgagggggattttcagtagacaaccccacactaactcgattcttcgcccttcacttcctccnnnnnnnnnnnnnnnnnnnnnnnncccttatccatctcacctttctccacgaatcaggctcaaacaaccccctcggaatcgcatcagactgcgacaaaatcccattccncccctacttctcaataaaagacatcctaggcctagcactaatactcctacccttaacaaccctagccctgttctgacctaacctcctcggagaccccgaaaacttcacnccagccaacccactagttactccccctcatatcaaaccagaatgatacttcctattcgcctacgccatcctacgctcaatccccaacaaactaggaggagtacttgcacttgccgcctccgtactagtcttattcctaatcccactacttcacaaatccaaacaacgagcaataaccttccgcccactctcccaactcctattttgaaccctaaccaccaacctacttatcctaacatgagtagg----------------------------------------------------------------------------------------------------------------------------

_Otus_longicornis_CHIMERA ----------------------------------------------------------------------------------------------------------------------tctgcctaataacccaaatcatcactggcctcctcctagccgcacactacacagctgattccaccctagccttctcatccgtagcacacatatgccgaaacgtacaatacggctgactgctccgaaacctccatgcaaacggagcctcattcttcttcatctgcatctacctccacatcggacgaggcctctactacggctcctacctctacaaagagacctggaacacaggcgtcatcctgctactgactctaatagcaaccgccttcgtaggatatgtactgccatgaggccaaatatcattttgaggggctaccgtcatcaccaacctcttctcagccgtaccctacatcggacaaacccttgtagaatgagcctgaggaggattttcagtagacaaccccacactaacccgattcttcgccctccacttcctcctccccttcatcattgccggactaaccctcatccacctcaccttcctccacgaatcaggctcgaacaaccctctcggaattacatccgactgtgacaaaatcccattccacccctacttctcgataaaagatatcctaggcctagcactaatactcctacccttaacaaccctagccctattctgacccaacttcctcggagaccctgaaaacttcacaccagccaacccactaatcaccccccctcatattaaaccagaatgatacttcctattcgcctacgccatcctacgctcaatccccaacaaactgggaggagtacttgcacttgccgcttctgtactaattctattcctaatccccctactccacaaatccaaacaacgagcaataaccttccgcccactctcccagctcttattctgaactctaaccaccaacctacttattctaacatgagtagg----------------------------------------------------------------------------------------------------------------------------

Otus_mirus_CHIMERA -----------------------------------------------------------------------------------------------------------------------ctgcttannnacccaaatcatcaccggcctcctcctagccgcacactacacagccgattctaccctagccttctcatccgtagcacacacatgccgaaacgtacaatacggctgactgctccgaaaccttcatgcaaacggagcctcattcttcttcatctgcatctaccttcacatcggacgaggcctctactacggctcctacctctacaaagaaacctgaaacacaggtgtcatcctactactaactctaatagcaaccgccttcgtaggatatgtgctgccatgaggccagatatcattctgaggggctaccgttatcaccaacctcttctcagccgtaccctacatcggacaaacccttgtagaatgagcctgagggggattttcagtagacaaccccacgctaactcgattcttcgccctccacttcctcctncccttcatnattgcnnnnnnnnnnnnnnnncacctcaccttcctccacgaatcaggctcgaacaaccctctcggaattacatcagactgtgacaaaatcccattccacccctacttctcaataaaagataccctaggcctagcactaatactcctacccttaacaaccttagccttactctgtcctaacctcctcggagaccctgaaaacttcacaccagccaacccactagtcaccccccctcacattaaaccagaatgatacttcctattcgcctacgccatcctacgctcaatccccaacaaactgggaggagtacttgcacttgccgcctccgtactaattctattcctaatccccctactccacaaatccaagcagcgagcaataaccttccgtccactctctcaactcttattctgagccctagccaccaatctactcgtcctaacatgagtagg----------------------------------------------------------------------------------------------------------------------------

Otus_mayottensis_60 ----------------------------------------------------------------------------------------------------------------------tctgcctaataacccaaatcatcactggcctcctcctagccgcacactacacagctgattctaccctagccttctcatccgtagcacatacatgccgaaatgtgcaatacggctgactgctccgaaatcttcatgcaaacggagcctcattcttcttcatctgtatttaccttcacatcggacgaggcctctactacggctcctacctctacaaagaaacctgaaacacaggcgtcatcctactactgactttaatagcaaccgccttcgtaggatatgtactgccatgaggccaaatatcattctgaggggctaccgtcatcaccaacctcttctcagctgtaccctacatcggacaaacccttgtggaatgagcctgaggaggattttcagtagacaaccccacactaactcgattcttcgccctccacttcctcctccccttcatcattgccggactgacccttatccacctcaccttcctccacgaaacaggatcaaacaaccctctcggaatcacatccgactgtgacaaaatcccattccacccctacttctcaataaaagacatcctaggcctagcagtaatactcctacccttaacaactctagccctattctgacctaacctcctcggagaccctgaaaacttcacaccagccaacccgctaattactccccctcatatcaaaccagaatgatacttcctattcgcctacgccatcctacgctcaatccccaacaaactaggaggagtacttgcacttgccgcctccgtactagtcctattcctaatccccctactccacaaatccaaacaacgagcaangaccttccgcccattctcccaactcntattctgaaccctaacc---------------------------------------------------------------------------------------------------------------------------------------------------------

Otus_capnodes_40 --------------------------------------------------------------------------------------------------------------------------------aacccaaatcatcaccggcctcctcctagccgcacactacacagctgactccaccttagccttctcatccgtagcacacacatgccgaaatgtacaatacggctgactactccgaaaccttcatgcaaacggagcctcattcttcttcatctgtatctaccttcacatcggacgaggactctactacggctcctacctctacaaagaaacctgaaacacaggcgtcatccttctactaaccctgatagcaaccgccttcgtaggatatgtactgccatgaggccaaatatcattctgaggggctaccgtcatcaccaacctcttctcagccgtaccctacatcggacaaacccttgtagaatgagcctgaggaggattttcagtagacaaccctacactaacccgattcttcgccctccacttcctcctccccttcatcattgctggactaacccttatccacctcaccttcctccacgaatcaggctcgaacaaccccctcggaatcacgtcagactgtgacaaaatcccattccacccttacttctcaataaaggacatcctaggcctagcaataatactcctacccttaacaaccatagccctattctgacctaacctcctcggagaccctgaaaacttcacaccagccaacccactagttactcctccccatatcaagccagaatgatacttcctattcgcctatgctatcctacgctcaatccccaacaaactaggaggggtacttgcacttgccgcctccgtactagtcctattcttaatccccctactccacaaatccaaacaacgagcaataaccttccgcccactctctcaactcntattctgatccctaaccaccaac---------------------------------------------------------------------------------------------------------------------------------------------------

_Otus_madagascariensis_CHIMERA ---------------------------------------------------------------------------------------------------ttcggatccctcctcggcatctgcttaacaacccaaatcatcactggcctcctcctagccgcacactacacagctgactctaccttagccttttcatccgtagcacacacatgccgaaacgtacaatacggctgactactccgaaaccttcatgcaaacggagcctcactcttcttcatctgtatctaccttcacatcggacgaggcctctactacggctcctacctctacaaagaaacctggaacacaggcgtcatcctgctactaactctaatagcaaccgctttcgtagggtatgtactgccatgaggccaaatatcattctgaggagctaccgtcatcaccaacctcttctcagccgtaccctacatcggacaaacccttgtggaatgagcctgaggggggttttcagtagacaaccccacactaactcgattcttcgccctccacttcctcctccccttcatcattgctggactgacccttatccacctcaccttccttcacgaatcgggctcaaacaaccctctcggaatcacatcagactgcgacaaaatcccattccacccctacttctcaataaaagacatcctaggcctagcaatcatactcctacccttgacaactctagccctattctgacctaacctcctcggagaccccgaaaacttcacaccagccaacccactagttactcccccccacattaaaccagaatgatacttcctattcgcctacgctattctacgctcaatccccaacaaactaggaggagtacttgcacttgccgcctccgtactagtcctattcctaatccccctactccacaaatccaaacaacgagcaataaccttccgcccactctcccaactcttattctgaaccctaaccaccaacctattcatcctaacatgagtaggcagccaaccagtagaacaccccttcatcatcattggccaattagcctcaatcacctacttcacaacaa--------------------------------------------------------

Otus_pauliani_76 ----------------------------------------------------------------------------------------------------------------------tctgcttagnaacccaaatcatcactggcctccttctagccgcacattatacagctgactctaccttagccttttcatccgtagcccacacatgtcgaaatgtacaatacggctgactactccgaaaccttcatgcaaacggagcctcactcttctttatctgtatctaccttcacatcggacgaggcctctactacggctcctacctctacaaagaaacatgaaacacaggcgtcatcctactactaactctaatagcaaccgccttcgtaggatatgtacttccatggggccaaatatcattctgaggggctaccgtcatcaccaacctcttctcagccgtaccctacatcggacagacccttgtagaatgagcctgagggggattttcagtagacaaccccacactaactcgattcttcgccctccacttcctcctccccttcatcattgctggactgacccttatccacctcaccttcctccacgaatcaggttcgaacaaccctctcggaatcacatcagactgtgacaaaattccattccacccctacttctcaacaaaagacatcctaggcctagcaacaatactcctacccttaacaaccctagccctattctgacctaacctccttggagaccctgaaaacttcacaccagccaacccgctagttactccccctcatattaagccagaatggtacttcctattcgcctacgccatcctacgttcaattcccaacaaactaggaggagtacttgcacttgccgcctccgtactagtcctgttcctaatccctctactccacaaatccaaacaacgagcaataaccttccgcccactctcccaactcctattttgaaccct-------------------------------------------------------------------------------------------------------------------------------------------------------------

Otus_insularis_47 ----------------------------------------------------------------------------------------------------------------------tttgcctagcaacccaaatcatcactgggctcctcctagccgcacactacacaactgactctaccctagccttctcatccgtagcacacacatgccgaaatgtacaatacggctgactactccgaaaccttcatgcaaacggagcctcattctttttcatctgtatctaccttcacatcggacgaggcctctactacggctcctacctctacaaagaaacctgaaacacaggtgtcatcctactactaactctaatagcaaccgccttcgtaggatatgtactgccatgaggccaaatgtcattctgaggggctaccgtcatcaccaacctcttctcagccgtaccctacatcggacaaaccctcgtagaatggacctgaggaggattttcagtagacaaccccacactaactcgattcttcgccctccacttcctcctccccttcatcattgctggactgaccctcatccacctcaccttcctccacgaatcaggctcgaacaaccctctcggaatcacatcagactgcgacaaaatcccattccatccctacttctcaataaaagacatcctaggcctagcaataatactcctacccttaacaaccctagccttattctgacccaacctcctcggagaccctgaaaacttcacaccagccaacccgctagttactcctccccatattaaaccagaatgatacttcctattcgcctatgccatcctacgctcaatccccaacaaactaggaggagtacttgcacttgccgcctccgtactagtcctattcctaatccccctactccacaaatccaaacaacgagcaataaccttccgcccactctctcaactcttattctgaaccctaaccaccaacctactcatcctaacatgagtagg----------------------------------------------------------------------------------------------------------------------------

Otus_socotranus_89 ---------------------------------------------------------------------------------------------------------------------------------------------------------------------------------------------------------------------------------------------------------------------------------------------------------------------------------------------------------------------------------------------------------------------------------------------------------------------------------------------------------------------------------------------------------------------------------------------------------------------------------------------------------------------------------------------------------------------------------------------------------------------------------------------------------------------------------------------------------------------------------------------------------------------------------------------------------------------------------------------------------------------------------------------------------------------------------------------------------------------------------------------------------------------------------------

_Otus_sunia_CHIMERA ---------------------------------------------------------------------------------------------------ttcggatctctccttggtatttgcctaacaacccaaatcatcactggactcctcctagccgcacactacacagctgactctaccttagccttctcatccgtagcacacacatgccgaaatgtacaatacggctgactactccgaaaccttcatgcaaacggagcctcattctttttcatctgtatctaccttcacatcggacgaggcctttactacggctcctacctctacaaagaaacctgaaacacaggtgtcatcctactactaactctaatagcaaccgccttcgtaggatatgtactgccatggggccaaatgtcattctgaggggctaccgtcatcaccaacctcttctcagccgtaccctacatcggacaaaccctcgtagagtgggcctgaggaggattttcagtagacaaccccacactaacccgattcttcgccctccacttcctcctccccttcatcattgccggactgaccctcatccacctcaccttcctccacgaatcaggctctaacaaccccctaggaattacatcagactgcgacaaaatcccattccatccctacttctcaataaaagacatcctaggcctagcaataatactcctaccccttacaaccctagccttattctgacccaacctcctcggagacccagaaaacttcacaccagccaacccgctagttactcccccccatattaaaccagaatgatatttcctattcgcctacgccatcctacgctcaatccccaacaaactaggaggagtacttgcactcgccgcctctgtactagttctattcctaattcccctactccacaaatccaaacaacgagcaataaccttccgcccactctctcaactcttattctgaaccctaaccaccaacctactcatcctaacatgagtaggcag-------------------------------------------------------------------------------------------------------------------------

Otus_moheliensis_CHIMERA ---------------------------------------------------------------------------------------------------------------------------------------------------------------------------------------------------------------------------------------------------------------------------------------------------------------------------------------------------------------------------------------------------------------------------------------------------------------------------------------------------------------------------------------------------------------------------------------------------------------------ttcctccacgaatcaggctcgaacaaccctctcggaatcacatcagactgtgacaaaatcccattccacccctatttctcaataaaagacatcctaggcctagcaataatactcctacctctaacaaccctagccttattctgacccaaccttctcgtagaccctgaaaacttcacaccagccaatccactagttactccccctcacattaaaccagaatggtacttcctattcgcctatgccatcctacgctcaatccccaacaaactaggaggagtacttgcacttgccgcctccgtactagtcctattcctaatccctctactccacaaatccaaacaacgagcaataaccttccgcccactctctcaactcttattctgaaccctaaccaccaacctactcatcctaacatgggtagg----------------------------------------------------------------------------------------------------------------------------

Otus_brucei_CHIMERA ---------------------------------------------------------------------------------------------------tttggatccctccttggcatctgcttagcaacccaaatcattactggcctcctcctagccgcacattacacagctgactccaccctagccttctcatccgtagcacatacatgccgaaatgtacaatacggctgactactccgaaaccttcatgcaaacggagcctcattcttcttcatctgtatctaccttcacatcggacgaggcctctactatggctcctacctctacaaagaaacctgaaatacaggtgtcatcttactactaactctaatagcaaccgccttcgtaggatatgtactgccatgaggacaaatatcattttgaggagctaccgtcatcaccaacctcttttcagccgtaccctacatcggacaaacccttgtagagtgggcctgaggaggattttcagtagacaaccccacactaactcgattctttgcccttcacttcctcctcccctttatcattgcaggactaaccctcatccatctcacctttctccacgaatcaggctcaaacaaccctctcggaattacatccgactgcgataaaatcccattccacccctacttctcaataaaagacatcctaggcctagcactaatactcctacccttaacaaccctagccttattctgacccaatctccttggagaccccgaaaacttcacaccagccaacccactagttaccccccctcatatcaaaccagaatgatacttcctattcgcctacgccatcctacgctcaatccccaacaaactaggaggagtacttgcacttgccgcctccgtactagtcctattcctaatcccactactccacaaatccaaacaacgagcaataaccttccgcccactctcccaactcctattttgaaccctagccaccaacctactcgtcctaacatgggtaggtagccaaccagtagaacaccccttcatcatcatcggtcaactagcttcaatcacctacttcacaacaattctaatcctcttcccagcaatcagtaccctagaaaacaaactacttaactac---

Otus_icterorhynchus_holerythrus_27 ---------------------------------------------------------------------------------------------------------------------------------------------------------------------------------------------------------------------------------------------------------------------------------------------------------------------------------------------------------------------------------------------------------------------------------------------------------------------------------------------------------------------------------------------------------------------------------------------------------------------------------------------------------------------------------------------------------------------------------------------------------------------------------------------------------------------------------------------------------------------------------------------------------------------------------------------------------------------------------------------------------------------------------------------------------------------------------------------------------------------------------------------------------------------------------------

Otus_icterorhynchus_CHIMERA ---------------------------------------------------------------------------------------------------tttggatcccttcttggcatctgcctagcaactcaaatcatcaccggcctcctcctagccgcccactacacagctgactccacaatagccttctcatccgtagcacacacctgccgaaacgtacagtacggctgactacttcgaaacctccacgcaaacggagcctcattcttcttcatctgtatttacctccatatcggacgaggcctctactacggctcctacctctacaaagaaacctgaaacacaggtgtcatcctactactgaccttaatagcaactgccttcgtggggtatgtactgccatgaggccaaatatcattctgaggggcaactgtcatcaccaatctattctcagccgtaccctatatcggccagacccttgtagaatgagcctgagggggattctcagtagataacccaacactaactcggttcttcgcccttcacttcctcctccccttcatcattgcaggactgacactcatccacctcaccttcctccacgaatcaggctcgaacaacccactcggaatcacatcagactgcgacaaaatcccattccacccctacttctcgacaaaagacatcctaggcttagcactaatactcctacccctaacaaccctagctttactctcacccaacctcctcggggaccccgaaaacttcacaccagccaacccactagttaccccccctcacatcaaaccagaatggtacttcctatttgcctacgctatcctacgctcaatcccc---------------------------------------------------------------------------------------------------------------------------------------------------------------------------------------------------------------------------------------------------------------------------------------------

Otus_ireneae_49 ----------------------------------------------------------------------------------------------------------------------tctgcttagtaacccaaatcatcaccggcctcctactagcggcacactatacagccgattcagccctagccttctcatccgtagcacacacctgccgaaatgtccaatatgggtgactactccgaagcctccatgcaaacggagcctcattcttcttcatctgcatctacctacacatcggacgaggcctctactatggctcctacctctacaaagaaacctgaaacactggcatcatcctactactgaccctgatagcaaccgccttcgtaggatacgtcctgccatgaggccaaatatcgttttgaggggcaaccgtcatcaccaacctattctcagccgtaccctacatcggacaaaccctagtagaatgggcctgaggtggattctcagtagacaaccctactctaacccgattcttcaccctccacttcctcctccccttcatcattgcaggattgacgctcatccacctcaccttcctccacgaatcaggctcaaacaatccactaggaattttatcagattgcgataaaatcccgttccacccctacttttctacaaaagacgccctaggcctgatactcatacttctacccctaatgactctaaccctactcgcacccaacctgctaggagacccagaaaacttcacaccagcaaacccactaatcaccccaccccacattaaaccagaatgatacttcttattcgcttatgccatcctacgctcgatccctaacaaactaggaggagtactagcacttgccgcctcagtactaatcctattcttagtcccacttctccacaaatcgaagcaacgaacaataaccttccgcccactctcccaactcctgttctgaaccctgatcgccaacctactcatcctaacctgagtggg----------------------------------------------------------------------------------------------------------------------------

Otus_rutilus_78 ---------------------------------------------------------------------------------------------------------------------------------acccaaatcatcactggcctcctcctagccgcacactacacagctgactctaccttagccttttcatccgtagcacacacatgccgaaacgtacaatacggctgactactccgaaaccttcatgcaaacggagcctcactcttcttcatctgtatctaccttcacatcggacgaggcctctactacggctcctacctctacaaagaaacctggaacacaggcgtcatcctgctactaactctaatagcaaccgctttcgtagggtatgtactgccatgaggccaaatatcattctgaggagctaccgtcatcaccaacctcttctcagccgtaccctacatcggacaaacccttgtggaatgagcctgaggggggttttcagtagacaaccccacactaactcgattcttcgc-------------------------------------------------------------------------------------------------------------------------------------------------------------------------------------------------------------------------------------------------------------------------------------------------------------------------------------------------------------------------------------------------------------------------------------------------------------------------------------------------------------------------------------------------------------------------------------------------------------------------

Otus_scops_scops_8 ---------------------------------------------------------------------------------------------------------------------------------------------------------------------------------------------------------------------------------------------------------------------------------------------------------------------------------------------------------------------------------------------------------------------------------------------------------------------------------------------------------------------------------------------------------------------------------------------------------------------tttctccacgaatcaggctcaaacaaccctctcggaattacatccgactgcgataaaatcccattccacccctacttctcaataaaagacatcctaggcctagcactaatactcctacccttaacaaccctagccttattctgacccaatctccttggagaccccgaaaacttcacaccagccaacccactagttaccccccctcatatcaaaccagaatgatacttcctattcgcctacgccatcctacgctcaatccccaacaaactaggaggagtacttgcacttgccgcctccgtactaatcctattcctaatcccactactccacaaatccaaacagcgagcaataaccttccgcccactctcccaactcctattttgaaccctagccaccaacctactcgtcctaacatgggtagg----------------------------------------------------------------------------------------------------------------------------

Otus_scops_scops_9 ---------------------------------------------------------------------------------------------------------------------------------------------------------------------------------------------------------------------------------------------------------------------------------------------------------------------------------------------------------------------------------------------------------------------------------------------------------------------------------------------------------------------------------------------------------------------------------------------------------------------tttctccacgaatcaggctcaaacaaccctctcggaattacatccgactgcgataaaatcccattccacccctacttctcaataaaagacatcctaggcctagcactaatactcctacccttaacaaccctagccttattctgacccaatctccttggagaccccgaaaacttcacaccagccaacccactagttaccccccctcatatcaaaccagaatgatacttcctattcgcctacgccatcctacgctcaatccccaacaaactaggaggagtacttgcacttgccgcctccgtactagtcctattcctaatcccactactccacaaatccaaacagcgagcaataaccttccgcccactctcccaactcctattttgaaccctagccaccaacctactcgtcctaacatgggtagg----------------------------------------------------------------------------------------------------------------------------

Otus_spilocephalus_luciae_94 -------------------------------------------------------------------------------------------------------------------------------------------------------------------------------------------------------------------------------------------------gaaacctccacgcaaacggagcctcattcttcttcatctgcatctacctccacatcggacgaggcctctactatggatcttacctctayaaagagacctgaaacacaggtgtcatcctactactaaccctcnnnnnnnnnnnnnnnnnnnnnnnnnnnnnnnnnnnnnnnnnnnnnnnnnnnnnnnnnnnnnnnnnnnnnnnnnnnnnnnnnnnnnnnnnnnnnnnnnnnnnnnnnnnnnnnnnnnnnngagcctgaggtgggttttcagtagacaaccccacactaacccgattcttc---------------------------------------------------------------------------------------------------------------------------------------------------------------------------------------------------------------------------------------------------------------------------------------------------------------------------------------------------------------------------------------------------------------------------------------------------------------------------------------------------------------------------------------------------------------------------------------------------------------------------

Otus_silvicola_87 -------------------------------------------------------------------------------------------------------------------------------------------------------------------------------------------------------------------------------------------------gaaacctccatgcaaacggagcctcattcttcttcatctgcatctaccttcacatcggacggggcctctactatggctcctacctctacaaagagacctgaaatacaggtgtcatcctactactaaccctcatagctaccgccttcgtagggtatgtcctaccgtgaggtcaaatatcattctgaggagctactgtcatcaccaacctattctcagccgtaccttacatcgggcaaaccctcgtagaatgagcctgaggtgggttctcagtagacaacccaacactaactcgattcttcgccctccmcttcctcctccccttcatcattgcaggcttgacactcattcacctcacctttctccacgaatcaggctcaaataacccactcggaatctcatcagattgcgacaaaatcccattccacccctacttctcaataaaagacacactaggcctagtactactactattacccctaacaaccctagccttattttcacctaacctccttggagaccccgaaaacttcacaccagctaatccgctagtcactcctccccatatcaaacccgaatggtacttcttatttgcttatgccatcctacgctcaatccccaacaaactaggaggagtactagcactggccgcttccgtactagtcctattcttaatccctctactacacaaatccaagcaacgggcaataaccttccgcccactctcccaactcctattctgaacactaaccgccaacctacttatccttacatgagtgggtag---------------------------------------------------------------------------------------------------------------------c---

_Otus_cyprius_CHIMERA ---------------------------------------------------------------------------------------------------tttggatccctccttggcatctgcttaacaacccaaatcatcactggcctcctcctagccgcacattacacagctgattccaccctagccttctcatccgtagcacatacatgccgaaatgtacaatacggctgactactccgaaaccttcatgcaaacggagcctcatttttcttcatctgtatctaccttcacatcggacgaggcctctactatggctcctacctctacaaagaaacctgaaatacaggtgtcatcttactactaactctaatagcaaccgccttcgtaggatatgtactgccatgaggacaaatatcattttgaggagctaccgtcatcaccaacctcttttcagccgtaccctacatcggacaaacccttgtagagtgggcctgaggaggattttcagtagacaaccccacactaactcgattctttgcccttcacttcctcctcccctttatcattgcaggactaaccctcatccatctcacctttctccacgaatcaggctcaaacaaccctctcggaattacatccgactgcgataaaatcccattccacccctacttctcaataaaagacatcctaggcctagcactaatactcctacccttaacaaccctagccttattctgacccaatctccttggagaccccgaaaatttcacaccagccaacccacaagttaccccccctcatatcaaaccagaatgatacttcctattcgcctacgccatcctacgctcaatccccaacaaactaggaggagtacttgcacttgccgcctccgtacta---------------------------------------------------------------------------------------------------------------------------------------------------------------------------------------------------------------------------------------------------

Otus_elegans_CHIMERA atggcccccaatatacgtaaatcccaccccctcctaaaaatagtcaacaactctctaattgacctacccaccccacccaacatctctgcatgatgaaacttcggatccctccttggcatctgcctaataacccaaatcatcactggcctcctcctagctgcacactacacagccgactctaccttagccttctcatccgtagcacacacatgccgaaatgtacaatacggctggctgctccgaaaccttcatgcaaatggagcctcattcttcttcatctgcatctaccttcacatcggacgaggcctctattacggctcctacctctacaaagaaacctgaaacacaggcgtcatcctactactgactctaatggcaaccgccttcgtaggatatgtactgccatgaggccaaatatcattttgaggtgccaccgtcatcaccaacctcttctcagccgtcccctacatcggacaaaccctcgtagaatgagcctgaggaggattttcagtagacaaccccacactaactcgattcttcgccctccacttcctcctccccttcatcattgctggactgaccctaatccacctcaccttcctccatgactcaggctcaaataaccccctcggaattacatcagactgtgacaaaatcccattccacccctacttctcaataaaagacatcctaggcctagcactaatactcctacccctaacaaccctagccttattctgacccaacctcctcggagaccccgaaaacttcacaccagccaacccgctagttaccccccctcatatcaaaccagaatggtacttcctattcgcctacgccatcctacgctcaatccccaacaaactaggaggagtacttgcacttgccgcctccgtactaatcctattcctaatccctctactccacaaatccaaacagcgagcaataaccttccgcccactctctcagctcctattttgaaccctgactatcaacctactcatcctaacatgagtaggcagccaaccagtagaacaccccttcatcatcatcggccaattagcctcaatcacctacttcacaacaattctagtcctcttcccagcaatcagtaccctagaaaacaaactactcaactactaa

[marker: tgfb2; length: 593; no. of seqs: 51; new coordinates: 3250-3842)]

Bubo_bubo_111 cctattgttttaggtaactatgcatccgtattcagtatacttacatagtagtaccagcctgtgaggtgtaatgcagtttcttatcatgttcccttacggttggcgtttatacagagcgcggaccctcatttgcagacccatggggtctgtgtgcagcaggtatctgcccagtggtctccagtcctgcagagacagtggcagatgaggaaggagggttgtggccttgggaagcagaagctgagttccatctgggatgattcccagacccatcagcctcataagtacagccgaattatcacctccactgagctcatgtagtgagaggccacttggtaggacctgaagaataatacttactaggcctccagggaaaccattagctttgtctgtataag-aagtcccgccatgtctaagcatctgatttcagctgcagtatatgctact-attgatgtgatttggttgtgaatttttgggactcagacttgcctgcctgtaaagcatcattcagggaacaagcaactggattattttaagtccttgtgtcgtcttttatgactgatgtgtttgtctgtatttttattccccaggaat

Strix_aluco_129 cctattgttttaggtaactatgcatccgtattcagtatacttacatagtagtaccagcctgtgaggtgtaatgcagtttcttatcacatttctttacggttggcgtttatacagagcgcggaccctcatttgcagccccatggggtctgtgtgcagcaggtatctgcccagtggtctccagtcctgcagagacagtggcagatgaggaaggagggttgtggccttgggaagcagaagctgagttccatctgggatgattcccagacccatcagccccataagtacagccgaattatcacctccactgagctcatgtagtcagaggccacttggtaggacctgaagaataatacttactagtcctccagggaaaccattaactttgtctgtataagcaagtcctgccatgtctaagcatctgatttcagctgcagtatatgctact-attgatgtgatttggttgtgaatttttgggactcagacttgcctgcctgtaaagcatcattcagggaacaagcaactggattattttaagtccttgtgttgtcttttatgactgatgtgtttgtctgtatttttattccccaggaat

Otus_bakkamoena_marathae_35 -----------------------------------------------------------------------------------------------------------------------------------------------------------------------------------------------------------------------------------------------------------------------------------------------------------------------------------------------------------------------------------------------------------------------------------------------------------------------------------------------------------------------------------------------------------------------------------------------------------------

_Otus_lempiji_CHIMERA cctattgttttaggtaactatgcatccgtattcagtatacttacatagtagtaccagcctgtgaggtgtcatgcagtttcttatcatgttgctttacggttggcgtttatacagagtgcggaccctcatttgcagacccacggggtctgtgtgcagcaggtatctgcccagtggtctccagccctgcagagacagtggcagatgaggaaggagggttgtggccttgggaagcagaagctgagttccctctgggatgattcccagacccatcagcctcataagtacagccgaattaccacctccactgagctcatgtagtcagaggccacttggtaggacctgaagaataa----tactagtcctccagggaaatcattagctttgtctgtwtaagcaagtcctgccatatctaagcatctgatttcagctgcagtatatgctact-actgatgtgatttggttgtgaattttkgggactcagacttgcctgcctgtaaagcatcgttcagggaacaagcaactggattattttaagtccttgtgttgtcttttatgactgatgtgtttgtctgtatttttattccccaggaat

Otus_lettia_lettia_CHIMERA cctattgttttaggtaactatgcatccgtattcagtatacttacatagtagtaccagcctgtgaggtgtcatgcagtttcttatcatgttgctttacggttggcgtttatacagagtgcggaccctcatttgcagacccacggggtctgtgtgcagcaggtatctgcccagtggtctccagccctgcagagacagtggcagatgaggaaggagrgttgtggccttgggaagcagaagctgagttccctctgggatgattcccagacccatcagcctcataagtacagccgaattaccacctccactgagctcatgtagtcagaggccacttggtaggacctgaagaataa----tactagtcctccagggaaatcattagctttgtctgtataagcaagtcctgccatatctaagcatctgatttcagctgcartatatgctact-actgatgtgatttggttgtgaatttttgggactcagacttgcctgcctgtaaagcatcgttcagggaacaagcaactggattattttaagtccttgtgttgtcttttatgactgatgtgtttgtctgtatttttattccccaggaat

Otus_megalotis_CHIMERA cctattgttttaggtaactatgcatccgtattcagtatacttacatagtagtaccagcctgtgaggtgtaatgcagtttcttatcatgttgctttacggttggcgtttatacagagcgcggaccctcatttgcagacccacggggtctgtgtgcagcaggtatctgcccagtggtctccagccctgcagagacagtggcagatgaggaaggagggttgtggccttgggaagcagaagctgagttccatctgggatgattcccagacccatcagcctcataagtacagccgaattatcacctccactgagctcatgtagtcagaggccacttggtaggacctgaaaaataatacttactagtcctccagggaaaccattagctttgtctgtataagcaagtcccgccatatctaagcatctgatttcagctgcagtatatgctact-attgatgtgatttggttgtgaatttttgggactcagacttgcctgcctgtaaagcaccattcagggaacaagcaactggattattttaagtccttgtgttgtcttttatgactgatgtgtttgtctgtatttttattccccaggaat

Otus_nigrorum_CHIMERA -----------------------------------------------------------------------------------------------------------------------------------------------------------------------------------------------------------------------------------------------------------------------------------------------------------------------------------------------------------------------------------------------------------------------------------------------------------------------------------------------------------------------------------------------------------------------------------------------------------------

Otus_everetti_CHIMERA -----------------------------------------------------------------------------------------------------------------------------------------------------------------------------------------------------------------------------------------------------------------------------------------------------------------------------------------------------------------------------------------------------------------------------------------------------------------------------------------------------------------------------------------------------------------------------------------------------------------

Otus_semitorques_CHIMERA cctattgttttaggtaactatgcatccgtattcagtatacttacatagtagtaccagcctgtgaggtgtaatgcagtttcttatcatgttgctttacggttggcgtttatacagagtgcggaccctcatttgcagacccacggggtctatgtgcagcaggtatctgcccagtggtctccagccctgcagagacagtggcagatgaggaaggagggttgtggccttgggaagcagaagctgagttccatctgggatgattcccagacccatcagcctcataagtacagccgagttatcacctccactgagctcatgtagtcagaggccacttggtaggacctgaaaaataatacttactagtcctccagggaaaccattagctttgtctgtattagcaagtcccgccatatctaagcatctgatttcagctgcagtatatgctact-attgatgtgatttggttgtgaatttttgggactcagacttgcctgcctgtaaagcaccattcagggaacaagcaactggattattttaagtccttgtgttgtcttttatgactgatgtgtttgtctgtatttttattccccaggaat

Otus_angelinae_29 -----------------------------------------------------------------------------------------------------------------------------------------------------------------------------------------------------------------------------------------------------------------------------------------------------------------------------------------------------------------------------------------------------------------------------------------------------------------------------------------------------------------------------------------------------------------------------------------------------------------

Otus_spilocephalus_vandewateri_97 -----------------------------------------------------------------------------------------------------------------------------------------------------------------------------------------------------------------------------------------------------------------------------------------------------------------------------------------------------------------------------------------------------------------------------------------------------------------------------------------------------------------------------------------------------------------------------------------------------------------

Otus_spilocephalus_vulpes_96 -----------------------------------------------------------------------------------------------------------------------------------------------------------------------------------------------------------------------------------------------------------------------------------------------------------------------------------------------------------------------------------------------------------------------------------------------------------------------------------------------------------------------------------------------------------------------------------------------------------------

Otus_spilocephalus_spilocephalus_90 cctattgttttaggtaactatgcatccgtattcagtatacttacatagtagtaccagcctgtgaggygtaatgcagtttcttatcatgttgctttacggttggcgtttatacagagcgcggaccctcattttcagacccacagggtctgtgtgcagcaggtatctgcccggtggtctccagtcctgcagagacagtggcagatgaggaaggagggttgtggccttgggaagcagaagctgagttccatctgggatgattcccagacccatcagcctcataagtacagccaaattrccacctccactgagctcatgtagtcagaggccacttggtaggacctgaasaataatacttactagtcctccagggaaaccattagctttgtctgtataagcaagtcctgccatatctaagcatctgatttcagctgcagtatatgctcct-attgatgtgatttggttgtraatttttgggactcagacttgcctgcctgtaaagcatcattcagggaacaagcaactggattattttaagtccttgtgttgtcttttatgactgatgtgtttgtctgtatttttattccccaggaat

Otus_spilocephalus_hambroecki_92 -----------------------------------------------------------------------------------------------------------------------------------------------------------------------------------------------------------------------------------------------------------------------------------------------------------------------------------------------------------------------------------------------------------------------------------------------------------------------------------------------------------------------------------------------------------------------------------------------------------------

_Otus_spilocephalus_latouchi_CHIMERA -----------------------------------------------------------------------------------------------------------------------------------------------------------------------------------------------------------------------------------------------------------------------------------------------------------------------------------------------------------------------------------------------------------------------------------------------------------------------------------------------------------------------------------------------------------------------------------------------------------------

Otus_hartlaubi_13 cctattgttttaggtaactatgcatccgtattcagtatacttacatagtagtaccagcctgtgaggtgtaatgcagtttcttatcacgttgctttacggttggtgtttatacagagcgcggaccctcatttgyagacccacggggtctgtgtgcagcaggtatctgcccagtggtctccagtcctgcagagacagtggcagatgaggaaggagggttgtggccttgggaagcagaagctgagttccatctgggatgattcccagacccatcagcctcataagtacagccgaattaccacctccactgagctcatgtagtcagaggctacttggtaggacctgaagattaatacttactagtcctccagggaaaccattagctttgtctgtataagcaagtcctgccatatgtaagcatctgatttcagctgcagtatatgctact-attgatgtgatttggttgtgaatttttgggactcagacttgcctgcctgtaaagcatcattcagggaacaagcagctggattattttaagtccttctgttgtcttttatgactgatgtgtttgtctgtatttttattccccaggaat

Otus_hartlaubi_15 ---------------------------gtattcagtatacttacatagtagtaccagcctgtgaggtgtaatgcagtttcttatcacgttgctttacggttggtgtttatacagagcgcggaccctcatttgcagacccacggggtctgtgtgcagcaggtatctgcccagtggtctccagtcctgcagagacagtggcagatgaggaaggagggttgtggccttgggaagcagaagctgagttccatctgggatgattcccagacccatcagcctcataagtacagccgaattaccacctccactgagctcatgtagtcagaggctacttggtaggacctgaagattaatacttactagtcctccagggaaaccattagctttgtctgtataagcaagtcctgccatatgtaagcatctgatttcagctgcagtatatgctact-attgatgtgatttggttgtgaatttttgggactcagacttgcctgcctgtaaagcatcattcagggaacaagcagctggattattttaagtccttctgttgtcttttatgactgatgtgtttgtctgtatttttattccccaggaat

Otus_hartlaubi_16 ---------------------------gtattcagtatacttacatagtagtaccagcctgtgaggtgtaatgcagtttcttatcacgttgctttacggttggtgtttatacagagcgcggaccctcatttgcagacccacggggtctgtgtgcagcaggtatctgcccagtggtctccagtcctgcagagacagtggcagatgaggaaggagggttgtggccttgggaagcagaagctgagttccatctgggatgattcccagacccatcagcctcataagtacagccgaattaccacctccactgagctcatgtagtcagaggctacttggtaggacctgaagattaatacttactagtcctccagggaaaccattagctttgtctgtataagcaagtcctgccatatgtaagcatctgatttcagctgcagtatatgctact-attgatgtgatttggttgtgaatttttgggactcagacttgcctgcctgtaaagcatcattcagggaacaagcagctggattattttaagtccttctgttgtcttttatgactgatgtgtttgtctgtatttttattccccaggaat

Otus_hartlaubi_14 ---------------------------------------cttacatagtagtaccagcctgtgaggtgtaatgcagtttcttatcacgttgctttacggttggtgtttatacagagcgcggaccctcatttgcagacccacggggtctgtgtgcagcaggtatctgcccagtggtctccagtcctgcagagacagtggcagatgaggaaggagggttgtggccttgggaagcagaagctgagttccatctgggatgattcccagacccatcagcctcataagtacagccgaattaccacctccactgagctcatgtagtcagaggctacttggtaggacctgaagattaatacttactagtcctccagggaaaccattagctttgtctgtataagcaagtcctgccatatgtaagcatctgatttcagctgcagtatatgctact-attgatgtgatttggttgtgaatttttgggactcagacttgcctgcctgtaaagcatcattcagggaacaagcagctggattattttaagtccttctgttgtcttttatgactgatgtgtttgtctgtatttttattccccaggaat

Otus_feae_45 ------------------------------ttcagtatacttacatagtagtaccagcctgtgaggtgtcatgcggtttcttatcacggtgctttacggttggtgtttatacagagcgcggaccctcatttgcagacccatggggkctgtgtgcagcaggtatctgcccagtggtctccagtcctgcagagacagtggcagatgaggaaggagggttgtggccttgggaagcagaagctgagttccatctgggatgattcccagacccatcagcctcataagtacagccgaattaccacctccactgagctcatgtagtcagaggccacttggtaggacctgaagagtaatacttactagtcctccagggaaaccattagctttgtctgtataagcaagtcctgccatatctaagcatctgatttcagctgcactatatgctactaattgatgtgatttggttgtgaatttttgggactcasacttgcctgcctgtaaagcatcattcagggaacaagcagctggattattttaagtccttctgttgtcttttatgactgatgtgtttgtctgtatttttattccccargaat

Otus_senegalensis_17 cctattgttttaggtaactatgcatccgtattcagtatacttacatagtagtaccagcctgtgaggtgtcatgcggtttcttatcacggtgctttacggttggtgtttatacagagcgcggaccctcatttgcagacccatggggtctgtgtgcagcaggtatctgcccagtggtctccagtcctgcagagacagtggcagatgaggaaggagggttgtggccttgggaagcagaagctgagttccatctgggatgattcccagacccatcagcctcataagtacagccgaattaccacctccactgagctcatgtagtcagaggctacttggtaggacctgaagagtaatacttactagtcctccagggaaaccattagctttggctgtataagcaagtcctgccatatctaagcatctgatttcagctgcagtatatgctact-attgatgtgatttggttgtgaatttttgggactcagacttgcctgcctgtaaagcatcattcagggaacaagcagctggattattttaagtccttctgttgtcttttatgactgatgtgtttgtctgtatttttattccccaggaat

Otus_pamelae_74 -----------------------------------------------------------------------------------------------------------------------------------------------------------------------------------------------------------------------------------------------------------------------------------------------------------------------------------------------------------------------------------------------------------------------------------------------------------------------------------------------------------------------------------------------------------------------------------------------------------------

Otus_sp_nov_1 ------------------------------------------acatagtagtaccagcctgtgaggtgtcatgcggtttcttatcacggtgctttacggttggtgtttatacagagcgcrgaccctcatttgcagacccatggggtctgtgtgcagcaggtatctgcccagtggtctccagtcctgcagagacagtggcagatgaggaaggagggttgtakccttgggaagcagaagctgagttccatctgggatgattcccagacccatcagcctcataagtacagccgaattaccacctccactgagctcatgtagtcagaggctacttggtaggacctgaagagtaatacttactagtcctccagggaaaccattagctttgtctgtataagcaagtcctgccatatctaagcatctgatttcagctgcagtatatgctact-attgatgtgatttggttgtgaatttttgggactcagacttgcctgcctgtaaagcatcattcagggaacaagcagctggattattttaagtccttctgttgtcttttatgactgatgtgtttgtctgtatttttattccccaggaat

Otus_sp_nov_2 ---------------------------gtattcagtatacttacatagtagtaccagcctgtgaggtgtcatgcggtttcttatcacggtgctttacggttggtgtttatacagagcgcggaccctcatttgcagacccatggggtctgtgtgcagcaggtatctgcccagtggtctccagtcctgcagagacagtggcagatgaggaaggagggttgtagccttgggaagcagaagctgagttccatctgggatgattcccaracccatcagcctcataagtacagccraattaccacctccactgagctcatgtagtcrgaggctacttggtaggacctgaagagtaatacttactagtcctccagggaaaccattagctttgtctgtataagcaagtcctgccatatctaagcatctgatttcagctgcagtatatgctact-attgatgtgatttggttgtgaatttttgggactcagacttgcctgcctgtaaagcatcattcagggaacaagcagctggattattttaagtccttctgttgtcttttatgactgatgtgtttgtctgtatttttattccccaggaat

Otus_sp_nov_3 ---------------------------gtattcagtatacttacatagtagtaccagcctgtgaggtgtcatgcggtttcttatcacggtgctttacggttggtgtttatacagagcgcggaccctcatttgcagacccatggggtctgtgtgcascrggtatctgcccagwggtctccagtcctgcagagacagtggcagatgaggaaggagggttgtagccttgggaagcagaagctgagttccwtctgggatgattcccagacccatsagcctcwkaagtacagscgaattaccacctccactgagctcatgwrgtcrgaggctacktggtasgacctgaagagtaatacttactagtcctccagggaaaccattakctttgtctgtatragcaagtcctgccatrtctragcatctgatttcagctgcagtatatgctact-attgatgtgatttggttgtgaatttttgggactcagacttgcctgcctgtaaagcatcattcagggaacaagcagctggattattttaagtscttctgytgtcttttatgactgatgtgtttgtctgtatttttattccccaggaat

Otus_sp_nov_4 ---------------------------gtattcagtatacttacatagtagtaccakcctgtgaggtgtcatgcggtttcttatcacggtgctttacggttggtgtttatacagascgcggaccctcatttgcagacccatggggtctgtgtgcascrggtatctgcccagwggtctccagtcctgcagagacagtggcagatgaggaaggagggttgtagccttgggaagcagaagctgagttccwtctgggatgattcccagacccatsascctcwkaagtacagscgaattaccacctccactgagctcatgwrgtcagaggctacktggwasgacctgaagagwaatacttactagtyctccagggaaaccattakctttgtctgtatragcaagtcctgccatrtctragcatctgatttcagctgcagtatatgctact-attgatgtgatttggttgtgaatttttgggactcagacttgcctgcctgtaaagcatcattsagggaacaagcagctggattattttargtscttctgytgtcttttatgactgatgtgtttgtctgtatttttattccccaggaat

Otus_scops_5 cctattgttttaggtaactatgcatccgtattcagtatacttacatagtagtaccagcctgtgaggtgtcatgcagtttcttatcatgttgctttacggttggtgtttatacagagcgcggaccctcatttgcagacccatggggtctgtgtgcagcaggtatctgcccagtggtctccagtcctgcagagacagtggcagatgaggaaggagggttgtggccytgggaagcagaagctgagttccatctgggatgattcccagacccatcagcctcataagtacagccgaattaccacctccactgagctcatgtagtcagaggccacttggtaggacctgaagagtaatacttactagtcctccagggaaaccattagctttgtctgtataagcaagtcctgccatatctaagcatctgatttcagctgcagtatatgctact-attgrtgtgatttggttgtgaatttttgggactcagacttgcctgcctgtaaagcatcattcagggaacaagcagctggattattttaagtccttgtgttgacttttatgactgatgtgtttgtctgtatttttattccccaggaat

_Otus_scops_JF5337_F -----------------------------------------------------------------------------------------------------------------------------------------------------------------------------------------------------------------------------------------------------------------------------------------------------------------------------------------------------------------------------------------------------------------------------------------------------------------------------------------------------------------------------------------------------------------------------------------------------------------

Otus_pembaensis_24 cctattgttttaggtaactatgcatccgtattcagtatacttacatagtagtaccagcctgtgaggtgtcatgcggtttcttatcacggtgctttacggttggtgtttatacagagcgcggaccctcatttgcagacccatggggtctstgtgcagcaggtatctgcccagtggtctccagtcctgcagagacagtggcagatgaggaaggagggttgtggccttgggaagcagaagctgagttccatctgggatgattcccagacccatcagcctcataagtacagccgaattaccacctccactgagctcatgtagtcagaggccacttggtaggacctgaagagtaatacttrctagtcctccagggaaaccattagctttgtttgtataagcaagtcctgccatatctaagcatctgatttcagctgcagtatatgctactaattgatgtgatttggttgtgaatttttgggactcagacttgcctgcctgtaaagcatcattcagggaacaagcagctggattattttaagtccttgtgttgtcttttatgactgatgtgtttgtctgtatttttattccccaggaat

Otus_pembaensis_25 cctattgttttaggtaactatgcatccgtattcagtatacttacatagtagtaccagcctgtgaggtgtcatgcggtttcttatcacggtgctttacggttggtgtttatacagagcgcggaccctcatttgcagacccatggggtctstgtgcagcaggtatctgcccagtggtctccagtcctgcagagacagtggcagatgaggaaggagggttgtggccttgggaagcagaagctgagttccatctgggatgattcccagacycatcagcctcataagtacagccgaattaccacctccactgagctcatgtagtcagaggccacttggtaggacctgaagagtaatacttactagtcctccagggaaaccattagctttgtttgtataagcaagtcctgccatatctaagcatctgatttcagctgcagtatatgctactaattgatgtgatttggttgtgaatttttgggactcagacttgcctgcctgtaaagcatcattcagggaacaagcagctggattattttaagtccttgtgttgtcttttatgactgatgtgtttgtctgtatttttattccccaggaat

_Otus_longicornis_CHIMERA cctattgttttaggtaactatgcatccgtattcagtatacttacatagtagtaccagcctgtgaggtgtcatgcagtttcttatcatgttgctttacagttggtgtttatacagagcgcggaccctcatttgcagacccatggggtctgtgtgcagcaggtatctgcccagtggtctccagtcctgcagagacagtggcagatgaggaaggagggttgtggccttgggaagcagaagctgagttctatctgggatgattcccagacccatcagcctcataagtacagttgaattaccacctccactgagctcatgtagtcagaggccacttggtaggacctgaagaataatacttactagtcctccagggaaaccattagctttgtctgtataagcaagtcctgccatatctaagcatctgatttcagctgcagtatatgctact-attgatgtgatttggttgtgaatttttgggactcagacttgcctgcctgtaaagcatcattcagggaacaagcagctggattattttaagtccttgtgttgtcttttatgactgatgtgtttgtctgtatttttattccccaggaat

Otus_mirus_CHIMERA cctattgttttaggtaactatgcatccgtattcagtatacttacatagtagtaccagcctgtgaggtgtcatgcagtttcttatcatgttgctttacrgttggtgtttatacagagcgcggaccctcatttgcagacccatggggtctgtgtgcagcaggtatctgcccagtggtctccagtcctgcagagacagtggcagatgaggaaggagggttgtggccttgggaagcagaagctgagttctatctgggatgattcccagacccatcagcctcataagtacagttgaattaccacctccactgagctcatgtagtcagaggccacttggtaggacctgaagaataatacttactagtcctccagggaaaccattagctttgtctgtataagcaagtcctgccatatctaagcatctgatttcagctgcagtatgtgctact-attgatgtgatttggttgtgaatttttgggactcagacttgcctgcctgtaaagcatcattcagggaacaagcagctggattattttaagtccttgtgttgtcttttatgactgatgtgtttgtctgtatttttattccccaggaat

Otus_mayottensis_60 cctattgttttaggtaactatgcatccgtattcagtatacttacatagtagtaccagcctgtgaggtgtcatgcagtttcttatcacgttgctttacggttggtgtttatacagagcgcggaccctcatttgcagacccacggggtctgtgtgcagcaggtatctgcccagtggtctccagtcctgcagagacagtggcagatgaggaaggagggttgtggccttgggaagcagaagctgagttccatctgggatgattcccagacccatcagcctcataagtacagccgaattaccacctccactgagctcatgtagtcagaggccacttggtaggacctgaagaataatacttactagtcctccagggaaaccattagctttgtctgtataagcaagtcctgccatatctaagcatctgatttcagctgcagtatatgctact-attgatgtgatttkgttgtgaatttttgggactcagacttgcctgcctgtaaagcatcattcagggaacaagcagctggattattttaagtccttgtgttgtctttgatgactgatgtgtttgtctgtattt---------------

Otus_capnodes_40 cctattgttttaggtaactatgcatccgtattcagtatacttacatagtagtaccagcctgtgaggtgtcatgcagtttcgtatcacgttgctttacggttggtgtttatacagagcgcggaccctcatttgcagacccacggggtctgtgtgcagcaggtatctgcccagtggtctccagtcctgcagagacagtggcagatgaggaaggagggttgtggccttgggaagcagaagctgagttccatctgggatgattcccagacccatcagcctcataagtacagccgaattaccacctccactgagctcatgtagtcagaggccacttggtaggacctgaagaataatacttactagtcctccagggaaaccattagctttgtctktataagcaagtcctgccatatctaagcatctgatttcagctgcagtatatgctact-attgatgtgatttggttgtgaatttttgggactcagacttgcctgcctgtaaagcatcattcagggaacaagcagctggattattttaagtccttgtgttgtctttgatgactgatgtgtttgtctgtatttttattccccaggaat

_Otus_madagascariensis_CHIMERA cctattgttttaggtaactatgcatccgtattcagtatmcttacatagtagtaccagcctgtgaggtgtcatgcagtttcttatcacgttgctttacggttggtgtttatacagagcgcggaccctcatttgcagacccacggggtctgtgtgcagcaggtatctgcccagtggtctccagtcctgcagagacagtggcagatgaggaaggagggttgtggccttgggaagcagaagctgagttccatctgggatgattcccagacccatcagcctcataagtacagccgaattaccacctccactgagctcatgtagtcagaggccacttggtaggacctgaagaataatacttactagtcctccagggaaaccattagctttgtctgtataagcaagtcctgccatatctaagcatmtgatttcagctgcagtatatgctact-attgatgtgatttggttgtgaatttttgggactcagacttgcctgcctgtaaagcatcattcagggarcaagcagctggattattttaagtccttgtgttgtctttgatgactgatgtgtttgtctgtatttttattccccaggaat

Otus_pauliani_76 cctattgttttaggtaactatgcatccgtattcagtatacttacatagtagtaccagcctgtgaggtgtcatgcagtttcttatcacgttgctttacggttggtgtttatacagagcgcggaccctcatttgcaggcccatagggtctgtgtgcagcaggtatctgcccagtggtctccagtcctgcagagacagtggcagatgaggaaggagggttgtggccttgggaagcagaagctgagtaccatctgggatgattcccagacccatcagcctcataagtacagccgaattaccacctccactgagctcatgtagtcagaggccacttggtaggacctgaagaataatacttactagtcctccagggaaaccattagctttgtctgtataagcaagtcctgccatatctaagcatctgatttcagctgcagtatatgctact-attgatgtgatttggttgtgaatttttgggactcagacttgcctgcctgtaaagcatcattcagggaacaagcagctggattattttaagtccttgtgttgtcttttatgactgatgtgtttgtctgtattttt-------------

Otus_insularis_47 cctattgttttaggtaactatgcatccgtattcagtatacttacatagtagtaccagcctgtgaggtgtcatgcagtttcttatcatgttgctttacggttggtgtttatacagagcgcggaccctcatttgcagacccacggggtctgtgtgcagcaggtatctgcccagtggtctccagtcctgcagagacagtggcagatgaggaaggagggttgtggccttgggaagcagaagctgagttccatctgggatgattcccagacccatcagcctcataagtacagctgaattaccacctccactgagctcatgtagtcagaggccacttggtaggacctgaagaataatacttactagtcctccagggaaaccattagctttgtctgtataagcaagtcctgccatatctaagcatctgatttcagctgcagtatatgctact-attgatgtgatttggttgtgaatttttgggactcagacttgcctgcctgtaaagcatcattcagggaacaagcagctggattattttaagtccttgtgttgtctttgatgactgatgtgtttgtctgtatttttattccccaggaat

Otus_socotranus_89 -ctattgttttaggtaactatgcatccgtattcagtatacttacatagtagtaccagcctgtgaggtgtcatgcagtttcttatcacgttgctttacggttggtgtttgtacagagcgcggaccctcatttgcagacccacggggtctgtgtgcagcaggtatctgcccagtggtctccagtcctgcagagacagtggcagatgaggaaggagggttgtggccttgggaagcagaagctgagttccatctgggatgattcccagacccatcagcctcataagtacagctgaattaccacctccactgagctcatgtagtcagaggccacttggtaggacctgaagaataatacttactagtcctccagggaaaccattagctttgtctgtataagcaagtcctgccatatctaagcatctgatttcagctgcagtatatgctact-attgatgtgatttggttgtgaatttttgggactcagacttgcctgcctgtaaagcatcattcagggaacaagcagctggattattttaagtccttgtgttgtctttgatgactgatgtgtttgtctgtatttttattccccaggaat

_Otus_sunia_CHIMERA cctattgttttaggtaactatgcatccrtattcagtatacttacatagtagtaccagcctgtgaggtgtcatgcagtttcttatcacgttgctttacggttggtgtttatacagagcgcggaccctcatttgcagacccayggggtctgtgtgcagcaggtatctgcccagtggtctccagtcctgcagagacagtggcagatgaggaaggagggttgtggccttgggaagcagaagctgagttccatctgggatgattcccagacccatcagcctcataagtacagctgaattaccacctccactgagctcatgtagtcagaggccacttggtaggacctgaagaataatacttactagtcctccagggaaaccattagctttgtctgtataagcaagtcctgccatatctaagcatctgatttcagctgcagtatatgctact-attgatgtgatttggttgtgaatttttgggactcagacttgcctgcctgtaaagcatcattcagggaacaagcagctggattattttaagtccttgtgttgtctttgatgactgatgtgtttgtctgtatttttattccccaggaat

Otus_moheliensis_CHIMERA ---------------------------gtattcagtatacttacatagtagtaccagcctgtgaggtgtcatgcagtttcttatcatgttgctttacggttggtgtttatacagagcgcggaccctcatttgcagacccacggggtctgtgtgcagcaggtatctgcccagtggtctccagtcctgcagagacagtggcagatgaggaaggagggttgtggccttgggaagcagaagctgagttccatctgggatgattcccagacccatcagcctcataagtacagccaaattaccacctccactgagctcatgtagtcagaggccacttggtaggacctgaagaataatacttactagtcctccagggaaaccattagctttgtctgtataagcaagtcctgccatatctaagcatctgatttcagctgcagtatatgctact-attgatgtgatttggttgtgaatttttgggactcagacttgcctgcctgtaaagcatcattcagggaacaagcagctggattattttaagtccttgtgttgtcttttatgactgatgtgtttgtctgtatttttattccccaggaat

Otus_brucei_CHIMERA -----------------------------------------------------------------------------------------------------------------------------------------------------------------------------------------------------------------------------------------------------------------------------------------------------------------------------------------------------------------------------------------------------------------------------------------------------------------------------------------------------------------------------------------------------------------------------------------------------------------

Otus_icterorhynchus_holerythrus_27 -----------------------------------------------------------------------------------------------------------------------------------------------------------------------------------------------------------------------------------------------------------------------------------------------------------------------------------------------------------------------------------------------------------------------------------------------------------------------------------------------------------------------------------------------------------------------------------------------------------------

Otus_icterorhynchus_CHIMERA -----------------------------------------------------------------------------------------------------------------------------------------------------------------------------------------------------------------------------------------------------------------------------------------------------------------------------------------------------------------------------------------------------------------------------------------------------------------------------------------------------------------------------------------------------------------------------------------------------------------

Otus_ireneae_49 cctattgttttaggtaactatgcatccgtattcagtatacttacgtagtagtaccagcctgtgaggtgtaatgcagtttcttatcatgtttctttacggttggcatttatacag-------accctcatttgcagacccacggggtctgtgtgcagcaggtatctgcccagtggtctccagtcctgcagagacagtggcagatgaggaaggagggttgtggccttgggaagcagaagctgagttscatctgggatgattcccagacccatcagcctcataagtacagccgaattatcacctccactgagctcatgtagtcagaggccacttggtaggacctgaagaataatacttactagtcctccagggaaaccattagctttgtctgtataagcaagtcctgccatgtctaagcatctgatttcagctgcagtatatgctact-attgatgtgatttggttgtgaatttttgggactcagacttgcctgcctgtaaagcatcattcagggaacaagcaactggattattttaagtccttgtgttgtcttttatgactgatgtgtttgtctgtaattttattccccaggaat

Otus_rutilus_78 cctattgttttaggtaactatgcatccgtattcagtatacttacatagtagtaccagcctgtgaggtgtcatgcagtttcttatcacgttgctttacggttggtgtttatacagagcgcggaccctcatttgcagacccacggggtctgtgtgcagcaggtatctgcccagtggtctccagtcctgcagagacagtggcagatgaggaaggagggttgtggccttgggaagcagaagctgagttccatctgggatgattcccagacccatcagcctcataagtacagccgaattaccacctccactgagctcatgtagtcagaggccacttggtaggacctgaagaataatacttactagtcctccagggaaaccattagctttgtctgtataagcaagtcctgccatatctaagcatctgatttcagctgcagtatatgctact-attgatgtgatttggttgtgaatttttgggactcagacttgcctgcctgtaaagcatcattcagggaacaagcagctggattattttaagtccttgtgttgtctttgatgactgatgtgtttgtctgtatttttattccccaggaat

Otus_scops_scops_8 ---------------------------gtattcagtatacttacatagtagtaccagcctgtgaggtgtcatgcagtttcttatcacgttgctttacggttggtgtttatacagagcgcggaccctcatttgcagacccacggggtctgtgtgcagcaggtatctgcccagtggtctccagtcctgcagagacagtggcagatgaggaaggagggttgtggccytgggaagcagaagctgagttccatctgggatgattcccagacccatcagcctcataagtacagccgaattaccacctccactgagctcatgtagtcagaggccacttggtaggacctgaarartaatacttactagtcctccagggaaaccattagctttgtctgtataagcaagtcctgccatatctaagcatctgatttcagctgcagtatatgctact-attgrtgtgatttggttgtgaatttttgggactcagacttgcctgcctgtaaagcatcattcagggaacaagcagctggattrttttaagtccttgtgttgtcttttatgactgatgtgtttgtctgtatttttattccccaggaat

Otus_scops_scops_9 ---------------------------gtattcagtatacttacatagtagtaccagcctgtgaggtgtcatgcagtttcttatcacgttrctttacggttggtgtttatacagagcgcggaccctcatttgcagacccayggggtctgtgtgcagcaggtatctgcccagtggtctccagtcctgcagagacagtggcagatgaggaaggagggttgtggccctgggaagcagaagctgagttccatctgggatgattcccagacccatcagcctcataagtacagccgaattaccacctccactgagctcatgtagtcagaggccacttggtaggacctgaagagtaatacttactagtcctccagggaaaccattagctttgtctgtataagcaagtcctgccatatctaagcatctgatttcagctgcagtatatgctact-attggtgtgatttggttgtgaatttttgggactcagacttgcctgcctgtaaagcatcattcagggaacaagcagctggattattttaagtccttgtgttgacttttatgactgatgtgtttgtctgtatttttattccccaggaat

Otus_spilocephalus_luciae_94 -----------------------------------------------------------------------------------------------------------------------------------------------------------------------------------------------------------------------------------------------------------------------------------------------------------------------------------------------------------------------------------------------------------------------------------------------------------------------------------------------------------------------------------------------------------------------------------------------------------------

Otus_silvicola_87 -----------------------------------------------------------------------------------------------------------------------------------------------------------------------------------------------------------------------------------------------------------------------------------------------------------------------------------------------------------------------------------------------------------------------------------------------------------------------------------------------------------------------------------------------------------------------------------------------------------------

_Otus_cyprius_CHIMERA -----------------------------------------------------------------------------------------------------------------------------------------------------------------------------------------------------------------------------------------------------------------------------------------------------------------------------------------------------------------------------------------------------------------------------------------------------------------------------------------------------------------------------------------------------------------------------------------------------------------

Otus_elegans_CHIMERA -----------------------------------------------------------------------------------------------------------------------------------------------------------------------------------------------------------------------------------------------------------------------------------------------------------------------------------------------------------------------------------------------------------------------------------------------------------------------------------------------------------------------------------------------------------------------------------------------------------------

[marker: myo2; length: 721; no. of seqs: 51; new coordinates: 3843-4563)]

Bubo_bubo_111 ----tatctggaggtatggaaaagggcagggaatcctgctgcctgatgtgtagtgaacgtgtgcaagacagctatgtgagagctgtgcttttatttactgatggctagttggacttcagtg-agctctccctcaagtccaaggtctctctgtacaggcaggaggagacacagaaagggctcatggtatgcgaatggtatgtgaatatccaagtttagatttcccattcctaacaccacatgcagtctgagcaacccttgacaataataaaccagcccaggcagcctctgcatgcctgggaaactacattacataacaactgtcagtggctggacacaagggacatacaattttagagtaagccctggaggatccattggagaccaagacccacaaaactaagtgttgtacaaacacatggcatgagttttcgaccttaatggatgaggcagacaaagagtgggaagggccatggtctactcaaggtcatgaagcagataagcatcagagctaggaatagagcccagttgttctgtccagcccagactccttgcatgctagagacctccctgcccctccagagactgtgggaagggctactgaattagctcagagatatttccaggctctgcaacctatttcttaacctg-ttttttctgaaaataaaccttgagtgtccgttcagtt-ttctctctctccttccttcctcacagttcattt

Strix_aluco_129 ----tatctggaggtatggaaaagggcagggaatcctgctgcctgatgtgtagtgaatgtgtgcaagacagctatgtgagagctgtgcttttatttactgatggctagttggacttcagtg-agctctccctcaagtccaaggtctctctgtacaggcaggaggagacacagaaagggctcatggtatgcgaatggtatgtgaatatccaagtttagatttcccattcctaacaccacatgcagtctgagcaacccttgacaataataaaccagcccaggcagcctctgcatgcctgggaaactacattacataacaactgtcagtggctggacacgagggacatacaattttagagtaagccctggaggatccattggagaccaagacccacaaaactaagtgttgcacaaacacagggcatgagtttttgaccttaagggatgaggcagacaaagactgggaagggccatggtctactcaaggtcatgaagcagataagcatcagagctaggaatagagcccagttgttctgcccagcccagactccttgcatgctagagacctccctgcccctccagagactgtgggaagggctactgaattagctcagagatatttccaggctctgcaacctatttattaacctg-ttttttctgaaaatataccttgagtgtccgttcagtt-ttctctctctccttccttcctcacagttcattt

Otus_bakkamoena_marathae_35 -------------------------------------------------------------------------------------------------------------------------------------------------------------------------------------------------------------------------------------------------------------------------------------------------------------------------------------------------------------------------------------------------------------------------------------------------------------------------------------------------------------------------------------------------------------------------------------------------------------------------------------------------------------------------------------------------------------------------------------------------

_Otus_lempiji_CHIMERA caaatatctggaggtatggaaaagggcagggaatcctgctgcctgatgtgtagtgaatgtgtgcaagacagctatgcgagagctgtgcttttatttactgatggctggttggacttcagtg-agctcttcctcaagtctaaggtctctctgtacaggcaggaggagacacagaaagggctcatggtatgcgaatggtatgtgaatatccaagtttagatttcccattcctaacaccacatgcagtctgagcaacccttgacaataataaaccagcccaggcagcctctgcatgcctgggaaactacattacataacaactgtcagtggctggacacaagggacatacaattttagagtaagccctggaggatccattggagaccaagacccacaaaactaagtgttgtacaaacacagggcatgagttttcgaccttaatggatgaggcagacaaagagtgggaagggccatggtctactcaaggtcatgaagcagataagtgtcagagctaggaatagagcccagttgttctgcccagctgagactccttgcatgctagagacctccttgcccctccagagactgtgggaagggctactgaattagctcagagatatttccaggctctgcaacctatttcttaacctg--tttttctgaaaataaaccttgagtgtcccttcagtttttctctctctccttccttcctcacagttcattt

Otus_lettia_lettia_CHIMERA caaatatctggaggtatggaaaagggcagggaatcctgctgcctgatgtgtagtgaatgtgtgcaagacagctatgcgagagctgtgcttttatttactgatggctggttggacttcagtg-agctcttcctcaagtctaaggtctctctgtacaggcaggaggagacacagaaagggctcatggtatgcgaatggtatgtgaatatccaagtttagatttcccattcctaacaccacatgcagtctgagcaacccttgacaataataaaccagcccaggcagcctctgcatgcctgggaaactacattacataacaactgtcagtggctggacacaagggacatacaattttagagtaagccctggaggatccattggagaccaagacccacaaaactaagtgttgtacaaacacagggcatgagttttcgaccttaatggatgaggcagacaaagagtgggaagggccatggtctactcaaggtcatgaagcagataagtgtcagagctaggaatagagcccagttgttctgcccagctgagactccttgcatgctagagacctccttgcccctccagagactgtgggaagggctactgaattagctcagagatatttccaggctctgcaacctatttcttaacctgtttttttctgaaaataaaccttgagtgtcccttcagtttttctctctctccttccttcctcacagttcattt

Otus_megalotis_CHIMERA caaatatctggaggtatggaaaagggcagggaatcctgctgcctgatgtgtagtgaatgtgtgcaagacagctatgcgagagctgtgcttttatttactgatggctagttggacttcagtg-agctctccctcaagtctaaggtctctctgtacaggcaggaggagacacagaaagggctcatggtatgcgaatggtatgtgaatatccaagtttagatttcccattcctaacaccacatgcagtctgagcaacccttgacaataataaaccagcccaggcagcctctgcatgcctgggaaactacattacataacaactgtcagtggctggacacaagggacatacaattttagagtaagccctggaggatccattggagaccaagacccacaaaactaagtgttgtacaaacacagggcatgagttttcgaccttaatggacgaggcagacaaagagtgggaagggccatggtctactcaaggtcatgaagcagataagcgtcagagctaggaatagagcccagctgttctgcccagccgagactccttgcatgctagagacctccttgcccctccagagactgtgggaagggctactgaattagctcagagatatttccaggctctgcaacctatttcttaacctg--tttttctgaaaataaaccttgagtgtcccttcagtttttctctctctccttccttcctcacagttcattt

Otus_nigrorum_CHIMERA -------------------------------------------------------------------------------------------------------------------------------------------------------------------------------------------------------------------------------------------------------------------------------------------------------------------------------------------------------------------------------------------------------------------------------------------------------------------------------------------------------------------------------------------------------------------------------------------------------------------------------------------------------------------------------------------------------------------------------------------------

Otus_everetti_CHIMERA -------------------------------------------------------------------------------------------------------------------------------------------------------------------------------------------------------------------------------------------------------------------------------------------------------------------------------------------------------------------------------------------------------------------------------------------------------------------------------------------------------------------------------------------------------------------------------------------------------------------------------------------------------------------------------------------------------------------------------------------------

Otus_semitorques_CHIMERA caaatatctggaggtatggaaaagggcagggaatcctgctgcctgatgtgtagtgaatgtgtgcaagacagctatgcgagagctgtgcttttatttactgatggctagttggacttcagtg-agctctccctcaagtctaaggtctctctgtacaggcaggaggagacacagaaagggctcatggtatgcgaatggtatgtgaatatccaagtttagatttcccattcctaacaccacatgcagtctgagcaacccttgacaataataaaccagcccaggcagcctctgcatgcctgggaaactacattacataacaactgtcagtggctggacacaagggacatacaattttagagtaagccctggaggatccatttgagaccaagacccacaaaactaagtgttgtacaaacacagggcatgagttttcgaccttaatggacgaggcagacaaagagtgggaagggccatggtctactcaaggtcatgaagcagataagcgtcagagctaggaatagagcccagctgttctgcccagccgagactccttgcatgctagagacctccttgcccctccagagactgtgggaagggctactgaattagctcagagatatttccaggctctgcaacctatttcttaacctg--tttttctgaaaataaaccttgagtgtcccttc--------------------------------------

Otus_angelinae_29 -------------------------------------------------------------------------------------------------------------------------------------------------------------------------------------------------------------------------------------------------------------------------------------------------------------------------------------------------------------------------------------------------------------------------------------------------------------------------------------------------------------------------------------------------------------------------------------------------------------------------------------------------------------------------------------------------------------------------------------------------

Otus_spilocephalus_vandewateri_97 -------------------------------------------------------------------------------------------------------------------------------------------------------------------------------------------------------------------------------------------------------------------------------------------------------------------------------------------------------------------------------------------------------------------------------------------------------------------------------------------------------------------------------------------------------------------------------------------------------------------------------------------------------------------------------------------------------------------------------------------------

Otus_spilocephalus_vulpes_96 -------------------------------------------------------------------------------------------------------------------------------------------------------------------------------------------------------------------------------------------------------------------------------------------------------------------------------------------------------------------------------------------------------------------------------------------------------------------------------------------------------------------------------------------------------------------------------------------------------------------------------------------------------------------------------------------------------------------------------------------------

Otus_spilocephalus_spilocephalus_90 caaatatctggaggtatggaaaagggcagggaatcctgctgcctgatgtgtagtgaatgtgtgcaagacagctatgcgagagctatacttttatttactgaatgctagttggacttcagtg-agctctccctcaagtctaaggtctctctgtacaggcaggaggagacacagaaagggctcatggtatgcgaatggtatgtgaatatccaagtttagatttcccattcctaacaccacatgcagtctgagcaacccttgacaataataaaccagcccaggcagcctctgcatgcctgggaaactacattacataacaactgtcagtggctggacacaagggacatacaattttagagtaagccctggaggatccattggagaccaagacccacaaaactaagtgttgtacaaacrcaaggcatgagttttcgaccttaatggatgaggcagacaaagagtgggaagggccatggtctactcaaggtcatgaagcagataagcgtcagagctaggaatagagcccagttgttctgcccagccgagactccttgcatgctagagacctccttgcccctccagagactgtgggaagggctactgaattagctcagagatatttccaggctctgcaacctatttcttaacctg--tttttctgaaaataagccttgagtgtcccttcagtttttctctctctccttccttcctcacagttcattt

Otus_spilocephalus_hambroecki_92 -------------------------------------------------------------------------------------------------------------------------------------------------------------------------------------------------------------------------------------------------------------------------------------------------------------------------------------------------------------------------------------------------------------------------------------------------------------------------------------------------------------------------------------------------------------------------------------------------------------------------------------------------------------------------------------------------------------------------------------------------

_Otus_spilocephalus_latouchi_CHIMERA -------------------------------------------------------------------------------------------------------------------------------------------------------------------------------------------------------------------------------------------------------------------------------------------------------------------------------------------------------------------------------------------------------------------------------------------------------------------------------------------------------------------------------------------------------------------------------------------------------------------------------------------------------------------------------------------------------------------------------------------------

Otus_hartlaubi_13 caaatatctggaggtatggaaaagggcagggaatcctgctgcctgatgtgtagtgaatgtgtgcaagacagctatgcgagagctgtgcttttatttactgatggctagttggacttcagtg-aactctccctcaagtctaaggtctctctgtacaggcaggaggagacacagaaagggctcatggtatgcgaatggtatgtgaatatccaagtttagatttcccattcctaacaccacacgcagtctgagcaacccttgacaataataaaccagcccaggcagcctctgcatgcctgggaaactacattacataacaactgtcagtggctggacacaagggacatacaattttagagtaagccctggaggatccattggagaccaagacccacaaaactaagtgttgtacaaacacagggcatgagttttcgaccttaatggatgaggcagacaaagagtgggaagggccatggtctactcaaggtcatgaagcagataagcgtcagagctaggaatagagcccagttgttctgcccagcccagactccttgcatgctagagacctccctgcccctccagagactgtgggaagggctagtgaattagctcagagatatttccaggctctgcaacctatttcttaacctg-ttttttctgaaaataaaccttgagtgtcccttcagtttttctctctctccttccttcctcacagttcattt

Otus_hartlaubi_15 ---------------------------------tcctgctgcctgatgtgtagtgaatgtgtgcaagacagctatgcgagagctgtgcttttatttactgatggctagttggacttcagtg-aactctccctcaagtctaaggtctctctgtacaggcaggaggagacacagaaagggctcatggtatgcgaatggtatgtgaatatccaagtttagatttcccattcctaacaccacacgcagtctgagcaacccttgacaataataaaccagcccaggcagcctctgcatgcctgggaaactacattacataacaactgtcagtggctggacacaagggacatacaattttagagtaagccctggaggatccattggagaccaagacccacaaaactaagtgttgtacaaacacagggcatgagttttcgaccttaatggatgaggcagacaaagagtgggaagggccatggtctactcaaggtcatgaagcagataagcgtcagagctaggaatagagcccagttgttctgcccagcccagactccttgcatgctagagacctccctgcccctccagagactgtgggaagggctagtgaattagctcagagatatttccaggctctgcaacctatttcttaacctg-ttttttctgaaaataaaccttgagtgtcccttcagtttttctctctctccttccttcctcacagttcattt

Otus_hartlaubi_16 ---------------------------------tcctgctgcctgatgtgtagtgaatgtgtgcaagacagctatgcgagagctgtgcttttatttactgatggctagttggacttcagtg-aactctccctcaagtctaaggtctctctgtacaggcaggaggagacacagaaagggctcatggtatgcgaatggtatgtgaatatccaagtttagatttcccattcctaacaccacacgcagtctgagcaacccttgacaataataaaccagcccaggcagcctctgcatgcctgggaaactacattacataacaactgtcagtggctggacacaagggacatacaattttagagtaagccctggaggatccattggagaccaagacccacaaaactaagtgttgtacaaacacagggcatgagttttcgaccttaatggatgaggcagacaaagagtgggaagggccatggtctactcaaggtcatgaagcagataagcgtcagagctaggaatagagcccagttgttctgcccagcccagactccttgcatgctagagacctccctgcccctccagagactgtgggaagggctagtgaattagctcagagatatttccaggctctgcaacctatttcttaacctg-ttttttctgaaaataaaccttgagtgtcccttcagtttttctctctctccttccttcctcacagttcattt

Otus_hartlaubi_14 ---------------------------------tcctgctgcctgatgtgtagtgaatgtgtgcaagacagctatgcgagagctgtgcttttatttactgatggctagttggacttcagtg-aactctccctcaagtctaaggtctctctgtacaggcaggaggagacacagaaagggctcatggtatgcgaatggtatgtgaatatccaagtttagatttcccattcctaacaccacacgcagtctgagcaacccttgacaataataaaccagcccaggcagcctctgcatgcctgggaaactacattacataacaactgtcagtggctggacacaagggacatacaattttagagtaagccctggaggatccattggagaccaagacccacaaaactaagtgttgtacaaacacagggcatgagttttcgaccttaatggatgaggcagacaaagagtgggaagggccatggtctactcaaggtcatgaagcagataagcgtcagagctaggaatagagcccagttgttctgcccagcccagactccttgcatgctagagacctccctgcccctccagagactgtgggaagggctagtgaattagctcagagatatttccaggctctgcaacctatttcttaacctg-ttttttctgaaaataaaccttgagtgtcccttcagtttttctctctctccttccttcctcacagttcattt

Otus_feae_45 -------------gtatggaaaagggcagggaatcctgctgcctgatgtgtagtgaatgtgtgcaagacagctatgcgagagctgtgcttttatttactgatggctagttggacttcagtg-agctctccctcaagtctaaggtctctctgtacaggcaggaggagacacagaaagggctcatggtatgcgaatggtatgtgaatatccaagtttagatttcccattcctaacaccacacgcagtctgagcaacccttgacaataataaaccagcccaggcagcctctgcatgcctgggaaactacattacataacaactgtcagtggctggacacaagggacatacaattttagagtaagccctggaggatccattggagaccaagacccacaaaactaagtgttgtacaaacacagggcatgagttttcgaccttaatggatgaggcagacaaagagtgggaagggccatggtctactcaaggtcatgaagcagataagcgtcagagctaggaatagagcccagttgttctgcccagcccagactccttgcatgctagagacctccctgcccctccagagactgtgggaagggctagtgaattagctcagagatatttccaggctctgcaacctatttcttaacctg-ttttttctgaaaataaaccttgagtgtcccttcagtttttctctctctccttccttcctcacag-------

Otus_senegalensis_17 caaatatctggaggtatggaaaagggcagggaatcctgctgcctgatgtgtagtgaatgtgtgcaagacagctatgcgagagctgtgcttttatttactgatggctagttggacttcagtg-agctctccctcaagtctaaggtctctctgtacaggcaggaggagacacagaaarggctcatggtatgcgaatggtatgtgaatatccaagtttagatttcccattcctaacaccacacgcagtctgagcaacccttgacaataataaaccagcccaggcagcctctgcatgcctgggaaactacattacataacaactgtcagtggctggacacaagggacatacaattttagagtaagccctggaggatccattggagaccaagacccacaaaactaagtgttgtacaaacacagggcatgagttttcgaccttaatggatgaggcagacaaagagtgggaagggccatggtctactcaaggtcaygaagcagataagcgtcagagctaggaatagagcccagttgttctgcccagcccagactccttgcatgctagagacctccctgcccctccagagactgtgggaagggctagtgaattagctcagagatatttccaggctctgcaacctatttcttaacctg-ttttttctgaaaataaaccytgagtgtcccttcagtttttctctctctccttccttcctcacagttcattt

Otus_pamelae_74 -aaatatctggaggtatggaaaagggcagggaatcctgctgcctgatgtgtagtgaatgtgtgcaagacagctatgcgagagctgtgcttttatttactgatggctagttggacttcagtg-agctctccctcaagtctaaggtctctctgtacaggcaggaggagacacagaaagggctcatggtatgcgaatggtatgtgaatatccaagtttagatttcccattcctaacaccacacgcagtctgagcaacccttgacaataataaaccagcccaggcagcctctgcatgcctgggaaa-----------------------------------------------------------------------------------------------------------------------------------------------------------------------------------------------------------------------------------------------------------------------------------------------------------------------------------------------------------------------------------------------------------------------------------

Otus_sp_nov_1 ---------------------------------tcctgctgcctgatgtgtagtgaatgtgtgcaagacagctatgcgagagctgtgcttttattwactgatggctagttggacttcagtg-agctctccctcaagtctaaggtctctctgtacaggcaggaggagacacagaaagggctcatggtatgcgaatggtatgtgaatatccaagtttagatttcccattcctaacaccacacgcagtctgagcaa-ccttgacaataataaaccagcccaggcagccactgcatgcctgggaaactacattacataacaactgtcagtggctggacacaagggacatacaattttagagtaagccctggaggatccattggagaccaagacccacaaaactaagtgttgtacaaacacagggcatgagttttcgaccttaatggatgaggcagacaaagagtgggaagggccatggtctactcaaggtcatgaagcagataagcgtcagagctaggaatagagcccagttgttctgcccagcccagactccttgcatgctagagacctccctgcccctccagagactgtgggaagggctagtgaattagctcagagatatttccaggctctgcaacctatttcttaacctg-ttttttctgaaaataaaccttgagtgtcccttcagtttttctctctctccttccttcctcacagttcattt

Otus_sp_nov_2 -------------------------------------------------------------------------------------------------------------------------------------------------------------------------------------------------------------------------------------------------------------------------------------------------------------------------------------------------------------------------------------------------------------------------------------------------------------------------------------------------------------------------------------------------------------------------------------------------------------------------------------------------------------------------------------------------------------------------------------------------

Otus_sp_nov_3 ---------------------------------tcctgctgcctgatgtgtagtgaatgtgtgcaagacagctatgcgagagctgtgcttttatttactgatggctagttggacttcagtg-agctctccctcaagtctaaggtctctctgtacaggcaggaggagacacagaaggggctcatggtatgcgaatggtatgtgaatatcccagtttagatttcccattcctaacaccacacgcagtcwgagcaa-ccttgaccataataaaccagcccaggcagccactgcatgcctgggaaactacattacataacaactgtcagtggctggacacaagggacatacaattttagagtaagccctggaggatccattggagaccaagacccacaaaactaagtgttgtacaaacacagggcatgagttttcgaccttaatggatgaggcagacaaagagtgggaagggccatggtctactcaaggtcatgaagcaaataagcgtcagagctaggaatagagcccagttgttctgcccagcccagactccttgcatgctagagacctccctgcccctccagagac-----------------------------------------------------------------------------------------------------------------------------------------

Otus_sp_nov_4 ---------------------------------tcctgctgcctgatgtgtagtgaatgtgtgcaagacagctatgcgagagctgtgcttttatttactgatggctagttggacttcagtg-agctctccctcaagtctaaggtctctctgtacaggcaggaggagacacagaaggggctcatggtatgcgaatggtatgtgaatatcccagtttagatttcccattcctaacaccacacgcagtcwgagcaa-ccttgaccataataaaccagcccaggcagccactgcatgcctgggaaactacattacataacaactgtcagtggctggacacaagggacatacaattttagagtaagccctggaggatccattggagaccaagacccacaaaactaagtgttgtacaaacacagggcatgagttttcgaccttaatggatgaggcagacaaagagtgggaagggccatggtctactcaaggtcatgaagcaaataagcgtcagagctaggaatagagcccagttgttctgcccagcccagactccttgcatgctagagacctccctgcccctccagagac-----------------------------------------------------------------------------------------------------------------------------------------

Otus_scops_5 caaatatctggaggtatggaaaagggcagggaatcctgctgcctgatgtgtagtgaatgtgtgcaagacagctatgcgagagctgtgcttttatttactgatggctagttggacttcagtg-agctctccctcaagtctaaggtctcyctgtacaggcaggaggagacacagaaagggctcatggtatgcgaatggtatgtgaatatccaagtttagatttcccattcctaacaccacacgcagtctgagcaacccttgacaataataaaccagcccaggcagcctctgcatgcctgggaaactacattacataacaactgtcagtggctggacacaagggacatacaattttagagtaagccctggaggatccattggagaccaagacccacaaaactaagtgttgtacaaacacagggcatgagttttcgaccttaatggatgaggcagacaaagagtgggaagggccatggtctactcaaggtcatgaagcagataagcgtcagagctaggaatagagcccagttgttctgcccagcccagactccttgcatgctagagacctccctgcccctccagagactgtgggaagggctagtgaattagctcagagatatttccaggctctgcaacctatttcttaacctg-ttttttctgaaaataaaccttgagtgtcccttcagtttttctctctctccttccttcctcacagttcattt

_Otus_scops_JF5337_F -------------------------------------------------------------------------------------------------------------------------------------------------------------------------------------------------------------------------------------------------------------------------------------------------------------------------------------------------------------------------------------------------------------------------------------------------------------------------------------------------------------------------------------------------------------------------------------------------------------------------------------------------------------------------------------------------------------------------------------------------

Otus_pembaensis_24 caaatatctggaggtatggaaaagggcagggaatcctgctgcctgatgtgtagtgaatgtgtgcaagacagctatgcgagagctgtgcttttatttactgatggctagttggacttcagtg-agctctccctcaagtctaaggtctctctgtacaggcaggaggagacacagaaagggctcatggtatgcgaatggtatgtgaatatccaagtttagatttcccattcctaacaccacacgcagtctgagcaacccttgacaataataaaccagcccaggcagcctctgcatgcctgggaaactacattacataacaactgtcagtggctggacacaagggacatacaattttagagtaagccctggaggatccattggagaccaagacccacaaaactaagtgttgtacaaacacagggcatgagttttcgaccttaatggatgaggcagacaaagagtgggaagggccatggtctactcaaggtcatgaagcagataagcgtcagagctaggaatagagcccagttgttctgcccagcccagactccttgcatgctagagacctccctgcccctccagagactgtgggaagggctagtgaattagctcagagatatttccaggttctgcaacctatttcttaacctg-ttttttctgaaaataaaccttgagtgtcccttcagtttttctctctctccttccttcctcacagttcattt

Otus_pembaensis_25 caaatatctggaggtatggaaaagggcagggaatcctgctgcctgatgtgtagtgaatgtgtgcaagacagctatgcgagagctgtgcttttatttactgatggctagttggacttcagtg-agctctccctcaagtctaaggtctctctgtacaggcaggaggagacacagaaagggctcatggtatgcgaatggtatgtgaatatccaagtttagatttcccattcctaacaccacacgcagtctgagcaacccttgacaataataaaccagcccaggcagcctctgcatgcctgggaaactacattacataacaactgtcagtggctggacacaagggacatacaattttagagtaagccctggaggatccattggagaccaagacccacaaaactaagtgttgtacaaacacagggcatgagttttcgaccttaatggatgaggcagacaaagagtgggaagggccatggtctactcaaggtcatgaagcagataagcgtcagagctaggaatagagcccagttgttctgcccagcccagactccttgcatgctagagacctccctgcccctccagagactgtgggaagggctagtgaattagctcagagatatttccaggttctgcaacctatttcttaacctg-ttttttctgaaaataaaccttgagtgtcccttcagtttttctctctctccttccttcctcacagttcattt

_Otus_longicornis_CHIMERA caaatatctggaggtatgaaaaaggacagggaatcctgctgcctgatgtgtagtgaatgtgtgcaagacagctatgcgagagctgtgcttttatttactgttggctagttggacttcagtg-agctctccctcaagtctaaggtctctctgtacaggcaggaggagacacagaaagggctcatggtatgcgaatggtatgtgaatatccaagtttagatttcccattcctaacaccacatgcagtctgagcaacccttgacaataataaaccagcccaggcagcctctgcatgcctgggaaactacattacataacaactgtcagtggctggacacgagggacatacaattttagagtaagccctggaggatccattggagaccaagacccacaaaactaagtgttgtacaaacacagggcatgagttttcgaccttaatggatgaggcagacaaagagtgggaagggccatggtctactcaaggtcatgaagcagataagcgtcagagctaggaatagagcccagttgttctgcccagcccagactccttgcatgctagagacctccctgcccctccagagactgtgggaagggctagtgaattagctcagagatatttccaggctctgcaacctatttcttaacctg-ttttttctgaaaataaaccttgagtgtcccttcagtttttctctctctccttccttcctcacagttcattt

Otus_mirus_CHIMERA caaatatctggaggtatgaaaaaggacagggaatcctgctgcctgatgtgtagtgaatgtgtgcaagacagctatgcgagagctgtgcttttatttactgttggctagttggacttcagtg-agctctccctcaagtctaaggtctctctgtacaggcaggaggagacacagaaagggctcatggtatgcgaatggtatgtgaatatccaagtttagatttcccattcctaacaccacatgcagtctgagcaacccttgacaataataaaccagcccaggcagcctctgcatgcctgggaaactacattacataacaactgtcagtggctggacacgagggacatacaattttagagtaagccctggaggatccattggagaccaagacccacaaaactaagtgttgtacaaacacagggcatgagttttcgaccttaatggatgaggcagacaaagagtgggaagggccatggtctactcaaggtcatgaagcagataagcgtcagagctaggaatagagcccagttgttctgcccagcccagactccttgcatgctagagacctccctgcccctccagagactgtgggaagggctagtgaattagctcagagatatttccaggctctgcaacctatttcttaacctg-ttttttctgaaaataaaccttgagtgtcccttcagtttttctctctctccttccttcctcacagttcattt

Otus_mayottensis_60 caaatatctggaggtatgaaaaaggacagggaatcctgctgcctgatgtgtagtgaatgtgtgcaagacagctatgcgagagctgtgcttttatttactgttggctagttggacttcagtg-agctctccctcaagtctaaggtctctctgtacaggcaggaggagacacagaaagggctcatggtatgcgaatggtatgtgaatatccaagtttagatttcccattcctaacaccacatgcagtctgagcaacccttgacaataataaaccagcccaggcagcctctgcatgcctgggaaactacattacataacaactgtcagtggctggacacgagggacatacaattttagagtaagccctggaggatccattggagaccaagacccacaaaactaagtgttgtacaaacaccgggcatgagttttcgaccttaatggatgaggcagacaaagagtgggaagggccatggtctactcaaggtcatgaagcagataagcgtcagagctaggaatagagcccagttgttctgcccagcccagactccttgcatgctagagacctccctgcccctccagagactgtgggaagggctagtgaattagctcagagatatttccaggctctgcaacctatttcttaacctg-ttttttctgaaaataaaccttgagtgtcccttcagtttttctctctctccttccttcctcacagttcattt

Otus_capnodes_40 caaatatctggaggtatgaaaaaggacagggaatcctgctgcctgatgtgtagtgaatgtgtgcaagacagctatgcgagagctgtgcttttatttactgttggctagttggacttcagtg-agctctccctcaagtctaaggtctctctgtacaggcaggaggagacacagaaagggctcatggtatgcaaatggtatgtgaatatccaagtttagatttcccattcctaacaccacatgcagtctgagcaacccttgacaataataaaccagcccaggcagcctctgcatgcctgggaaactacattacataacaactgtcagtggctggacacgagggacatacaattttagagtaagccctggaggatccattggagaccaagacccacaaaactaagtgttgtacaaacacagggcatgagttttcgaccttaatggatgaggcagacaaagagtgggaagggccatggtctactcaaggtcatgaagcagataagcgtcagagctaggaatagagcccagttgttctgcccagcccagactccttgcatgctagagacctccctgcccctccagagactgtgggaagggctagtgaattagctcagagatatttccaggccctgcaacctatttcttaacctg-ttttttctgaaaataaaccttgagtgtcccttcagtttttctctctctccttccttcctcacagttcattt

_Otus_madagascariensis_CHIMERA caaatatctggaggtatgaaaaaggacagggaatcctgctgcctgatgtgtagtgaatgtgtgcaagacagctatgcgagagctgtgcttttatttactgttggctagttggacttcagtg-agctctccctcaagtctaaggtctctctgtacaggcaggaggagacacagaaagggctcatggtatgcgaatggtatgtgaatatccaagtttagatttcccattcctaacaccacatgcagtctgagcaacccttgacaataataaaccagcccaggcagcctctgcatgcctgggaaactacattacataacaactgtcagtggctggacacgagggacatacaattttagagtaagccctggaggatccattggagaccaagacccacaaaactaagtgttgtacaaacacagggcatgagttttcgaccttaatggatgaggcagacaaagagtgggaagggccatggtytactcaaggtcatgaagcagataagcrtcagagctaggaatagagcccagttgttctgcccagcccagactccttgcatgctagagacctccctgcccctccagagactgtgggaagggctagtgaattagctcagagatatttccaggctctgcaacctatttcttaacctg-ttttttctgaaaataaaccttgagtgtcccttcagtttttckctctctccttccttcctcacagttcattt

Otus_pauliani_76 caaatatctggaggtatgaaaaaggacagggaatcctgctgcctgatgtgtagtgaatgtgtgcaagacagctatgcgagagctgtgcttttatttactgttggctagttggacttcagtg-agctctccctcaagtctaaggtctctctgtacaggcaggaggagacacagaaagggctcatggtatgcgaatggtatgtgaatatccaagtttagatttcccattcctaacaccacatgcagtctgagcaacccttgacaataataaaccagcccaggcagcctctgcatgcctgggaaactacattacataacaactgtcagtggctggacacgagggacatacaattttagagtaagccctggaggatccattggagaccaagacccacaaaactaagtgttgtacaaacacagggcatgagttttcgaccttaatggatgaggcagacaaagagtgggaagggccatggtctactcaaggtcatgaagcagataagcgtcagagctaggaatagagcccagttgttctgcccagcccaractccttgcatgctagagacctccctgcccctccagagactgtgggaagggctagtgaattagctcagagatatttccaggctctgcaacctatttcttaacctg-ttttttctgaaaataaaccttgagtgtcccttcagtttttctctctctccttccttcctcacagttcattt

Otus_insularis_47 -------ctggaggtatgaaaaaggacagggaatcctgctgcctgatgtgtagtgaatgtgtgcaagacagctatgcgagagctgtgcttttatttactgttggctagttggacttcagtg-agctctccctcaagtctaaggtctctctgtacaggcaggaggagacacagaaagggctcatggtatgcgaatggtatgtgaatatccaagtttagatttcccattcctaacaccacatgcagtctgagcaacccttgacaataataaaccagcccaggcagcctctgcatgcctgggaaactacattacataacaactgtcagtggctggacacgagggacatacaattttagagtaagccctggaggatccattggagaccaagacccacaaaactaagtgttgtacaaacacagggcatgagttttcgaccttaatggatgaggcagacaaagagtgggaagggccatggtctactcaaggtcatgaagcagataagcgtcagagctaggaatagagcccagttgttctgcccagcccagactccttgcatgctagagacctccctgcccctccagagactgtgggaagggctagtgaattagctcagagatatttccaggctctgcaacctatttcttaacctg-ttttttctgaaaataaaccttgagtgtcccttcagtttttctctctctccttccttcctcacagttcattt

Otus_socotranus_89 -aaatatctggaggtatgaaaaaggacagggaatcctgctgcctgatgtgtagtgaatgtgtgcaagacagcyatgcgagagctgtgcttttatttactgttggctagttggacttcagtg-agctctccctcaagtctaaggtctctctgtacaggcaggaggagacacagaaagggctcatggtatgcgaatggtatgtgaatatccaagtttagatttcccattcctaacaccacatgcagtctgagcaacccttgacaataataaaccagcccaggcagcctctgcatgcctgggaaactacattacataacaactgtcagtggctggacacgagggacatacaattttagagtaagccctggaggatccattggagaccaagacccacaaaactaagtgttgtacaaacacagggcatgagttttcgaccttaatggatgaggcagacaaagagtgggaagggccatggtctactcaaggtcatgaagcagataagcgtcagagctaggaatagagcccagttgttctgcccagcccagactccttgcatgctagagacctccctgcccctccagagactgtgggaagggctagtgaattagctcagagatatttccaggctctgcaacctatttcttaacctg-ttttttctgaaaataaaccttgagtgtcccttcagtttttctctctctccttccttcctcacagttcattt

_Otus_sunia_CHIMERA -------ctggaggtatgaaaaaggacagggaatcctgctgcctgatgtgtagtgaatgtgtgcaagacagctatgtgagagctgtgcttttatttactgttggctagttggacttcagtg-agctctccctcaagtctaaggtctctctgtacaggcaggaggagacacagaaagggctcatggtatgcgaatggtatgtgaatatccaagtttagatttcccattcctaacaccacatgcagtctgagcaacccttgacaataataaaccagcccaggcagcctctgcatgcctgggaaactgcattacataacaactgtcagtggctggacacgagggacatacaattttagagtaagccctggaggatccattggagaccaagacccacaaaactaagtgttgtacaaacacagggcatgagttttcgaccttaatggatgaggcagacaaagagtgggaagggccatggtctactcaaggtcatgaagcagataagcgtcagagctaggaatagagcccagttgttctgcccagcccagactccttgcatgctagagacctccctgcccctccagagactgtgggaagggctagtgaattagctcagagatatttccaggctctgcaacctatttcttaacctg-ttttttctgaaaataaaccttgagtgtcccttcagtttttctctctctccttccttcctcacagttcattt

Otus_moheliensis_CHIMERA ----------------------------------------------------------------------------------------------------------------------------------------------------------------gaggagacacagaaagggctcatggtatgcgaatggtatgtgaatatccaagtttagatttcccattccwaacaccacatgcagtctgagcaacgcttgacaataataaaccagcccaggcagcctctgcatgcctgggaaactacattacataacaactgtcagtggctggacacgagggacatacaattttagagtaagccctggaggatccattggagaccaagacccacaaaactaagtgttgtacaaacacagggcatgagttttcgaccttaatggatgaggcagacaaagagtgggaagggccatggtctactcaaggtcatgaagcagataagcgtcagagctaggaatagagcccagttgttctgcccagcccagactccttgcatgctagagacctccctgcccctccagagactgtgggaagggctagtgaattagctcagagatatttccaggctctgcaacctatttcttaacctg-ttttttctgaaaataaaccttgagtgtcccttcagtttttctctctctccttccttcctcacagttcattt

Otus_brucei_CHIMERA -aaatatctggaggtatggaaaagggcagggaatcctgctgcctgatgtgtagtgaatgtgtgcaagacagctatgcgagagctgtgcttttatttactgatggctagttggacttcagtg-agctctccctcaagtctaaggcctctctgtacaggcaggaggagacacagaaagggctcatggtatgcgaatggtatgtgaatatccaagtttagatttcccattcctaacaccacatgcagtctgagtaacccttgacaataataaaccagcccaggcagcctctgcatgcctgggaaa-----------------------------------------------------------------------------------------------------------------------------------------------------------------------------------------------------------------------------------------------------------------------------------------------------------------------------------------------------------------------------------------------------------------------------------

Otus_icterorhynchus_holerythrus_27 -------------------------------------------------------------------------------------------------------------------------------------------------------------------------------------------------------------------------------------------------------------------------------------------------------------------------------------------------------------------------------------------------------------------------------------------------------------------------------------------------------------------------------------------------------------------------------------------------------------------------------------------------------------------------------------------------------------------------------------------------

Otus_icterorhynchus_CHIMERA ---------------------------------tcctgctgcctgatgtgtagtgaatgtgtgcaagacagctatgcgagagctgtgcttttatttactgatggctagttggacttcagtg-agctctccctcaagtctaaggtctctctgtacaggcaggaggagacacagaaagggctcatggtatgcgaatggtatgtgaatatccaagtttaga-----------------------------------------------------------------------------------------------------------------------------------------------------------------------------------------------------------------------------------------------------------------------------------------------------------------------------------------------------------------------------------------------------------------------------------------------------------------------------------------------------------------------

Otus_ireneae_49 ---------------atggaaaaggacagggaatcctgctgcctgatgtgtagtgaatgtgtgcaagacagctatgcgagagctgtgcttttatttactgatggctagttggacttcagtg-agctctccctcaagtctaaggcctctctgtacaggcaggaggagacacagaaagggctcatggtatgcgaatggtatgtgaatatccaagtttagatttcccattcctgacaccacatgcagtctgagcaacccttgacaataataaaccagcccaggcagcctctgcatgcctgggaaactacattacataacaactgtcagtggctggacacgagggacatacagttttagagtaagccctggaggatccattggagaccaagacccacaaaactaagtgttgtacaaacacagggcatgagttttcgaccttaatggatgaggcagacaaagagtgggaagggccatggtctactcaaggtcatgaagcagataagcgtcagagctaggaatagagcccagttgttctgcccagcccagactccttgcatgctagagacctccctgcccctccagagactgtgggaagggctactgaattagctcagagatatttccaggctctgcaacctatttcttaacctg-ttttttctgaaaataaaccttgagtgtcctttcagtttttctctctctccttccttcctcacagttcattt

Otus_rutilus_78 caaatatctggaggtaygaaaaaggacagggaatcctgctgcctgatgtgtagtgaatgtgtgcaagacagctatgcgagagctgtgyttttatttactgttggctagttggacttcagtg-agctctccctcaagtctaaggtctctctgtacaggcaggaggagacacagaaagggctcatggtatgcgaatggtatgtgaatatccaagtttagatttcccattcctaacaccacatgcagtctgagcaacccttgacaataataaaccagcccaggcagcctctgcatgcctgggaaactacattacataacaactgtcagtggctggacacgagggacatacaattttagagtaagccctggaggatccattggagaccaagacccacaaaactaagtgttgtacaaacacagggcatgagttttcgaccttaatggatgaggcagacaaagagtgggaagggccatggtctactcaaggtcatgaagcagataagcgtcagagctaggaatagagcccagttgttctgcccagcccagactccttgcatgctagagacctccctgccsctccagagactgtgggaagggctagtgaattagctcagagatatttccaggctctgcaacctatttcttaacctg-ttttttctgaaaataaaccttgagtgtcccttcagtttttctctctctccttccttcctcacagttcattt

Otus_scops_scops_8 -------------------------------------------------------------------------------------------------------------tggacttcagtg-agctctccctcaagtctaaggtctctctgtacaggcaggaggagacacagaaagggctcatggtatgcgaatggtatgtgaatatccaagtttagatttcccattccwaacaccacacgcagtctgagcaacccttgacaataataaaccagcccaggcagcctctgcatgcctgggaaactacattacataacaactgtcagtggctggacacaagggacatacaattttagagtaagccctggaggatccattggagaccaagacccacaaaactaagtgttgtacaaacacagggcatgagttttcgaccttaatggatgaggcagacaaagagtgggaagggccatggtctactcaaggtcatgaagcagataagcgtcagagctaggaatagagcccagttgttctgcccagcccagactccttgcatgctagagacctccctgcccctccagagactttgggaagggctagtgaattagctcagagatatttccaggctctgcaacctatttcttaacctg-ttttttctgaaaataaaccttgagtgtcccttcagtttttctctctctccttccttcctcacagttcattt

Otus_scops_scops_9 ---------------------------------tcctgctgcctgatgtgtagtgaatgtgtgcaagacagctatgcgagagctgtgcttttattwactgatggctagttggacttcagtgttggactccctcaagtctaaggtctctctgtacaggcaggaggagacacagaaagggctcatggtatgcgaatggtatgtgaatatccaagtttagatttcccattcmtaacaccacacgcagtctgagcaacccttgacaataataaaccagcccaggcagcctctgcatgcctgggaaactacattacataacaactgtcagtggctggacacaagggacatacaattttagagtaagccctggaggatccattggagaccaagacccacaaaactaagtgttgtacaaacacagggcatgagttttcgaccttaatggatgaggcagacaaagagtgggaagggccatggtctactcaaggtcatgaagcagataagcgtcagagctaggaatagagcccagttgttctgcccagcccagactccttgcatgctagagacctccctgcccctccagagactttgggaagggctagtgaattagctcagagatatttccaggctctgcaacctatttcttaacctg-ttttttctgaaaataaaccttgagtgtcccttcagtttttctctctctccttccttcctcacagttcattt

Otus_spilocephalus_luciae_94 -------------------------------------------------------------------------------------------------------------------------------------------------------------------------------------------------------------------------------------------------------------------------------------------------------------------------------------------------------------------------------------------------------------------------------------------------------------------------------------------------------------------------------------------------------------------------------------------------------------------------------------------------------------------------------------------------------------------------------------------------

Otus_silvicola_87 -------------------------------------------------------------------------------------------------------------------------------------------------------------------------------------------------------------------------------------------------------------------------------------------------------------------------------------------------------------------------------------------------------------------------------------------------------------------------------------------------------------------------------------------------------------------------------------------------------------------------------------------------------------------------------------------------------------------------------------------------

_Otus_cyprius_CHIMERA -------------------------------------------------------------------------------------------------------------------------------------------------------------------------------------------------------------------------------------------------------------------------------------------------------------------------------------------------------------------------------------------------------------------------------------------------------------------------------------------------------------------------------------------------------------------------------------------------------------------------------------------------------------------------------------------------------------------------------------------------

Otus_elegans_CHIMERA -------------------------------------------------------------------------------------------------------------------------------------------------------------------------------------------------------------------------------------------------------------------------------------------------------------------------------------------------------------------------------------------------------------------------------------------------------------------------------------------------------------------------------------------------------------------------------------------------------------------------------------------------------------------------------------------------------------------------------------------------

[marker: KIAA; length: 870; no. of seqs: 51; new coordinates: 4564-5433)]

Bubo_bubo_111 ------------------------------------------------------------------------------------------------------------------------------------------------------------------------------------------------------------------------------------------------------------------------------------------------------------------------------------------------------------------------------------------------------------------------------------------------------------------------------------------------------------------------------------------------------------------------------------------------------------------------------------------------------------------------------------------------------------------------------------------------------------------------------------------------------------------------------------------------------------------------------------------------------

Strix_aluco_129 ------------------------------------------------------------------------------------------------------------------------------------------------------------------------------------------------------------------------------------------------------------------------------------------------------------------------------------------------------------------------------------------------------------------------------------------------------------------------------------------------------------------------------------------------------------------------------------------------------------------------------------------------------------------------------------------------------------------------------------------------------------------------------------------------------------------------------------------------------------------------------------------------------

Otus_bakkamoena_marathae_35 ------------------------------------------------------------------------------------------------------------------------------------------------------------------------------------------------------------------------------------------------------------------------------------------------------------------------------------------------------------------------------------------------------------------------------------------------------------------------------------------------------------------------------------------------------------------------------------------------------------------------------------------------------------------------------------------------------------------------------------------------------------------------------------------------------------------------------------------------------------------------------------------------------

_Otus_lempiji_CHIMERA ------------------------------------------------------------------------------------------------------------------------------------------------------------------------------------------------------------------------------------------------------------------------------------------------------------------------------------------------------------------------------------------------------------------------------------------------------------------------------------------------------------------------------------------------------------------------------------------------------------------------------------------------------------------------------------------------------------------------------------------------------------------------------------------------------------------------------------------------------------------------------------------------------

Otus_lettia_lettia_CHIMERA ------------------------------------------------------------------------------------------------------------------------------------------------------------------------------------------------------------------------------------------------------------------------------------------------------------------------------------------------------------------------------------------------------------------------------------------------------------------------------------------------------------------------------------------------------------------------------------------------------------------------------------------------------------------------------------------------------------------------------------------------------------------------------------------------------------------------------------------------------------------------------------------------------

Otus_megalotis_CHIMERA ------------------------------------------------------------------------------------------------------------------------------------------------------------------------------------------------------------------------------------------------------------------------------------------------------------------------------------------------------------------------------------------------------------------------------------------------------------------------------------------------------------------------------------------------------------------------------------------------------------------------------------------------------------------------------------------------------------------------------------------------------------------------------------------------------------------------------------------------------------------------------------------------------

Otus_nigrorum_CHIMERA ------------------------------------------------------------------------------------------------------------------------------------------------------------------------------------------------------------------------------------------------------------------------------------------------------------------------------------------------------------------------------------------------------------------------------------------------------------------------------------------------------------------------------------------------------------------------------------------------------------------------------------------------------------------------------------------------------------------------------------------------------------------------------------------------------------------------------------------------------------------------------------------------------

Otus_everetti_CHIMERA ------------------------------------------------------------------------------------------------------------------------------------------------------------------------------------------------------------------------------------------------------------------------------------------------------------------------------------------------------------------------------------------------------------------------------------------------------------------------------------------------------------------------------------------------------------------------------------------------------------------------------------------------------------------------------------------------------------------------------------------------------------------------------------------------------------------------------------------------------------------------------------------------------

Otus_semitorques_CHIMERA ------------------------------------------------------------------------------------------------------------------------------------------------------------------------------------------------------------------------------------------------------------------------------------------------------------------------------------------------------------------------------------------------------------------------------------------------------------------------------------------------------------------------------------------------------------------------------------------------------------------------------------------------------------------------------------------------------------------------------------------------------------------------------------------------------------------------------------------------------------------------------------------------------

Otus_angelinae_29 ------------------------------------------------------------------------------------------------------------------------------------------------------------------------------------------------------------------------------------------------------------------------------------------------------------------------------------------------------------------------------------------------------------------------------------------------------------------------------------------------------------------------------------------------------------------------------------------------------------------------------------------------------------------------------------------------------------------------------------------------------------------------------------------------------------------------------------------------------------------------------------------------------

Otus_spilocephalus_vandewateri_97 ------------------------------------------------------------------------------------------------------------------------------------------------------------------------------------------------------------------------------------------------------------------------------------------------------------------------------------------------------------------------------------------------------------------------------------------------------------------------------------------------------------------------------------------------------------------------------------------------------------------------------------------------------------------------------------------------------------------------------------------------------------------------------------------------------------------------------------------------------------------------------------------------------

Otus_spilocephalus_vulpes_96 ------------------------------------------------------------------------------------------------------------------------------------------------------------------------------------------------------------------------------------------------------------------------------------------------------------------------------------------------------------------------------------------------------------------------------------------------------------------------------------------------------------------------------------------------------------------------------------------------------------------------------------------------------------------------------------------------------------------------------------------------------------------------------------------------------------------------------------------------------------------------------------------------------

Otus_spilocephalus_spilocephalus_90 ------------------------------------------------------------------------------------------------------------------------------------------------------------------------------------------------------------------------------------------------------------------------------------------------------------------------------------------------------------------------------------------------------------------------------------------------------------------------------------------------------------------------------------------------------------------------------------------------------------------------------------------------------------------------------------------------------------------------------------------------------------------------------------------------------------------------------------------------------------------------------------------------------

Otus_spilocephalus_hambroecki_92 ------------------------------------------------------------------------------------------------------------------------------------------------------------------------------------------------------------------------------------------------------------------------------------------------------------------------------------------------------------------------------------------------------------------------------------------------------------------------------------------------------------------------------------------------------------------------------------------------------------------------------------------------------------------------------------------------------------------------------------------------------------------------------------------------------------------------------------------------------------------------------------------------------

_Otus_spilocephalus_latouchi_CHIMERA ------------------------------------------------------------------------------------------------------------------------------------------------------------------------------------------------------------------------------------------------------------------------------------------------------------------------------------------------------------------------------------------------------------------------------------------------------------------------------------------------------------------------------------------------------------------------------------------------------------------------------------------------------------------------------------------------------------------------------------------------------------------------------------------------------------------------------------------------------------------------------------------------------

Otus_hartlaubi_13 gatgtggaagaacagatgatcttctgtatggagtcattatgaacttcagctggttgtacactatgattagaatagggcaatttgataaagcactttccgacatagaactggcttacacctactcacaagaaaaagagctgaaatttctggccagtaccctccgcagtataaagttcaaagtagtaaaatacccaggttcactctctgctgaattgcagcagaggcttctcccagtagtaagttcattgcccaaactcagacatctcctcttagaatgtgacaaggatggacccaagtactgctctatcgtccctttgcattcctccatggatgtgacttacagccccgagcgcctgccgctgtcatccagctgcatgcacgtcactgagattttgcctacttttaatcccagcacaattattgctgctttagaaaatggctccatcagtacttgggatgtagagacccgccagttactraggcagatcacaacagctccatctgttattttagggatgaagcttactagtgatgaaaagtatcttgtagtggctacaacaaaaaacactcttttgatatacgataacataaattcctgtcttctgtctgaggtagaaattaaggggtcaaaacattgtggaattggggggggg-------------------------------------------------------------------------------------------------------------------------------------------------------------------------------------------------------------------------

Otus_hartlaubi_15 gatgtggaagaacagatgatcttctgtatggagtcattatgaacttcagctggttgtacactatgattagaatagggcaatttgataaagcactttccgacatagaactggcttacacctactcacaagaaaaagagctgaaatttctggccagtaccctccgcagtataaagttcaaagtagtaaaatacccaggttcactctctgctgaattgcagcagaggcttctcccagtagtaagttcattgcccaaactcagacatctcctcttagaatgtgacaaggatggacccaagtactgctctatcgtccctttgcattcctccatggatgtgacttacagccccgagcgcctgccgctgtcatccagctgcatgcacgtcactgagattttgcctacttttaatcccagcacaattattgctgctttagaaaatggctccatcagtacttgggatgtagagacccgccagttactraggcagatcacaacagctccatctgttattttagggatgaagcttactagtgatgaaaagtatcttgtagtggctacaacaaaaaacactcttttgatatacgataacataaattcctgtcttctgtctgaggtagaaattaaggggtcaaaacattgtggaattggggggggg-------------------------------------------------------------------------------------------------------------------------------------------------------------------------------------------------------------------------

Otus_hartlaubi_16 gatgtggaagaacagatgatcttctgtatggagtcattatgaacttcagctggttgtacactatgattagaatagggcaatttgataaagcactttccgacatagaactggcttacacctactcacaagaaaaagagctgaaatttctggccagtaccctccgcagtataaagttcaaagtagtaaaatacccaggttcactctctgctgaattgcagcagaggcttctcccagtagtaagttcattgcccaaactcagacatctcctcttagaatgtgacaaggatggacccaagtactgctctatcgtccctttgcattcctccatggatgtgacttacagccccgagcgcctgccgctgtcatccagctgcatgcacgtcactgagattttgcctacttttaatcccagcacaattattgctgctttagaaaatggctccatcagtacttgggatgtagagacccgccagttactraggcagatcacaacagctccatctgttattttagggatgaagcttactagtgatgaaaagtatcttgtagtggctacaacaaaaaacactcttttgatatacgataacataaattcctgtcttctgtctgaggtagaaattaaggggtcaaaacattgtggaattggggggggg-------------------------------------------------------------------------------------------------------------------------------------------------------------------------------------------------------------------------

Otus_hartlaubi_14 gatgtggaagaacagatgatcttctgtatggagtcattatgaacttcagctggttgtacactatgattagaatagggcaatttgataaagcactttccgacatagaactggcttacacctactcacaagaaaaagagctgaaatttctggccagtaccctccgcagtataaagttcaaagtagtaaaatacccaggttcactctctgctgaattgcagcagaggcttctcccagtagtaagttcattgcccaaactcagacatctcctcttagaatgtgacaaggatggacccaagtactgctctatcgtccctttgcattcctccatggatgtgacttacagccccgagcgcctgccgctgtcatccagctgcatgcacgtcactgagattttgcctacttttaatcccagcacaattattgctgctttagaaaatggctccatcagtacttgggatgtagagacccgccagttactraggcagatcacaacagctccatctgttattttagggatgaagcttactagtgatgaaaagtatcttgtagtggctacaacaaaaaacactcttttgatatacgataacataaattcctgtcttctgtctgaggtagaaattaaggggtcaaaacattgtggaattggggggggg-------------------------------------------------------------------------------------------------------------------------------------------------------------------------------------------------------------------------

Otus_feae_45 gatgtggaagaacagatgatcttctgtatggagtcattatgaacttcagctggttgtacactatgattagaatagggcaatttgataaagcactttccgacatagaactggcttacacctactcacaagaaaaagagctgaaatttctggccagtaccctccgcagtataaagttcaaagtagtaaaatacccaggttcactctctgctgaattgcagcagaggcttctcccagtagtaagttcattgcccaaactcagacatctcctcttagaatgtgacaaggatggacccaagtactgctctatcgtccctttgcattcctccatggatgtgacttacagccccgagcgcctgccgctgtcatccagctgcatgcacgtcactgagattttgcctacttttaatcccagcacaattattgctgctttagaaaatggctccatcagtacttgggatgtagagacccgccagttactgaggcagatcacaacagctccatccgttattttagggatgaagcttactagtgatgaaaagtatcttgtagtggctacaacaaaaaacactcttttgatatacgataacataaattcctgtcttctgtctgaggtagaaattaaggggtcaaaacattgtggaattgggggggggtccagttttataaatggatttacattatcagtgaaccatgcacttgcttggctggaggccagcaaaratgttactgttatagatctgctttatggttggcctctctatcacttccactgctggtatgaagtgacctgtgtgcagtgttctccagatggagtttatgcattctgcggacagtatttgaacaccgcaaccatttttcacttgggcagcg

Otus_senegalensis_17 ------------------------------------------------------------------------------------------------------------------------------------------------------------------------------------------------------------------------------------------------------------------------------------------------------------------------------------------------------------------------------------------------------------------------------------------------------------------------------------------------------------------------------------------------------------------------------------------------------------------------------------------------------------------------------------------------------------------------------------------------------------------------------------------------------------------------------------------------------------------------------------------------------

Otus_pamelae_74 ------------------------------------------------------------------------------------------------------------------------------------------------------------------------------------------------------------------------------------------------------------------------------------------------------------------------------------------------------------------------------------------------------------------------------------------------------------------------------------------------------------------------------------------------------------------------------------------------------------------------------------------------------------------------------------------------------------------------------------------------------------------------------------------------------------------------------------------------------------------------------------------------------

Otus_sp_nov_1 gatgtggaagaacagatgatcttctgtatggagtcattatgaacttcagctggttgtacactatgattagaatagggcaatttgataaagcactttccgacatagaactggcttacacctactcacaagaaaaagagctgaaatttctggccagtaccctccgcagtataaagttcaaagtagtaaaatacccaggttcactctctgctgaattgcagcagaggcttctcccagtagtaagttcattgcccaaactcagacatctcctcttagaatgtgacaaggatggacccaagtactgctctatcgtccctttgcattcctccatggatgtgacttacagccctgagcgcctgccgctgtcatccagctgcatgcacgtcactgagattttgcctacttttaatcccagcacaattattgctgctttagaaaatggctccatcagtacttgggatgtagagacccgccagttactgaggcagatcacaacagctccatctgttattttagggatgaagcttactagtgatgaaaagtatcttgtagtggctacaacaaaaaacactcttttgatatacgataacataaattcctgtcttctgtctgaggtagaaattaaggggtcaaaacattgtggaattggggggggg-------------------------------------------------------------------------------------------------------------------------------------------------------------------------------------------------------------------------

Otus_sp_nov_2 ------------------------------------------------------------------------------------------------------------------------------------------------------------------------------------------------------------------------------------------------------------------------------------------------------------------------------------------------------------------------------------------------------------------------------------------------------------------------------------------------------------------------------------------------------------------------------------------------------------------------------------------------------------------------------------------------------------------------------------------------------------------------------------------------------------------------------------------------------------------------------------------------------

Otus_sp_nov_3 ------------------------------------------------------------------------------------------------------------------------------------------------------------------------------------------------------------------------------------------------------------------------------------------------------------------------------------------------------------------------------------------------------------------------------------------------------------------------------------------------------------------------------------------------------------------------------------------------------------------------------------------------------------------------------------------------------------------------------------------------------------------------------------------------------------------------------------------------------------------------------------------------------

Otus_sp_nov_4 gatgtggaagaacagatgatcttctgtatggagtcattatgaacttcagctggttgtacactatgattagaatagggcaatttgataaagcactttccgacatagaactggcttacacctactcacaagaaaaagagctgaaatttctggccagtaccctccgcagtataaagttcaaagtagtaaaatacccaggttcactctctgctgaattgcagcagaggcttctcccagtagtaagttcattgcccaaactcagacatctcctcttagaatgtgacaaggatggacccaagtactgctctatcgtccctttgcattcctccatggatgtgacttacagccctgagcgcctgccgctgtcatccagctgcatgcacgtcactgagattttgcctacttttaatcccagcacaattattgctgctttagaaaatggctccatcagtacttgggatgtagagacccgccagttactgaggcagatcacaacagctccatctgttattttagggatgaagcttactagtgatgaaaagtatcttgtagtggctacaacaaaaaacactcttttgatatacgataacataaattcctgtcttctgtctgaggtagaaattaaggggtcaaaacattgtggaattgggggggggtccagttttataaakggatttacattatcagtgaaccatgcacttgcttggytggaggccagcaaaratgttactgttataratctgctttatggttggcctctctatcacttccactgctggtatgaagtgacctgtgtgcagkgttctccaracggagtttatgcattctgcggacagtatttgaacaccgcaaccatttttcacttgggcagcg

Otus_scops_5 ------------------------------------------------------------------------------------------------------------------------------------------------------------------------------------------------------------------------------------------------------------------------------------------------------------------------------------------------------------------------------------------------------------------------------------------------------------------------------------------------------------------------------------------------------------------------------------------------------------------------------------------------------------------------------------------------------------------------------------------------------------------------------------------------------------------------------------------------------------------------------------------------------

_Otus_scops_JF5337_F ------------------------------------------------------------------------------------------------------------------------------------------------------------------------------------------------------------------------------------------------------------------------------------------------------------------------------------------------------------------------------------------------------------------------------------------------------------------------------------------------------------------------------------------------------------------------------------------------------------------------------------------------------------------------------------------------------------------------------------------------------------------------------------------------------------------------------------------------------------------------------------------------------

Otus_pembaensis_24 ------------------------------------------------------------------------------------------------------------------------------------------------------------------------------------------------------------------------------------------------------------------------------------------------------------------------------------------------------------------------------------------------------------------------------------------------------------------------------------------------------------------------------------------------------------------------------------------------------------------------------------------------------------------------------------------------------------------------------------------------------------------------------------------------------------------------------------------------------------------------------------------------------

Otus_pembaensis_25 ------------------------------------------------------------------------------------------------------------------------------------------------------------------------------------------------------------------------------------------------------------------------------------------------------------------------------------------------------------------------------------------------------------------------------------------------------------------------------------------------------------------------------------------------------------------------------------------------------------------------------------------------------------------------------------------------------------------------------------------------------------------------------------------------------------------------------------------------------------------------------------------------------

_Otus_longicornis_CHIMERA ------------------------------------------------------------------------------------------------------------------------------------------------------------------------------------------------------------------------------------------------------------------------------------------------------------------------------------------------------------------------------------------------------------------------------------------------------------------------------------------------------------------------------------------------------------------------------------------------------------------------------------------------------------------------------------------------------------------------------------------------------------------------------------------------------------------------------------------------------------------------------------------------------

Otus_mirus_CHIMERA ------------------------------------------------------------------------------------------------------------------------------------------------------------------------------------------------------------------------------------------------------------------------------------------------------------------------------------------------------------------------------------------------------------------------------------------------------------------------------------------------------------------------------------------------------------------------------------------------------------------------------------------------------------------------------------------------------------------------------------------------------------------------------------------------------------------------------------------------------------------------------------------------------

Otus_mayottensis_60 ------------------------------------------------------------------------------------------------------------------------------------------------------------------------------------------------------------------------------------------------------------------------------------------------------------------------------------------------------------------------------------------------------------------------------------------------------------------------------------------------------------------------------------------------------------------------------------------------------------------------------------------------------------------------------------------------------------------------------------------------------------------------------------------------------------------------------------------------------------------------------------------------------

Otus_capnodes_40 ------------------------------------------------------------------------------------------------------------------------------------------------------------------------------------------------------------------------------------------------------------------------------------------------------------------------------------------------------------------------------------------------------------------------------------------------------------------------------------------------------------------------------------------------------------------------------------------------------------------------------------------------------------------------------------------------------------------------------------------------------------------------------------------------------------------------------------------------------------------------------------------------------

_Otus_madagascariensis_CHIMERA ------------------------------------------------------------------------------------------------------------------------------------------------------------------------------------------------------------------------------------------------------------------------------------------------------------------------------------------------------------------------------------------------------------------------------------------------------------------------------------------------------------------------------------------------------------------------------------------------------------------------------------------------------------------------------------------------------------------------------------------------------------------------------------------------------------------------------------------------------------------------------------------------------

Otus_pauliani_76 ------------------------------------------------------------------------------------------------------------------------------------------------------------------------------------------------------------------------------------------------------------------------------------------------------------------------------------------------------------------------------------------------------------------------------------------------------------------------------------------------------------------------------------------------------------------------------------------------------------------------------------------------------------------------------------------------------------------------------------------------------------------------------------------------------------------------------------------------------------------------------------------------------

Otus_insularis_47 ------------------------------------------------------------------------------------------------------------------------------------------------------------------------------------------------------------------------------------------------------------------------------------------------------------------------------------------------------------------------------------------------------------------------------------------------------------------------------------------------------------------------------------------------------------------------------------------------------------------------------------------------------------------------------------------------------------------------------------------------------------------------------------------------------------------------------------------------------------------------------------------------------

Otus_socotranus_89 ------------------------------------------------------------------------------------------------------------------------------------------------------------------------------------------------------------------------------------------------------------------------------------------------------------------------------------------------------------------------------------------------------------------------------------------------------------------------------------------------------------------------------------------------------------------------------------------------------------------------------------------------------------------------------------------------------------------------------------------------------------------------------------------------------------------------------------------------------------------------------------------------------

_Otus_sunia_CHIMERA ------------------------------------------------------------------------------------------------------------------------------------------------------------------------------------------------------------------------------------------------------------------------------------------------------------------------------------------------------------------------------------------------------------------------------------------------------------------------------------------------------------------------------------------------------------------------------------------------------------------------------------------------------------------------------------------------------------------------------------------------------------------------------------------------------------------------------------------------------------------------------------------------------

Otus_moheliensis_CHIMERA gatgtggaagaacagatgatcttctgtatggagtcattatgaacttcagctggttgtacactatgattagaatagggcaatttgataaagcactttccgacatagaactggcttacacctactcacaagaaaaagagctgaaatttctggccagtaccctccgcagtataaagttcaaagtagtaaaatacccaggttcactctctgctgaattgcagcagaggcttctcccagtagtaagttcattgcccaaactcagacatctcctcttagaatgtgacaaggatggacccaagtactgctctatcgtccctttgcattcctccatggatgtgacttacagccccgagcgcctgccgctgtcatccagctgcatgcacgtcactgagattttgcctacttttaatcccagcacaattattgctgctttagaaaatggctccatcagtacttgggatgtagagacccgccagttactgaggcagatcacaacagctccatctgttattttagggatgaagcttactagtgatgaaaagtatcttgtagtggctacaacaaaaaacactcttttgatatacgataacataaattcctgtcttctgtctgaggtagaaattaaggggtcaaaacattgtggaattggggggggg-------------------------------------------------------------------------------------------------------------------------------------------------------------------------------------------------------------------------

Otus_brucei_CHIMERA ------------------------------------------------------------------------------------------------------------------------------------------------------------------------------------------------------------------------------------------------------------------------------------------------------------------------------------------------------------------------------------------------------------------------------------------------------------------------------------------------------------------------------------------------------------------------------------------------------------------------------------------------------------------------------------------------------------------------------------------------------------------------------------------------------------------------------------------------------------------------------------------------------

Otus_icterorhynchus_holerythrus_27 ------------------------------------------------------------------------------------------------------------------------------------------------------------------------------------------------------------------------------------------------------------------------------------------------------------------------------------------------------------------------------------------------------------------------------------------------------------------------------------------------------------------------------------------------------------------------------------------------------------------------------------------------------------------------------------------------------------------------------------------------------------------------------------------------------------------------------------------------------------------------------------------------------

Otus_icterorhynchus_CHIMERA ------------------------------------------------------------------------------------------------------------------------------------------------------------------------------------------------------------------------------------------------------------------------------------------------------------------------------------------------------------------------------------------------------------------------------------------------------------------------------------------------------------------------------------------------------------------------------------------------------------------------------------------------------------------------------------------------------------------------------------------------------------------------------------------------------------------------------------------------------------------------------------------------------

Otus_ireneae_49 ------------------------------------------------------------------------------------------------------------------------------------------------------------------------------------------------------------------------------------------------------------------------------------------------------------------------------------------------------------------------------------------------------------------------------------------------------------------------------------------------------------------------------------------------------------------------------------------------------------------------------------------------------------------------------------------------------------------------------------------------------------------------------------------------------------------------------------------------------------------------------------------------------

Otus_rutilus_78 ------------------------------------------------------------------------------------------------------------------------------------------------------------------------------------------------------------------------------------------------------------------------------------------------------------------------------------------------------------------------------------------------------------------------------------------------------------------------------------------------------------------------------------------------------------------------------------------------------------------------------------------------------------------------------------------------------------------------------------------------------------------------------------------------------------------------------------------------------------------------------------------------------

Otus_scops_scops_8 gatgtggaagaacagatgatcttctgtatggagtcattatgaacttcagctggttgtacactatgattagaatagggcaatttgataaagcactttccgacatagaactggcttacacctactcacaagaaaaagagctgaaatttctggccagtaccctccgcagtataaagttcaaagtagtaaaatacccaggttcactctctgctgaattgcagcagaggcttctcccagtagtaagttcattgcccaaactcagacatctcctcttagaatgtgacaaggatggacccaagtactgctctatcgtccctttgcattcctccatggatgtgacttacagccccgagcgcctgccgctgtcatccagctgcatgcacgtcactgagattttgcctacttttaatcccagcacaattattgctgctttagaaaatggctccatcagtacttgggatgtagagacccgccagttactgaggcagatcacaacagctccatctgttattttagggatgaagcttactagtgatgaaaagtatcttgtagtggctacaacaaaaaacactcttttgatatacgataacataaattcctgtcttctgtctgaggtagaaattaaggggtcaaagcattgtggaattgggggggggtccagttttataaakggatttacattatcagtgaaccatgcacttgcttggctggaggccagcaaagatgtcactgttataratctgctttatggttggcctctctatcacttccactgctggtatgaagtgacctgtgtgcagkgttctccaratggagtttatgcattctgcggacagtatttgaacaccgcaaccatttttcacttgggcagcg

Otus_scops_scops_9 gatgtggaagaacagatgatcttctgtatggagtcattatgaacttcagctggttgtacactatgattagaatagggcaatttgataaagcactttccgacatagaactggcttacacctactcacaagaaaaagagctgaaatttctggccagtaccctccgcagtataaagttcaaagtagtaaaatacccaggttcactctctgctgaattgcagcagaggcttctcccagtagtaagttcattgcccaaactcagacatctcctcttagaatgtgacaaggatggacccaagtactgctctatcgtccctttgcattcctccatggatgtgacttacagccccgagcgcctgccgctgtcatccagctgcatgcacgtcactgagattttgcctacttttaatcccagcacaattattgctgctttagaaaatggctccatcagtacttgggatgtagagacccgccagttactgaggcagatcacaacagctccatctgttattttagggatgaagcttactagtgatgaaaagtatcttgtagtggctacaacaaaaaacactcttttgatatacgataacataaattcctgtcttctgtctgaggtagaaattaaggggtcaaagcattgtggaattggggggggg-------------------------------------------------------------------------------------------------------------------------------------------------------------------------------------------------------------------------

Otus_spilocephalus_luciae_94 ------------------------------------------------------------------------------------------------------------------------------------------------------------------------------------------------------------------------------------------------------------------------------------------------------------------------------------------------------------------------------------------------------------------------------------------------------------------------------------------------------------------------------------------------------------------------------------------------------------------------------------------------------------------------------------------------------------------------------------------------------------------------------------------------------------------------------------------------------------------------------------------------------

Otus_silvicola_87 ------------------------------------------------------------------------------------------------------------------------------------------------------------------------------------------------------------------------------------------------------------------------------------------------------------------------------------------------------------------------------------------------------------------------------------------------------------------------------------------------------------------------------------------------------------------------------------------------------------------------------------------------------------------------------------------------------------------------------------------------------------------------------------------------------------------------------------------------------------------------------------------------------

_Otus_cyprius_CHIMERA ------------------------------------------------------------------------------------------------------------------------------------------------------------------------------------------------------------------------------------------------------------------------------------------------------------------------------------------------------------------------------------------------------------------------------------------------------------------------------------------------------------------------------------------------------------------------------------------------------------------------------------------------------------------------------------------------------------------------------------------------------------------------------------------------------------------------------------------------------------------------------------------------------

Otus_elegans_CHIMERA ------------------------------------------------------------------------------------------------------------------------------------------------------------------------------------------------------------------------------------------------------------------------------------------------------------------------------------------------------------------------------------------------------------------------------------------------------------------------------------------------------------------------------------------------------------------------------------------------------------------------------------------------------------------------------------------------------------------------------------------------------------------------------------------------------------------------------------------------------------------------------------------------------

[marker: TTN; length: 884; no. of seqs: 51; new coordinates: 5434-6317)]

Bubo_bubo_111 --------------------------------------------------------------------------------------------------------------------------------------------------------------------------------------------------------------------------------------------------------------------------------------------------------------------------------------------------------------------------------------------------------------------------------------------------------------------------------------------------------------------------------------------------------------------------------------------------------------------------------------------------------------------------------------------------------------------------------------------------------------------------------------------------------------------------------------------------------------------------------------------------------------------

Strix_aluco_129 --------------------------------------------------------------------------------------------------------------------------------------------------------------------------------------------------------------------------------------------------------------------------------------------------------------------------------------------------------------------------------------------------------------------------------------------------------------------------------------------------------------------------------------------------------------------------------------------------------------------------------------------------------------------------------------------------------------------------------------------------------------------------------------------------------------------------------------------------------------------------------------------------------------------

Otus_bakkamoena_marathae_35 --------------------------------------------------------------------------------------------------------------------------------------------------------------------------------------------------------------------------------------------------------------------------------------------------------------------------------------------------------------------------------------------------------------------------------------------------------------------------------------------------------------------------------------------------------------------------------------------------------------------------------------------------------------------------------------------------------------------------------------------------------------------------------------------------------------------------------------------------------------------------------------------------------------------

_Otus_lempiji_CHIMERA --------------------------------------------------------------------------------------------------------------------------------------------------------------------------------------------------------------------------------------------------------------------------------------------------------------------------------------------------------------------------------------------------------------------------------------------------------------------------------------------------------------------------------------------------------------------------------------------------------------------------------------------------------------------------------------------------------------------------------------------------------------------------------------------------------------------------------------------------------------------------------------------------------------------

Otus_lettia_lettia_CHIMERA --------------------------------------------------------------------------------------------------------------------------------------------------------------------------------------------------------------------------------------------------------------------------------------------------------------------------------------------------------------------------------------------------------------------------------------------------------------------------------------------------------------------------------------------------------------------------------------------------------------------------------------------------------------------------------------------------------------------------------------------------------------------------------------------------------------------------------------------------------------------------------------------------------------------

Otus_megalotis_CHIMERA --------------------------------------------------------------------------------------------------------------------------------------------------------------------------------------------------------------------------------------------------------------------------------------------------------------------------------------------------------------------------------------------------------------------------------------------------------------------------------------------------------------------------------------------------------------------------------------------------------------------------------------------------------------------------------------------------------------------------------------------------------------------------------------------------------------------------------------------------------------------------------------------------------------------

Otus_nigrorum_CHIMERA --------------------------------------------------------------------------------------------------------------------------------------------------------------------------------------------------------------------------------------------------------------------------------------------------------------------------------------------------------------------------------------------------------------------------------------------------------------------------------------------------------------------------------------------------------------------------------------------------------------------------------------------------------------------------------------------------------------------------------------------------------------------------------------------------------------------------------------------------------------------------------------------------------------------

Otus_everetti_CHIMERA --------------------------------------------------------------------------------------------------------------------------------------------------------------------------------------------------------------------------------------------------------------------------------------------------------------------------------------------------------------------------------------------------------------------------------------------------------------------------------------------------------------------------------------------------------------------------------------------------------------------------------------------------------------------------------------------------------------------------------------------------------------------------------------------------------------------------------------------------------------------------------------------------------------------

Otus_semitorques_CHIMERA --------------------------------------------------------------------------------------------------------------------------------------------------------------------------------------------------------------------------------------------------------------------------------------------------------------------------------------------------------------------------------------------------------------------------------------------------------------------------------------------------------------------------------------------------------------------------------------------------------------------------------------------------------------------------------------------------------------------------------------------------------------------------------------------------------------------------------------------------------------------------------------------------------------------

Otus_angelinae_29 --------------------------------------------------------------------------------------------------------------------------------------------------------------------------------------------------------------------------------------------------------------------------------------------------------------------------------------------------------------------------------------------------------------------------------------------------------------------------------------------------------------------------------------------------------------------------------------------------------------------------------------------------------------------------------------------------------------------------------------------------------------------------------------------------------------------------------------------------------------------------------------------------------------------

Otus_spilocephalus_vandewateri_97 --------------------------------------------------------------------------------------------------------------------------------------------------------------------------------------------------------------------------------------------------------------------------------------------------------------------------------------------------------------------------------------------------------------------------------------------------------------------------------------------------------------------------------------------------------------------------------------------------------------------------------------------------------------------------------------------------------------------------------------------------------------------------------------------------------------------------------------------------------------------------------------------------------------------

Otus_spilocephalus_vulpes_96 --------------------------------------------------------------------------------------------------------------------------------------------------------------------------------------------------------------------------------------------------------------------------------------------------------------------------------------------------------------------------------------------------------------------------------------------------------------------------------------------------------------------------------------------------------------------------------------------------------------------------------------------------------------------------------------------------------------------------------------------------------------------------------------------------------------------------------------------------------------------------------------------------------------------

Otus_spilocephalus_spilocephalus_90 --------------------------------------------------------------------------------------------------------------------------------------------------------------------------------------------------------------------------------------------------------------------------------------------------------------------------------------------------------------------------------------------------------------------------------------------------------------------------------------------------------------------------------------------------------------------------------------------------------------------------------------------------------------------------------------------------------------------------------------------------------------------------------------------------------------------------------------------------------------------------------------------------------------------

Otus_spilocephalus_hambroecki_92 --------------------------------------------------------------------------------------------------------------------------------------------------------------------------------------------------------------------------------------------------------------------------------------------------------------------------------------------------------------------------------------------------------------------------------------------------------------------------------------------------------------------------------------------------------------------------------------------------------------------------------------------------------------------------------------------------------------------------------------------------------------------------------------------------------------------------------------------------------------------------------------------------------------------

_Otus_spilocephalus_latouchi_CHIMERA --------------------------------------------------------------------------------------------------------------------------------------------------------------------------------------------------------------------------------------------------------------------------------------------------------------------------------------------------------------------------------------------------------------------------------------------------------------------------------------------------------------------------------------------------------------------------------------------------------------------------------------------------------------------------------------------------------------------------------------------------------------------------------------------------------------------------------------------------------------------------------------------------------------------

Otus_hartlaubi_13 -------------------------------------------------ttacagaagaccagcgatatgaattccgtgtgattgcaaagaatgccgctggactcttcagtcagccatctgaatcaactggacctgtgactgtaaaagatgatgtagaggctccaagaattatgatggatgccaaattcagggatgttgtagttgtgaaagctggagaagtgtttaaagtcaatgctgatattgcagggcgcccgataccagtgatttcatggacaaaggatggcaaggagcttgaaggaaaagctagagttgaaatagtctcaacagatcacactactgcaataaccgttaaggactgtatccgaggtgattcaggacagtatgtactaacattacaaaatgttgctggaacaagatctttggcaattaattgcaaagtacttgatagacctggcccacctgcaggcccattagaaataaatggccttactgctgaaaagtgccatttatcatggggaccccctcaagaaaatggcggtgcagatattgattattatgttgtagaaaaacgtgagaccagcagaattgcatggacactttgtgaaggagagcttagaacaacatcctgtaaggtgacaaaactattgaagggtaatgagtatattttcagagtgatgggagttaacaaatatggtgttggtgagcctctagaaagtgttgctgtcaaagccctagacccatttacagttccaagtccacccacatctttagagatcaccaatgtgagcaaagagtcaataactctgtgctgggcaagacctgagtctgatggaggcaatgagatctctggctatgtaattgaaagg-----------------

Otus_hartlaubi_15 --------------------------------------------------------------------------------------------------------------------atctgaatcaactggacctgtgactgtaraagatgatgtagaggctccaagaattatgatggatgccaaattcagggatgttgtagttgtgaaagctggagaagtgtttaaagtcaatgctgatattgcagggcgcccgataccagtgatttcatggacaaaggatggcaaggagcttgaaggaaaagctagagttgaaatagtctcaacagatcacactactgcaataaccgttaaggactgtatccgaggtgattcaggacagtatgtactaacattacaaaatgttgctggaacaagatctttggcaattaattgcaaagtacttgatagacctggcccacctgcaggcccattagaaataaatggccttactgctgaaaagtgccatttatcatggggaccccctcaagaaaatggcggtgcagatattgattattatgttgtagaaaaacgtgagaccagcagaattgcatggacactttgtgaaggagagcttagaacaacatcctgtaaggtgacaaaactattgaagggtaatgagtatattttcagagtgatgggagttaacaaatatggtgttggtgagcctctagaaagtgttgctgtcaaagccctagacccatttacagttccaagtccacccacatctttagagatcaccaatgtgagcaaagagtcaataactctgtgctgggcaagacctgagtctgatggaggcaatgagatctctggctatgtaattgaaaggcgtgagaaaactagcct

Otus_hartlaubi_16 aagtgcaattttactaatgtgcaagaaacatactttgatgtaggtggacttacagaagaccagcgatatgaattccgtgtgattgcaaagaatgccgctggactcttcagtcagccatctgaatcaactggacctgtgactgtaaaagatgatgtagaggctccaagaattatgatggatgccaaattcagggatgttgtagttgtgaaagctggagaagtgtttaaagtcaatgctgatattgcagggcgcccgataccagtgatttcatggacaaaggatggcaaggagcttgaaggaaaagctagagttgaaatagtctcaacagatcacactactgcaataaccgttaaggactgtatccgaggtgattcaggacagtatgtactaacattacaaaatgttgctggaacaagatctttggcaattaattgcaaagtacttgatagacctggcccacctgcaggcccattagaaataaatggccttactgctgaaaagtgccatttatcatggggaccccctcaagaaaatggcggtgcagatattgattattatgttgtagaaaaacgtgagaccagcagaattgcatggacactttgtgaaggagagcttagaacaacatcctgtaaggtgacaaaactattgaagggtaatgagtatattttcagagtgatgggagttaacaaatatggtgttggtgagcctctagaaagtgttgctgtcaaagccctagacccatttacagttccaagtccacccacatctttagagatcaccaatgtgagcaaagagtcaataactctgtgctgggcaagacctgagtctgatggaggcaatgagatctctggctatgtaattgaaaggcgtgagaaaactagcct

Otus_hartlaubi_14 -----------------------------------------------------------------------------------------------------------------------------------------------------------------tccaagaattatgatggatgccaaattcagggatgttgtagttgtgaaagctggagaagtgtttaaagtcaatgctgatattgcagggcgcccgataccagtgatttcatggacaaaggatggcaaggagcttgaaggaaaagctagagttgaaatagtctcaacagatcacactactgcaataaccgttaaggactgtatccgaggtgattcaggacagtatgtactaacattacaaaatgttgctggaacaagatctttggcaattaattgcaaagtacttgatagacctggcccacctgcaggcccattagaaataaatggccttactgctgaaaagtgccatttatcatggggaccccctcaagaaaatggcggtgcagatattgattattatgttgtagaaaaacgtgagaccagcagaattgcatggacactttgtgaaggagagcttagaacaacatcctgtaaggtgacaaaactattgaagggtaatgagtatattttcagagtgatgggagttaacaaatatggtgttggtgagcctctagaaagtgttgctgtcaaagccctagacccatttacagttccaagtccacccacatctttagagatcaccaatgtgagcaaagagtcaataactctgtgctgggcaagacctgagtctgatggaggcaatgagatctctggctatgtaattgaaaggcgtgagaaaactagcct

Otus_feae_45 ------------------------------------------------------------------------ttccgtgygattgcaaagaatgccgctggactcttcagtcakccatctgaatcaactggayctgtgactgtaaaagatgatgtagaggmtccaagaattatgatggatgccaaattcagggatgttgtagttgtgaaagctggagaagtgtttaaagtcaatgctgatattgcagggcggccgataccagtgatttcatggacaaaggatggcaaggagcttgaaggaaaagctagagttgaaatagtctcaacagatcacactactgcaataaccgttaaggactgtatccgaggtgattcaggacagtatgtactaacattacaaaatgttgctggaacaagatctttggcaattaattgcaaagtacttgatagacctggcccacctgcaggcccattagaaataaatggccttactgctgaaaagtgccatttatcatggggaccccctcaagaaaatggcggtgcagatattgattattatgttgtagaaaaacgtgagaccagcagaattgcatggacactttgtgaaggagagcttagaacaacatcctgtaaggtgacaaaactattgaagggtaatgagtatattttcagagtgatgggagttaacaaatatggtgttggtgagcctctagaaagtgttgctgtcaaagccctagacccatttacagttccaagtccacccacatctttagagatcaccaatgtgagcaaagagtcaataactctgtgctgggcaagacctgagtctgatggaggcaatgagatctctggctatgtaattgaaaggcgtgagaaaactagcct

Otus_senegalensis_17 --------------------------------------------------------------------------------------------------------------------------------------------------------------------------------------------------------------------------------------------------------------------------------------------------------------------------------------------------------------------------------------------------------------------------------------------------------------------------------------------------------------------------------------------------------------------------------------------------------------------------------------------------------------------------------------------------------------------------------------------------------------------------------------------------------------------------------------------------------------------------------------------------------------------

Otus_pamelae_74 --------------------------------------------------------------------------------------------------------------------------------------------------------------------------------------------------------------------------------------------------------------------------------------------------------------------------------------------------------------------------------------------------------------------------------------------------------------------------------------------------------------------------------------------------------------------------------------------------------------------------------------------------------------------------------------------------------------------------------------------------------------------------------------------------------------------------------------------------------------------------------------------------------------------

Otus_sp_nov_1 ---------------------------------------------------------------------------------------------------------------------------tcaactggacctgtgactgtaraagatgatgtagaggctccaagaattatgatggatgccaaattcagggatgttgtagttgtgaaagctggagaagtgtttaaagtcaatgctgatattgcagggcggccgataccagtgatttcatggacaaaggatggcaaggagcttgaaggaaaagctagagttgaaatagtctcaacagatcacactactgcaataaccgttaaggactgtatccgaggtgattcaggacagtatgtactaacattacaaaatgttgctggaacaagatctttggcaattaattgcaaagtacttgatagacctggcccacctgcaggcccattagaaataaatggccttactgctgaaaagtgccatttatcatggggaccccctcaagaaaatggcggtgcagatattgattattatgttgtagaaaaacgtgagaccagcagaattgcatggacactttgtgaaggagagcttagaacaacatcctgtaaggtgacaaaactattgaagggtaatgagtatattttcagagtgatgggagttaacaaatatggtgttggtgagcctctagaaagtgttgctgtcaaagccctagacccatttacagttccaagtccacccacatctttagagatcaccaatgtgagcaaagagtcaataactctgtgctgggcaagacctgagtctgatggaggcaatgagatctctggctatgtaattgaaaggcgtgagaaaactagcct

Otus_sp_nov_2 aagtgcaattttactaatgtgcaagaaacatactttgatgtaggtggacttacagaagaccagcgatatgaattccgtgtgattgcaaagaatgccgctggactcttcagtcagccatctgaatcaactggacctgtgactgtaaaagatgatgtagaggctccaagaattatgatggatgccaaattcagggatgttgtagttgtgaaagctggagaagtgtttaaagtcaatgctgatattgcagggcggccgataccagtgatttcatggacaaaggatggcaaggagcttgaaggaaaagctagagttgaaatagtctcaacagatcacactactgcaataaccgttaaggactgtatccgaggtgattcaggacagtatgtactaacattacaaaatgttgctggaacaagatctttggcaattaattgcaaagtacttgatagacctggcccacctgcaggcccattagaaataaatggccttactgctgaaaagtgccatttatcatggggaccccctcaagaaaatggcggtgcagatattgattattatgtwgtagaaaaacgtgagaccagcagaattgcatggacactttgtgaaggagagcttagaacaacatcctgtaaggtgacaaaactattgaagggtaatgagtatattttcagagtgatgggagttaacaaatatggtgttggtgagcctctagaaagtgttgctgtcaaagccctagacccatttacagttccaagtccacccacatctttagagatcaccaatgtgagcaaagagtcaataactctgtgctgggcaagacctgagtctgatggaggcaatgagatctctggctatgtaattgaaaggcgtgagaaaactagcct

Otus_sp_nov_3 aagtgcaattttactaatgtgcaagaaacatactttgatgtaggtggacttacagaagaccagcgatatgaattccgtgtgattgcaaagaatgccgctggactcttcagtcagccatctgaatcaactggacctgtgactgtaaaagatgatgtagaggctccaagaattatgatggatgccaaattcagggatgttgtagttgtgaaagctggagaagtgtttaaagtcaatgctgatattgcagggcggccgataccagtgatttcatggacaaaggatggcaaggagcttgaaggaaaagctagagttgaaatagtctcaacagatcacactactgcaataaccgttaaggactgtatccgaggtgattcaggacagtatgtactaacattacaaaatgttgctggaacaagatctttggcaattaattgcaaagtacttgatagacctggcccacctgcaggcccattagaaataaatggccttactgctgaaaagtgccatttatcatggggaccccctcaagaaaatggcggtgcagatattgattattatgttgtagaaaaacgtgagaccagcagaattgcatggacactttgtgaaggagagcttagaacaacatcctgtaaggtgacaaaactattgaagggtaatgagtatattttcagagtgatgggagttaacaaatatggtgttggtgagcctctagaaagtgttgctgtcaaagccctagacccatttacagttccaagtccacccacatctttagagatcaccaatgtgagcaaagagtcaataactctgtgctgggcaagacctgagtctgatggaggcaatgagatctctggctatgtaattgaaaggcgtgagaaaactagcct

Otus_sp_nov_4 aagtgcaattttactaatgtgcaagaaacatactttgatgtaggtggacttacagaagaccagcgatatgaattccgtgtgattgcaaagaatgccgctggactcttcagtcagccatctgaatcaactggacctgtgactgtaaaagatgatgtagaggctccaagaattatgatggatgccaaattcagggatgttgtagttgtgaaagctggagaagtgtttaaagtcaatgctgatattgcagggcggccgataccagtgatttcatggacaaaggatggcaaggagcttgaaggaaaagctagagttgaaatagtctcaacagatcacactactgcaataaccgttaaggactgtatccgaggtgattcaggacagtatgtactaacattacaaaatgttgctggaacaagatctttggcaattaattgcaaagtacttgatagacctggcccacctgcaggcccattagaaataaatggccttactgctgaaaagtgccatttatcatggggaccccctcaagaaaatggcggtgcagatattgattattatgttgtagaaaaacgtgagaccagcagaattgcatggacactttgtgaaggagagcttagaacaacatcctgtaaggtgacaaaactattgaagggtaatgagtatattttcagagtgatgggagttaacaaatatggtgttggtgagcctctagaaagtgttgctgtcaaagccctagacccatttacagttccaagtccacccacatctttagagatcaccaatgtgagcaaagagtcaataactctgtgctgggcaagacctgagtctgatggaggcaatgagatctctggctatgtaattgaaaggcgtgagaaaactagcct

Otus_scops_5 --------------------------------------------------------------------------------------------------------------------------------------------------------------------------------------------------------------------------------------------------------------------------------------------------------------------------------------------------------------------------------------------------------------------------------------------------------------------------------------------------------------------------------------------------------------------------------------------------------------------------------------------------------------------------------------------------------------------------------------------------------------------------------------------------------------------------------------------------------------------------------------------------------------------

_Otus_scops_JF5337_F --------------------------------------------------------------------------------------------------------------------------------------------------------------------------------------------------------------------------------------------------------------------------------------------------------------------------------------------------------------------------------------------------------------------------------------------------------------------------------------------------------------------------------------------------------------------------------------------------------------------------------------------------------------------------------------------------------------------------------------------------------------------------------------------------------------------------------------------------------------------------------------------------------------------

Otus_pembaensis_24 --------------------------------------------------------------------------------------------------------------------------------------------------------------------------------------------------------------------------------------------------------------------------------------------------------------------------------------------------------------------------------------------------------------------------------------------------------------------------------------------------------------------------------------------------------------------------------------------------------------------------------------------------------------------------------------------------------------------------------------------------------------------------------------------------------------------------------------------------------------------------------------------------------------------

Otus_pembaensis_25 --------------------------------------------------------------------------------------------------------------------------------------------------------------------------------------------------------------------------------------------------------------------------------------------------------------------------------------------------------------------------------------------------------------------------------------------------------------------------------------------------------------------------------------------------------------------------------------------------------------------------------------------------------------------------------------------------------------------------------------------------------------------------------------------------------------------------------------------------------------------------------------------------------------------

_Otus_longicornis_CHIMERA --------------------------------------------------------------------------------------------------------------------------------------------------------------------------------------------------------------------------------------------------------------------------------------------------------------------------------------------------------------------------------------------------------------------------------------------------------------------------------------------------------------------------------------------------------------------------------------------------------------------------------------------------------------------------------------------------------------------------------------------------------------------------------------------------------------------------------------------------------------------------------------------------------------------

Otus_mirus_CHIMERA --------------------------------------------------------------------------------------------------------------------------------------------------------------------------------------------------------------------------------------------------------------------------------------------------------------------------------------------------------------------------------------------------------------------------------------------------------------------------------------------------------------------------------------------------------------------------------------------------------------------------------------------------------------------------------------------------------------------------------------------------------------------------------------------------------------------------------------------------------------------------------------------------------------------

Otus_mayottensis_60 --------------------------------------------------------------------------------------------------------------------------------------------------------------------------------------------------------------------------------------------------------------------------------------------------------------------------------------------------------------------------------------------------------------------------------------------------------------------------------------------------------------------------------------------------------------------------------------------------------------------------------------------------------------------------------------------------------------------------------------------------------------------------------------------------------------------------------------------------------------------------------------------------------------------

Otus_capnodes_40 --------------------------------------------------------------------------------------------------------------------------------------------------------------------------------------------------------------------------------------------------------------------------------------------------------------------------------------------------------------------------------------------------------------------------------------------------------------------------------------------------------------------------------------------------------------------------------------------------------------------------------------------------------------------------------------------------------------------------------------------------------------------------------------------------------------------------------------------------------------------------------------------------------------------

_Otus_madagascariensis_CHIMERA --------------------------------------------------------------------------------------------------------------------------------------------------------------------------------------------------------------------------------------------------------------------------------------------------------------------------------------------------------------------------------------------------------------------------------------------------------------------------------------------------------------------------------------------------------------------------------------------------------------------------------------------------------------------------------------------------------------------------------------------------------------------------------------------------------------------------------------------------------------------------------------------------------------------

Otus_pauliani_76 --------------------------------------------------------------------------------------------------------------------------------------------------------------------------------------------------------------------------------------------------------------------------------------------------------------------------------------------------------------------------------------------------------------------------------------------------------------------------------------------------------------------------------------------------------------------------------------------------------------------------------------------------------------------------------------------------------------------------------------------------------------------------------------------------------------------------------------------------------------------------------------------------------------------

Otus_insularis_47 --------------------------------------------------------------------------------------------------------------------------------------------------------------------------------------------------------------------------------------------------------------------------------------------------------------------------------------------------------------------------------------------------------------------------------------------------------------------------------------------------------------------------------------------------------------------------------------------------------------------------------------------------------------------------------------------------------------------------------------------------------------------------------------------------------------------------------------------------------------------------------------------------------------------

Otus_socotranus_89 --------------------------------------------------------------------------------------------------------------------------------------------------------------------------------------------------------------------------------------------------------------------------------------------------------------------------------------------------------------------------------------------------------------------------------------------------------------------------------------------------------------------------------------------------------------------------------------------------------------------------------------------------------------------------------------------------------------------------------------------------------------------------------------------------------------------------------------------------------------------------------------------------------------------

_Otus_sunia_CHIMERA --------------------------------------------------------------------------------------------------------------------------------------------------------------------------------------------------------------------------------------------------------------------------------------------------------------------------------------------------------------------------------------------------------------------------------------------------------------------------------------------------------------------------------------------------------------------------------------------------------------------------------------------------------------------------------------------------------------------------------------------------------------------------------------------------------------------------------------------------------------------------------------------------------------------

Otus_moheliensis_CHIMERA ------------------------------------------------------------------------------------------------------------------------------------cctgtgactgtaaaagatgatgtagaggmtccaagaattatgatggatgccaaattcagggatgttgtagttgtgaaagctggagaagtgtttaaagtcaatgctgatattgcagggcggccgataccagtgatttcatggacaaaggatggcaaggagcttgaaggaaaagctagagttgaaatagtctcaacagatcacactactgcaataaccgttaaggactgtatccgaggtgattcaggacagtatgtactaacattacaaaatgttgctggaacaagatctttggcaattaattgcaaagtacttgatagacctgrccyacctgcaggcccattagaaataaatggccttactgctgaaaagtgccatttatcatggggaccccctcaagaaaatggcggtgcagatattgattattatgttgtagaaaaacgtgagaccagcagaattgcatggacactttgtgaaggagagcttagaacaacatcctgtaaggtgacaaaactattgaagggtaatgagtatattttcagagtgatgggagttaacaaatatggcgttggtgagcctctagaaagtgttgctgtcaaagccctagacccatttacagttccaagtccacccacatctttagagatcaccaatgtgagcaaagattcaataactctgtgctgggcaagacctgagtctgatggaggcaatgagatctctggctatgtaattgaaaggcgtgagaaaactagcct

Otus_brucei_CHIMERA --------------------------------------------------------------------------------------------------------------------------------------------------------------------------------------------------------------------------------------------------------------------------------------------------------------------------------------------------------------------------------------------------------------------------------------------------------------------------------------------------------------------------------------------------------------------------------------------------------------------------------------------------------------------------------------------------------------------------------------------------------------------------------------------------------------------------------------------------------------------------------------------------------------------

Otus_icterorhynchus_holerythrus_27 --------------------------------------------------------------------------------------------------------------------------------------------------------------------------------------------------------------------------------------------------------------------------------------------------------------------------------------------------------------------------------------------------------------------------------------------------------------------------------------------------------------------------------------------------------------------------------------------------------------------------------------------------------------------------------------------------------------------------------------------------------------------------------------------------------------------------------------------------------------------------------------------------------------------

Otus_icterorhynchus_CHIMERA --------------------------------------------------------------------------------------------------------------------------------------------------------------------------------------------------------------------------------------------------------------------------------------------------------------------------------------------------------------------------------------------------------------------------------------------------------------------------------------------------------------------------------------------------------------------------------------------------------------------------------------------------------------------------------------------------------------------------------------------------------------------------------------------------------------------------------------------------------------------------------------------------------------------

Otus_ireneae_49 --------------------------------------------------------------------------------------------------------------------------------------------------------------------------------------------------------------------------------------------------------------------------------------------------------------------------------------------------------------------------------------------------------------------------------------------------------------------------------------------------------------------------------------------------------------------------------------------------------------------------------------------------------------------------------------------------------------------------------------------------------------------------------------------------------------------------------------------------------------------------------------------------------------------

Otus_rutilus_78 --------------------------------------------------------------------------------------------------------------------------------------------------------------------------------------------------------------------------------------------------------------------------------------------------------------------------------------------------------------------------------------------------------------------------------------------------------------------------------------------------------------------------------------------------------------------------------------------------------------------------------------------------------------------------------------------------------------------------------------------------------------------------------------------------------------------------------------------------------------------------------------------------------------------

Otus_scops_scops_8 ------------------------------------------------------------cagcgatatgaattccgtgtgattgcaaagaatgccgctggactcttcagtcarccatcwgaatcaactggacctgtgactgtaaaagatgatgtagaggctccaagaattatgatggatgccaaattcagggatgttgtagttgtgaaagctggagaagtgtttaaagtcaatgctgatattgcagggcggccgataccagtgatttcatggacaaaggatggcaaggagcttgaaggaaaagctagagttgaaatagtctcaacagatcacactactgcaataaccgttaaggactgtatccgaggtgattcaggacagtatgtactaacattacaaaatgttgctggaacaagatctttggcaattaattgcaaagtacttgatagacctggcccacctgcaggcccattagaaataaatggccttactgctgaaaagtgccatttatcatggggaccccctcaagaaaatggcggtgcagatattgattattatgttgtagaaaaacgtgagaccagcagaattgcatggacactttgtgaaggagagcttagaacaacatcctgtaaggtgacaaaactattgaagggtaatgagtatattttcagagtgatgggagttaacaaatatggcattggtgagcctctagaaagtgttgctgtcaaagccctagacccatttacagttccaagtccacccacatctttagagatcaccaatgtgagcaaagattcaataactctgtgctgggcaagacctgagtctgatggaggcaatgagatctctggctatgtaattgaaaggcgtgagaaaactagcct

Otus_scops_scops_9 ---------------------------------------------------------------------------------------aagaatgccgctggactcttcagtcakccatcwgaatcwactggacctgtgactgtaaaagatgatgtagaggctccaagaattatgatggatgccaaattcagggatgttgtagttgtgaaagctggagaagtgtttaaagtcaatgctgatattgcagggcggccgataccagtgatttcatggacaaaggatggcaaggagcttgaaggaaaagctagagttgaaatagtctcaacagatcacactactgcaataaccgttaaggactgtatccgaggtgattcaggacagtatgtactaacattacaaaatgttgctggaacaagatctttggcaattaattgcaaagtacttgatagacctggcccacctgcaggcccattagaaataaatggccttactgctgaaaagtgccatttatcatggggaccccctcaagaaaatggcggtgcagatattgattattatgttgtagaaaaacgtgagaccagcagaattgcatggacactttgtgaaggagagcttagaacaacatcctgtaaggtgacaaaactattgaagggtaatgagtatattttcagagtgatgggagttaacaaatatggcattggtgagcctctagaaagtgttgctgtcaaagccctagacccatttacagttccaagtccacccacatctttagagatcaccaatgtgagcaaagattcaataactctgtgctgggcaagacctgagtctgatggaggcaatgagatctctggctatgtaattgaaaggcgtgagaaaactagcct

Otus_spilocephalus_luciae_94 --------------------------------------------------------------------------------------------------------------------------------------------------------------------------------------------------------------------------------------------------------------------------------------------------------------------------------------------------------------------------------------------------------------------------------------------------------------------------------------------------------------------------------------------------------------------------------------------------------------------------------------------------------------------------------------------------------------------------------------------------------------------------------------------------------------------------------------------------------------------------------------------------------------------

Otus_silvicola_87 --------------------------------------------------------------------------------------------------------------------------------------------------------------------------------------------------------------------------------------------------------------------------------------------------------------------------------------------------------------------------------------------------------------------------------------------------------------------------------------------------------------------------------------------------------------------------------------------------------------------------------------------------------------------------------------------------------------------------------------------------------------------------------------------------------------------------------------------------------------------------------------------------------------------

_Otus_cyprius_CHIMERA --------------------------------------------------------------------------------------------------------------------------------------------------------------------------------------------------------------------------------------------------------------------------------------------------------------------------------------------------------------------------------------------------------------------------------------------------------------------------------------------------------------------------------------------------------------------------------------------------------------------------------------------------------------------------------------------------------------------------------------------------------------------------------------------------------------------------------------------------------------------------------------------------------------------

Otus_elegans_CHIMERA --------------------------------------------------------------------------------------------------------------------------------------------------------------------------------------------------------------------------------------------------------------------------------------------------------------------------------------------------------------------------------------------------------------------------------------------------------------------------------------------------------------------------------------------------------------------------------------------------------------------------------------------------------------------------------------------------------------------------------------------------------------------------------------------------------------------------------------------------------------------------------------------------------------------

[marker: SACSA; length: 977; no. of seqs: 51; new coordinates: 6318-7294)]

Bubo_bubo_111 -----------------------------------------------------------------------------------------------------------------------------------------------------------------------------------------------------------------------------------------------------------------------------------------------------------------------------------------------------------------------------------------------------------------------------------------------------------------------------------------------------------------------------------------------------------------------------------------------------------------------------------------------------------------------------------------------------------------------------------------------------------------------------------------------------------------------------------------------------------------------------------------------------------------------------------------------------------------------------------------------------------------

Strix_aluco_129 -----------------------------------------------------------------------------------------------------------------------------------------------------------------------------------------------------------------------------------------------------------------------------------------------------------------------------------------------------------------------------------------------------------------------------------------------------------------------------------------------------------------------------------------------------------------------------------------------------------------------------------------------------------------------------------------------------------------------------------------------------------------------------------------------------------------------------------------------------------------------------------------------------------------------------------------------------------------------------------------------------------------

Otus_bakkamoena_marathae_35 -----------------------------------------------------------------------------------------------------------------------------------------------------------------------------------------------------------------------------------------------------------------------------------------------------------------------------------------------------------------------------------------------------------------------------------------------------------------------------------------------------------------------------------------------------------------------------------------------------------------------------------------------------------------------------------------------------------------------------------------------------------------------------------------------------------------------------------------------------------------------------------------------------------------------------------------------------------------------------------------------------------------

_Otus_lempiji_CHIMERA -----------------------------------------------------------------------------------------------------------------------------------------------------------------------------------------------------------------------------------------------------------------------------------------------------------------------------------------------------------------------------------------------------------------------------------------------------------------------------------------------------------------------------------------------------------------------------------------------------------------------------------------------------------------------------------------------------------------------------------------------------------------------------------------------------------------------------------------------------------------------------------------------------------------------------------------------------------------------------------------------------------------

Otus_lettia_lettia_CHIMERA -----------------------------------------------------------------------------------------------------------------------------------------------------------------------------------------------------------------------------------------------------------------------------------------------------------------------------------------------------------------------------------------------------------------------------------------------------------------------------------------------------------------------------------------------------------------------------------------------------------------------------------------------------------------------------------------------------------------------------------------------------------------------------------------------------------------------------------------------------------------------------------------------------------------------------------------------------------------------------------------------------------------

Otus_megalotis_CHIMERA -----------------------------------------------------------------------------------------------------------------------------------------------------------------------------------------------------------------------------------------------------------------------------------------------------------------------------------------------------------------------------------------------------------------------------------------------------------------------------------------------------------------------------------------------------------------------------------------------------------------------------------------------------------------------------------------------------------------------------------------------------------------------------------------------------------------------------------------------------------------------------------------------------------------------------------------------------------------------------------------------------------------

Otus_nigrorum_CHIMERA -----------------------------------------------------------------------------------------------------------------------------------------------------------------------------------------------------------------------------------------------------------------------------------------------------------------------------------------------------------------------------------------------------------------------------------------------------------------------------------------------------------------------------------------------------------------------------------------------------------------------------------------------------------------------------------------------------------------------------------------------------------------------------------------------------------------------------------------------------------------------------------------------------------------------------------------------------------------------------------------------------------------

Otus_everetti_CHIMERA -----------------------------------------------------------------------------------------------------------------------------------------------------------------------------------------------------------------------------------------------------------------------------------------------------------------------------------------------------------------------------------------------------------------------------------------------------------------------------------------------------------------------------------------------------------------------------------------------------------------------------------------------------------------------------------------------------------------------------------------------------------------------------------------------------------------------------------------------------------------------------------------------------------------------------------------------------------------------------------------------------------------

Otus_semitorques_CHIMERA -----------------------------------------------------------------------------------------------------------------------------------------------------------------------------------------------------------------------------------------------------------------------------------------------------------------------------------------------------------------------------------------------------------------------------------------------------------------------------------------------------------------------------------------------------------------------------------------------------------------------------------------------------------------------------------------------------------------------------------------------------------------------------------------------------------------------------------------------------------------------------------------------------------------------------------------------------------------------------------------------------------------

Otus_angelinae_29 -----------------------------------------------------------------------------------------------------------------------------------------------------------------------------------------------------------------------------------------------------------------------------------------------------------------------------------------------------------------------------------------------------------------------------------------------------------------------------------------------------------------------------------------------------------------------------------------------------------------------------------------------------------------------------------------------------------------------------------------------------------------------------------------------------------------------------------------------------------------------------------------------------------------------------------------------------------------------------------------------------------------

Otus_spilocephalus_vandewateri_97 -----------------------------------------------------------------------------------------------------------------------------------------------------------------------------------------------------------------------------------------------------------------------------------------------------------------------------------------------------------------------------------------------------------------------------------------------------------------------------------------------------------------------------------------------------------------------------------------------------------------------------------------------------------------------------------------------------------------------------------------------------------------------------------------------------------------------------------------------------------------------------------------------------------------------------------------------------------------------------------------------------------------

Otus_spilocephalus_vulpes_96 -----------------------------------------------------------------------------------------------------------------------------------------------------------------------------------------------------------------------------------------------------------------------------------------------------------------------------------------------------------------------------------------------------------------------------------------------------------------------------------------------------------------------------------------------------------------------------------------------------------------------------------------------------------------------------------------------------------------------------------------------------------------------------------------------------------------------------------------------------------------------------------------------------------------------------------------------------------------------------------------------------------------

Otus_spilocephalus_spilocephalus_90 -----------------------------------------------------------------------------------------------------------------------------------------------------------------------------------------------------------------------------------------------------------------------------------------------------------------------------------------------------------------------------------------------------------------------------------------------------------------------------------------------------------------------------------------------------------------------------------------------------------------------------------------------------------------------------------------------------------------------------------------------------------------------------------------------------------------------------------------------------------------------------------------------------------------------------------------------------------------------------------------------------------------

Otus_spilocephalus_hambroecki_92 -----------------------------------------------------------------------------------------------------------------------------------------------------------------------------------------------------------------------------------------------------------------------------------------------------------------------------------------------------------------------------------------------------------------------------------------------------------------------------------------------------------------------------------------------------------------------------------------------------------------------------------------------------------------------------------------------------------------------------------------------------------------------------------------------------------------------------------------------------------------------------------------------------------------------------------------------------------------------------------------------------------------

_Otus_spilocephalus_latouchi_CHIMERA -----------------------------------------------------------------------------------------------------------------------------------------------------------------------------------------------------------------------------------------------------------------------------------------------------------------------------------------------------------------------------------------------------------------------------------------------------------------------------------------------------------------------------------------------------------------------------------------------------------------------------------------------------------------------------------------------------------------------------------------------------------------------------------------------------------------------------------------------------------------------------------------------------------------------------------------------------------------------------------------------------------------

Otus_hartlaubi_13 attatcttaacccaattattttggttaaacttgwtcagttgggaatggctaaagatgatattttatgggaagatctgatagagcgtgcagagtcagtagctgaaattaacaagattgatcatgctgcagcttgtctcagaagcagtgttatattgagtcttattgatgaaaaactaaaatctagggaccctagagctaaagaatttgctgcaaaatgtcaaaccatccctttccttccttttcttagcaaaccagcaggcttctcactgcattggaaaggcaatgattttcagcctgaagcaatgttttcagcaactgatcttttcacagctgatcatcaagatatagtttgcctaatacaaccaattcttaatgaaaattcccactcctttaaaggttgtggtgctttgtcattagctgtcaaagaatttttgggtttactgaagaaaccagctgtcaatttggtcataagtcagttagaagaagttgcaaagttatttgatgggatcacattatatcaagaaaatatcactaatgcatgttacaaatatctacatgaagcaatgttacagaatgaatcaaccaaagctatgataattgaacaactgctaaactgtagttttattctggttgagaatgtgtatgttgatccaacaaaagtgtcttttcatttgaattttgaagcagcaccctatctctatcagttgcctaataaatataaaaatagttttcgtgagctatttgaaagtgtgggtgtaagacaggcttttacagttgaagattttgctgtagttctggaattaataaatcaggaaagagggaccaaacaactaacagaagacaactttcagctttgcaggagaataattagtgaaggaatatggggcctca--------------------------------------------------------------------------------------

Otus_hartlaubi_15 attatcttaacccaattattttggttaaacttgwtcagttgggaatggctaaagatgatattttatgggaagatctgatagagcgtgcagagtcagtagctgaaattaacaagattgatcatgctgcagcttgtctcagaagcagtgttatattgagtcttattgatgaaaaactaaaatctagggaccctagagctaaagaatttgctgcaaaatgtcaaaccatccctttccttccttttcttagcaaaccagcaggcttctcactgcattggaaaggcaatgattttcagcctgaagcaatgttttcagcaactgatcttttcacagctgatcatcaagatatagtttgcctaatacaaccaattcttaatgaaaattcccactcctttaaaggttgtggtgctttgtcattagctgtcaaagaatttttgggtttactgaagaaaccagctgtcaatttggtcataagtcagttagaagaagttgcaaagttatttgatgggatcacattatatcaagaaaatatcactaatgcatgttacaaatatctacatgaagcaatgttacagaatgaatcaaccaaagctatgataattgaacaactgctaaactgtagttttattctggttgagaatgtgtatgttgatccaacaaaagtgtcttttcatttgaattttgaagcagcaccctatctctatcagttgcctaataaatataaaaatagttttcgtgagctatttgaaagtgtgggtgtaagacaggcttttacagttgaagattttgctgtagttctggaattaataaatcaggaaagagggaccaaacaactaacagaagacaactttcagctttgcaggagaataattagtgaaggaatatggggcctca--------------------------------------------------------------------------------------

Otus_hartlaubi_16 attatcttaacccaattattttggttaaacttgwtcagttgggaatggctaaagatgatattttatgggaagatctgatagagcgtgcagagtcagtagctgaaattaacaagattgatcatgctgcagcttgtctcagaagcagtgttatattgagtcttattgatgaaaaactaaaatctagggaccctagagctaaagaatttgctgcaaaatgtcaaaccatccctttccttccttttcttagcaaaccagcaggcttctcactgcattggaaaggcaatgattttcagcctgaagcaatgttttcagcaactgatcttttcacagctgatcatcaagatatagtttgcctaatacaaccaattcttaatgaaaattcccactcctttaaaggttgtggtgctttgtcattagctgtcaaagaatttttgggtttactgaagaaaccagctgtcaatttggtcataagtcagttagaagaagttgcaaagttatttgatgggatcacattatatcaagaaaatatcactaatgcatgttacaaatatctacatgaagcaatgttacagaatgaatcaaccaaagctatgataattgaacaactgctaaactgtagttttattctggttgagaatgtgtatgttgatccaacaaaagtgtcttttcatttgaattttgaagcagcaccctatctctatcagttgcctaataaatataaaaatagttttcgtgagctatttgaaagtgtgggtgtaagacaggcttttacagttgaagattttgctgtagttctggaattaataaatcaggaaagagggaccaaacaactaacagaagacaactttcagctttgcaggagaataattagtgaaggaatatggggcctca--------------------------------------------------------------------------------------

Otus_hartlaubi_14 ---------------------------------------------------------------------------------agcgtgcagagtcagtagctgaaattaacaagattgatcatgctgcagcttgtctcagaagcagtgttatattgagtcttattgatgaaaaactaaaatctagggaccctagagctaaagaatttgctgcaaaatgtcaaaccatccctttccttccttttcttagcaaaccagcaggcttctcactgcattggaaaggcaatgattttcagcctgaagcaatgttttcagcaactgatcttttcacagctgatcatcaagatatagtttgcctaatacaaccaattcttaatgaaaattcccactcctttaaaggttgtggtgctttgtcattagctgtcaaagaatttttgggtttactgaagaaaccagctgtcaatttggtcataagtcagttagaagaagttgcaaagttatttgatgggatcacattatatcaagaaaatatcactaatgcatgttacaaatatctacatgaagcaatgttacagaatgaatcaaccaaagctatgataattgaacaactgctaaactgtagttttattctggttgagaatgtgtatgttgatccaacaaaagtgtcttttcatttgaattttgaagcagcaccctatctctatcagttgcctaataaatataaaaatagttttcgtgagctatttgaaagtgtgggtgtaagacaggcttttacagttgaagattttgctgtagttctggaattaataaatcaggaaagagggaccaaacaactaacagaagacaactttcagctttgcaggagaataattagtgaaggaatatggggcctca--------------------------------------------------------------------------------------

Otus_feae_45 attatcttaacccaattattttggttaaacttgwtcagttgggaatggctaaagatgatattttatgggaagatctgatagagcgtgcagagtcagtagctgaaattaacaagattgatcatgctgcagcttgtctcagaagcagtgttatattgagtcttattgatgaaaaactaaaatctagggaccctagagctaaagaatttgctgcaaaatgtcaaaccatccctttccttccttttcttagcaaaccagcaggcttctcactgcattggaaaggcaatgattttcagcctgaagcaatgttttcagcaactgatcttttcacagctgatcatcaagatatagtttgcctaatacaaccaattcttaatgaaaattcccactcctttaaaggttgtggtgctttgtcattagctgtcaaagaatttttgggtttactgaagaaaccagctgtcaatttggtcataagtcagttagaagaagttgcaaagttatttgatgggatcacattatatcaagaaaatatcactaatgcatgttacaaatatctacatgaagcaatgttacagaatgaatcaaccaaagctatgataattgaacaactgctaaactgtagttttattctggttgagaatgtgtatgttgatccaacaaaagtgtcttttcatttgaattttgaagcagcaccctatctctatcagttgcctaataaatataaaaatagttttcgtgagctatttgaaagtgtgggtgtaagacaggcttttacagttgaagattttgctgtagttctggaattaataaatcaggaaagagggaccaaacaactaacagaagacaactttcagctttgcaggagaataattagtgaaggaatatggggcctca--------------------------------------------------------------------------------------

Otus_senegalensis_17 -----------------------------------------------------------------------------------------------------------------------------------------------------------------------------------------------------------------------------------------------------------------------------------------------------------------------------------------------------------------------------------------------------------------------------------------------------------------------------------------------------------------------------------------------------------------------------------------------------------------------------------------------------------------------------------------------------------------------------------------------------------------------------------------------------------------------------------------------------------------------------------------------------------------------------------------------------------------------------------------------------------------

Otus_pamelae_74 -----------------------------------------------------------------------------------------------------------------------------------------------------------------------------------------------------------------------------------------------------------------------------------------------------------------------------------------------------------------------------------------------------------------------------------------------------------------------------------------------------------------------------------------------------------------------------------------------------------------------------------------------------------------------------------------------------------------------------------------------------------------------------------------------------------------------------------------------------------------------------------------------------------------------------------------------------------------------------------------------------------------

Otus_sp_nov_1 attatcttaacccaattattttggttaaacttgttcagttrggaatggctaaagatgatattttatgggaagatctgataragcgtgcagagtcagtagctgaaattaacaagattgatcatgctgcagcttgtctcagaagcagtgttatattgagtcttattgatgaaaaactaaaatctagggaccctagagctaaagaatttgctgcaaaatgtcaaaccatccctttccttccttttcttagcaaaccagcaggcttctcactgcattggaaaggcaatgattttcagcctgaagcaatgttttcagcaactgatcttttcacagctgatcatcaagatatagtttgcctaatacaaccaattcttaatgaaaattcccactcctttaaaggttgtggtgctttgtcattagctgtcaaagaatttttgggtttactgaagaaaccagctgtcaatttggtcataagtcagttagaagaagttgcaaagttatttgatgggatcacattatatcaagaaaatatcactaatgcatgttacaaatatctacatgaagcaatgttacagaatgaatcaaccaaagctatgataattgaacaactgctaaactgtagttttattctggttgagaatgtgtatgttgatccaacaaaagtgtcttttcatttgaattttgaagcagcaccctatctctatcagttgcctaataaatataaaaatagttttcgtgagctatttgaaagtgtgggtgtaagacaggcttttacagttgaagattttgctgtagttctggaattaataaatcaggaaagagggaccaaacaactaacagaagacaactttcagctttgcaggagaataattagtgaaggaatatggggcctca--------------------------------------------------------------------------------------

Otus_sp_nov_2 attatcttaacccaattattttggttaaacttgttcagttgggaatggctaaagatgatattttatgggaagatctgatagagcgtgcagagtcagtagctgaaattaacaagattgatcatgctgcagcttgtctcagaagcagtgttatattgagtcttattgatgaaaaactaaaatctagggaccctagagctaaagaatttgctgcaaaatgtcaaaccatccctttccttccttttcttagcaaaccagcaggcttctcactgcattggaaaggcaatgattttcagcctgaagcaatgttttcagcaactgatcttttcacagctgatcatcaagatatagtttgcctaatacaaccaattcttaatgaaaattcccactcctttaaaggttgtggtgctttgtcattagctgtcaaagaatttttgggtttactgaagaaaccagctgtcaatttggtcataagtcagttagaagaagttgcaaagttatttgatgggatcacattatatcaagaaaatatcactaatgcatgttacaaatatctacatgaagcaatgttacagaatgaatcaaccaaagctatgataattgaacaactgctaaactgtagttttattctggttgagaatgtgtatgttgatccaacaaaagtgtcttttcatttgaattttgaagcagcaccctatctctatcagttgcctaataaatataaaaatagttttcgtgagctatttgaaagtgtgggtgtaagacaggcttttacagttgaagattttgctgtagttctggaattaataaatcaggaaagagggaccaaacaactaacagaagacaactttcagctttgcaggagaataattagtgaaggaatatggggcctca--------------------------------------------------------------------------------------

Otus_sp_nov_3 attatcttaacccaattattttggttaaacttgttcagttgggaatggctaaagatgatattttatgggaagatctgatagagcgtgcagagtcagtagctgaaattaacaagattgatcatgctgcagcttgtctcagaagcagtgttatattgagtcttattgatgaaaaactaaaatctagggaccctagagctaaagaatttgctgcaaaatgtcaaaccatccctttccttccttttcttagcaaaccagcaggcttctcactgcattggaaaggcaatgattttcagcctgaagcaatgttttcagcaactgatcttttcacagctgatcatcaagatatagtttgcctaatacaaccaattcttaatgaaaattcccactcctttaaaggttgtggtgctttgtcattagctgtcaaagaatttttgggtttactgaagaaaccagctgtcaatttggtcataagtcagttagaagaagttgcaaagttatttgatgggatcacattatatcaagaaaatatcactaatgcatgttacaaatatctacatgaagcaatgttacagaatgaatcaaccaaagctatgataattgaacaactgctaaactgtagttttattctggttgagaatgtgtatgttgatccaacaaaagtgtcttttcatttgaattttgaagcagcaccctatctctatcagttgcctaataaatataaaaatagttttcgtgagctatttgaaagtgtgggtgtaagacaggcttttacagttgaagattttgctgtagttctggaattaataaatcaggaaagagggaccaaacaactaacagaagacaactttcagctttgcaggagaataattagtgaaggaatatggggcctcattagagagaaaaagcaggaattttgtgagaaaaagtatggtgagattttgctacccgatactcgtcttgcacttctgcctgcaaaa

Otus_sp_nov_4 attatcttaacccaattattttggttaaacttgttcagttgggaatggctaaagatgatattttatgggaagatctgatagagcgtgcagagtcagtagctgaaattaacaagattgatcatgctgcagcttgtctcagaagcagtgttatattgagtcttattgatgaaaaactaaaatctagggaccctagagctaaagaatttgctgcaaaatgtcaaaccatccctttccttccttttcttagcaaaccagcaggcttctcactgcattggaaaggcaatgattttcagcctgaagcaatgttttcagcaactgatcttttcacagctgatcatcaagatatagtttgcctaatacaaccaattcttaatgaaaattcccactcctttaaaggttgtggtgctttgtcattagctgtcaaagaatttttgggtttactgaagaaaccagctgtcaatttggtcataagtcagttagaagaagttgcaaagttatttgatgggatcacattatatcaagaaaatatcactaatgcatgttacaaatatctacatgaagcaatgttacagaatgaatcaaccaaagctatgataattgaacaactgctaaactgtagttttattctggttgagaatgtgtatgttgatccaacaaaagtgtcttttcatttgaattttgaagcagcaccctatctctatcagttgcctaataaatataaaaatagttttcgtgagctatttgaaagtgtgggtgtaagacaggcttttacagttgaagattttgctgtagttctggaattaataaatcaggaaagagggaccaaacaactaacagaagacaactttcagctttgcaggagaataattagtgaaggaatatggggcctcattagagagaaaaagcaggaattttgtgagaaaaagtatggtgagattttgctacccgatactcgtcttgcacttctgcctgcaaaa

Otus_scops_5 -----------------------------------------------------------------------------------------------------------------------------------------------------------------------------------------------------------------------------------------------------------------------------------------------------------------------------------------------------------------------------------------------------------------------------------------------------------------------------------------------------------------------------------------------------------------------------------------------------------------------------------------------------------------------------------------------------------------------------------------------------------------------------------------------------------------------------------------------------------------------------------------------------------------------------------------------------------------------------------------------------------------

_Otus_scops_JF5337_F -----------------------------------------------------------------------------------------------------------------------------------------------------------------------------------------------------------------------------------------------------------------------------------------------------------------------------------------------------------------------------------------------------------------------------------------------------------------------------------------------------------------------------------------------------------------------------------------------------------------------------------------------------------------------------------------------------------------------------------------------------------------------------------------------------------------------------------------------------------------------------------------------------------------------------------------------------------------------------------------------------------------

Otus_pembaensis_24 -----------------------------------------------------------------------------------------------------------------------------------------------------------------------------------------------------------------------------------------------------------------------------------------------------------------------------------------------------------------------------------------------------------------------------------------------------------------------------------------------------------------------------------------------------------------------------------------------------------------------------------------------------------------------------------------------------------------------------------------------------------------------------------------------------------------------------------------------------------------------------------------------------------------------------------------------------------------------------------------------------------------

Otus_pembaensis_25 -----------------------------------------------------------------------------------------------------------------------------------------------------------------------------------------------------------------------------------------------------------------------------------------------------------------------------------------------------------------------------------------------------------------------------------------------------------------------------------------------------------------------------------------------------------------------------------------------------------------------------------------------------------------------------------------------------------------------------------------------------------------------------------------------------------------------------------------------------------------------------------------------------------------------------------------------------------------------------------------------------------------

_Otus_longicornis_CHIMERA -----------------------------------------------------------------------------------------------------------------------------------------------------------------------------------------------------------------------------------------------------------------------------------------------------------------------------------------------------------------------------------------------------------------------------------------------------------------------------------------------------------------------------------------------------------------------------------------------------------------------------------------------------------------------------------------------------------------------------------------------------------------------------------------------------------------------------------------------------------------------------------------------------------------------------------------------------------------------------------------------------------------

Otus_mirus_CHIMERA -----------------------------------------------------------------------------------------------------------------------------------------------------------------------------------------------------------------------------------------------------------------------------------------------------------------------------------------------------------------------------------------------------------------------------------------------------------------------------------------------------------------------------------------------------------------------------------------------------------------------------------------------------------------------------------------------------------------------------------------------------------------------------------------------------------------------------------------------------------------------------------------------------------------------------------------------------------------------------------------------------------------

Otus_mayottensis_60 -----------------------------------------------------------------------------------------------------------------------------------------------------------------------------------------------------------------------------------------------------------------------------------------------------------------------------------------------------------------------------------------------------------------------------------------------------------------------------------------------------------------------------------------------------------------------------------------------------------------------------------------------------------------------------------------------------------------------------------------------------------------------------------------------------------------------------------------------------------------------------------------------------------------------------------------------------------------------------------------------------------------

Otus_capnodes_40 -----------------------------------------------------------------------------------------------------------------------------------------------------------------------------------------------------------------------------------------------------------------------------------------------------------------------------------------------------------------------------------------------------------------------------------------------------------------------------------------------------------------------------------------------------------------------------------------------------------------------------------------------------------------------------------------------------------------------------------------------------------------------------------------------------------------------------------------------------------------------------------------------------------------------------------------------------------------------------------------------------------------

_Otus_madagascariensis_CHIMERA -----------------------------------------------------------------------------------------------------------------------------------------------------------------------------------------------------------------------------------------------------------------------------------------------------------------------------------------------------------------------------------------------------------------------------------------------------------------------------------------------------------------------------------------------------------------------------------------------------------------------------------------------------------------------------------------------------------------------------------------------------------------------------------------------------------------------------------------------------------------------------------------------------------------------------------------------------------------------------------------------------------------

Otus_pauliani_76 -----------------------------------------------------------------------------------------------------------------------------------------------------------------------------------------------------------------------------------------------------------------------------------------------------------------------------------------------------------------------------------------------------------------------------------------------------------------------------------------------------------------------------------------------------------------------------------------------------------------------------------------------------------------------------------------------------------------------------------------------------------------------------------------------------------------------------------------------------------------------------------------------------------------------------------------------------------------------------------------------------------------

Otus_insularis_47 -----------------------------------------------------------------------------------------------------------------------------------------------------------------------------------------------------------------------------------------------------------------------------------------------------------------------------------------------------------------------------------------------------------------------------------------------------------------------------------------------------------------------------------------------------------------------------------------------------------------------------------------------------------------------------------------------------------------------------------------------------------------------------------------------------------------------------------------------------------------------------------------------------------------------------------------------------------------------------------------------------------------

Otus_socotranus_89 -----------------------------------------------------------------------------------------------------------------------------------------------------------------------------------------------------------------------------------------------------------------------------------------------------------------------------------------------------------------------------------------------------------------------------------------------------------------------------------------------------------------------------------------------------------------------------------------------------------------------------------------------------------------------------------------------------------------------------------------------------------------------------------------------------------------------------------------------------------------------------------------------------------------------------------------------------------------------------------------------------------------

_Otus_sunia_CHIMERA -----------------------------------------------------------------------------------------------------------------------------------------------------------------------------------------------------------------------------------------------------------------------------------------------------------------------------------------------------------------------------------------------------------------------------------------------------------------------------------------------------------------------------------------------------------------------------------------------------------------------------------------------------------------------------------------------------------------------------------------------------------------------------------------------------------------------------------------------------------------------------------------------------------------------------------------------------------------------------------------------------------------

Otus_moheliensis_CHIMERA attatcttaacccaattattttggttaaacttgttcagttgggaatggctaaagatgatattttatgggaagatctgatagagcgtgcagagtcagtagctgaaattaacaagattgatcatgctgcagcttgtctcagaagcagtgttatattgagtcttattgatgaaaaactaaaatctagggaccctagagctaaagaatttgctgcaaaatgtcaaaccatccctttccttccttttcttagcaaaccagcaggcttctcactgcattggaaaggcaatgattttcagcctgaagcaatgttttcagcaactgatcttttcacagctgatcatcaagatatagtttgcctaatacaaccaattcttaatgaaaattcccactcctttaaaggttgtgrtgctttgtcactagctgtcaaagaatttttgggtttactgaagaaaccagctgtcaatttggtcataagtcagttagaagaagttgcaaagttatttgatgggatcacattatatcaagaaaatatcactaatgcatgttacaaatatctacatgaagcaatgttacagaatgaatcaaccaaagctatgataattgaacaactgctaaactgtagttttattctggttgagaatgtgtatgttgatccaacaaaagtgtcttttcatttgaattttgaagcagcaccctatctctatcagttgcctaataaatataaaaatagttttcgtgagctatttgaaagtgtgggtgtaagacaggcttttacagttgaagattttgctgtagttctggaattaataaatcaggaaagagggaccaaacaactaacagaagacaactttcagctttgcaggagaataattagtgaaggaatatggggcctca--------------------------------------------------------------------------------------

Otus_brucei_CHIMERA -----------------------------------------------------------------------------------------------------------------------------------------------------------------------------------------------------------------------------------------------------------------------------------------------------------------------------------------------------------------------------------------------------------------------------------------------------------------------------------------------------------------------------------------------------------------------------------------------------------------------------------------------------------------------------------------------------------------------------------------------------------------------------------------------------------------------------------------------------------------------------------------------------------------------------------------------------------------------------------------------------------------

Otus_icterorhynchus_holerythrus_27 -----------------------------------------------------------------------------------------------------------------------------------------------------------------------------------------------------------------------------------------------------------------------------------------------------------------------------------------------------------------------------------------------------------------------------------------------------------------------------------------------------------------------------------------------------------------------------------------------------------------------------------------------------------------------------------------------------------------------------------------------------------------------------------------------------------------------------------------------------------------------------------------------------------------------------------------------------------------------------------------------------------------

Otus_icterorhynchus_CHIMERA -----------------------------------------------------------------------------------------------------------------------------------------------------------------------------------------------------------------------------------------------------------------------------------------------------------------------------------------------------------------------------------------------------------------------------------------------------------------------------------------------------------------------------------------------------------------------------------------------------------------------------------------------------------------------------------------------------------------------------------------------------------------------------------------------------------------------------------------------------------------------------------------------------------------------------------------------------------------------------------------------------------------

Otus_ireneae_49 -----------------------------------------------------------------------------------------------------------------------------------------------------------------------------------------------------------------------------------------------------------------------------------------------------------------------------------------------------------------------------------------------------------------------------------------------------------------------------------------------------------------------------------------------------------------------------------------------------------------------------------------------------------------------------------------------------------------------------------------------------------------------------------------------------------------------------------------------------------------------------------------------------------------------------------------------------------------------------------------------------------------

Otus_rutilus_78 -----------------------------------------------------------------------------------------------------------------------------------------------------------------------------------------------------------------------------------------------------------------------------------------------------------------------------------------------------------------------------------------------------------------------------------------------------------------------------------------------------------------------------------------------------------------------------------------------------------------------------------------------------------------------------------------------------------------------------------------------------------------------------------------------------------------------------------------------------------------------------------------------------------------------------------------------------------------------------------------------------------------

Otus_scops_scops_8 ----------------tattttggttaaacttgttcagttgggaatggctaaagatgatattttatgggaagatctgataragcgtgcagagtcagtagctgaaattaacaagattgatcatgctgcagcttgtctcagaagcagtgttatattgagtcttattgatgaaaaactaaaatctagggaccctagagctaaagaatttgctgcaaaatgtcaaaccatccctttccttccttttcttagcaaaccagcaggcttctcactgcattggaaaggcaatgattttcagcctgaagcaatgttttcagcaactgatcttttcacagctgatcatcaagatatagtttgcctaatacaaccaattcttaatgaaaattcccactcctttaaaggttgtggtgctttgtcattagctgtcaaagaatttttgggtttactgaagaaaccagctgtcaatttggtcataagtcagttagaagaagttgcaaagttatttgatgggatcacattatatcaagaaaatatcactaatgcatgttacaaatatctacatgaagcaatgttacagaatgaatcaaccaaagctatgataattgaacaactgctaaactgtagttttattctggttgagaatgtgtatgttgatccaacaaaagtgtcttttcatttgaattttgaagcagcaccctatctctatcagttgcctaataaatataaaaatagttttcgtgagctatttgaaagtgtgggtgtaagacaggcttttacagttgaagattttgctgtagttctggaattaataaatcaggaaagagggaccaaacaactaacagaagacaactttcagctttgcaggagaataattagtgaaggaatatggggcctca--------------------------------------------------------------------------------------

Otus_scops_scops_9 attatcttaacccaattattttggttaaacttgttcagttgggaatggctaaagatgatattttatgggaagatctgatagagcgtgcagagtcagtagctgaaattaacaagattgatcatgctgcagcttgtctcagaagcagtgttatattgagtcttattgatgaaaaactaaaatctagggaccctagagctaaagaatttgctgcaaaatgtcaaaccatccctttccttccttttcttagcaaaccagcaggcttctcactgcattggaaaggcaatgattttcagcctgaagcaatgttttcagcaactgatcttttcacagctgatcatcaagatatagtttgcctaatacaaccaattcttaatgaaaattcccactcctttaaaggttgtggtgctttgtcattagctgtcaaagaatttttgggtttactgaagaaaccagctgtcaatttggtcataagtcagttagaagaagttgcaaarttatttgatgggatcacattatatcaagaaaatatcactaatgcatgttacaaatatctacatgaagcaatgttacagartgaatcaaccaaagctatgatamttgaacaactgctaaactgtagttttattctggttgagaatgtgtatgttgatccaacaaaagtgtcttttcatttgaattttgaagcagcaccctatctctatcagttgcctaataaatataaaaatagttttcgtgagctatttgaaagtgtgggtgtaagacaggcttttacagttgaagattttgctgtagttctggaattaataaatcaggaaagagggaccaaacaactaacagaagacaactttcagctttgcaggagaataattagtgaaggaatatggggcctca--------------------------------------------------------------------------------------

Otus_spilocephalus_luciae_94 -----------------------------------------------------------------------------------------------------------------------------------------------------------------------------------------------------------------------------------------------------------------------------------------------------------------------------------------------------------------------------------------------------------------------------------------------------------------------------------------------------------------------------------------------------------------------------------------------------------------------------------------------------------------------------------------------------------------------------------------------------------------------------------------------------------------------------------------------------------------------------------------------------------------------------------------------------------------------------------------------------------------

Otus_silvicola_87 -----------------------------------------------------------------------------------------------------------------------------------------------------------------------------------------------------------------------------------------------------------------------------------------------------------------------------------------------------------------------------------------------------------------------------------------------------------------------------------------------------------------------------------------------------------------------------------------------------------------------------------------------------------------------------------------------------------------------------------------------------------------------------------------------------------------------------------------------------------------------------------------------------------------------------------------------------------------------------------------------------------------

_Otus_cyprius_CHIMERA -----------------------------------------------------------------------------------------------------------------------------------------------------------------------------------------------------------------------------------------------------------------------------------------------------------------------------------------------------------------------------------------------------------------------------------------------------------------------------------------------------------------------------------------------------------------------------------------------------------------------------------------------------------------------------------------------------------------------------------------------------------------------------------------------------------------------------------------------------------------------------------------------------------------------------------------------------------------------------------------------------------------

Otus_elegans_CHIMERA -----------------------------------------------------------------------------------------------------------------------------------------------------------------------------------------------------------------------------------------------------------------------------------------------------------------------------------------------------------------------------------------------------------------------------------------------------------------------------------------------------------------------------------------------------------------------------------------------------------------------------------------------------------------------------------------------------------------------------------------------------------------------------------------------------------------------------------------------------------------------------------------------------------------------------------------------------------------------------------------------------------------

[marker: 16S; length: 1626; no. of seqs: 51; new coordinates: 7295-8920)]

Bubo_bubo_111 ------------------------------------------------------------------------------------------------------------------------------------------------------------------------------------------------------------------------------------------------------------------------------------------------------------------------------------------------------------------------------------------------------------------------------------------------------------------------------------------------------------------------------------------------------------------------------------------------------------------------------------------------------------------------------------------------------------------------------------------------------------------------------------------------------------------------------------------------------------------------------------------------------------------------------------------------------------------------------------------------------------------------------------------------------------------------------------------------------------------------------------------------------------------------------------------------------------------------------------------------------------------------------------------------------------------------------------------------------------------------------------------------------------------------------------------------------------------------------------------------------------------------------------------------------------------------------------------------------------------------------------------------------------------------------------------

Strix_aluco_129 ------------------------------------------------------------------------------------------------------------------------------------------------------------------------------------------------------------------------------------------------------------------------------------------------------------------------------------------------------------------------------------------------------------------------------------------------------------------------------------------------------------------------------------------------------------------------------------------------------------------------------------------------------------------------------------------------------------------------------------------------------------------------------------------------------------------------------------------------------------------------------------------------------------------------------------------------------------------------------------------------------------------------------------------------------------------------------------------------------------------------------------------------------------------------------------------------------------------------------------------------------------------------------------------------------------------------------------------------------------------------------------------------------------------------------------------------------------------------------------------------------------------------------------------------------------------------------------------------------------------------------------------------------------------------------------------

Otus_bakkamoena_marathae_35 ------------------------------------------------------------------------------------------------------------------------------------------------------------------------------------------------------------------------------------------------------------------------------------------------------------------------------------------------------------------------------------------------------------------------------------------------------------------------------------------------------------------------------------------------------------------------------------------------------------------------------------------------------------------------------------------------------------------------------------------------------------------------------------------------------------------------------------------------------------------------------------------------------------------------------------------------------------------------------------------------------------------------------------------------------------------------------------------------------------------------------------------------------------------------------------------------------------------------------------------------------------------------------------------------------------------------------------------------------------------------------------------------------------------------------------------------------------------------------------------------------------------------------------------------------------------------------------------------------------------------------------------------------------------------------------------

_Otus_lempiji_CHIMERA agcccccctctagccccaccca----acacccattaaaaataccctcactga-gcctaatcaaaacatttttctccaaacaagccctagtataggtgatagaaaaggcaacac----cacccatgaggcgcaatagaga-ctacgtaccgtaagggaagaatgaaatagtaatgaaacccaaagcaatacaaagcaaagacaaacccttgtacctcttgcatcatggtttagccagaacaaccaagcaaaacgaacttaagcttgcccccccgaaacccaagcgagctactcgcgagcagctacgtacctgagcgaacccatctctgtggcaaaagagcgggatgacttgctagtagcggtgaaaagcctaccgagctgggtgatagctggttgcctgtgaaatgaatctaagttcccccttaagcccctccccaacagacacacaatctgtcatgagaagcctaagagcaatttaaagggggtacagcccctttaaaacagggtacaacctcctccagaggataataaccaagtataata-atagtaggccctcaagcagccaccaacaaagagtgcgtcaaagctcaccaccctaaaaatccaacaaatactgcgactccctccctaccaacaggctaatctatacatatagaagaatcaatgctagaatgagtaatctggg-----tccccccctcttaagcgcaaacttaccccaaacaattaacagaaaaactaaatacaaaaatca--acaagaactatatatcaacccaccctgttaacccaacccaggagcgcctactagaaagattaaaatctgtagaaggaactaggcaaacc---aaaggcccgactgtttaccaaaaacatagccttcagcgtaccaagtattgaaggtgatgcctgcccagtgacaccatgtttaacggccgcggtatcctaaccgtgcgaaggtagcgcaatcaattgtcccataaatcgagacctgtatgaatggctaaacgaggtcttaactgtcccctacagacaatcgatgaaattgatccccccgtgcaaaagcgaggatacccccataagacgagaagaccctgtggaacttttaaaatcaacggccaacacgtacaaccccccctccccctaaggggccactg----------acattaactgcactggccgc-aatttttcggttggggcgaccttggagaaaaacagaccctccaaagcaaagaccacacctcttgaccaagaacaacacctcaacgtgctaatagcaacctgacccaat-accaattgagcaatgaaccaagctaccccagggataacagcgcaatcccctccaagagcccctatcgacgaggaggtttacgacctcgatgttggatcaggacatcctaatggtgcagccgctattaagggttcgtttgttcaacgattaacagtcctacgtgatctgagttcagaccggagcaatccaggtcggtttctatctatgacctagaccccttcctagtacgaaaggaccggaagagcggggccaatacttcacagcacgcccccaaaaaaacaatgaacacaactcaatta-caaatta-cctcattagccctagacaagggca

Otus_lettia_lettia_CHIMERA ------------------------------------------------------------------------------------------------------------------------------------------------------------------------------------------------------------------------------------------------------------------------------------------------------------------------------------------------------------------------------------------------------------------------------------------------------------------------------------------------------------------------------------------------------------------------------------------------------------------------------------------------------------------------------------------------------------------------------------------------------------------------------------------------------------------------------------------------------------------------------------------------------------------------------------------------------------------------------------------------------------------------------------------------------------------------------------------------------------------------------------------------------------------------------------------------------------------------------------------------------------------------------------------------------------------------------------------------------------------------------------------------------------------------------------------------------------------------------------------------------------------------------------------------------------------------------------------------------------------------------------------------------------------------------------------

Otus_megalotis_CHIMERA ------------------------------------------------------------------------------------------------------------------------------------------------------------------------------------------------------------------------------------------------------------------------------------------------------------------------------------------------------------------------------------------------------------------------------------------------------------------------------------------------------------------------------------------------------------------------------------------------------------------------------------------------------------------------------------------------------------------------------------------------------------------------------------------------------------------------------------------------------------------------------------------------------------------------------------------------------------------------------------------------------------------------------------------------------------------------------------------------------------------------------------------------------------------------------------------------------------------------------------------------------------------------------------------------------------------------------------------------------------------------------------------------------------------------------------------------------------------------------------------------------------------------------------------------------------------------------------------------------------------------------------------------------------------------------------------

Otus_nigrorum_CHIMERA ------------------------------------------------------------------------------------------------------------------------------------------------------------------------------------------------------------------------------------------------------------------------------------------------------------------------------------------------------------------------------------------------------------------------------------------------------------------------------------------------------------------------------------------------------------------------------------------------------------------------------------------------------------------------------------------------------------------------------------------------------------------------------------------------------------------------------------------------------------------------------------------------------------------------------------------------------------------------------------------------------------------------------------------------------------------------------------------------------------------------------------------------------------------------------------------------------------------------------------------------------------------------------------------------------------------------------------------------------------------------------------------------------------------------------------------------------------------------------------------------------------------------------------------------------------------------------------------------------------------------------------------------------------------------------------------

Otus_everetti_CHIMERA ------------------------------------------------------------------------------------------------------------------------------------------------------------------------------------------------------------------------------------------------------------------------------------------------------------------------------------------------------------------------------------------------------------------------------------------------------------------------------------------------------------------------------------------------------------------------------------------------------------------------------------------------------------------------------------------------------------------------------------------------------------------------------------------------------------------------------------------------------------------------------------------------------------------------------------------------------------------------------------------------------------------------------------------------------------------------------------------------------------------------------------------------------------------------------------------------------------------------------------------------------------------------------------------------------------------------------------------------------------------------------------------------------------------------------------------------------------------------------------------------------------------------------------------------------------------------------------------------------------------------------------------------------------------------------------------

Otus_semitorques_CHIMERA ------------------------------------------------------------------------------------------------------------------------------------------------------------------------------------------------------------------------------------------------------------------------------------------------------------------------------------------------------------------------------------------------------------------------------------------------------------------------------------------------------------------------------------------------------------------------------------------------------------------------------------------------------------------------------------------------------------------------------------------------------------------------------------------------------------------------------------------------------------------------------------------------------------------------------------------------------------------------------------------------------------------------------------------------------------------------------------------------------------------------------------------------------------------------------------------------------------------------------------------------------------------------------------------------------------------------------------------------------------------------------------------------------------------------------------------------------------------------------------------------------------------------------------------------------------------------------------------------------------------------------------------------------------------------------------------
[truncated: 277,339 more chars]
