# Supplementary material for: ﻿A new species of scops-owl (Aves, Strigiformes, Strigidae, Otus) from Príncipe Island (Gulf of Guinea, Africa) and novel insights into the systematic affinities within Otus
Source: Zookeys. 2022 Oct 30;1126:1–54. doi: 10.3897/zookeys.1126.87635 (PMC9836643; doi:10.3897/zookeys.1126.87635)
Supplement: Supplementary material 3 — Figure S1. Topography Owls [file zookeys-1126-001_article-87635__-s003.pdf]

Suppl. material 3

TOPOGRAPHY OF A SCOPS-OWL

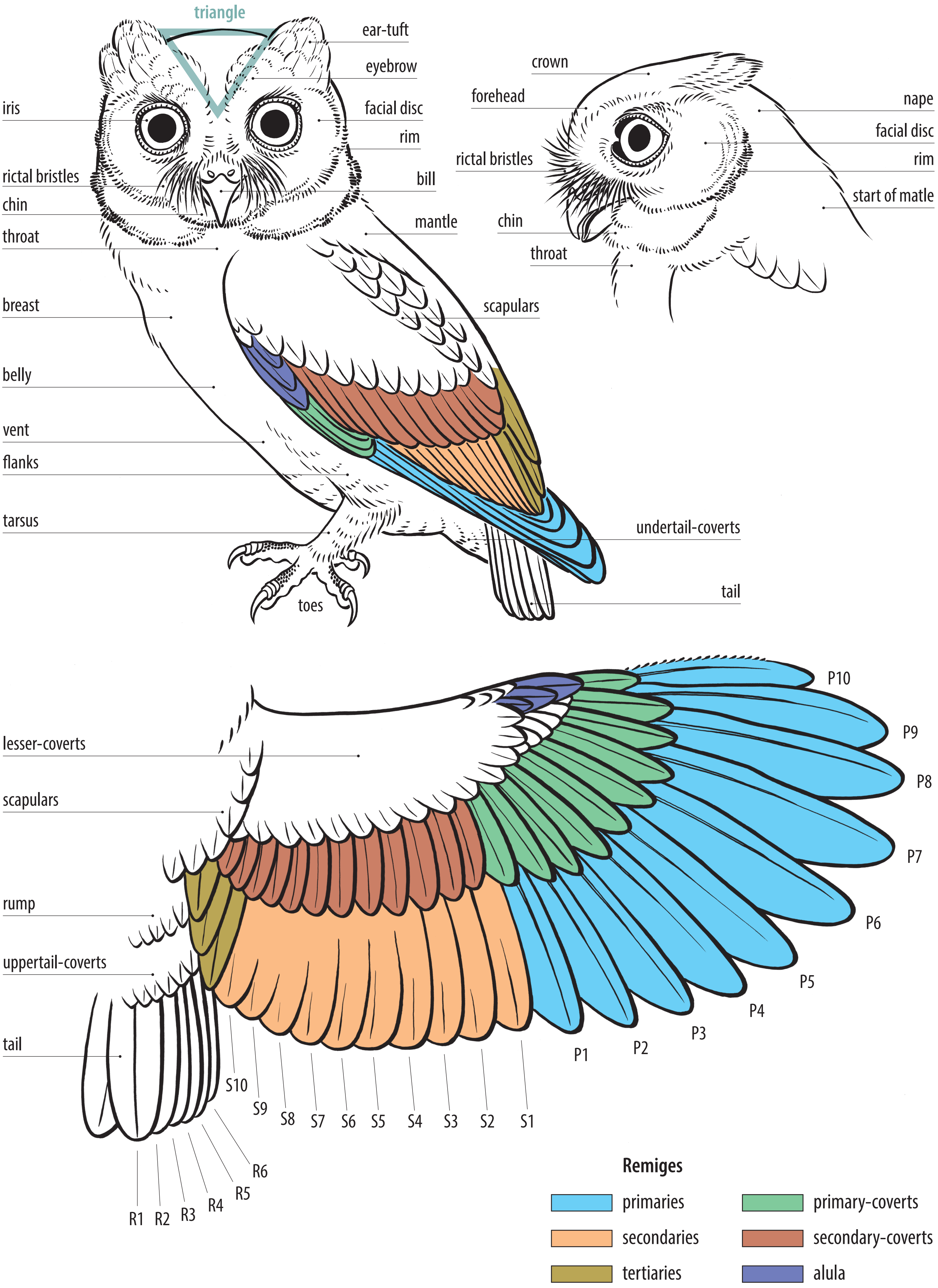

Figure S1. Topography of owls, with the terms used for the description and diagnosis of the Principe Scops-Owl *Otus bikegila* sp. nov. Illustration by MNC.
