# Supplementary material for: ﻿A new species of scops-owl (Aves, Strigiformes, Strigidae, Otus) from Príncipe Island (Gulf of Guinea, Africa) and novel insights into the systematic affinities within Otus
Source: Zookeys. 2022 Oct 30;1126:1–54. doi: 10.3897/zookeys.1126.87635 (PMC9836643; doi:10.3897/zookeys.1126.87635)
Supplement: Supplementary material 4 — Figure S2. Song oscillograms and spectrograms [file zookeys-1126-001_article-87635__-s004.pdf]

Suppl. material 4

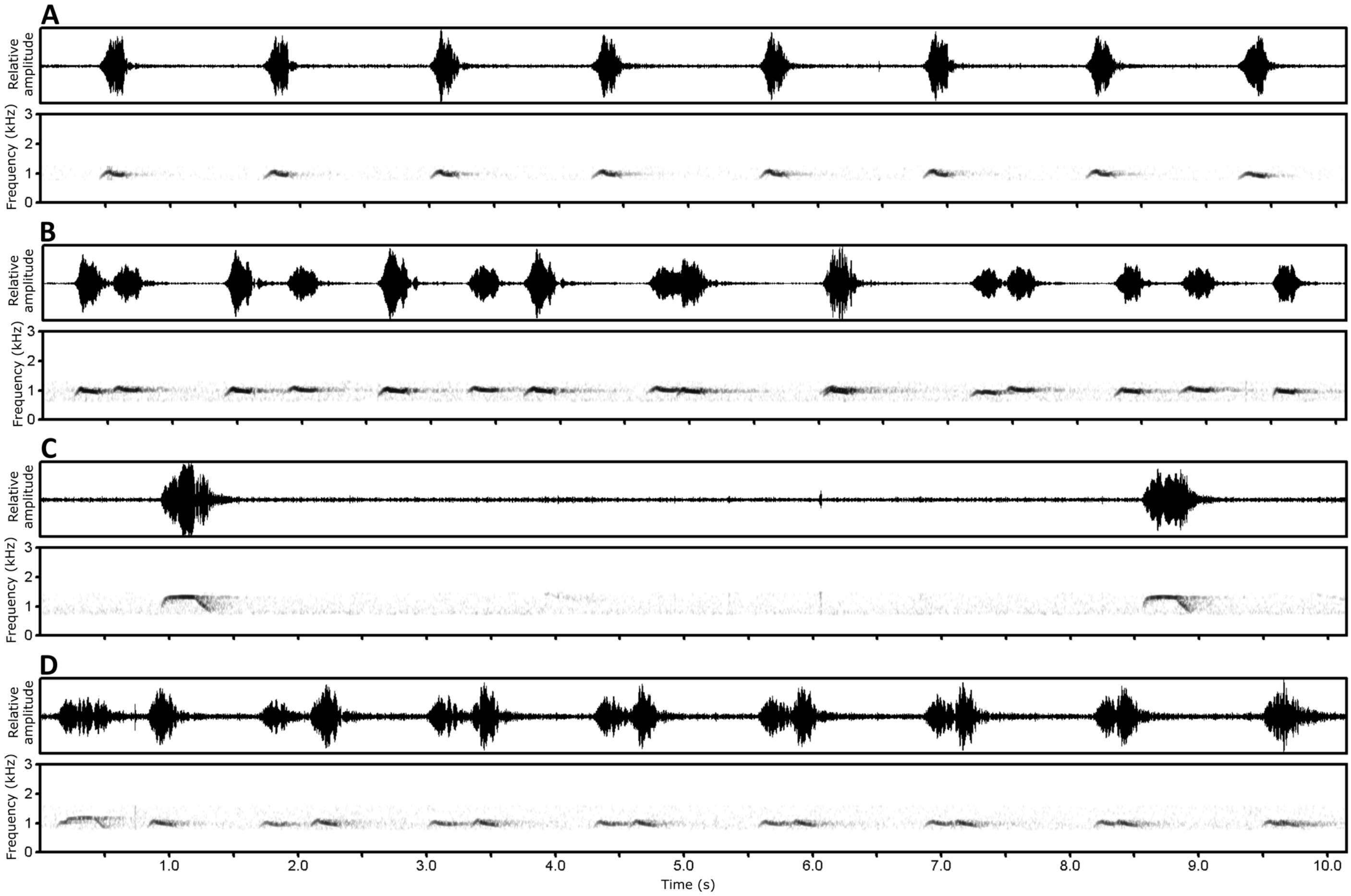

Figure S2. Oscillograms and spectrograms of 10s sections of the call of *O. bikegila* sp. nov.: **A** primary call of an individual, recorded on January 20, 2019, XC619448 **B** two different individuals duetting, recorded on July 28, 2018, XC619439 **C** cat-like "kee-a-u" call of one individual, recorded on January 15, 2019, XC619443 **D** two different individuals duetting, in which one emits a cat-like "kee-a-u" call, recorded on January 20, 2019, XC619448. Codes from Xeno-canto.org database.
