# Supplementary material for: ﻿A new species of scops-owl (Aves, Strigiformes, Strigidae, Otus) from Príncipe Island (Gulf of Guinea, Africa) and novel insights into the systematic affinities within Otus
Source: Zookeys. 2022 Oct 30;1126:1–54. doi: 10.3897/zookeys.1126.87635 (PMC9836643; doi:10.3897/zookeys.1126.87635)
Supplement: Supplementary material 5 — Figure S3. Bikegila [file zookeys-1126-001_article-87635__-s005.pdf]

Suppl. material 5

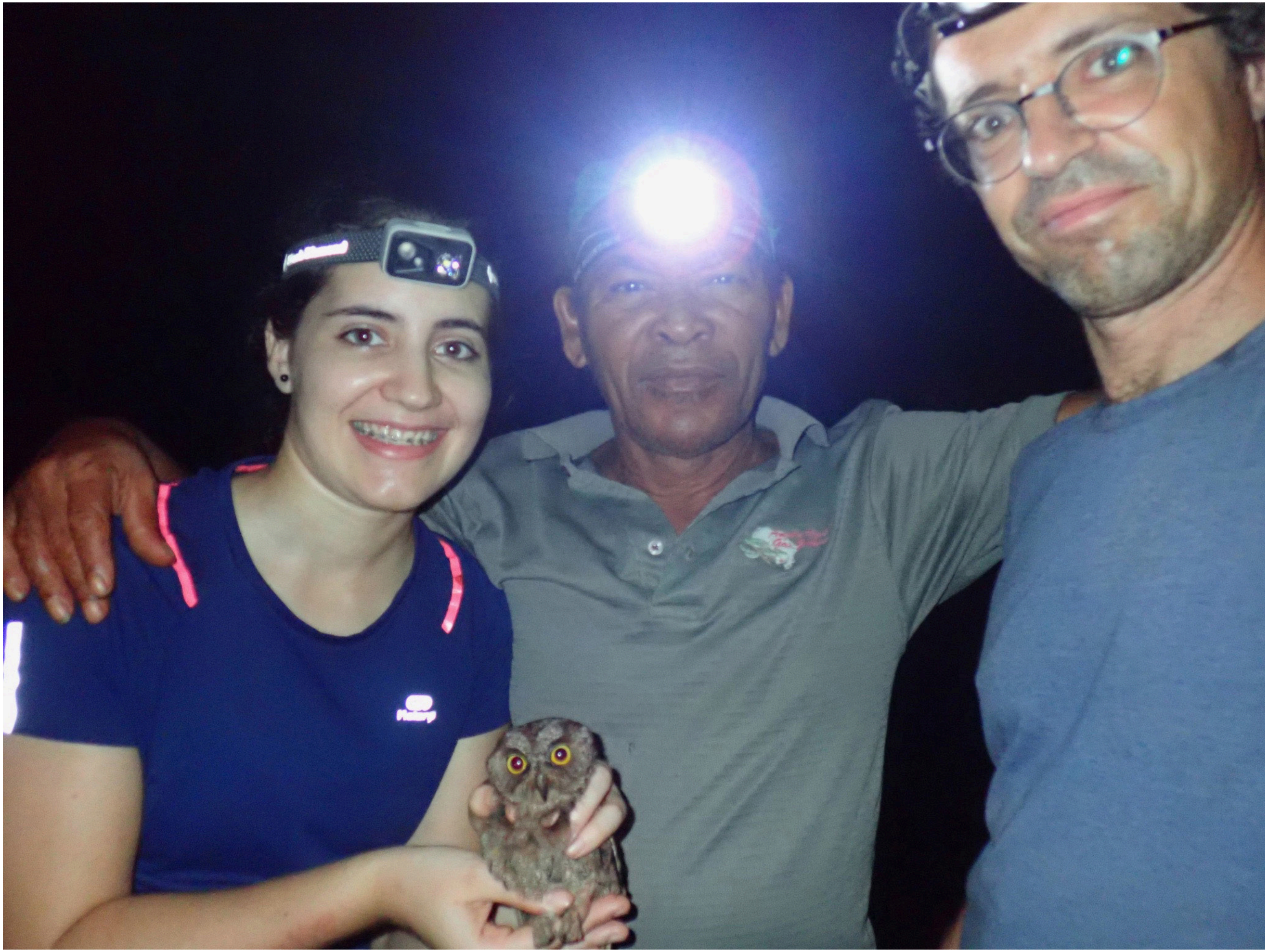

Figure S3. Photograph taken at Boca do Inferno, Príncipe Island, January 27, 2019, showing the Principe Scops-Owl *Otus bikegila* sp. nov., the two first authors of the paper (BF on the left and MM on the right), and Ceciliano do Bom Jesus, known as 'Bikegila' (centre), who started the 20-year saga that led to this discovery, and in honour of whom the new species was named (see 'Etymology').
